# Before-treatment body mass index as a prognostic factor in cancer patients receiving immune checkpoint inhibitors: a systematic review and dose-response meta-analysis

**DOI:** 10.3389/fimmu.2026.1921470

**Published:** 2026-09-24

**Authors:** Siyu Sun, Junhao Zhang, Yan Li, Zhijun Li, Yuxin He, Jing Ni, Erkang Zhao, Yanbing Xu

**Affiliations:** Department of Epidemiology, School of Public Health, Beihua University, Jilin, Jilin, China

**Keywords:** BMI, immune-related adverse events, immunotherapy, meta-analysis, obesity, overweight, prognosis, systematic review

## Abstract

**Background:**

Pretreatment body mass index (BMI) has been associated with outcomes during immune checkpoint inhibitor (ICI) therapy, but study-specific categories and incomplete adjustment complicate interpretation. We evaluated associations with efficacy and toxicity across tumor types and BMI definitions.

**Methods:**

We searched the Cochrane Library, Embase, PubMed, and Web of Science from inception through November 30, 2025. Random-effects models pooled hazard ratios (HRs) for overall survival (OS) and progression-free survival (PFS), and odds ratios (ORs) for objective response rate (ORR) and immune-related adverse events (irAEs). Adjusted estimates defined the primary analyses. We also evaluated category-specific contrasts, clinical subgroups, cutoff meta-regression, and linear dose-response associations per 5-kg/m^2^ increase in BMI.

**Results:**

Sixty-one studies involving 46,572 patients were included. Higher BMI was associated with a lower adjusted hazard of death (HR = 0.74, 95% CI: 0.67-0.82, *P* < 0.001; *I*^2^ = 70.6%; 95% prediction interval: 0.48-1.13) and a lower adjusted hazard of progression or death (HR = 0.78, 95% CI: 0.69-0.89, *P* < 0.001; *I*^2^ = 60.3%; 95% prediction interval: 0.47-1.32). However, both prediction intervals included the null value. Category-specific analyses showed that obesity, but not overweight, was associated with improved OS and PFS relative to normal weight. BMI cutoff did not significantly moderate either endpoint. Per 5-kg/m^2^ increase in BMI, adjusted PFS improved (HR = 0.87, 95% CI: 0.81-0.95, *P* < 0.001; *I*^2^ = 47.7%), whereas OS did not (HR = 0.91, 95% CI: 0.81-1.01, *P*>0.05; *I*^2^ = 64.0%). Adjusted associations with ORR and any-grade irAEs were not significant, and evidence for grade 3 or higher irAEs was insufficient.

**Conclusion:**

In this systematic review and meta-analysis, higher pretreatment BMI was associated with longer adjusted OS and PFS in patients receiving ICIs, with the most consistent favorable association observed for obesity. However, the prediction intervals included the null value, and associations with treatment response and irAEs remained inconsistent, warranting cautious interpretation.

## 1 Introduction

Immune checkpoint inhibitors (ICIs) targeting cytotoxic T-lymphocyte-associated protein 4 (CTLA-4), programmed cell death protein 1 (PD-1), or programmed death-ligand 1 (PD-L1) have transformed the treatment of multiple malignancies (1–3). Despite these therapeutic advances, primary resistance remains common, and some initial responders subsequently develop acquired resistance (4, 5). At the same time, immune-related adverse events (irAEs) also differ considerably in occurrence and severity, reflecting the variable systemic consequences of immune activation (6). Tumor-centered biomarkers, including PD-L1 expression, immune contexture, and tumor mutational burden, capture complementary biological features but remain imperfect when used alone (7). These limitations highlight the potential value of clinically accessible host factors, which may provide additional context for interpreting efficacy and toxicity.

Among these host factors, body mass index (BMI) is inexpensive, routinely recorded, and available before treatment in most oncology settings (8). Its relation to cancer outcomes is complex. Excess body weight contributes to the incidence and population burden of several malignancies (9), yet higher BMI has been associated with longer survival after some cancer diagnoses (10). This apparent obesity paradox has particular relevance to immunotherapy because adiposity can alter inflammatory, endocrine, and immune pathways (11). Yet BMI remains an imprecise measure, because it is only a ratio of weight to height and cannot distinguish visceral from subcutaneous adiposity, skeletal muscle reserve, edema, or recent catabolic weight loss. A favorable association may therefore reflect nutritional reserve, immunometabolic biology, or confounding by disease severity and body composition. Indeed, adjustment for cancer type, weight loss, and reduced skeletal muscle can substantially attenuate the apparent survival advantage (12).

In this context, existing evidence supports an association between higher BMI and survival during ICI therapy, but its interpretation remains unsettled (13, 14). Early aggregate-data meta-analyses reported improved overall survival (OS) and progression-free survival (PFS), although they included relatively few studies and combined non-equivalent BMI definitions. At the individual-patient level, a meta-analysis associated overweight or obesity with longer survival but found no stronger association for obesity than for overweight (15). Even so, other syntheses emphasized inconsistency across tumor types, BMI thresholds, and toxicity outcomes (16). More recent tumor-specific analyses have also suggested that estimates may vary with cancer type and treatment composition (17, 18). These differences are clinically important because pooled higher-versus-lower BMI comparisons can obscure distinct effects of underweight, overweight, and obesity. Methodologically, this uncertainty may be compounded when adjusted and unadjusted estimates are combined or when correlated contrasts from one cohort are treated as independent (19).

To address these uncertainties, we conducted an updated systematic review and meta-analysis of pretreatment BMI among patients receiving ICIs. To distinguish adjusted from crude associations, adjusted estimates were designated as the primary evidence, and unadjusted estimates were examined separately. We then evaluated OS, PFS, objective response rate (ORR), and irAEs using complementary higher-versus-lower, category-specific, cutoff meta-regression, and linear dose-response analyses. Formal subgroup tests, influence analyses, and outcome-specific assessments of small-study effects were used to examine robustness and heterogeneity. This framework was designed to determine which features of the reported BMI association remained consistent across complementary analytical approaches and which depended on contrast definition, cutoff selection, or covariate adjustment.

## 2 Methods

### 2.1 Search strategy

This research was performed according to the Preferred Reporting Items for Systematic Reviews and Meta-Analyses (PRISMA) (20) and Meta-analysis of Observational Studies in Epidemiology (MOOSE) (21) reporting guidelines. We searched the Cochrane Library, Embase, PubMed, and Web of Science for original publications from database inception until November 30, 2025. The following search terms were used for the initial search: cancer, body mass index, overweight, obesity, PD-1, PD-L1, CTLA-4, nivolumab, pembrolizumab, atezolizumab, avelumab, durvalumab, sintilimab, ipilimumab, and tremelimumab. Additionally, we manually screened the reference lists of relevant studies.

### 2.2 Selection criteria

Eligible reports were prospective or retrospective cohorts, including cohort analyses derived from clinical-trial datasets. Participants had cancer, received ICIs, and had pretreatment BMI assessed in relation to at least one eligible outcome. Studies had to report, or permit calculation of, HRs for OS or PFS, or ORs for ORR or irAEs. Only English-language reports with extractable effect estimates or 2 × 2 data were included. We excluded non-human studies, case reports, editorials, commentaries, letters, reviews, meta-analyses, duplicate populations, and reports without usable outcome data.

### 2.3 Data extraction and quality assessment

Two reviewers (J.H.Zhang and S.Y.Sun) independently screened titles, abstracts, and full texts, and then extracted the data. Extracted study characteristics included author, publication year, design, location, sample size, follow-up, cancer type, ICI regimen, and BMI definition. Outcome data included OS, PFS, ORR, any-grade irAEs, and grade 3 or higher irAEs. We also recorded cohort identifiers, analytic sample size, comparison direction, adjustment status, and covariates.

HRs and 95% CIs were extracted for OS and PFS. ORs and 95% CIs, or the 2 × 2 data required to calculate them, were extracted for ORR and irAEs. Adjusted and unadjusted estimates were retained separately when both were available. Independent cohorts within one publication were treated as separate estimates. For overlapping participants, we selected the prespecified BMI contrast and the most comprehensively adjusted eligible model.

Risk of bias was assessed with the Newcastle-Ottawa Scale (NOS) because the eligible evidence comprised observational BMI-outcome associations (22). Domain-level judgments and total scores were recorded. Scores of 0-4, 5-7, and 8–9 were classified as low, moderate, and high quality, respectively.

### 2.4 BMI definition and classification

BMI was defined as weight in kilograms divided by height in meters squared. World Health Organization categories were used as a clinical reference: underweight (<18.5 kg/m^2^), normal weight (18.5 to <25.0 kg/m^2^), overweight (25.0 to <30.0 kg/m^2^), and obesity (≥30.0 kg/m^2^). Participant-level data were unavailable, so the main analysis retained each study’s original BMI definition. Higher BMI was the exposure and lower BMI the reference in study-defined high-versus-low analyses.

Secondary analyses compared overweight with normal weight, obesity with normal weight, underweight with normal weight, and overweight/obesity with normal/underweight. The numeric cutoff from each study was extracted for meta-regression of threshold-related heterogeneity.

### 2.5 Statistical analysis

Effect estimates were analyzed on the logarithmic scale. HRs were used for OS and PFS, and ORs for ORR and irAEs. Comparison directions were harmonized as higher versus lower BMI. Reverse estimates were inverted using 1/estimate, 1/upper CI, and 1/lower CI. Category-specific analyses retained the direction specified by each named contrast. Standard errors were calculated from 95% CIs as [ln(upper CI) - ln(lower CI)]/(2 × 1.96).

Adjusted estimates constituted the primary evidence, while unadjusted estimates were pooled separately. Each model included one independent cohort-level estimate. Independent cohorts from one publication were retained, but correlated estimates from the same participants were not entered simultaneously.

Inverse-variance random-effects models were fitted, with between-study variance (τ^2^) estimated by restricted maximum likelihood (23). Normal-approximation 95% CIs were used for principal analyses, ORR, irAEs, and clinical subgroups. Knapp-Hartung inference was used for category-specific models and cutoff meta-regression (24). Heterogeneity was evaluated using Cochran’s Q, τ^2^, and *I*^2^. *I*^2^ values below 25%, from 25% to 50%, and above 50% indicated low, moderate, and substantial heterogeneity. For the primary adjusted OS and PFS models and the linear dose-response models, 95% prediction intervals were calculated when estimable. Leave-one-out analyses assessed the influence of individual studies.

Prespecified subgroup analyses evaluated cancer type, study region, and ICI class: anti-PD-1/PD-L1, anti-CTLA-4, or their combination. Differences were assessed with omnibus moderator tests rather than comparisons of within-subgroup significance. Subgroups containing fewer than three estimates were considered exploratory. Separate univariable meta-regressions evaluated numeric BMI cutoff as a moderator of adjusted OS and PFS. The cutoff was centered at 25 kg/m^2^, and coefficients were reported as ratios of HRs per 5-kg/m^2^ higher cutoff. Models used restricted maximum likelihood, Knapp-Hartung inference, and leave-one-out assessment.

Linear dose-response meta-analysis estimated the HR per 5-kg/m^2^ increase in BMI for OS and PFS. Generalized least-squares trend estimation accounted for correlated category-specific log HRs (25). Reported means or medians were assigned to each category; otherwise, category midpoints were used. Open-ended intervals were estimated using the width of the adjacent category. When the lowest category was not the reference, estimates were re-expressed with the Hamling method (26). Within-study covariance was reconstructed with Hamling-based generalized least squares when sufficient data were available. Otherwise, slopes were derived from continuous estimates, two-group contrasts, or inverse-variance weighted least squares. Independent cohorts from one publication were first combined using a fixed-effect model. Publication-level slopes were then pooled with a random-effects model. Only adjusted publication-level estimates were included in the linear dose-response analyses.

Reported ORs for ORR and irAEs were pooled directly; otherwise, ORs were calculated from 2×2 tables (27). No continuity correction was required because no included table contained a zero cell. Potential small-study effects were evaluated by visual inspection of funnel plots and Egger’s regression test when at least ten estimates were available. Egger’s test was interpreted as an assessment of funnel-plot asymmetry rather than direct evidence of publication bias. Analyses used R version 4.5.2 with the metafor and dosresmeta packages. All tests were two-sided.

## 3 Results

### 3.1 Systematic search

The database search identified 2,454 records, of which 938 remained after deduplication. Screening excluded 877 records, and 61 reports underwent full-text assessment. After full-text assessment and citation searching, 61 studies were included in the meta-analysis (12, 28–87) (**Figure 1**).

**Figure 1:**
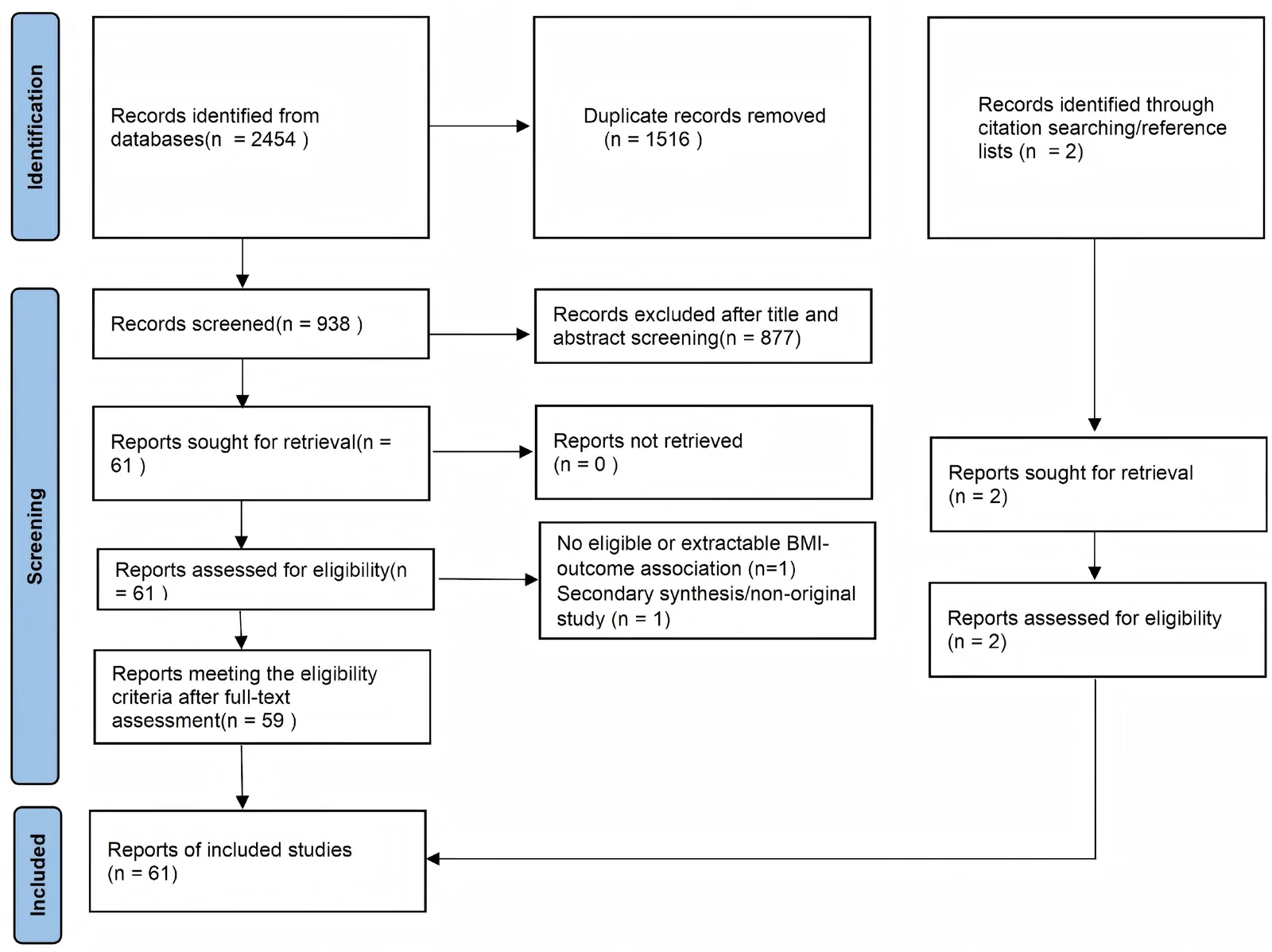
PRISMA flow diagram of the literature search and study selection process.

### 3.2 Study characteristics

The 61 studies comprised 55 retrospective and 6 prospective cohorts. Fourteen were conducted in Europe, 23 in Asia, 16 in the United States, 1 in Australia, and 7 across multiple regions. Six studies enrolled patients with melanoma, 18 with non-small cell lung cancer, 8 with renal cell carcinoma, and 5 with hepatocellular carcinoma. The remaining 24 evaluated other specific malignancies or mixed populations.

Forty-nine studies evaluated only anti-PD-1/PD-L1-based ICIs. Eleven included anti-PD-1/PD-L1 and anti-CTLA-4 agents in separate or combination regimens, and one did not specify ICI class. Three studies analyzed BMI continuously. Other studies used categorical definitions with boundaries from 18.5 to 35 kg/m^2^; 25 and 30 kg/m^2^ were most common. The NOS classified 35 studies as high quality and 26 as moderate quality. **Table 1** summarizes study characteristics and NOS ratings.

**Table 1:** Characteristics and NOS quality assessment of the included studies.

Study	Country/setting	Design	Cancer type	ICI treatment	N	Male, n (%)	Age, years	BMI categories, kg/m^2^	Outcomes	Follow-up, months	NOS
Heidelberger, 2017 (46)	France	Retrospective	Melanoma	Nivo, Pembro	68	36 (53.0)	65 (22-91)	<18.5, 18.5-25, 25-30, >30	ORR, irAEs	6 (0.5-18)	7
Kondo, 2018 (56)	Japan	Retrospective	Melanoma	Nivo	39	24 (61.5)	65 (28-84)	<20, ≥20	OS, PFS, ORR	11.9 (5.0-36.1)	8
McQuade, 2018 (64)	Multicenter	Retrospective	Melanoma	Anti-CTLA-4, Anti-PD-1/PD-L1	538	349 (64.9)	23-88	<18.5, 18.5-24.9, 25.0-29.9, ≥30	OS, PFS, ORR	38.4 (35.6-40.4)	7
Dumenil, 2018 (43)	France	Retrospective	NSCLC	Nivo	67	46 (69.0)	68.5 (60-77)	<18.5, ≥18.5	OS, PFS	NR	8
Shiroyama, 2018 (75)	Japan	Retrospective	NSCLC	Nivo	201	135 (67.2)	68 (27-87)	<18.5, 18.5-24.99, ≥25	PFS, ORR	12.4	7
Qi, 2019 (71)	China	Retrospective	Mixed cancers	Anti-PD-1	85	42 (49.4)	NR	<24, ≥24	OS, PFS, ORR	11.0 (1.5-28.8)	7
Martini, 2019 (60)	USA	Retrospective	Mixed cancers	Immunotherapy	90	53 (59.0)	NR	<25, 25-30, ≥30	OS, PFS	NR	7
Cortellini, 2019 (38)	Italy	Retrospective	Mixed cancers	Pembro, Nivo, Atezo	976	663 (67.9)	68 (24-92)	<18.5, 18.5-24.9, 25.0-29.9, ≥30.0	OS, PFS, ORR, irAEs	17.2	8
Giorgi, 2019 (40)	Italy	Prospective	RCC	Nivo	313	235 (75.1)	65 (40-84)	<18.5, 18.5-25, 25-30, ≥30	OS, ORR	NR	8
Donnelly, 2019 (42)	USA	Prospective	Melanoma	Anti-CTLA-4, Anti-PD-1, Anti-CTLA-4+Anti-PD-1	423	267 (63.1)	NR	<25, 25-29.9, ≥30	OS, PFS, ORR, irAEs	36.3 (1.4-173.3)	7
Katayama, 2019 (54)	Japan	Retrospective	NSCLC	Pembro, Nivo, Atezo	35	24 (68.6)	70 (48-83)	≤20, 20-25, >25	OS, PFS, ORR	NR	8
Kichenadasse, 2019 (55)	Australia	Prospective	NSCLC	Atezo	1,434	890 (62.0)	64 (57-70)	<18.5, 18.5-24.9, 25.0-29.9, ≥30.0	OS, PFS, irAEs	NR	8
Labadie, 2019 (58)	Multicenter	Retrospective	RCC	Nivo, Pembro, Atezo	90	65 (72.2)	66 (17-92)	<18.5, 18.5-25, 25-30, ≥30	OS, PFS, irAEs	NR	8
Magri, 2019 (59)	Italy	Retrospective	NSCLC	Nivo	46	28 (60.87)	65 (39-85)	Continuous	OS	22	8
Popinat, 2019 (70)	France	Retrospective	NSCLC	Nivo	55	41 (75.0)	63.5 (37.8-82.4)	<24.7, ≥24.7	OS, ORR, irAEs	NR	7
Wang, 2019 (81)	USA	Retrospective	Mixed cancers	Anti-PD-1/PD-L1	250	114 (45.6)	61.7 (23-91)	<30, ≥30	OS, PFS, irAEs	NR	8
Naik, 2019 (67)	USA	Retrospective	Melanoma	Pembro, Nivo, Nivo+Ipi	139	79 (56.8)	NR	18.5-25, 25-35, ≥35	OS, PFS	25.3 (17.4-30.3)	8
Cortellini, 2020 (38)	Multicenter	Retrospective	NSCLC	Pembro	962	635 (66.0)	70.1 (28-92)	<18.5, 18.5-25, 25-30, ≥30	OS, PFS, ORR	14.6	8
Nitipir, 2020 (69)	Romania	Retrospective	Mixed cancers	Nivo	57	44 (77.2)	62.0 (39-80)	<18.5, 18.5-24.9, >25	PFS, ORR	12	7
Rutkowski, 2020 (73)	Multicenter	Retrospective	Melanoma	Anti-PD-1, Ipi	446	272 (61.0)	65.5 (54.3-73.7)	<18.5, 18.5-24.9, 25-29.9, ≥30	OS, PFS	38.8	8
Ichihara, 2020 (47)	Japan	Retrospective	NSCLC	Pembro, Nivo, Atezo	513	406 (79.1)	28-93	<22, ≥22, <18.5, 18.5-24.9, 25-29.9, ≥30	OS, PFS, ORR	NR	6
Rogado, 2020 (72)	Spain	Retrospective	Mixed cancers	Nivo, Pembro	132	95 (71.9)	69 (32-86)	<18.5, 18.5-24.9, 25-29.9, ≥30	PFS, ORR, irAEs	6 (0.5-32)	8
Minami, 2020 (65)	Japan	Retrospective	NSCLC	Anti-PD-1/PD-L1	74	48 (64.9)	61.3-75	<18.5, ≥18.5	OS, PFS, ORR	NR	7
Ahmed, 2021 (28)	USA	Prospective	Mixed cancers	Nivo, Pembro, Durva, Ipi	297	177 (60.0)	68.0 (27-92)	<25, ≥25, <18.5, 25-29.9, ≥30	OS, PFS	10.77	7
Bartl, 2021 (33)	Austria	Retrospective	Mixed cancers	Pembro	36	0 (0.0)	56.5 (45.8-65.8)	<25, ≥25	OS, PFS, ORR, irAEs	NR	8
Collet, 2021 (37)	France	Retrospective	Mixed cancers	Nivo, Pembro, Atezo, Durva, Ave, Ipi, Treme	272	174 (64.0)	61.4	<18.5, 18.5-24.9, 25.0-29.9, ≥30, <25, ≥25	OS, irAEs	10	7
Imai, 2021 (49)	Japan	Retrospective	NSCLC	Pembro	142	117 (82.4)	70.0 (47-86)	<21.4, ≥21.4	OS, PFS, ORR	15.7	8
Akce, 2021 (29)	USA	Retrospective	HCC	Anti-PD-1	57	44 (77.2)	66	<25, ≥25	OS, PFS	6	7
Esposito, 2021 (44)	Italy	Retrospective	Mixed cancers	Anti-PD-1/PD-L1	153	62 (40.5)	58 (32-80)	<18.5, 18.5-24.9, 25.0-29.9, ≥30.0	OS, PFS, ORR, irAEs	27.9	7
Zhang, 2021 (85)	USA	Retrospective	Lung cancer	Atezo, Nivo, Pembro	220	100 (45.5)	66.5 (36-92)	<25, ≥25	OS, PFS, ORR, irAEs	11.4	8
Chen, 2022 (15)	China	Retrospective	HCC	Anti-PD-1	101	82 (81.2)	50	≤24.9, >24.9	PFS, ORR, irAEs	NR	8
Ged, 2022 (45)	USA	Retrospective	RCC	Nivo, Ipi	205	152 (74.0)	63.0 (40-90)	<18.5, 18.5-25, 25-30, ≥30	OS, PFS, ORR	31.2	7
Imai, 2022 (50)	Japan	Retrospective	NSCLC	Nivo	99	77 (77.8)	69.0 (31-80)	<22.1, ≥22.1	OS, PFS, ORR	12.7 (0.3-63.7)	7
Joshi, 2022 (53)	USA	Retrospective	NSCLC	Pembro	136	67 (49.3)	68 (61-74)	Continuous	OS	NR	7
Wang, 2023 (79)	China	Retrospective	RCC	Anti-PD-1	224	159 (71.0)	55 (46-63)	<18.5, 18.5-23.9, 24.0-27.9, ≥28.0	OS, PFS	10.1 (6.7-16.4)	7
Tateishi, 2022 (77)	Japan	Retrospective	NSCLC	Nivo, Pembro	324	235 (72.5)	63 (37-83)	<25, ≥25	OS, PFS, ORR	24	8
Wasamoto, 2023 (82)	Japan	Retrospective	SCLC	Atezo	84	70 (83.3)	71.0 (43-89)	<22, ≥22	OS, PFS, ORR	12.9 (1.5-24.4)	8
Incorvaia, 2023 (51)	Italy	Prospective	MCC	Ave	32	17 (53.1)	75.0 (43-89)	18.5-24.9, 25-29.9, ≥30.0	OS, PFS, ORR	NR	8
Jain, 2023 (52)	USA	Retrospective	NSCLC	Nivo, Pembro, Atezo	200	91 (46.0)	67.0 (36-93)	Continuous	OS, PFS, ORR	32	8
Vithayathil, 2023 (78)	Multicenter	Retrospective	HCC	Atezo, Beva	191	161 (84.3)	68.4 (61.8-75.2)	<18.5, 18.5-25, 25-30, ≥30	OS, PFS, ORR, irAEs	NR	8
Wu, 2023 (83)	China	Retrospective	Esophageal cancer	Camre, Pembro, Nivo, Sinti, Tisle	78	65 (83.3)	58.0 (46-87)	<18.8, ≥18.8	OS, PFS	NR	7
Zhang, 2023 (86)	China	Retrospective	HNSCC	Pembro	49	35 (71.4)	59.0 (34-79)	<18.5, 18.5-23.5, 23.5-27.5, ≥27.5	OS, PFS, ORR, irAEs	11.1	8
McManus, 2023 (63)	USA	Retrospective	RCC	Ipi, Nivo	99	73 (73.7)	62 (37-81)	18.5-25, 25-30, ≥30	OS, PFS, ORR	25.7	8
Miyamoto, 2023 (66)	Japan	Retrospective	HNSCC	Anti-PD-1	106	83 (78.3)	68 (21-88)	<20, ≥20	OS, PFS, ORR	NR	8
Antoun, 2023 (12)	France	Prospective	Mixed cancers	Pembro, Nivo	800	457 (57.0)	NR	<18.5, 18.5-24.9, 25-29.9, ≥30.0	OS	25.4 (14.5-39.6)	6
Santoni, 2023 (74)	Multicenter	Retrospective	RCC	Nivo+Ipi	675	495 (73.0)	64 (25-88)	≤25, >25	OS, PFS	18.1	7
Alam, 2024 (30)	USA	Retrospective	MCC	Pembro, Nivo, Ave, Nivo+Ipi	183	140 (76.5)	70.0 (19-91)	18.5-24.9, 25-29.9, ≥30	OS, PFS, ORR	27	8
Bernard, 2024 (34)	USA	Retrospective	Endometrial cancer	Pembro	44	0 (0.0)	64.1 (41-87)	<30, ≥30	OS, PFS, ORR	33 (5-61)	8
Gómez-Banoy, 2024 (87)	USA	Retrospective	Endometrial cancer	Pembro, Nivo, Durva	524	0 (0.0)	67.0 (30-94)	18.5-24.9, 25-29.9, ≥30	OS, PFS, irAEs	NR	8
Nie, 2024 (13)	Multicenter	Retrospective	NSCLC	Pembro, Penpu, Nivo	1,558	691 (44.4)	65 (30-94)	18.5-24.9, 25-29.9, ≥30	OS, PFS, ORR, irAEs	6-31	8
Tan, 2024 (76)	China	Retrospective	Oral tongue squamous cell carcinoma	Pembro	56	41 (73.2)	50 (23-79)	<24, ≥24	OS, PFS	15 (6-30)	7
Wang, 2024 (80)	China	Retrospective	HCC	Camre	126	80 (63.5)	60	<25, ≥25	OS, PFS, ORR	NR	8
Llerena, 2024	USA	Retrospective	HNSCC	Anti-PD-1	166	112 (67.1)	NR	<20, 20-24.9, 25-29.9, ≥30	OS, irAEs	NR	7
Ihara, 2024 (48)	Japan	Retrospective	NSCLC	Anti-PD-1/PD-L1	12,816	10,287 (80.3)	70.2	<18.5, 18.5-24.9, 25-29.9, ≥30.0	OS	NR	6
Şen, 2024	Turkey	Retrospective	Mixed cancers	Anti-PD-1/PD-L1	113	95 (84.1)	61 (23-91)	<18.5, 18.5-24.9, 25.0-29.9, ≥30.0	OS, PFS, ORR, irAEs	NR	8
Yamamoto, 2024 (84)	Japan	Retrospective	HCC	Atezo+Bev	116	93 (80.2)	74	<25, ≥25	OS, PFS, ORR	15.3	8
Demir, 2025 (41)	Turkey	Retrospective	NSCLC	Nivo	300	274 (91.3)	66 (37-85)	18.5-24.9, ≥25	OS, PFS, ORR, irAEs	7.25 (0.10-72.83)	8
Mastrolonardo, 2025 (61)	USA	Retrospective	Mixed cancers	Anti-PD-1	18,434	4,559 (24.7)	NR	≥30, 20-24.9	OS, irAEs	NR	7
Çolak, 2025 (36)	Turkey	Retrospective	RCC	Nivo	126	93 (74.6)	62 (29-83)	<18.5, 18.5-25, 25-30, ≥30	OS	18.6 (3-66)	7
Krejčí, 2025 (57)	Czechia	Retrospective	NSCLC	Anti-PD-1/PD-L1	154	91 (59.1)	(61.6-74.4)	<25, ≥25	OS, PFS, ORR	NR	8
Anami, 2025 (32)	Japan	Retrospective	RCC	Nivo+Ipi	243	183 (75.3)	67	<25, ≥25	OS, PFS, ORR, irAEs	39	8

### 3.3 Association between BMI and overall survival

Across 38 publications contributing 42 independent cohort estimates, the primary multivariable-adjusted analysis showed that higher BMI was associated with a lower average hazard of death than lower BMI (HR = 0.74, 95% CI: 0.67-0.82, *P* < 0.001; *I*^2^ = 70.6%; 95% prediction interval: 0.48-1.13). Because the prediction interval crossed the null value, the magnitude and potentially the direction of the association may vary across comparable clinical settings (**Figure 2**).

**Figure 2:**
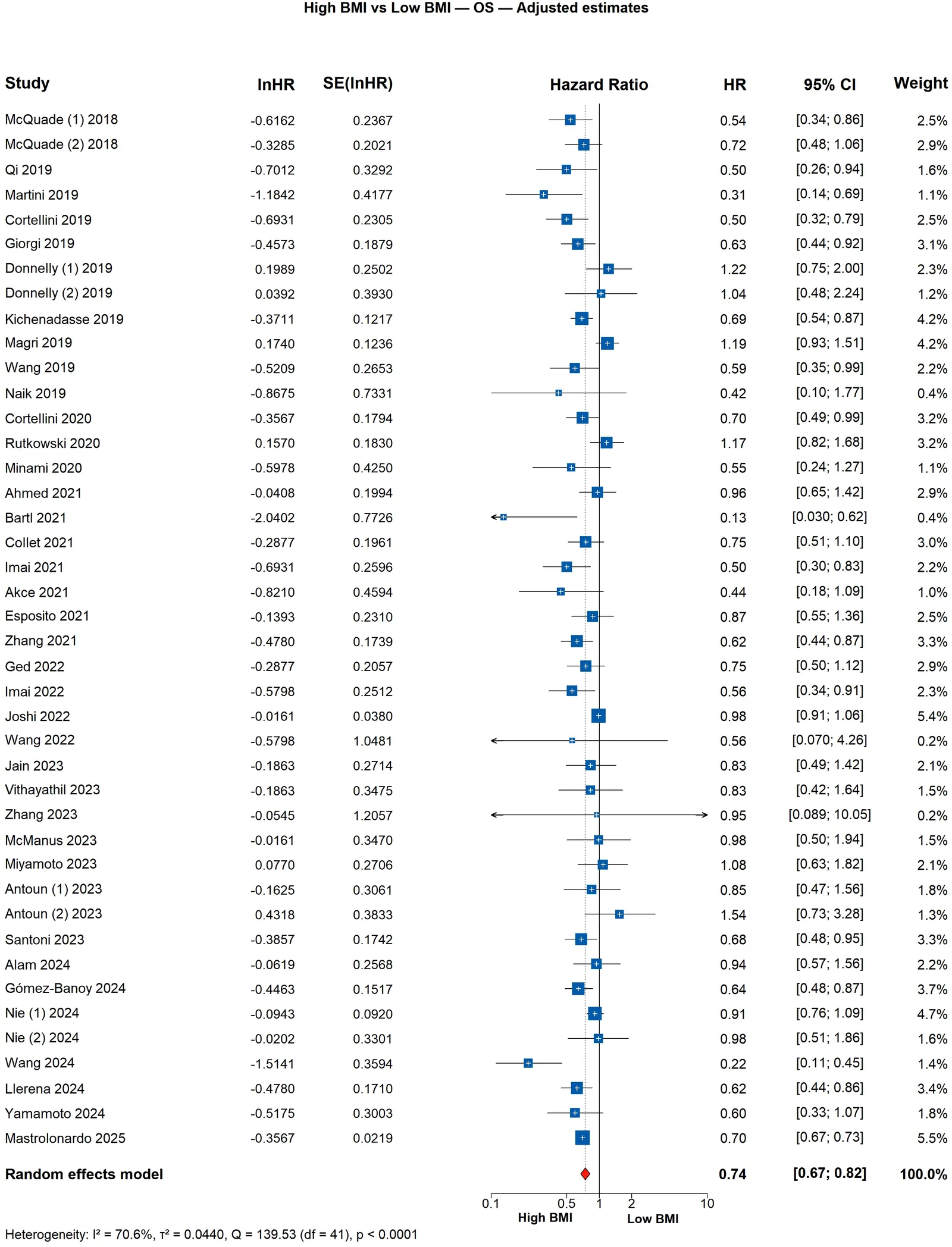
Forest plot of adjusted HRs for OS comparing patients with high versus low BMI.

### 3.4 Association between BMI and progression-free survival

Thirty-one publications contributed 34 independent adjusted cohort estimates for PFS. Higher BMI was associated with a lower average hazard of progression or death (HR = 0.78, 95% CI: 0.69-0.89, *P* < 0.001; *I*^2^ = 60.3%; 95% prediction interval: 0.47-1.32). As for OS, the prediction interval included the null value, suggesting that this association may not be observed consistently across all comparable populations or treatment settings (**Figure 3**).

**Figure 3:**
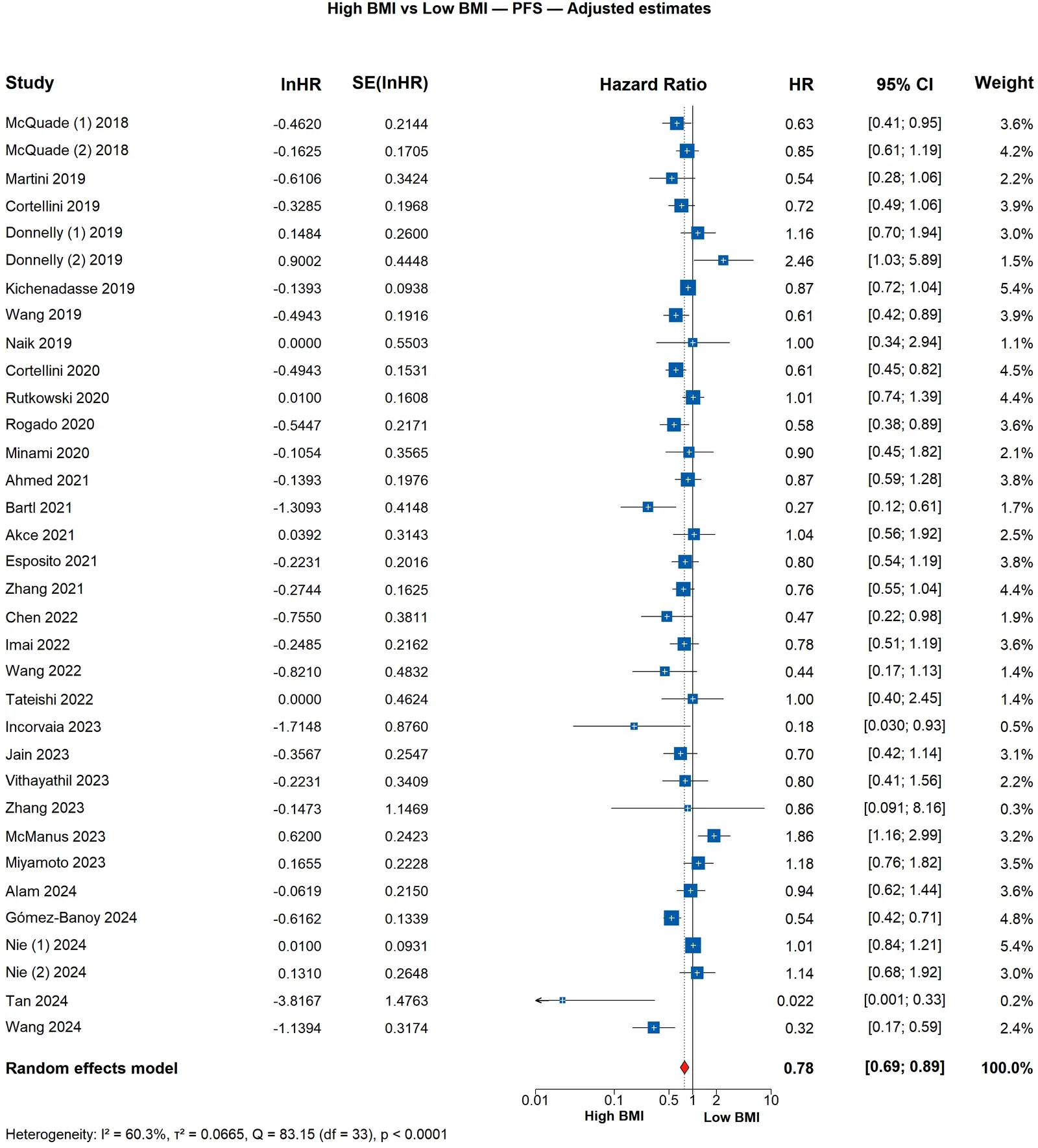
Forest plot of adjusted HRs for PFS comparing patients with high versus low BMI.

### 3.5 Meta-regression of BMI cutoff values

The cutoff meta-regression included 17 publications for adjusted OS and 15 publications for adjusted PFS; each publication contributed one cohort estimate. Seven BMI cutoffs ranging from 18.5 to 30 kg/m^2^ were represented. The ratio of HRs per 5-kg/m^2^ higher cutoff was 1.10 for OS (95% CI: 0.80-1.51; moderator-test *P*>0.05; residual *I*^2^ = 29.4%) and 1.00 for PFS (95% CI: 0.63-1.57; moderator-test *P*>0.05; residual *I*^2^ = 65.0%) (**Supplementary Figures S1**, **S2**).

### 3.6 Associations of BMI categories with OS and PFS

The association with OS varied across BMI contrasts. Compared with normal weight, overweight showed no significant association with OS across nine publications contributing 12 cohort estimates (HR = 0.91, 95% CI: 0.76-1.10, *P*>0.05; *I*^2^ = 43.8%). In contrast, obesity was associated with a lower mortality hazard in nine publications contributing 12 estimates (HR = 0.75, 95% CI: 0.66-0.85, *P* < 0.001; *I*^2^ = 34.0%). No significant association was observed for underweight versus normal weight across five publications contributing six estimates (HR = 1.08, 95% CI: 0.74-1.58, *P*>0.05; *I*^2^ = 0.0%). Similarly, the analysis of 12 publications comparing overweight/obesity with normal/underweight favored the higher-BMI group (HR = 0.65, 95% CI: 0.52-0.81, *P* < 0.05; *I*^2^ = 47.6%) (**Supplementary Figure S3**).

Compared with normal weight, overweight was not significantly associated with PFS across seven publications contributing nine cohort estimates (HR = 0.91, 95% CI: 0.78-1.06, *P*>0.05; *I*^2^ = 27.6%). By contrast, obesity was associated with a lower hazard of progression or death in seven publications contributing nine estimates (HR = 0.74, 95% CI: 0.59-0.93, *P* < 0.05; *I*^2^ = 60.7%). The underweight comparison, based on three publications, was imprecise and showed no significant association (HR = 1.85, 95% CI: 0.47-7.35, *P*>0.05; *I*^2^ = 29.7%). A favorable association was likewise observed across ten publications comparing overweight/obesity with normal/underweight (HR = 0.73, 95% CI: 0.62-0.87, *P* < 0.05; *I*^2^ = 0.0%) (**Supplementary Figure S4**).

### 3.7 Dose-response meta-analysis

In the adjusted linear dose-response analysis, 13 publications contributed OS estimates. Each 5-kg/m^2^ increase in BMI was not significantly associated with OS (HR = 0.91, 95% CI: 0.81-1.01, *P*>0.05; *I*^2^ = 64.0%), and the 95% prediction interval crossed the null value (0.67-1.23) (**Figure 4**). By contrast, 11 publications contributed adjusted PFS estimates, and each 5-kg/m^2^ increase in BMI was associated with a lower hazard of progression or death (HR = 0.87, 95% CI: 0.81-0.95, *P* < 0.001; *I*^2^ = 47.7%), although the prediction interval also included the null value (0.72-1.05) (**Figure 5**).

**Figure 4:**
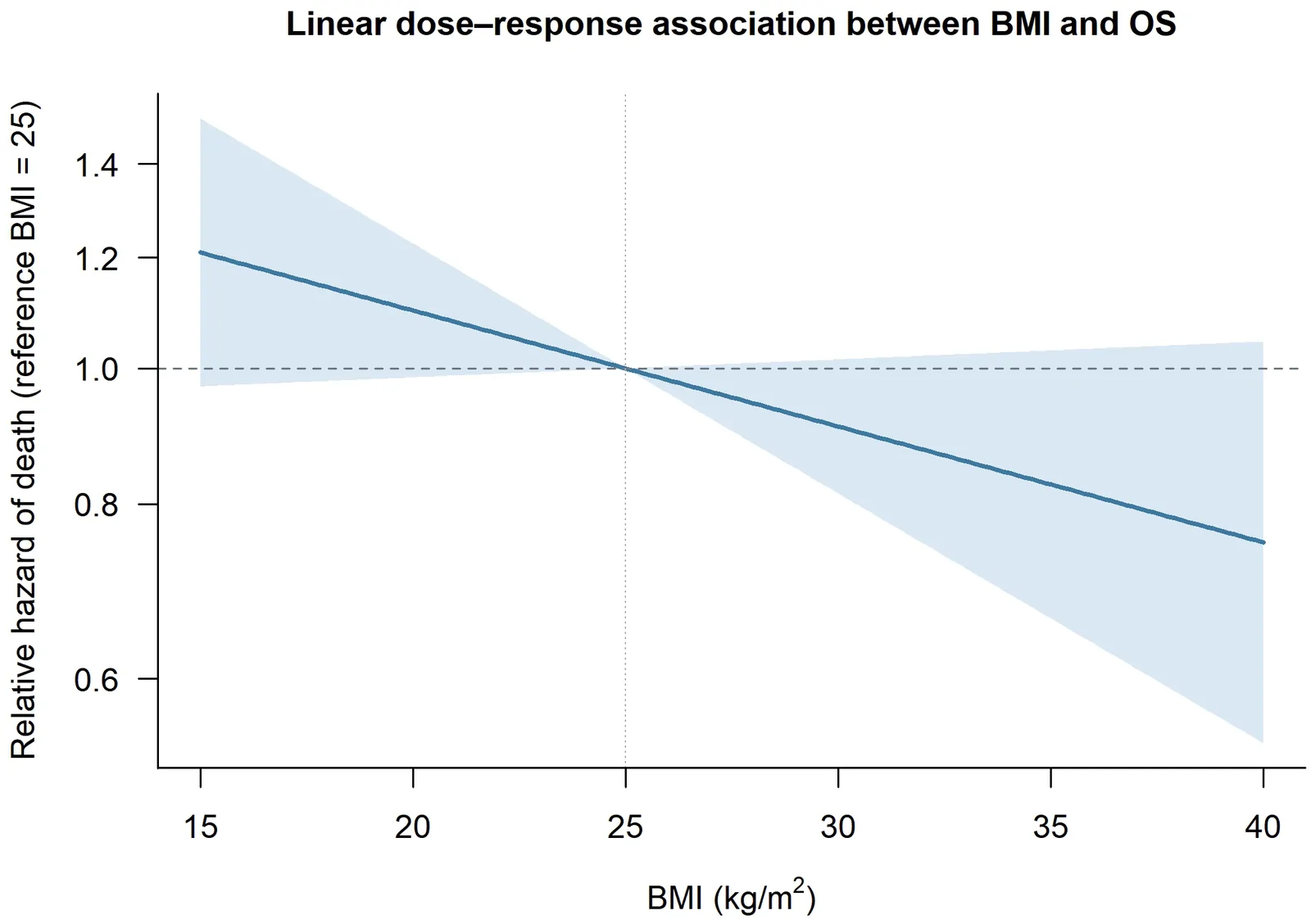
Linear dose–response association between BMI and OS.

**Figure 5:**
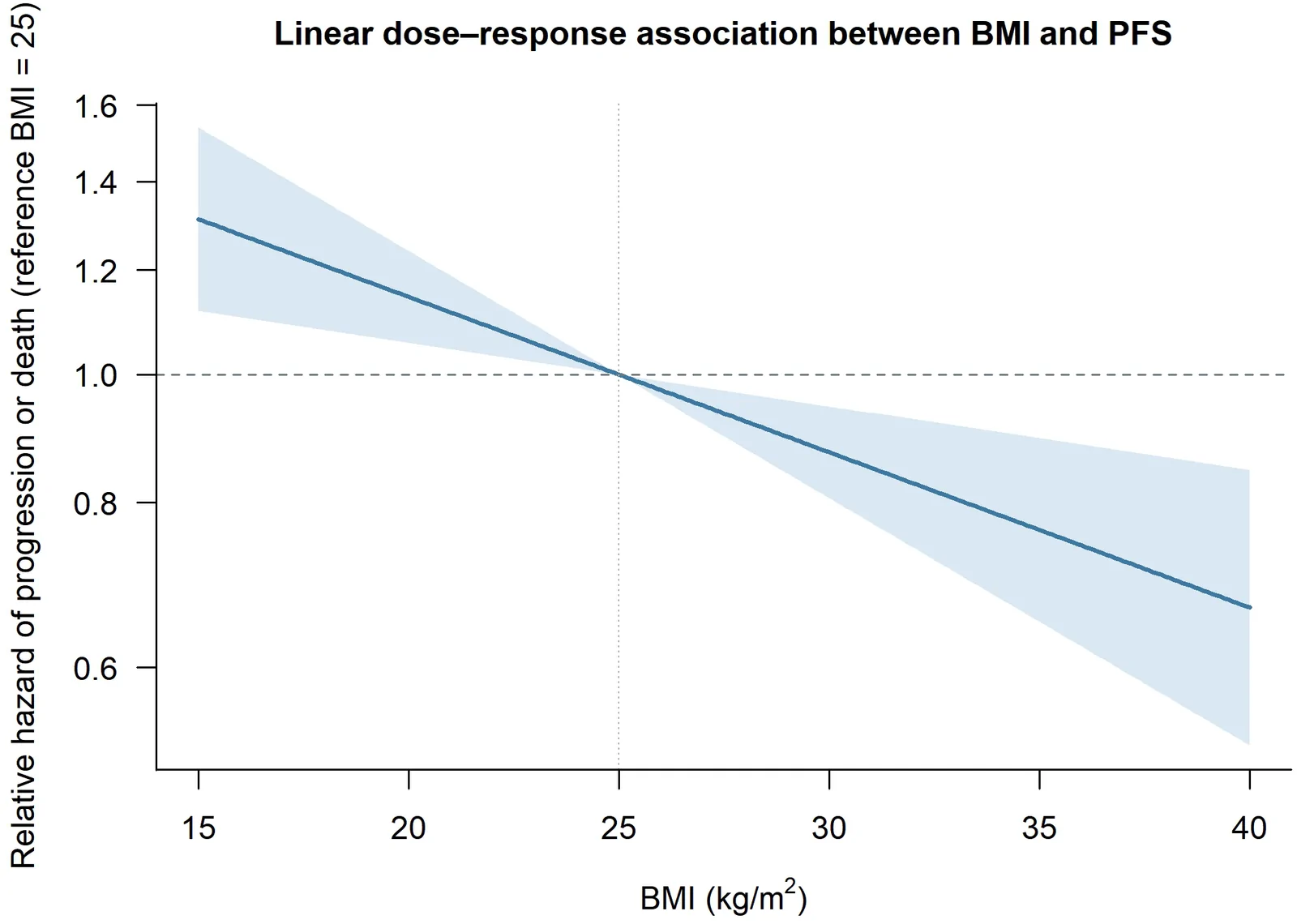
Linear dose–response association between BMI and PFS.

### 3.8 Associations of BMI with objective response rate and immune-related adverse events

Eight publications provided adjusted ORR estimates; the pooled association was not statistically significant (OR = 1.50, 95% CI: 0.94-2.41, *P*>0.05; *I*^2^ = 60.9%) (**Figure 6**). Adjusted evidence for any-grade irAEs was limited to two publications and likewise showed no clear association (OR = 1.00, 95% CI: 0.63-1.57, *P*>0.05; *I*^2^ = 58.9%) (**Figure 7**). Only one publication reported an adjusted estimate for grade 3 or higher irAEs (OR = 4.08, 95% CI: 0.95-17.56, *P*>0.05), precluding meta-analysis. Thus, unlike the survival endpoints, the adjusted response and toxicity evidence did not establish a consistent higher-BMI advantage or risk.

**Figure 6:**
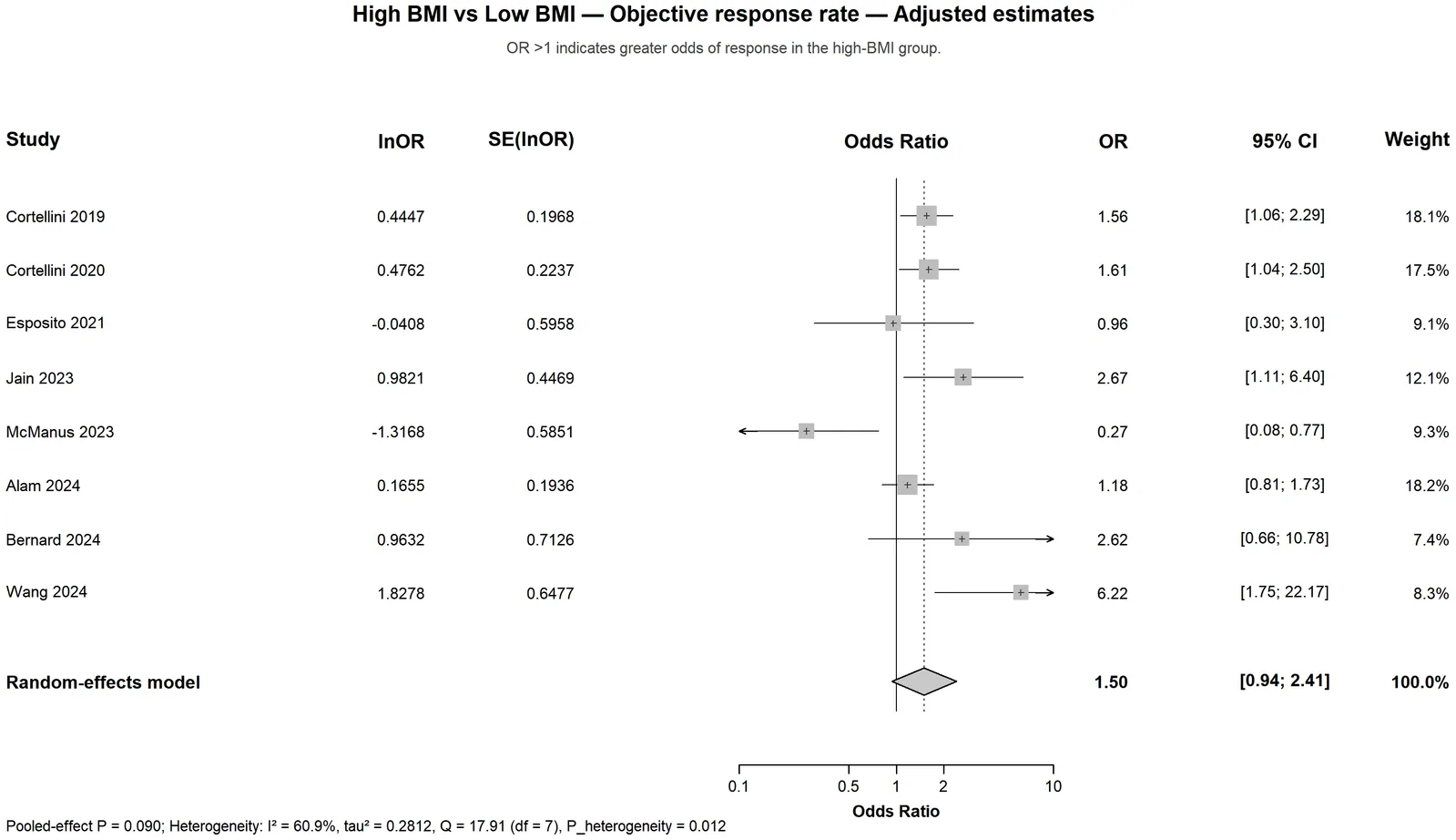
Forest plot of adjusted ORs for ORR comparing patients with high versus low BMI.

**Figure 7:**
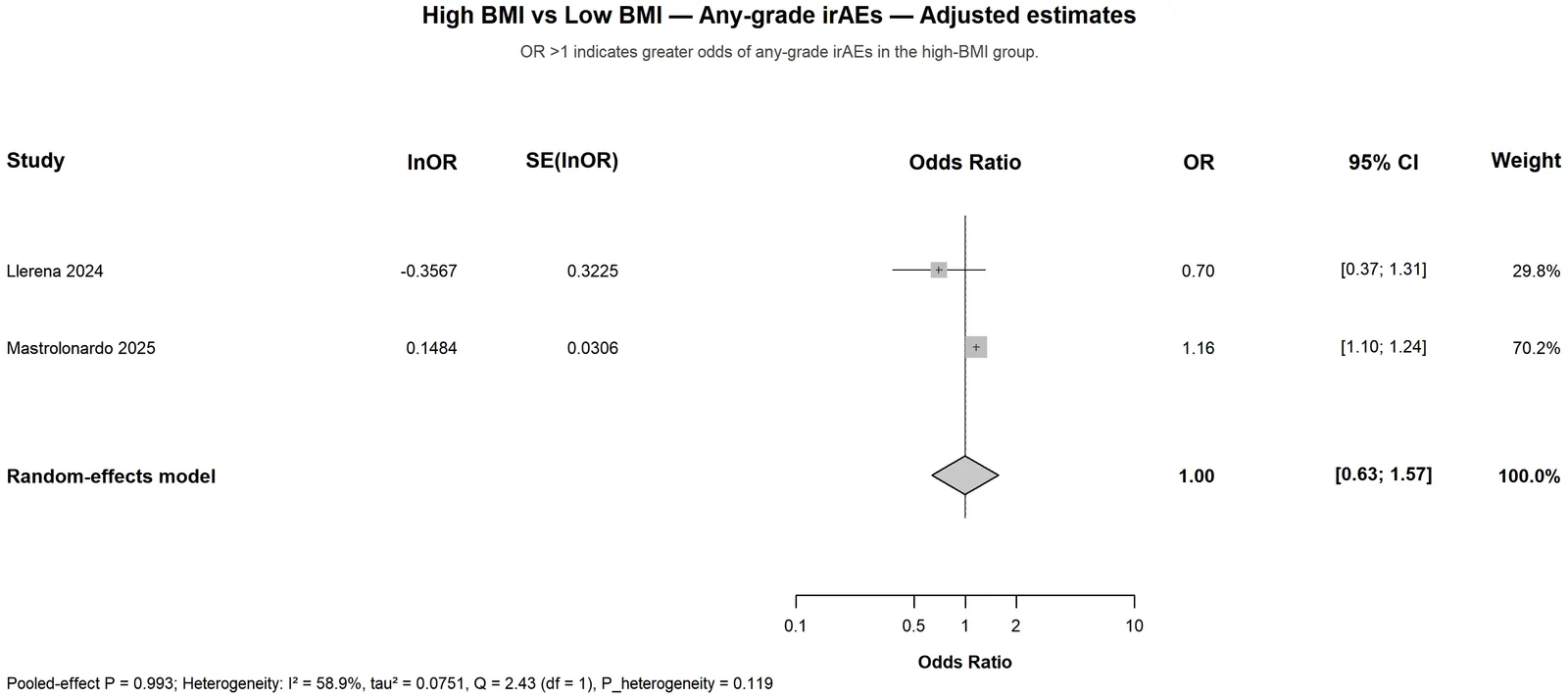
Forest plot of adjusted ORs for any-grade irAEs comparing patients with high versus low BMI.

### 3.9 Subgroup analyses

Subgroup analyses were performed across three dimensions: cancer type, study region, and ICI used. The adjusted subgroup analyses included 38 publications contributing 42 OS cohort estimates and 31 publications contributing 34 PFS estimates. Within the anti-PD-1/PD-L1 subgroup, higher BMI was associated with a lower hazard of death across 30 publications contributing 32 estimates (HR = 0.71, 95% CI: 0.63-0.79, *P* < 0.001; *I*^2^ = 75.1%) and a lower hazard of progression or death across 26 publications contributing 27 estimates (HR = 0.73, 95% CI: 0.64-0.84, *P* < 0.001; *I*^2^ = 59.4%). No subgroup differences were detected by cancer type, study region, or ICI used for OS or PFS (all *P* for interaction >0.05).

The adjusted ORR subgroup analysis included eight publications. A nominal subgroup difference was observed across cancer types (*P* for interaction <0.05), although this finding was exploratory because several subgroups contained only one or two estimates. No differences were detected by study region or ICI used (both *P* for interaction >0.05). For unadjusted any-grade irAEs, 12 publications contributed to the subgroup analyses, with no differences detected across the three dimensions (all *P* for interaction >0.05). Evidence for grade 3 or higher irAEs was insufficient for subgroup analysis. **Tables 2**, **3** provide the complete estimates. The subgroup forest plots are shown in **Supplementary Figures S5**-**S16**.

**Table 2:** Subgroup analyses of adjusted HRs for OS and PFS comparing high versus low BMI.

Outcome and analysis set	Subgroup analysis		*k*	HR (95% CI)	Heterogeneity		P for subgroup difference
	Moderator	Subgroup			I^2^ (%)	τ^2^	
OS (adjusted)	Overall	All estimates	42	0.74 (0.67-0.82)	70.6	0.0440	—
	Cancer type	Melanoma	6	0.86 (0.63-1.18)	52.7	0.0764	0.285
		NSCLC	11	0.77 (0.64-0.94)	70.0	0.0612	
		RCC	5	0.70 (0.57-0.86)	0.0	0.0000	
		HCC	4	0.47 (0.27-0.84)	61.3	0.2057	
		HNSCC	3	0.79 (0.48-1.30)	34.5	0.0867	
		Multiple/Other	13	0.70 (0.67-0.73)	35.7	0.0000	
	Study region	Asia	9	0.55 (0.40-0.75)	39.4	0.0990	0.142
		Europe	8	0.79 (0.59-1.04)	70.7	0.0941	
		USA	16	0.76 (0.66-0.87)	81.0	0.0298	
		Others	9	0.78 (0.67-0.91)	36.5	0.0185	
	ICIs used	Anti-PD-1/PD-L1	32	0.71 (0.63-0.79)	75.1	0.0509	0.329
		Anti-CTLA-4	2	0.81 (0.36-1.80)	82.1	0.2728	
		Anti-PD-1/PD-L1 + Anti-CTLA-4	8	0.85 (0.72-1.01)	1.4	0.0090	
PFS (adjusted)	Overall	All estimates	34	0.78 (0.69-0.89)	60.3	0.0665	—
	Cancer type	Melanoma	6	0.96 (0.74-1.25)	46.6	0.0422	0.067
		NSCLC	9	0.83 (0.71-0.98)	33.0	0.0207	
		RCC	2	0.96 (0.24-3.92)	85.9	0.8922	
		HCC	4	0.60 (0.35-1.02)	63.0	0.1861	
		HNSCC	2	1.17 (0.76-1.79)	0.0	0.0000	
		Multiple/Other	11	0.67 (0.57-0.78)	53.3	0.0182	
	Study region	Asia	9	0.65 (0.44-0.95)	60.7	0.1635	0.167
		Europe	5	0.59 (0.42-0.83)	51.3	0.0653	
		USA	12	0.88 (0.69-1.12)	68.0	0.1133	
		Others	8	0.86 (0.74-0.99)	42.5	0.0188	
	ICIs used	Anti-PD-1/PD-L1	27	0.73 (0.64-0.84)	59.4	0.0522	0.072
		Anti-CTLA-4	2	0.84 (0.46-1.53)	69.5	0.1295	
		Anti-PD-1/PD-L1 + Anti-CTLA-4	5	1.08 (0.82-1.42)	41.1	0.0426	

**Table 3:** Subgroup analyses of adjusted and unadjusted ORs for ORR and unadjusted ORs for any-grade irAEs comparing high versus low BMI.

Outcome and analysis set	Subgroup analysis		*k*	OR (95% CI)	Heterogeneity		P for subgroup difference
	Moderator	Subgroup			I^2^ (%)	τ^2^	
ORR (adjusted)	Overall	All estimates	8	1.50 (0.94-2.41)	60.9	0.2812	—
	Cancer type	NSCLC	2	1.79 (1.20-2.67)	2.4	0.0030	0.044
		RCC	1	0.27 (0.09-0.84)	NR	NR	
		HCC	1	6.22(1.75-22.14)	NR	NR	
		Multiple/Other	4	1.36 (1.05-1.76)	0.0	0.0000	
	Study region	Asia	1	6.22(1.75-22.14)	NR	NR	0.349
		Europe	2	1.49 (1.03-2.15)	0.0	0.0000	
		USA	4	1.22 (0.46-3.23)	72.6	0.7430	
		Others	1	1.61 (1.04-2.50)	NR	NR	
	ICIs used	Anti-PD-1/PD-L1	6	1.74 (1.34-2.25)	23.6	0.0000	0.057
		Anti-PD-1/PD-L1 + Anti-CTLA-4	2	0.62 (0.15-2.63)	82.7	0.9087	
ORR (unadjusted)	Overall	All estimates	22	1.32 (1.04-1.69)	55.4	0.1440	—
	Cancer type	NSCLC	7	1.44 (0.95-2.18)	48.7	0.1495	0.397
		RCC	4	0.98 (0.64-1.49)	43.4	0.0595	
		HCC	3	1.42 (0.81-2.48)	0.0	0.0000	
		HNSCC	1	0.72 (0.32-1.63)	NR	NR	
		Multiple/Other	7	1.65 (1.01-2.67)	49.3	0.1784	
	Study region	Asia	9	1.35 (0.92-1.98)	28.8	0.1169	0.696
		Europe	8	1.42 (0.95-2.12)	68.4	0.1763	
		USA	3	0.95 (0.31-2.94)	69.9	0.7314	
		Others	2	1.41 (0.84-2.39)	0.0	0.0000	
	ICIs used	Anti-PD-1/PD-L1	19	1.44 (1.12-1.85)	50.7	0.1255	0.136
		Anti-PD-1/PD-L1 + Anti-CTLA-4	3	0.84 (0.41-1.70)	59.2	0.2351	
Any-grade irAEs (unadjusted)	Overall	All estimates	12	1.50 (1.10-2.05)	76.1	0.1723	—
	Cancer type	NSCLC	1	1.34 (0.97-1.85)	NR	NR	0.515
		RCC	1	1.12 (0.63-1.99)	NR	NR	
		HCC	2	0.92 (0.54-1.55)	0.0	0.0000	
		HNSCC	1	2.48(0.49-12.57)	NR	NR	
		Multiple/Other	7	1.78 (1.16-2.73)	76.2	0.2013	
	Study region	Asia	3	1.09 (0.66-1.80)	2.8	0.0000	0.579
		Europe	6	1.73 (1.03-2.91)	79.0	0.2660	
		USA	1	1.87 (1.21-2.90)	NR	NR	
		Others	2	1.25 (0.94-1.66)	0.0	0.0000	
	ICIs used	Anti-PD-1/PD-L1	10	1.56 (1.07-2.27)	78.1	0.2141	0.551
		Anti-PD-1/PD-L1 + Anti-CTLA-4	2	1.25 (0.86-1.83)	0.0	0.0000	

### 3.10 Sensitivity analyses and assessment of small-study effects

Unadjusted OS estimates from 40 publications contributing 45 cohort estimates were directionally consistent with the adjusted analysis (HR = 0.72, 95% CI: 0.65-0.79, *P* < 0.001; *I*^2^ = 67.5%) (**Supplementary Figure S17**). For PFS, 36 publications contributed 40 estimates and showed the same pattern (HR = 0.77, 95% CI: 0.70-0.86, *P* < 0.001; *I*^2^ = 58.9%) (**Supplementary Figure S18**). Across 22 publications, unadjusted estimates also indicated higher ORR (OR = 1.32, 95% CI: 1.04-1.69, *P* < 0.05; *I*^2^ = 55.4%) (**Supplementary Figure S19**). Any-grade irAEs were more frequent at higher BMI across 12 publications (OR = 1.50, 95% CI: 1.10-2.05, *P* < 0.05; *I*^2^ = 76.1%) (**Supplementary Figure S20**). Grade 3 or higher irAEs remained inconclusive across six publications (OR = 1.20, 95% CI: 0.77-1.88, *P*>0.05; *I*^2^ = 23.5%) (**Supplementary Figure S21**). Leave-one-out findings were stable except for unadjusted ORR, which was sensitive to selected study omissions. Egger’s tests did not detect statistically significant funnel-plot asymmetry for adjusted OS, adjusted PFS, or unadjusted any-grade irAEs (all *P*>0.05). Statistically significant asymmetry was observed for unadjusted OS, PFS, and ORR (all *P* < 0.05), suggesting possible small-study effects (**Supplementary Figures S22**-**S53**).

## 4 Discussion

This meta-analysis of 61 studies and 46,572 patients found that higher pretreatment BMI was associated with longer adjusted OS and PFS during ICI therapy. More specifically, category-specific analyses provided the clearest evidence for obesity and the combined overweight/obesity category, whereas overweight alone and underweight comparisons remained inconclusive. In addition, cutoff meta-regression did not identify threshold value as a moderator, and only PFS showed a significant adjusted linear dose-response association. However, adjusted analyses did not establish associations with ORR or irAEs, despite positive unadjusted ORR and any-grade irAE estimates. Likewise, clinical subgroup tests also did not identify robust effect modification by cancer type, region, or ICI class. Taken together, these findings indicate favorable average associations of higher BMI with OS and PFS. However, the 95% prediction intervals for both primary models included the null value, indicating that the magnitude and potentially the direction of these associations may vary across clinical settings.

Broadly consistent with our findings, earlier evidence generally favored higher BMI, but the strength and scope of the association varied (13–15). Initial aggregate-data meta-analyses reported stronger favorable survival associations than observed here (14, 15). Similarly, an individual-patient-data synthesis found longer survival among overweight or obese patients without an additional benefit for obesity over overweight (13). In contrast, other reviews emphasized the interpretive limitations created by retrospective cohorts, mixed tumors, and inconsistent BMI categories (88). Against this background, our larger dataset helps reconcile these apparently divergent findings. The overall adjusted association remained favorable, but its magnitude was more conservative and overweight alone was inconclusive. Notably, this finding contrasts with that of Guo et al., who reported favorable OS for both overweight and obesity under World Health Organization categories (89). Moreover, tumor-specific reviews have produced heterogeneous estimates in lung cancer and renal cell carcinoma, consistent with the imprecision of several clinical subgroups in our analysis (90, 91). Importantly, similar BMI-survival patterns outside ICI therapy further limit treatment-specific interpretation (92–94). Thus, without a non-ICI comparator and a treatment-by-BMI interaction, BMI remains a prognostic correlate; the present evidence does not establish differential ICI efficacy (95, 96).

Beyond survival outcomes, evidence linking BMI to response and toxicity has been less consistent than the survival literature. Specifically, earlier syntheses reported either no association with all-grade irAEs or greater toxicity among overweight or obese patients (97). For example, in pooled CheckMate data, obesity was associated with more any-grade irAEs during nivolumab monotherapy, but not with grade 3 or 4 events overall (98). Consistent with this uncertainty, positive unadjusted ORR and any-grade irAE findings in our study were not confirmed by the smaller adjusted evidence sets, and unadjusted ORR was sensitive to study omission. Specifically, performance status, disease burden, treatment duration, sex, tumor type, and treatment line can influence both BMI and outcome ascertainment (98, 99). In addition, longer exposure creates more opportunity to document response and irAEs (100). These factors may partly explain attenuation after adjustment. Nevertheless, the association remains biologically plausible. Obesity can alter leptin signaling, PD-1 expression, T-cell function, macrophage polarization, adipokines, microbial pathways, and systemic metabolism (101, 102). However, BMI cannot distinguish adiposity from skeletal muscle depletion or recent catabolic weight loss (103, 104). Consistent with this limitation, sarcopenia, cachexia, and longitudinal body-composition changes provide prognostic information beyond baseline BMI. Taken together, the observed association may reflect both immunometabolic biology and residual confounding (105, 106).

Finally, the principal advance of this study lies in the triangulation of the BMI-outcome association across complementary analytic frameworks. We separated adjusted from unadjusted estimates, harmonized comparison direction, and prevented simultaneous inclusion of correlated contrasts. Study-defined comparisons were evaluated alongside named BMI categories, cutoff meta-regression, and dose-response models. Formal interaction tests, influence analyses, and outcome-specific small-study assessments reduced reliance on isolated subgroup significance. Consequently, this design helps explain how a favorable higher-versus-lower survival association can coexist with inconclusive overweight and linear OS results. However, several limitations should be acknowledged. First, this systematic review was not prospectively registered, and the absence of an *a priori* public protocol should be considered when interpreting the findings. Second, most included studies were retrospective, and covariate adjustment was neither uniform nor comprehensive. Third, aggregate data prevented consistent control for weight loss, skeletal muscle, performance status, smoking, corticosteroid use, disease burden, and treatment line. Fourth, cutoff meta-regression was ecological and may have lacked power, while dose-response estimation required assumptions about category midpoints and within-study covariance. Available data did not support a robust nonlinear analysis. In addition, baseline BMI ignores prediagnosis weight trajectories and changes during therapy. Finally, adjusted ORR and irAE evidence was sparse, and potential small-study effects were detected in selected unadjusted analyses. Thus, BMI may provide inexpensive prognostic context, but it should not guide ICI selection, dosing, or weight-gain recommendations. Accordingly, prospective studies should integrate standardized BMI categories, longitudinal weight, computed tomography-derived muscle and adipose compartments, inflammatory markers, and prespecified confounders. Ultimately, individual-participant-data analyses and comparative treatment cohorts with treatment-by-BMI interactions are needed to assess nonlinear, sex-specific, and ICI-specific effects.

## 5 Conclusion

Higher pretreatment BMI was associated with longer adjusted OS and PFS during ICI therapy, but the association varied by BMI contrast and did not consistently extend to response or toxicity. BMI should therefore be regarded as an accessible prognostic correlate, not as a treatment-selection or dosing biomarker. Prospective studies incorporating longitudinal weight, body composition, and appropriate treatment comparators are needed to determine whether BMI-related phenotypes are associated with differential outcomes across treatment strategies.

## Data Availability

The original contributions presented in the study are included in the article/**Supplementary Material**. Further inquiries can be directed to the corresponding author.
