# Supplementary material for: Before-treatment body mass index as a prognostic factor in cancer patients receiving immune checkpoint inhibitors: a systematic review and dose-response meta-analysis
Source: Front Immunol. 2026 Sep 24;17:1921470. doi: 10.3389/fimmu.2026.1921470 (PMC13646123; doi:10.3389/fimmu.2026.1921470)
Supplement: Supplementary file 1 [file SupplementaryFile1.docx]

Supplementary Material

**Supplementary Figure S1.**Meta-regression of BMI cutoff values for adjusted OS

**Supplementary Figure S2.**Meta-regression of BMI cutoff values for adjusted PFS

**Supplementary Figure S3.**Associations of BMI Categories with adjusted OS

**Supplementary Figure S4.**Associations of BMI Categories with adjusted PFS

**Supplementary Figure S5.**Subgroups of Cancer Types for adjusted OS

**Supplementary Figure S6.**Subgroups of ICIs Used for adjusted OS

**Supplementary Figure S7.**Subgroups of Study Region for adjusted OS

**Supplementary Figure S8.**Subgroups of Cancer Types for adjusted PFS

**Supplementary Figure S9.**Subgroups of ICIs Used for adjusted PFS

**Supplementary Figure S10.**Subgroups of Study Region for adjusted PFS

**Supplementary Figure S11.**Subgroups of Cancer Types for adjusted ORR

**Supplementary Figure S12.**Subgroups of ICIs Used for adjusted ORR

**Supplementary Figure S13.**Subgroups of Study Region for adjusted ORR

**Supplementary Figure S14.**Subgroups of Cancer Types for unadjusted any grade irAEs

**Supplementary Figure S15.**Subgroups of ICIs Used for unadjusted any grade irAEs

**Supplementary Figure S16.**Subgroups of Study Region for unadjusted any grade irAEs

**Supplementary Figure S17.**Associations of BMI and unadjusted OS estimates

**Supplementary Figure S18.**Associations of BMI and unadjusted PFS estimates

**Supplementary Figure S19.**Associations of BMI and unadjusted ORR estimates

**Supplementary Figure S20.**Associations of BMI and unadjusted any grade irAEs estimates

**Supplementary Figure S21.**Associations of BMI and unadjusted ≥3 irAEs estimates

**Supplementary Figure S22.**Sensitivity Analyse of adjusted OS estimates

**Supplementary Figure S23.**Sensitivity Analyse of unadjusted OS estimates

**Supplementary Figure S24.**Sensitivity Analyse of adjusted PFS estimates

**Supplementary Figure S25.**Sensitivity Analyse of unadjusted PFS estimates

**Supplementary Figure S26.**Sensitivity analysis of Overweight vs normal weight in OS

**Supplementary Figure S27.**Sensitivity analysis of Obese vs normal weight in OS

**Supplementary Figure S28.**Sensitivity analysis of Underweight vs normal weight in OS

**Supplementary Figure S29.**Sensitivity analysis of Overweight/obese vs normal/underweight in OS

**Supplementary Figure S30.**Sensitivity analysis of Overweight vs normal weight in PFS

**Supplementary Figure S31.**Sensitivity analysis of Obese vs normal weight in PFS

**Supplementary Figure S32.**Sensitivity analysis of Underweight vs normal weight in PFS

**Supplementary Figure S33.**Sensitivity analysis of Overweight/obese vs normal/underweight in PFS

**Supplementary Figure S34.**Sensitivity Analyse of adjusted ORR estimates

**Supplementary Figure S35.**Sensitivity Analyse of unadjusted ORR estimates

**Supplementary Figure S36.**Sensitivity Analyse of unadjusted any grade irAEs estimates

**Supplementary Figure S37.**Sensitivity Analyse of unadjusted ≥3 irAEs estimates

**Supplementary Figure S38.**Funnel plot of adjusted OS estimates

**Supplementary Figure S39.**Funnel plot of unadjusted OS estimates

**Supplementary Figure S40.**Funnel plot of adjusted PFS estimates

**Supplementary Figure S41.**Funnel plot of unadjusted PFS estimates

**Supplementary Figure S42.**Funnel plot of Overweight vs normal weight in OS

**Supplementary Figure S43.**Funnel plot of Obese vs normal weight in OS

**Supplementary Figure S44.**Funnel plot of Underweight vs normal weight in OS

**Supplementary Figure S45.**Funnel plot of Overweight/Obese vs Underweight/normal weight in OS

**Supplementary Figure S46.**Funnel plot of Overweight vs normal weight in PFS

**Supplementary Figure S47.**Funnel plot of Obese vs normal weight in PFS

**Supplementary Figure S48.**Funnel plot of Underweight vs normal weight in PFS

**Supplementary Figure S49.**Funnel plot of Overweight/Obese vs Underweight/normal weight in PFS

**Supplementary Figure S50.**Funnel plot of adjusted ORR estimates

**Supplementary Figure S51.**Funnel plot of unadjusted ORR estimates

**Supplementary Figure S52.**Funnel plot of unadjusted any grade irAEs estimates

**Supplementary Figure S53.**Funnel plot of unadjusted ≥3 irAEs estimates

**Supplementary Table S1.**Full search strategies for PubMed, Embase, the Cochrane Library, and Web of Science

| **Database** | **Searchterms** |
| --- | --- |
| PubMed | #1:("Tumors"[Title/Abstract] OR "Neoplasia"[Title/Abstract] OR "Neoplasias"[Title/Abstract] OR  "Neoplasm"[Title/Abstract] OR "Tumor"[Title/Abstract] OR "Cancer"[Title/Abstract] OR  "Cancers"[Title/Abstract] OR "malignant neoplasm"[Title/Abstract] OR  "Malignancy"[Title/Abstract] OR "Malignancies"[Title/Abstract] OR  "malignant neoplasms"[Title/Abstract] OR "neoplasm malignant"[Title/Abstract] OR  "neoplasms malignant"[Title/Abstract] OR "benign neoplasms"[Title/Abstract] OR  "neoplasms benign"[Title/Abstract] OR "neoplasm benign"[Title/Abstract] OR  "benign neoplasm"[Title/Abstract]) AND (1784:2025[pdat]) |
|  | #2:("nivolumab"[Title/Abstract] OR "Opdivo"[Title/Abstract] OR "ono 4538"[Title/Abstract] OR  "MDX-1106"[Title/Abstract] OR "BMS-936558"[Title/Abstract] OR "pembrolizumab"[Title/Abstract] OR  "SCH-900475"[Title/Abstract] OR "Keytruda"[Title/Abstract] OR "MK-3475"[Title/Abstract] OR  "atezolizumab"[Title/Abstract] OR "MPDL-3280A"[Title/Abstract] OR "rg 7446"[Title/Abstract] OR  "durvalumab"[Title/Abstract] OR "MEDI-4736"[Title/Abstract] OR "Imfinzi"[Title/Abstract] OR  "avelumab"[Title/Abstract] OR ("msb"[All Fields] AND "0010682"[Title/Abstract]) OR  "MSB-0010718C"[Title/Abstract] OR "PD-1"[Title/Abstract] OR "SHR-1210"[Title/Abstract] OR  "PD-1"[Title/Abstract] OR "PD-L1"[Title/Abstract] OR "anti-PD-1"[Title/Abstract] OR  "anti-PD-L1"[Title/Abstract] OR "immune checkpoint inhibitor"[Title/Abstract] OR  "immune checkpoint inhibitors"[Title/Abstract] OR "ICI"[Title/Abstract] OR "ICIs"[Title/Abstract] OR  "immune checkpoint blocker"[Title/Abstract] OR "immune checkpoint blockers"[Title/Abstract] OR  "ICB"[Title/Abstract] OR "ICBs"[Title/Abstract] OR "immunotherapy"[Title/Abstract] OR  "CTLA-4"[Title/Abstract] OR "Anti-CTLA-4"[Title/Abstract] OR "Ipilimumab"[Title/Abstract] OR  "mdx 010"[Title/Abstract] OR "Yervoy"[Title/Abstract] OR "Tremelimumab"[Title/Abstract] OR  "Sintilimab"[Title/Abstract] OR "sintilimab"[Title/Abstract] OR "CP-675"[Title/Abstract]) AND (1945:2025[pdat]) |
|  | #3:"body mass index"[Title/Abstract] OR "index body mass"[Title/Abstract] OR  "overweight"[Title/Abstract] OR "obesity"[Title/Abstract] |
|  | #4:#1 AND #2 AND #3 |
| Embase | #1:('malignant neoplasm' OR 'neoplasms' OR 'neoplasia' OR 'neoplasias' OR 'neoplasm' OR 'tumors' OR 'tumor' OR  'cancer' OR 'cancers' OR 'malignancy' OR 'malignant neoplasms' OR 'neoplasm, malignant' OR 'neoplasms, malignant' OR  'benign neoplasms' OR 'neoplasms, benign' OR 'benign neoplasm' OR 'neoplasm, benign') |
|  | #2:('nivolumab' OR 'pembrolizumab' OR 'atezolizumab' OR 'durvalumab' OR 'avelumab' OR 'pd-1' OR 'pd-l1' OR  'anti-pd-1' OR 'anti-pd-l1' OR 'immune checkpoint inhibitor' OR 'immunecheckpoint inhibitors' OR 'ici' OR 'icis' OR  'immune checkpointblocker' OR 'immune checkpoint blockers' OR 'icb' OR 'icbs' OR 'immunotherapy' OR 'ctla-4' OR  'anti-ctla-4' OR 'ipilimumab' OR 'tremelimumab'OR 'sintilimab'OR'Sintilimab') |
|  | #3:('body mass' OR 'body mass index' OR 'index, body mass' OR 'quetelet index' OR 'index, quetelet' OR 'quetelets index' OR  'overweight' OR 'obesity') |
|  | #4:#1 AND #2 AND #3 |
| Cochrane | #1:(nivolumab) OR (Opdivo) OR (ONO-4538) OR (MDX-1106) OR (BMS-936558) OR (pembrolizumab) OR  (SCH-900475) OR (Keytruda) OR (MK-3475) OR (atezolizumab) OR (MPDL-3280A) OR (RG-7446) OR (durvalumab) OR  (MEDI-4736) OR (Imfinzi) OR (avelumab) OR (MSB-0010682) OR (MSB-0010718C) OR (PD-1) OR (SHR-1210) OR  (PD-1) OR (PD-L1) OR (anti-PD-1) OR (anti-PD-L1) OR (immune checkpointinhibitor) OR (immune checkpoint inhibitors) OR  (ICI) OR (ICIs) OR (immune checkpoint blocker) OR (immune checkpointblockers) OR (ICB) OR (ICBs) OR  (immunotherapy) OR (CTLA-4) OR (Anti-CTLA-4) OR (Ipilimumab) OR (MDX 010) OR (Yervoy) OR (Tremelimumab) OR  (CP-675)OR(sintilimab)OR(Sintilimab) |
|  | #2:(malignant neoplasm) OR (neoplasms) OR (neoplasia) OR (neoplasias) OR (neoplasm) OR (tumors) OR (tumor) OR  (cancer) OR (cancers) OR (malignancy) OR (malignant neoplasms) OR (neoplasm, malignant) OR (neoplasms, malignant) OR  (benign neoplasms) OR (neoplasms, benign) OR (benign neoplasm) OR (neoplasm, benign) |
|  | #3:(body mass) OR (body mass index) OR (index, body mass) OR (quetelet index) OR (index, quetelet) OR  (quetelets index) OR (overweight) OR (obesity) |
|  | #4:#1 AND #2 AND #3 |
| Web of Science | #1：TS=(tumor OR tumour OR neoplas OR cancer OR malignan) |
|  | #2：TS=(nivolumab OR Opdivo OR "ONO-4538" OR "ONO 4538" OR "MDX-1106" OR "BMS-936558" OR pembrolizumab OR "SCH-900475" OR Keytruda OR "MK-3475" OR atezolizumab OR "MPDL-3280A" OR "RG-7446" OR "RG 7446" OR durvalumab OR "MEDI-4736" OR Imfinzi OR avelumab OR "MSB-0010682" OR "MSB-0010718C" OR "PD-1" OR "PD-L1" OR "anti-PD-1" OR "anti-PD-L1" OR "programmed cell death protein 1" OR "programmed cell death 1" OR "programmed death-ligand 1" OR "programmed death ligand 1" OR "immune checkpoint inhibitor" OR "immune checkpoint blockade" OR "immune checkpoint blocker" OR ICI OR ICIs OR ICB OR ICBs OR immunotherap OR "CTLA-4" OR "anti-CTLA-4" OR ipilimumab OR "MDX-010" OR "MDX 010" OR Yervoy OR tremelimumab OR "CP-675" OR sintilimab OR "SHR-1210") |
|  | #3：TS=("body mass index" OR BMI OR "index body mass" OR "Quetelet index" OR "index Quetelet" OR "Quetelet's index" OR overweight OR obesity OR underweight) |
|  | #4:#1 AND #2 AND #3 |

**Supplementary Table S2.**Item-level Newcastle-Ottawa Scale assessment of the included studies

| **Study** | **S1** | **S2** | **S3** | **S4** | **C1** | **C2** | **O1** | **O2** | **O3** | **Calculated total** | **Original total** | **Quality** |
| --- | --- | --- | --- | --- | --- | --- | --- | --- | --- | --- | --- | --- |
| Heidelberger 2017 | 1 | 1 | 1 | 1 | 0 | 0 | 1 | 1 | 1 | 7 | 7 | Moderate |
| Kondo 2018 | 1 | 1 | 1 | 1 | 1 | 0 | 1 | 1 | 1 | 8 | 8 | High |
| Mcquade 2018 | 0 | 1 | 1 | 1 | 1 | 1 | 1 | 1 | 0 | 7 | 7 | Moderate |
| Dumenil 2018 | 1 | 1 | 1 | 1 | 1 | 0 | 1 | 1 | 1 | 8 | 8 | High |
| Shiroyama 2018 | 1 | 1 | 1 | 1 | 0 | 0 | 1 | 1 | 1 | 7 | 7 | Moderate |
| Qi 2019 | 0 | 1 | 1 | 1 | 1 | 1 | 1 | 1 | 0 | 7 | 7 | Moderate |
| Martini 2019 | 0 | 1 | 1 | 1 | 1 | 1 | 1 | 1 | 0 | 7 | 7 | Moderate |
| Cortellini 2019 | 1 | 1 | 1 | 1 | 1 | 1 | 1 | 1 | 0 | 8 | 8 | High |
| Giorgi 2019 | 1 | 1 | 1 | 1 | 1 | 1 | 1 | 1 | 0 | 8 | 8 | High |
| Donnelly 2019 | 0 | 1 | 1 | 1 | 1 | 1 | 1 | 1 | 0 | 7 | 7 | Moderate |
| Katayama 2019 | 1 | 1 | 1 | 1 | 1 | 0 | 1 | 1 | 1 | 8 | 8 | High |
| Kichenadasse 2019 | 1 | 1 | 1 | 1 | 1 | 1 | 1 | 1 | 0 | 8 | 8 | High |
| Labadie 2019 | 1 | 1 | 1 | 1 | 1 | 0 | 1 | 1 | 1 | 8 | 8 | High |
| Magri 2019 | 0 | 1 | 1 | 1 | 1 | 1 | 1 | 1 | 1 | 8 | 8 | High |
| Popinat 2019 | 1 | 1 | 1 | 1 | 0 | 0 | 1 | 1 | 1 | 7 | 7 | Moderate |
| Wang 2019 | 1 | 1 | 1 | 1 | 1 | 1 | 1 | 1 | 0 | 8 | 8 | High |
| Naik 2019 | 1 | 1 | 1 | 1 | 1 | 1 | 1 | 1 | 0 | 8 | 8 | High |
| Cortellini 2020 | 1 | 1 | 1 | 1 | 1 | 1 | 1 | 1 | 0 | 8 | 8 | High |
| Nitipir 2020 | 1 | 1 | 1 | 1 | 0 | 0 | 1 | 1 | 1 | 7 | 7 | Moderate |
| Rutkowski 2020 | 1 | 1 | 1 | 1 | 1 | 1 | 1 | 1 | 0 | 8 | 8 | High |
| Ichihara 2020 | 1 | 1 | 1 | 1 | 0 | 0 | 1 | 1 | 0 | 6 | 6 | Moderate |
| Rogado 2020 | 1 | 1 | 1 | 1 | 1 | 1 | 1 | 1 | 0 | 8 | 8 | High |
| Minami 2020 | 0 | 1 | 1 | 1 | 1 | 1 | 1 | 1 | 0 | 7 | 7 | Moderate |
| Ahmed 2021 | 0 | 1 | 1 | 1 | 1 | 1 | 1 | 1 | 0 | 7 | 7 | Moderate |
| Bartl 2021 | 0 | 1 | 1 | 1 | 1 | 1 | 1 | 1 | 1 | 8 | 8 | High |
| Collet 2021 | 0 | 1 | 1 | 1 | 1 | 1 | 1 | 1 | 0 | 7 | 7 | Moderate |
| Imai 2021 | 1 | 1 | 1 | 1 | 1 | 1 | 1 | 1 | 0 | 8 | 8 | High |
| Akce 2021 | 0 | 1 | 1 | 1 | 1 | 1 | 1 | 1 | 0 | 7 | 7 | Moderate |
| Esposito 2021 | 0 | 1 | 1 | 1 | 1 | 1 | 1 | 1 | 0 | 7 | 7 | Moderate |
| Zhang 2021 | 1 | 1 | 1 | 1 | 1 | 1 | 1 | 1 | 0 | 8 | 8 | High |
| Chen 2022 | 1 | 1 | 1 | 1 | 1 | 1 | 1 | 1 | 0 | 8 | 8 | High |
| Ged 2022 | 1 | 1 | 1 | 1 | 1 | 0 | 1 | 1 | 0 | 7 | 7 | Moderate |
| Imai 2022 | 0 | 1 | 1 | 1 | 1 | 0 | 1 | 1 | 1 | 7 | 7 | Moderate |
| Joshi 2022 | 0 | 1 | 1 | 1 | 1 | 1 | 1 | 1 | 0 | 7 | 7 | Moderate |
| Wang 2022 | 0 | 1 | 1 | 1 | 1 | 1 | 1 | 1 | 0 | 7 | 7 | Moderate |
| Tateishi 2022 | 1 | 1 | 1 | 1 | 1 | 1 | 1 | 1 | 0 | 8 | 8 | High |
| Wasamoto 2023 | 1 | 1 | 1 | 1 | 1 | 0 | 1 | 1 | 1 | 8 | 8 | High |
| Incorvaia 2023 | 1 | 1 | 1 | 1 | 1 | 1 | 1 | 1 | 0 | 8 | 8 | High |
| Jain 2023 | 1 | 1 | 1 | 1 | 1 | 1 | 1 | 1 | 0 | 8 | 8 | High |
| Vithayathil 2023 | 1 | 1 | 1 | 1 | 1 | 1 | 1 | 1 | 0 | 8 | 8 | High |
| Wu 2023 | 1 | 1 | 1 | 1 | 0 | 0 | 1 | 1 | 1 | 7 | 7 | Moderate |
| Zhang 2023 | 0 | 1 | 1 | 1 | 1 | 1 | 1 | 1 | 1 | 8 | 8 | High |
| McManus 2023 | 0 | 1 | 1 | 1 | 1 | 1 | 1 | 1 | 1 | 8 | 8 | High |
| Miyamoto 2023 | 1 | 1 | 1 | 1 | 1 | 1 | 1 | 1 | 0 | 8 | 8 | High |
| Antoun 2023 | 0 | 1 | 1 | 1 | 1 | 0 | 1 | 1 | 0 | 6 | 6 | Moderate |
| Santoni 2023 | 0 | 1 | 1 | 1 | 1 | 1 | 1 | 1 | 0 | 7 | 7 | Moderate |
| Alam 2024 | 1 | 1 | 1 | 1 | 1 | 1 | 1 | 1 | 0 | 8 | 8 | High |
| Bernard 2024 | 0 | 1 | 1 | 1 | 1 | 1 | 1 | 1 | 1 | 8 | 8 | High |
| Gómez-Banoy 2024 | 1 | 1 | 1 | 1 | 1 | 1 | 1 | 1 | 0 | 8 | 8 | High |
| Nie 2024 | 1 | 1 | 1 | 1 | 1 | 1 | 1 | 1 | 0 | 8 | 8 | High |
| Tan 2024 | 0 | 1 | 1 | 1 | 1 | 1 | 1 | 1 | 0 | 7 | 7 | Moderate |
| Wang 2024 | 1 | 1 | 1 | 1 | 1 | 1 | 1 | 1 | 0 | 8 | 8 | High |
| Llerena 2024 | 0 | 1 | 1 | 1 | 1 | 1 | 1 | 1 | 0 | 7 | 7 | Moderate |
| Ihara 2024 | 1 | 1 | 1 | 1 | 0 | 0 | 1 | 1 | 0 | 6 | 6 | Moderate |
| Şen 2024 | 1 | 1 | 1 | 1 | 1 | 0 | 1 | 1 | 1 | 8 | 8 | High |
| Yamamoto 2024 | 1 | 1 | 1 | 1 | 1 | 1 | 1 | 1 | 0 | 8 | 8 | High |
| Demir 2025 | 1 | 1 | 1 | 1 | 1 | 0 | 1 | 1 | 1 | 8 | 8 | High |
| Mastrolonardo 2025 | 0 | 1 | 1 | 1 | 1 | 1 | 1 | 1 | 0 | 7 | 7 | Moderate |
| Çolak 2025 | 1 | 1 | 1 | 1 | 0 | 0 | 1 | 1 | 1 | 7 | 7 | Moderate |
| Krejčí 2025 | 1 | 1 | 1 | 1 | 1 | 0 | 1 | 1 | 1 | 8 | 8 | High |
| Anami 2025 | 1 | 1 | 1 | 1 | 1 | 0 | 1 | 1 | 1 | 8 | 8 | High |

**Supplementary Table S3.**Study- and outcome-specific covariates and sources of effect estimates

| **Study** | **Cohort label** | **Cohort/source** | **Outcome** | **Effect measure** | **Adjustment** | **Effect** | **Lower 95% CI** | **Upper 95% CI** | **Adjusted covariates** | **Effect source** | **Analysis N** | **Meta-analysis use** |
| --- | --- | --- | --- | --- | --- | --- | --- | --- | --- | --- | --- | --- |
| Mcquade 2018 | Mcquade (1) | Ipilimumab + DTIC cohort | OS | HR | Adjusted | 0.540 | 0.340 | 0.860 | Sex, age, stage, LDH, ECOG PS | Multivariable Cox regression | 207 | Primary adjusted analysis |
| Mcquade 2018 | Mcquade (1) | Ipilimumab + DTIC cohort | OS | HR | Unadjusted | 0.640 | 0.420 | 0.970 | None (unadjusted estimate) | Reported crude/univariable HR | 207 | Separate unadjusted sensitivity analysis |
| Mcquade 2018 | Mcquade (2) | Anti-PD-1/PD-L1 cohort | OS | HR | Adjusted | 0.720 | 0.480 | 1.060 | Sex, age, stage, LDH, ECOG PS | Multivariable Cox regression | 329 | Primary adjusted analysis |
| Mcquade 2018 | Mcquade (2) | Anti-PD-1/PD-L1 cohort | OS | HR | Unadjusted | 0.700 | 0.480 | 1.010 | None (unadjusted estimate) | Reported crude/univariable HR | 329 | Separate unadjusted sensitivity analysis |
| Dumenil 2018 | Dumenil | Overall cohort | OS | HR | Unadjusted | 0.775 | 0.328 | 1.818 | None (unadjusted estimate) | Reported crude/univariable HR | 67 | Separate unadjusted sensitivity analysis |
| Qi 2019 | Qi | Overall cohort | OS | HR | Adjusted | 0.496 | 0.260 | 0.945 | ECOG PS;  PLR | Multivariable Cox regression | 85 | Primary adjusted analysis |
| Qi 2019 | Qi | Overall cohort | OS | HR | Unadjusted | 0.496 | 0.261 | 0.943 | None (unadjusted estimate) | Reported crude/univariable HR | 85 | Separate unadjusted sensitivity analysis |
| Martini 2019 | Martini | Overall cohort | OS | HR | Adjusted | 0.306 | 0.135 | 0.694 | Histology;  number of distant metastases;  number of prior lines of therapy;  prior checkpoint inhibitor;  RMH risk group;  age;  checkpoint inhibitor-indicated histology;  ECOG PS;  sex;  race | Multivariable Cox regression | 81 | Primary adjusted analysis |
| Cortellini 2019 | Cortellini | Overall cohort | OS | HR | Adjusted | 0.500 | 0.320 | 0.790 | Weight(continuous), irAEs of any grade, primary tumor, sex, treatment line, ECOG-PS, number of metastatic sites | Multivariable Cox regression | 936 | Primary adjusted analysis |
| Cortellini 2019 | Cortellini | Overall cohort | OS | HR | Unadjusted | 0.370 | 0.270 | 0.510 | None (unadjusted estimate) | Reported crude/univariable HR | 936 | Separate unadjusted sensitivity analysis |
| Giorgi 2019 | Giorgi | Overall cohort | OS | HR | Adjusted | 0.633 | 0.439 | 0.917 | Age;  SII | Multivariable Cox regression | 293 | Primary adjusted analysis |
| Giorgi 2019 | Giorgi | Overall cohort | OS | HR | Unadjusted | 0.667 | 0.465 | 0.952 | None (unadjusted estimate) | Reported crude/univariable HR | 293 | Separate unadjusted sensitivity analysis |
| Donnelly 2019 | Donnelly (1) | Anti-CTLA-4 cohort | OS | HR | Adjusted | 1.220 | 0.750 | 2.000 | Age;  gender;  stage;  LDH;  ECOG PS;  number of metastatic sites;  BRAF mutation status | Multivariable Cox regression | 200 | Primary adjusted analysis |
| Donnelly 2019 | Donnelly (1) | Anti-CTLA-4 cohort | OS | HR | Unadjusted | 1.070 | 0.630 | 1.800 | None (unadjusted estimate) | Reported crude/univariable HR | 200 | Separate unadjusted sensitivity analysis |
| Donnelly 2019 | Donnelly (2) | Anti-PD-1 cohort | OS | HR | Adjusted | 1.040 | 0.480 | 2.240 | Age;  gender;  stage;  LDH;  ECOG PS;  number of metastatic sites;  BRAF mutation status | Multivariable Cox regression | 145 | Primary adjusted analysis |
| Donnelly 2019 | Donnelly (2) | Anti-PD-1 cohort | OS | HR | Unadjusted | 0.880 | 0.420 | 1.850 | None (unadjusted estimate) | Reported crude/univariable HR | 145 | Separate unadjusted sensitivity analysis |
| Katayama 2019 | Katayama | Overall cohort | OS | HR | Unadjusted | 0.920 | 0.260 | 3.250 | None (unadjusted estimate) | Reported crude/univariable HR | 35(3 vs 32) | Separate unadjusted sensitivity analysis |
| Kichenadasse 2019 | Kichenadasse | Overall cohort | OS | HR | Adjusted | 0.690 | 0.540 | 0.870 | Age, sex, race, ECOG PS, histology, number of tumor sites, number of prior treatments, smoking history, PD-L1 expression, LDH, log-transformed CRP, and NLR; model stratified by study | Multivariable Cox regression | 944 | Primary adjusted analysis |
| Kichenadasse 2019 | Kichenadasse | Overall cohort | OS | HR | Unadjusted | 0.640 | 0.510 | 0.810 | None (unadjusted estimate) | Reported crude/univariable HR | 944 | Separate unadjusted sensitivity analysis |
| Labadie 2019 | Labadie | Overall cohort | OS | HR | Unadjusted | 0.280 | 0.090 | 0.910 | None (unadjusted estimate) | Reported crude/univariable HR | 90 | Separate unadjusted sensitivity analysis |
| Magri 2019 | Magri | Overall cohort | OS | HR | Adjusted | 1.190 | 0.930 | 1.510 | Sex, age, PS, histology, smoking, previous treatment lines, albumin, SMI, FFMI, FMI, weight change | Multivariable Cox regression | NR | Primary adjusted analysis |
| Magri 2019 | Magri | Overall cohort | OS | HR | Unadjusted | 1.010 | 0.940 | 1.090 | None (unadjusted estimate) | Reported crude/univariable HR | NR | Separate unadjusted sensitivity analysis |
| Popinat 2019 | Popinat | Overall cohort | OS | HR | NR |  |  |  | NR | No extractable HR/OR reported in data.xlsx | 55 | Not meta-analysed |
| Wang 2019 | Wang | Overall cohort | OS | HR | Adjusted | 0.594 | 0.350 | 0.990 | ECOG PS;  line of treatment;  age;  sex;  cancer type | Multivariable Cox regression | 250 | Primary adjusted analysis |
| Naik 2019 | Naik | Overall cohort | OS | HR | Adjusted | 0.420 | 0.100 | 1.770 | Age; gender; serum creatinine; treatment; current drinking; smoking; KPS; LDH; stage; Charlson score; hemoglobin; ANC; ALC; albumin; autoimmune disease; diabetes; cardiovascular disease; CKD; BRAF; NRAS; hypertension; hyperlipidemia; comedications; prior treatments; fasting glucose; melanoma type | Multivariable Cox regression | 127/un136 | Primary adjusted analysis |
| Naik 2019 | Naik | Overall cohort | OS | HR | Unadjusted | 0.880 | 0.410 | 1.910 | None (unadjusted estimate) | Reported crude/univariable HR | 127/un136 | Separate unadjusted sensitivity analysis |
| Cortellini 2020 | Cortellini | Overall cohort | OS | HR | Adjusted | 0.700 | 0.490 | 0.990 | Age;  gender;  ECOG-PS;  CNS metastases;  bone metastases;  liver metastases | Multivariable Cox regression | 962 | Primary adjusted analysis |
| Rutkowski 2020 | Rutkowski | Overall cohort | OS | HR | Adjusted | 1.170 | 0.820 | 1.680 | Age; sex; BRAF mutation; AJCC stage; ECOG PS; LDH; type of immunotherapy; model stratified by study center | Multivariable Cox regression | 393 | Primary adjusted analysis |
| Ichihara 2020 | Ichihara (1) | Pembrolizumab cohort (N=84) | OS | HR | Unadjusted | 0.670 | 0.320 | 1.400 | None (unadjusted estimate) | Reported crude/univariable HR | 84 | Separate unadjusted sensitivity analysis |
| Ichihara 2020 | Ichihara (2) | Mixed anti-PD-1/PD-L1 cohort (N=429) | OS | HR | Unadjusted | 0.280 | 0.085 | 0.890 | None (unadjusted estimate) | Reported crude/univariable HR | 429 | Separate unadjusted sensitivity analysis |
| Minami 2020 | Minami | Overall cohort | OS | HR | Adjusted | 0.550 | 0.240 | 1.270 | Albumin, NLR, ECOG-PS, EGFR/ALK mutation | Multivariable Cox regression | 74 | Primary adjusted analysis |
| Ahmed 2021 | Ahmed | Overall cohort | OS | HR | Adjusted | 0.960 | 0.650 | 1.420 | Age, sex, race, cancer type, anti-CTLA4, concurrent/sequential therapy, dosing strategy, BMI×dosing interaction | Multivariable Cox regression | 297 | Primary adjusted analysis |
| Ahmed 2021 | Ahmed | Overall cohort | OS | HR | Unadjusted | 0.770 | 0.570 | 1.040 | None (unadjusted estimate) | Reported crude/univariable HR | 297 | Separate unadjusted sensitivity analysis |
| Bartl 2021 | Bartl | Overall cohort | OS | HR | Adjusted | 0.130 | 0.030 | 0.620 | NLR, CCI, subcutaneous fat volume | Multivariable Cox regression | 36 | Primary adjusted analysis |
| Bartl 2021 | Bartl | Overall cohort | OS | HR | Unadjusted | 0.530 | 0.300 | 0.930 | None (unadjusted estimate) | Reported crude/univariable HR | 36 | Separate unadjusted sensitivity analysis |
| Collet 2021 | Collet | Overall cohort | OS | HR | Adjusted | 0.750 | 0.510 | 1.100 | irAEs, ECOG PS, and MSI status | Multivariable Cox regression | 272 | Primary adjusted analysis |
| Collet 2021 | Collet | Overall cohort | OS | HR | Unadjusted | 0.630 | 0.440 | 0.920 | None (unadjusted estimate) | Reported crude/univariable HR | 272 | Separate unadjusted sensitivity analysis |
| Imai 2021 | Imai | Overall cohort | OS | HR | Adjusted | 0.500 | 0.300 | 0.830 | ECOG PS, GPS, NLR | Multivariable Cox regression | 142 | Primary adjusted analysis |
| Imai 2021 | Imai | Overall cohort | OS | HR | Unadjusted | 0.510 | 0.300 | 0.830 | None (unadjusted estimate) | Reported crude/univariable HR | 142 | Separate unadjusted sensitivity analysis |
| Akce 2021 | Akce | Overall cohort | OS | HR | Adjusted | 0.440 | 0.180 | 1.090 | Baseline Child–Pugh score and NLR | Multivariable Cox regression | 57 | Primary adjusted analysis |
| Esposito 2021 | Esposito | Overall cohort | OS | HR | Adjusted | 0.870 | 0.550 | 1.360 | Age, ECOG PS, sex/breast cancer, albumin, number of metastatic sites, NLR, number of prior treatment lines, and prior immunotherapy | Multivariable Cox regression | 153 | Primary adjusted analysis |
| Esposito 2021 | Esposito | Overall cohort | OS | HR | Unadjusted | 0.800 | 0.530 | 1.210 | None (unadjusted estimate) | Reported crude/univariable HR | 153 | Separate unadjusted sensitivity analysis |
| Zhang 2021 | Zhang | Overall cohort | OS | HR | Adjusted | 0.620 | 0.440 | 0.870 | Age, sex, ECOG PS, smoking status, and time-dependent irAE | Multivariable Cox regression | 200 | Primary adjusted analysis |
| Ged 2022 | Ged | Overall cohort | OS | HR | Adjusted | 0.750 | 0.500 | 1.120 | IMDC risk score, line of ICB treatment | Multivariable Cox regression | 205 | Primary adjusted analysis |
| Ged 2022 | Ged | Overall cohort | OS | HR | Unadjusted | 0.660 | 0.450 | 0.970 | None (unadjusted estimate) | Reported crude/univariable HR | 205 | Separate unadjusted sensitivity analysis |
| Imai 2022 | Imai | Overall cohort | OS | HR | Adjusted | 0.560 | 0.340 | 0.910 | ECOG performance status | Multivariable Cox regression | 99 | Primary adjusted analysis |
| Imai 2022 | Imai | Overall cohort | OS | HR | Unadjusted | 0.490 | 0.300 | 0.780 | None (unadjusted estimate) | Reported crude/univariable HR | 99 | Separate unadjusted sensitivity analysis |
| Joshi 2022 | Joshi | Overall cohort | OS | HR | Adjusted | 0.984 | 0.914 | 1.061 | Age, sex, PD-L1, TMB, ECOG PS, therapy type | Multivariable Cox regression | 135 | Primary adjusted analysis |
| Joshi 2022 | Joshi | Overall cohort | OS | HR | Unadjusted | 0.952 | 0.911 | 0.996 | None (unadjusted estimate) | Reported crude/univariable HR | 135 | Separate unadjusted sensitivity analysis |
| Wang 2022 | Wang | Overall cohort | OS | HR | Adjusted | 0.560 | 0.070 | 4.260 | Age, sex, IMDC risk category, prior nephrectomy, visceral metastasis, bone metastasis | Multivariable Cox regression | 201 | Primary adjusted analysis |
| Wang 2022 | Wang | Overall cohort | OS | HR | Unadjusted | 1.090 | 0.380 | 3.130 | None (unadjusted estimate) | Reported crude/univariable HR | 201 | Separate unadjusted sensitivity analysis |
| Tateishi 2022 | Tateishi | Overall cohort | OS | HR | Unadjusted | 1.030 | 0.680 | 1.540 | None (unadjusted estimate) | Reported crude/univariable HR | 324 | Separate unadjusted sensitivity analysis |
| Wasamoto 2023 | Wasamoto | Overall cohort | OS | HR | Unadjusted | 0.660 | 0.380 | 1.140 | None (unadjusted estimate) | Reported crude/univariable HR | 84 | Separate unadjusted sensitivity analysis |
| Incorvaia 2023 | Incorvaia | Overall cohort | OS | HR | Unadjusted | 0.610 | 0.110 | 3.370 | None (unadjusted estimate) | Reported crude/univariable HR | 32 | Separate unadjusted sensitivity analysis |
| Jain 2023 | Jain | Overall cohort | OS | HR | Adjusted | 0.830 | 0.490 | 1.420 | Age, sex, ECOG PS, histology, and number of prior treatment lines | Multivariable Cox regression | 200 | Primary adjusted analysis |
| Vithayathil 2023 | Vithayathil | Overall cohort | OS | HR | Adjusted | 0.830 | 0.420 | 1.640 | BCLC stage, Child–Turcotte–Pugh class, tumor diameter, macrovascular invasion, metastatic disease, and AFP | Multivariable Cox regression | NR | Primary adjusted analysis |
| Vithayathil 2023 | Vithayathil | Overall cohort | OS | HR | Unadjusted | 1.290 | 0.700 | 2.390 | None (unadjusted estimate) | Reported crude/univariable HR | NR | Separate unadjusted sensitivity analysis |
| Wu 2023 | Wu | Overall cohort | OS | HR | Unadjusted | 0.620 | 0.320 | 1.200 | None (unadjusted estimate) | Reported crude/univariable HR | 78 | Separate unadjusted sensitivity analysis |
| Zhang 2023 | Zhang | Overall cohort | OS | HR | Adjusted | 0.947 | 0.089 | 10.047 | Sex, prior treatment, serum albumin, ECOG PS, CPS, and T stage of the recurrent tumor | Multivariable Cox regression | 49 | Primary adjusted analysis |
| Zhang 2023 | Zhang | Overall cohort | OS | HR | Unadjusted | 0.438 | 0.056 | 3.401 | None (unadjusted estimate) | Reported crude/univariable HR | 49 | Separate unadjusted sensitivity analysis |
| McManus 2023 | McManus | Overall cohort | OS | HR | Adjusted | 0.984 | 0.498 | 1.941 | Age, sex, and IMDC risk score | Multivariable Cox regression | 99 | Primary adjusted analysis |
| McManus 2023 | McManus | Overall cohort | OS | HR | Unadjusted | 0.916 | 0.489 | 1.718 | None (unadjusted estimate) | Reported crude/univariable HR | 99 | Separate unadjusted sensitivity analysis |
| Miyamoto 2023 | Miyamoto | Overall cohort | OS | HR | Adjusted | 1.080 | 0.630 | 1.820 | Age, ECOG PS, treatment line, and HPV status | Multivariable Cox regression | 106 | Primary adjusted analysis |
| Miyamoto 2023 | Miyamoto | Overall cohort | OS | HR | Unadjusted | 1.010 | 0.600 | 1.690 | None (unadjusted estimate) | Reported crude/univariable HR | 106 | Separate unadjusted sensitivity analysis |
| Antoun 2023 | Antoun (1) | Melanoma cohort | OS | HR | Adjusted | 0.850 | 0.470 | 1.560 | Sex, pretreatment weight loss, and skeletal muscle index | Multivariable Cox regression | NR | Primary adjusted analysis |
| Antoun 2023 | Antoun (1) | Melanoma cohort | OS | HR | Unadjusted | 0.770 | 0.520 | 1.160 | None (unadjusted estimate) | Reported crude/univariable HR | NR | Separate unadjusted sensitivity analysis |
| Antoun 2023 | Antoun (2) | NSCLC cohort | OS | HR | Adjusted | 1.540 | 0.730 | 3.280 | Sex, pretreatment weight loss, and skeletal muscle index | Multivariable Cox regression | NR | Primary adjusted analysis |
| Antoun 2023 | Antoun (2) | NSCLC cohort | OS | HR | Unadjusted | 0.690 | 0.430 | 1.110 | None (unadjusted estimate) | Reported crude/univariable HR | NR | Separate unadjusted sensitivity analysis |
| Santoni 2023 | Santoni | Overall cohort | OS | HR | Adjusted | 0.680 | 0.480 | 0.950 | IMDC prognostic group, prior nephrectomy, histology, bone metastasis, liver metastasis, brain metastasis, and NLR | Multivariable Cox regression | 675 | Primary adjusted analysis |
| Santoni 2023 | Santoni | Overall cohort | OS | HR | Unadjusted | 0.520 | 0.380 | 0.730 | None (unadjusted estimate) | Reported crude/univariable HR | 675 | Separate unadjusted sensitivity analysis |
| Alam 2024 | Alam | Overall cohort | OS | HR | Adjusted | 0.940 | 0.570 | 1.560 | Age, sex, disease stage, immunosuppression status, and ECOG PS | Multivariable Cox regression | 169 | Primary adjusted analysis |
| Bernard 2024 | Bernard | Overall cohort | OS | HR | Unadjusted | 0.710 | 0.260 | 1.980 | None (unadjusted estimate) | Reported crude/univariable HR | 44 | Separate unadjusted sensitivity analysis |
| Gómez-Banoy 2024 | Gómez-Banoy | Overall cohort | OS | HR | Adjusted | 0.640 | 0.480 | 0.870 | Age;  self-reported race;  histology;  checkpoint inhibitor treatment;  combination therapies;  baseline ECOG performance status;  stage at diagnosis;  previous lines of therapy;  previous pelvic radiotherapy | Multivariable Cox regression | 233 vs 128; total cohort N = 524 | Primary adjusted analysis |
| Gómez-Banoy 2024 | Gómez-Banoy | Overall cohort | OS | HR | Unadjusted | 0.650 | 0.490 | 0.860 | None (unadjusted estimate) | Reported crude/univariable HR | 233 vs 128; total cohort N = 524 | Separate unadjusted sensitivity analysis |
| Nie 2024 | Nie (1) | CGDB-Immu | OS | HR | Adjusted | 0.910 | 0.760 | 1.090 | Age, sex, race, ECOG PS, tumor type, smoking status, stage, metastatic sites, gene mutations, tissue TMB, and PD-L1 | Multivariable Cox regression | 170/298; 366/618 | Primary adjusted analysis |
| Nie 2024 | Nie (1) | CGDB-Immu | OS | HR | Unadjusted | 0.910 | 0.760 | 1.090 | None (unadjusted estimate) | Reported crude/univariable HR | 170/298; 366/618 | Separate unadjusted sensitivity analysis |
| Nie 2024 | Nie (2) | Chowell-Immu | OS | HR | Adjusted | 0.980 | 0.510 | 1.860 | Age, sex, stage, ECOG PS, LOH, NLR, hemoglobin, platelets, TMB, FCNA, HLA-I evolutionary divergence, and treatment type | Multivariable Cox regression | 23/48; 31/62 | Primary adjusted analysis |
| Nie 2024 | Nie (2) | Chowell-Immu | OS | HR | Unadjusted | 1.020 | 0.590 | 1.750 | None (unadjusted estimate) | Reported crude/univariable HR | 23/48; 31/62 | Separate unadjusted sensitivity analysis |
| Tan 2024 | Tan | Overall cohort | OS | HR | Unadjusted | 0.150 | 0.000 | 12.945 | None (unadjusted estimate) | Reported crude/univariable HR | 19 vs 37; total N = 56 | Separate unadjusted sensitivity analysis |
| Wang 2024 | Wang | Overall cohort | OS | HR | Adjusted | 0.220 | 0.110 | 0.450 | AFP, BCLC, PVTT | Multivariable Cox regression | 75 vs 51; total N = 126 | Primary adjusted analysis |
| Wang 2024 | Wang | Overall cohort | OS | HR | Unadjusted | 0.390 | 0.210 | 0.700 | None (unadjusted estimate) | Reported crude/univariable HR | 75 vs 51; total N = 126 | Separate unadjusted sensitivity analysis |
| Llerena 2024 | Llerena | Overall cohort | OS | HR | Adjusted | 0.620 | 0.440 | 0.860 | Age, sex, race, cardiovascular disease, nicotine dependence, ECOG PS, and pathological TNM stage | Multivariable Cox regression | 83 vs 83; total N = 166 | Primary adjusted analysis |
| Şen 2024 | Şen | Overall cohort | OS | HR | Unadjusted | 1.060 | 0.690 | 1.640 | None (unadjusted estimate) | Reported crude/univariable HR | 66 vs 47 | Separate unadjusted sensitivity analysis |
| Yamamoto 2024 | Yamamoto | Overall cohort | OS | HR | Adjusted | 0.596 | 0.331 | 1.074 | Age, sex, etiology, type of systemic treatment, Child–Pugh class, BCLC stage, and low SMI | Multivariable Cox regression | 43 vs 73 | Primary adjusted analysis |
| Yamamoto 2024 | Yamamoto | Overall cohort | OS | HR | Unadjusted | 0.486 | 0.283 | 0.836 | None (unadjusted estimate) | Reported crude/univariable HR | 43 vs 73 | Separate unadjusted sensitivity analysis |
| Demir 2025 | Demir | Overall cohort | OS | HR | Unadjusted | 1.010 | 0.640 | 1.560 | None (unadjusted estimate) | Reported crude/univariable HR | NR | Separate unadjusted sensitivity analysis |
| Mastrolonardo 2025 | Mastrolonardo | Overall cohort | OS | HR | Adjusted | 0.700 | 0.670 | 0.730 | PSM: age, sex, race, ethnicity, cardiovascular disease, respiratory disease, smoking, alcohol use disorder, pathological TNM stage, and other cancers | Multivariable Cox regression | 9,217 vs 9,217 | Primary adjusted analysis |
| Çolak 2025 | Çolak | Overall cohort | OS | HR | NR |  |  |  | NR | No extractable HR/OR reported in data.xlsx | 85 vs 41 | Not meta-analysed |
| Krejčí 2025 | Krejčí | Overall cohort | OS | HR | Unadjusted | 0.660 | 0.420 | 1.050 | None (unadjusted estimate) | Reported crude/univariable HR | 88 vs 66 | Separate unadjusted sensitivity analysis |
| Anami 2025 | Anami | Overall cohort | OS | HR | Unadjusted | 0.630 | 0.410 | 0.980 | None (unadjusted estimate) | Reported crude/univariable HR | 65 vs 178 | Separate unadjusted sensitivity analysis |
| Kondo 2018 | Kondo | Overall cohort | PFS | HR | Unadjusted | 0.243 | 0.108 | 0.543 | None (unadjusted estimate) | Reported crude/univariable HR | 39 | Separate unadjusted sensitivity analysis |
| Mcquade 2018 | Mcquade (1) | Ipilimumab + DTIC cohort | PFS | HR | Adjusted | 0.630 | 0.410 | 0.950 | Sex, age, stage, LDH, ECOG PS | Multivariable Cox regression | 207 | Primary adjusted analysis |
| Mcquade 2018 | Mcquade (1) | Ipilimumab + DTIC cohort | PFS | HR | Unadjusted | 0.670 | 0.450 | 0.990 | None (unadjusted estimate) | Reported crude/univariable HR | 207 | Separate unadjusted sensitivity analysis |
| Mcquade 2018 | Mcquade (2) | Anti-PD-1/PD-L1 cohort | PFS | HR | Adjusted | 0.850 | 0.610 | 1.190 | Sex, age, stage, LDH, ECOG PS | Multivariable Cox regression | 330 | Primary adjusted analysis |
| Mcquade 2018 | Mcquade (2) | Anti-PD-1/PD-L1 cohort | PFS | HR | Unadjusted | 0.800 | 0.580 | 1.100 | None (unadjusted estimate) | Reported crude/univariable HR | 330 | Separate unadjusted sensitivity analysis |
| Dumenil 2018 | Dumenil | Overall cohort | PFS | HR | Unadjusted | 0.935 | 0.366 | 2.381 | None (unadjusted estimate) | Reported crude/univariable HR | 67 | Separate unadjusted sensitivity analysis |
| Shiroyama 2018 | Shiroyama | Overall cohort | PFS | HR | Unadjusted | 0.877 | 0.610 | 1.250 | None (unadjusted estimate) | Reported crude/univariable HR | 201 | Separate unadjusted sensitivity analysis |
| Qi 2019 | Qi | Overall cohort | PFS | HR | Unadjusted | 0.751 | 0.458 | 1.232 | None (unadjusted estimate) | Reported crude/univariable HR | 85 | Separate unadjusted sensitivity analysis |
| Martini 2019 | Martini | Overall cohort | PFS | HR | Adjusted | 0.543 | 0.278 | 1.064 | Histology; number of distant metastases; number of prior lines of therapy; prior checkpoint inhibitor; RMH risk group; age; checkpoint inhibitor-indicated histology; ECOG PS; sex; race | Multivariable Cox regression | 81 | Primary adjusted analysis |
| Cortellini 2019 | Cortellini | Overall cohort | PFS | HR | Adjusted | 0.720 | 0.490 | 1.060 | Weight(continuous), irAEs of any grade, primary tumor, sex, treatment line, ECOG-PS, number of metastatic sites | Multivariable Cox regression | 936 | Primary adjusted analysis |
| Cortellini 2019 | Cortellini | Overall cohort | PFS | HR | Unadjusted | 0.480 | 0.370 | 0.640 | None (unadjusted estimate) | Reported crude/univariable HR | 936 | Separate unadjusted sensitivity analysis |
| Donnelly 2019 | Donnelly (1) | Anti-CTLA-4 cohort | PFS | HR | Adjusted | 1.160 | 0.700 | 1.940 | Age; gender; stage; LDH; ECOG PS; number of metastatic sites; BRAF mutation status | Multivariable Cox regression | 200 | Primary adjusted analysis |
| Donnelly 2019 | Donnelly (1) | Anti-CTLA-4 cohort | PFS | HR | Unadjusted | 0.980 | 0.590 | 1.630 | None (unadjusted estimate) | Reported crude/univariable HR | 200 | Separate unadjusted sensitivity analysis |
| Donnelly 2019 | Donnelly (2) | Anti-PD-1 cohort | PFS | HR | Adjusted | 2.460 | 1.030 | 5.890 | Age; gender; stage; LDH; ECOG PS; number of metastatic sites; BRAF mutation status | Multivariable Cox regression | 145 | Primary adjusted analysis |
| Donnelly 2019 | Donnelly (2) | Anti-PD-1 cohort | PFS | HR | Unadjusted | 1.490 | 0.710 | 3.130 | None (unadjusted estimate) | Reported crude/univariable HR | 145 | Separate unadjusted sensitivity analysis |
| Katayama 2019 | Katayama | Overall cohort | PFS | HR | Unadjusted | 0.540 | 0.190 | 1.590 | None (unadjusted estimate) | Reported crude/univariable HR | 35(3 vs 32) | Separate unadjusted sensitivity analysis |
| Kichenadasse 2019 | Kichenadasse | Overall cohort | PFS | HR | Adjusted | 0.870 | 0.720 | 1.040 | Age, sex, race, ECOG PS, histology, number of tumor sites, number of prior treatments, smoking history, PD-L1 expression, LDH, log-transformed CRP, and NLR; model stratified by study | Multivariable Cox regression | 944 | Primary adjusted analysis |
| Kichenadasse 2019 | Kichenadasse | Overall cohort | PFS | HR | Unadjusted | 0.860 | 0.730 | 1.010 | None (unadjusted estimate) | Reported crude/univariable HR | 944 | Separate unadjusted sensitivity analysis |
| Labadie 2019 | Labadie | Overall cohort | PFS | HR | Unadjusted | 0.390 | 0.190 | 0.820 | None (unadjusted estimate) | Reported crude/univariable HR | 90 | Separate unadjusted sensitivity analysis |
| Wang 2019 | Wang | Overall cohort | PFS | HR | Adjusted | 0.610 | 0.420 | 0.890 | ECOG PS; line of treatment; age; sex; cancer type | Multivariable Cox regression | 250 | Primary adjusted analysis |
| Naik 2019 | Naik | Overall cohort | PFS | HR | Adjusted | 1.000 | 0.340 | 2.940 | Age; gender; serum creatinine; treatment; current drinking; smoking; KPS; LDH; stage; Charlson score; hemoglobin; ANC; ALC; albumin; autoimmune disease; diabetes; cardiovascular disease; CKD; BRAF; NRAS; hypertension; hyperlipidemia; comedications; prior treatments; fasting glucose; melanoma type | Multivariable Cox regression | 127/un136 | Primary adjusted analysis |
| Naik 2019 | Naik | Overall cohort | PFS | HR | Unadjusted | 1.030 | 0.530 | 2.000 | None (unadjusted estimate) | Reported crude/univariable HR | 127/un136 | Separate unadjusted sensitivity analysis |
| Cortellini 2020 | Cortellini | Overall cohort | PFS | HR | Adjusted | 0.610 | 0.450 | 0.820 | Age; gender; ECOG-PS; CNS metastases; bone metastases; liver metastases | Multivariable Cox regression | 962 | Primary adjusted analysis |
| Nitipir 2020 | Nitipir | Overall cohort | PFS | HR | NR |  |  |  | NR | No extractable HR/OR reported in data.xlsx | 57 | Not meta-analysed |
| Rutkowski 2020 | Rutkowski | Overall cohort | PFS | HR | Adjusted | 1.010 | 0.740 | 1.390 | Age; sex; BRAF mutation; AJCC stage; ECOG PS; LDH; type of immunotherapy; model stratified by study center | Multivariable Cox regression | 410 | Primary adjusted analysis |
| Ichihara 2020 | Ichihara (1) | Pembrolizumab cohort (N=84) | PFS | HR | Unadjusted | 0.940 | 0.530 | 1.650 | None (unadjusted estimate) | Reported crude/univariable HR | 84 | Separate unadjusted sensitivity analysis |
| Ichihara 2020 | Ichihara (2) | Mixed anti-PD-1/PD-L1 cohort (N=429) | PFS | HR | Unadjusted | 0.580 | 0.270 | 1.280 | None (unadjusted estimate) | Reported crude/univariable HR | 429 | Separate unadjusted sensitivity analysis |
| Rogado 2020 | Rogado | Overall cohort | PFS | HR | Adjusted | 0.580 | 0.380 | 0.890 | Sex, histology, smoking status, prior treatments, anti-PD-1 agent, ECOG PS, and other covariates | Multivariable Cox regression | 128 | Primary adjusted analysis |
| Minami 2020 | Minami | Overall cohort | PFS | HR | Adjusted | 0.900 | 0.450 | 1.820 | Albumin, NLR, ECOG-PS, EGFR/ALK mutation | Multivariable Cox regression | 74 | Primary adjusted analysis |
| Ahmed 2021 | Ahmed | Overall cohort | PFS | HR | Adjusted | 0.870 | 0.590 | 1.280 | Age, sex, race, cancer type, anti-CTLA4, concurrent/sequential therapy, dosing strategy, BMI×dosing interaction | Multivariable Cox regression | 297 | Primary adjusted analysis |
| Ahmed 2021 | Ahmed | Overall cohort | PFS | HR | Unadjusted | 0.690 | 0.510 | 0.940 | None (unadjusted estimate) | Reported crude/univariable HR | 297 | Separate unadjusted sensitivity analysis |
| Bartl 2021 | Bartl | Overall cohort | PFS | HR | Adjusted | 0.270 | 0.120 | 0.610 | NLR, CCI, subcutaneous fat volum | Multivariable Cox regression | 36 | Primary adjusted analysis |
| Bartl 2021 | Bartl | Overall cohort | PFS | HR | Unadjusted | 0.650 | 0.430 | 0.970 | None (unadjusted estimate) | Reported crude/univariable HR | 36 | Separate unadjusted sensitivity analysis |
| Imai 2021 | Imai | Overall cohort | PFS | HR | Unadjusted | 0.690 | 0.450 | 1.030 | None (unadjusted estimate) | Reported crude/univariable HR | 142 | Separate unadjusted sensitivity analysis |
| Akce 2021 | Akce | Overall cohort | PFS | HR | Adjusted | 1.040 | 0.560 | 1.920 | Baseline Child–Pugh score and PLR | Multivariable Cox regression | 57 | Primary adjusted analysis |
| Esposito 2021 | Esposito | Overall cohort | PFS | HR | Adjusted | 0.800 | 0.540 | 1.190 | Age, ECOG PS, sex/breast cancer, albumin, number of metastatic sites, NLR, number of prior treatment lines, and prior immunotherapy | Multivariable Cox regression | 153 | Primary adjusted analysis |
| Esposito 2021 | Esposito | Overall cohort | PFS | HR | Unadjusted | 0.790 | 0.550 | 1.140 | None (unadjusted estimate) | Reported crude/univariable HR | 153 | Separate unadjusted sensitivity analysis |
| Zhang 2021 | Zhang | Overall cohort | PFS | HR | Adjusted | 0.760 | 0.550 | 1.040 | Age, sex, ECOG PS, smoking status, and time-dependent irAE | Multivariable Cox regression | 200 | Primary adjusted analysis |
| Chen 2022 | Chen | Overall cohort | PFS | HR | Adjusted | 0.470 | 0.220 | 0.980 | AFP, tumor size, irAEs | Multivariable Cox regression | 101 | Primary adjusted analysis |
| Ged 2022 | Ged | Overall cohort | PFS | HR | Unadjusted | 0.990 | 0.710 | 1.380 | None (unadjusted estimate) | Reported crude/univariable HR | 205 | Separate unadjusted sensitivity analysis |
| Imai 2022 | Imai | Overall cohort | PFS | HR | Adjusted | 0.780 | 0.510 | 1.190 | ECOG performance status | Multivariable Cox regression | 99 | Primary adjusted analysis |
| Imai 2022 | Imai | Overall cohort | PFS | HR | Unadjusted | 0.720 | 0.470 | 1.100 | None (unadjusted estimate) | Reported crude/univariable HR | 99 | Separate unadjusted sensitivity analysis |
| Wang 2022 | Wang | Overall cohort | PFS | HR | Adjusted | 0.440 | 0.170 | 1.130 | Age, sex, IMDC risk category, prior nephrectomy, visceral metastasis, bone metastasis | Multivariable Cox regression | 201 | Primary adjusted analysis |
| Wang 2022 | Wang | Overall cohort | PFS | HR | Unadjusted | 0.610 | 0.270 | 1.420 | None (unadjusted estimate) | Reported crude/univariable HR | 201 | Separate unadjusted sensitivity analysis |
| Tateishi 2022 | Tateishi | Overall cohort | PFS | HR | Adjusted | 1.000 | 0.400 | 2.450 | Age, sex, smoking, ECOG PS, PD-L1 TPS | Multivariable Cox regression | 324 | Primary adjusted analysis |
| Tateishi 2022 | Tateishi | Overall cohort | PFS | HR | Unadjusted | 0.860 | 0.600 | 1.250 | None (unadjusted estimate) | Reported crude/univariable HR | 324 | Separate unadjusted sensitivity analysis |
| Wasamoto 2023 | Wasamoto | Overall cohort | PFS | HR | Unadjusted | 0.730 | 0.450 | 1.160 | None (unadjusted estimate) | Reported crude/univariable HR | 84 | Separate unadjusted sensitivity analysis |
| Incorvaia 2023 | Incorvaia | Overall cohort | PFS | HR | Adjusted | 0.180 | 0.030 | 0.930 | Age, baseline platelet count | Multivariable Cox regression | 32 | Primary adjusted analysis |
| Incorvaia 2023 | Incorvaia | Overall cohort | PFS | HR | Unadjusted | 0.120 | 0.030 | 0.530 | None (unadjusted estimate) | Reported crude/univariable HR | 32 | Separate unadjusted sensitivity analysis |
| Jain 2023 | Jain | Overall cohort | PFS | HR | Adjusted | 0.700 | 0.420 | 1.140 | Age, sex, ECOG PS, histology, and number of prior treatment lines | Multivariable Cox regression | 200 | Primary adjusted analysis |
| Vithayathil 2023 | Vithayathil | Overall cohort | PFS | HR | Adjusted | 0.800 | 0.410 | 1.560 | BCLC stage, Child–Turcotte–Pugh class, tumor diameter, macrovascular invasion, metastatic disease, and AFP | Multivariable Cox regression | NR | Primary adjusted analysis |
| Vithayathil 2023 | Vithayathil | Overall cohort | PFS | HR | Unadjusted | 1.110 | 0.600 | 2.060 | None (unadjusted estimate) | Reported crude/univariable HR | NR | Separate unadjusted sensitivity analysis |
| Wu 2023 | Wu | Overall cohort | PFS | HR | Unadjusted | 0.720 | 0.390 | 1.320 | None (unadjusted estimate) | Reported crude/univariable HR | 78 | Separate unadjusted sensitivity analysis |
| Zhang 2023 | Zhang | Overall cohort | PFS | HR | Adjusted | 0.863 | 0.091 | 8.156 | Sex, prior treatment, serum albumin, ECOG PS, CPS, and T stage of the recurrent tumor | Multivariable Cox regression | 49 | Primary adjusted analysis |
| Zhang 2023 | Zhang | Overall cohort | PFS | HR | Unadjusted | 0.489 | 0.064 | 3.737 | None (unadjusted estimate) | Reported crude/univariable HR | 49 | Separate unadjusted sensitivity analysis |
| McManus 2023 | McManus | Overall cohort | PFS | HR | Adjusted | 1.859 | 1.156 | 2.989 | Age, sex, and IMDC risk score | Multivariable Cox regression | 99 | Primary adjusted analysis |
| McManus 2023 | McManus | Overall cohort | PFS | HR | Unadjusted | 1.765 | 1.119 | 2.783 | None (unadjusted estimate) | Reported crude/univariable HR | 99 | Separate unadjusted sensitivity analysis |
| Miyamoto 2023 | Miyamoto | Overall cohort | PFS | HR | Adjusted | 1.180 | 0.760 | 1.820 | Age, ECOG PS, treatment line, and HPV status | Multivariable Cox regression | 106 | Primary adjusted analysis |
| Miyamoto 2023 | Miyamoto | Overall cohort | PFS | HR | Unadjusted | 1.080 | 0.710 | 1.640 | None (unadjusted estimate) | Reported crude/univariable HR | 106 | Separate unadjusted sensitivity analysis |
| Santoni 2023 | Santoni | Overall cohort | PFS | HR | Unadjusted | 0.790 | 0.610 | 1.020 | None (unadjusted estimate) | Reported crude/univariable HR | 675 | Separate unadjusted sensitivity analysis |
| Alam 2024 | Alam | Overall cohort | PFS | HR | Adjusted | 0.940 | 0.620 | 1.440 | Age, sex, disease stage, immunosuppression status, and ECOG PS | Multivariable Cox regression | 169 | Primary adjusted analysis |
| Bernard 2024 | Bernard | Overall cohort | PFS | HR | Unadjusted | 0.380 | 0.148 | 0.960 | None (unadjusted estimate) | Reported crude/univariable HR | 44 | Separate unadjusted sensitivity analysis |
| Gómez-Banoy 2024 | Gómez-Banoy | Overall cohort | PFS | HR | Adjusted | 0.540 | 0.420 | 0.710 | Age; self-reported race; histology; checkpoint inhibitor treatment; combination therapies; baseline ECOG performance status; stage at diagnosis; previous lines of therapy; previous pelvic radiotherapy | Multivariable Cox regression | 233 vs 128; total cohort N = 524 | Primary adjusted analysis |
| Gómez-Banoy 2024 | Gómez-Banoy | Overall cohort | PFS | HR | Unadjusted | 0.610 | 0.470 | 0.770 | None (unadjusted estimate) | Reported crude/univariable HR | 233 vs 128; total cohort N = 524 | Separate unadjusted sensitivity analysis |
| Nie 2024 | Nie (1) | CGDB-Immu | PFS | HR | Adjusted | 1.010 | 0.840 | 1.210 | Age, sex, race, ECOG PS, tumor type, smoking status, stage, metastatic sites, gene mutations, tissue TMB, and PD-L1 | Multivariable Cox regression | 184/298; 331/618 | Primary adjusted analysis |
| Nie 2024 | Nie (1) | CGDB-Immu | PFS | HR | Unadjusted | 1.050 | 0.880 | 1.260 | None (unadjusted estimate) | Reported crude/univariable HR | 184/298; 331/618 | Separate unadjusted sensitivity analysis |
| Nie 2024 | Nie (2) | Chowell-Immu | PFS | HR | Adjusted | 1.140 | 0.680 | 1.920 | Age, sex, stage, ECOG PS, LOH, NLR, hemoglobin, platelets, TMB, FCNA, HLA-I evolutionary divergence, and treatment type | Multivariable Cox regression | 37/48; 44/62 | Primary adjusted analysis |
| Nie 2024 | Nie (2) | Chowell-Immu | PFS | HR | Unadjusted | 1.050 | 0.680 | 1.630 | None (unadjusted estimate) | Reported crude/univariable HR | 37/48; 44/62 | Separate unadjusted sensitivity analysis |
| Tan 2024 | Tan | Overall cohort | PFS | HR | Adjusted | 0.022 | 0.001 | 0.326 | Alcohol history, radiotherapy history, radiotherapy, cN, smoking history, hypertension, family history, PNI | Multivariable Cox regression | 19 vs 37; total N = 56 | Primary adjusted analysis |
| Tan 2024 | Tan | Overall cohort | PFS | HR | Unadjusted | 0.099 | 0.013 | 0.768 | None (unadjusted estimate) | Reported crude/univariable HR | 19 vs 37; total N = 56 | Separate unadjusted sensitivity analysis |
| Wang 2024 | Wang | Overall cohort | PFS | HR | Adjusted | 0.320 | 0.170 | 0.590 | Age, BCLC stage, Child–Pugh class, ECOG PS, and total bilirubin | Multivariable Cox regression | 75 vs 51; total N = 126 | Primary adjusted analysis |
| Wang 2024 | Wang | Overall cohort | PFS | HR | Unadjusted | 0.280 | 0.150 | 0.520 | None (unadjusted estimate) | Reported crude/univariable HR | 75 vs 51; total N = 126 | Separate unadjusted sensitivity analysis |
| Şen 2024 | Şen | Overall cohort | PFS | HR | Unadjusted | 1.160 | 0.760 | 1.750 | None (unadjusted estimate) | Reported crude/univariable HR | 66 vs 47 | Separate unadjusted sensitivity analysis |
| Demir 2025 | Demir | Overall cohort | PFS | HR | Unadjusted | 0.820 | 0.590 | 1.310 | None (unadjusted estimate) | Reported crude/univariable HR | NR | Separate unadjusted sensitivity analysis |
| Anami 2025 | Anami | Overall cohort | PFS | HR | Unadjusted | 0.850 | 0.630 | 1.140 | None (unadjusted estimate) | Reported crude/univariable HR | 65 vs 178 | Separate unadjusted sensitivity analysis |
| Heidelberger 2017 | Heidelberger | Overall cohort | ORR | OR | NR |  |  |  | NR | No extractable HR/OR reported in data.xlsx | 60 | Not meta-analysed |
| Cortellini 2019 | Cortellini | Overall cohort | ORR | OR | Adjusted | 1.560 | 1.061 | 2.295 | Any-grade irAEs, primary tumor, treatment line, metastatic sites, ECOG-PS | Calculated from reported regression coefficient and SE | 910 | Primary adjusted analysis |
| Cortellini 2019 | Cortellini | Overall cohort | ORR | OR | Unadjusted | 2.668 | 1.990 | 3.577 | None (unadjusted estimate) | Calculated from reported regression coefficient and SE | 910 | Separate unadjusted sensitivity analysis |
| Giorgi 2019 | Giorgi | Overall cohort | ORR | OR | Unadjusted | 1.168 | 0.675 | 2.022 | None (unadjusted estimate) | OR derived from reported 2×2 data or crude model | 268 | Separate unadjusted sensitivity analysis |
| Cortellini 2020 | Cortellini | Overall cohort | ORR | OR | Adjusted | 1.610 | 1.040 | 2.500 | Age; gender; ECOG-PS; CNS metastases; bone metastases; liver metastases | Multivariable logistic/regression model | 840 | Primary adjusted analysis |
| Nitipir 2020 | Nitipir | Overall cohort | ORR | OR | NR |  |  |  | NR | No extractable HR/OR reported in data.xlsx | 57 | Not meta-analysed |
| Rogado 2020 | Rogado | Overall cohort | ORR | OR | Unadjusted | 2.400 | 0.810 | 7.130 | None (unadjusted estimate) | OR derived from reported 2×2 data or crude model | 82 | Separate unadjusted sensitivity analysis |
| Minami 2020 | Minami | Overall cohort | ORR | OR | Unadjusted | 1.060 | 0.200 | 5.550 | None (unadjusted estimate) | OR derived from reported 2×2 data or crude model | 74 | Separate unadjusted sensitivity analysis |
| Bartl 2021 | Bartl | Overall cohort | ORR | OR | Adjusted | 64.090 | 1.900 | 2160.480 | NLR, CCI, subcutaneous fat volume | Multivariable logistic/regression model | 36 | Primary adjusted analysis |
| Bartl 2021 | Bartl | Overall cohort | ORR | OR | Unadjusted | 10.930 | 2.390 | 49.820 | None (unadjusted estimate) | OR derived from reported 2×2 data or crude model | 36 | Separate unadjusted sensitivity analysis |
| Imai 2021 | Imai | Overall cohort | ORR | OR | Unadjusted | 3.370 | 1.650 | 6.860 | None (unadjusted estimate) | OR derived from reported 2×2 data or crude model | 142 | Separate unadjusted sensitivity analysis |
| Esposito 2021 | Esposito | Overall cohort | ORR | OR | Adjusted | 0.960 | 0.300 | 3.100 | ECOG PS, number of metastatic sites, and number of prior treatment lines | Multivariable logistic/regression model | 148 | Primary adjusted analysis |
| Esposito 2021 | Esposito | Overall cohort | ORR | OR | Unadjusted | 0.930 | 0.310 | 2.790 | None (unadjusted estimate) | OR derived from reported 2×2 data or crude model | 148 | Separate unadjusted sensitivity analysis |
| Chen 2022 | Chen | Overall cohort | ORR | OR | Unadjusted | 2.290 | 0.600 | 8.670 | None (unadjusted estimate) | OR derived from reported 2×2 data or crude model | 101 | Separate unadjusted sensitivity analysis |
| Ged 2022 | Ged | Overall cohort | ORR | OR | Unadjusted | 0.890 | 0.440 | 1.800 | None (unadjusted estimate) | OR derived from reported 2×2 data or crude model | 192 | Separate unadjusted sensitivity analysis |
| Imai 2022 | Imai | Overall cohort | ORR | OR | Unadjusted | 1.060 | 0.390 | 2.880 | None (unadjusted estimate) | OR derived from reported 2×2 data or crude model | 99 | Separate unadjusted sensitivity analysis |
| Tateishi 2022 | Tateishi | Overall cohort | ORR | OR | Unadjusted | 0.870 | 0.430 | 1.730 | None (unadjusted estimate) | OR derived from reported 2×2 data or crude model | 324 | Separate unadjusted sensitivity analysis |
| Wasamoto 2023 | Wasamoto | Overall cohort | ORR | OR | Unadjusted | 1.610 | 0.610 | 4.270 | None (unadjusted estimate) | OR derived from reported 2×2 data or crude model | 84 | Separate unadjusted sensitivity analysis |
| Incorvaia 2023 | Incorvaia | Overall cohort | ORR | OR | Unadjusted | 0.480 | 0.110 | 2.040 | None (unadjusted estimate) | OR derived from reported 2×2 data or crude model | 32 | Separate unadjusted sensitivity analysis |
| Jain 2023 | Jain | Overall cohort | ORR | OR | Adjusted | 2.670 | 1.110 | 6.400 | Age, sex, and ECOG PS | Multivariable logistic/regression model | 200 | Primary adjusted analysis |
| Vithayathil 2023 | Vithayathil | Overall cohort | ORR | OR | Unadjusted | 1.330 | 0.650 | 2.710 | None (unadjusted estimate) | OR derived from reported 2×2 data or crude model | 163 | Separate unadjusted sensitivity analysis |
| McManus 2023 | McManus | Overall cohort | ORR | OR | Adjusted | 0.268 | 0.078 | 0.773 | Age, sex, and IMDC risk score | Multivariable logistic/regression model | 99 | Primary adjusted analysis |
| McManus 2023 | McManus | Overall cohort | ORR | OR | Unadjusted | 0.364 | 0.132 | 1.005 | None (unadjusted estimate) | OR derived from reported 2×2 data or crude model | 99 | Separate unadjusted sensitivity analysis |
| Miyamoto 2023 | Miyamoto | Overall cohort | ORR | OR | Unadjusted | 0.720 | 0.320 | 1.640 | None (unadjusted estimate) | OR derived from reported 2×2 data or crude model | 99 | Separate unadjusted sensitivity analysis |
| Alam 2024 | Alam | Overall cohort | ORR | OR | Adjusted | 1.180 | 0.810 | 1.730 | Age, sex, disease stage, immunosuppression status, and ECOG PS | Multivariable logistic/regression model | NR | Primary adjusted analysis |
| Bernard 2024 | Bernard | Overall cohort | ORR | OR | Adjusted | 2.620 | 0.660 | 10.780 | Age and prior pelvic radiotherapy or pelvic radiotherapy plus brachytherapy | Multivariable logistic/regression model | 44 | Primary adjusted analysis |
| Bernard 2024 | Bernard | Overall cohort | ORR | OR | Unadjusted | 3.200 | 0.900 | 12.300 | None (unadjusted estimate) | OR derived from reported 2×2 data or crude model | 44 | Separate unadjusted sensitivity analysis |
| Nie 2024 | Nie | Overall cohort | ORR | OR | Unadjusted | 1.520 | 0.700 | 3.300 | None (unadjusted estimate) | OR derived from reported 2×2 data or crude model | 48 vs 62 | Separate unadjusted sensitivity analysis |
| Wang 2024 | Wang | Overall cohort | ORR | OR | Adjusted | 6.220 | 1.750 | 22.170 | PFS: age, BCLC stage, Child–Pugh class, ECOG PS, and total bilirubin. OS: AFP, BCLC stage, and PVTT | Multivariable logistic/regression model | 126 | Primary adjusted analysis |
| Şen 2024 | Şen | Overall cohort | ORR | OR | Unadjusted | 0.840 | 0.280 | 2.560 | None (unadjusted estimate) | OR derived from reported 2×2 data or crude model | 20 vs 41 | Separate unadjusted sensitivity analysis |
| Yamamoto 2024 | Yamamoto | Overall cohort | ORR | OR | Unadjusted | 1.170 | 0.350 | 3.880 | None (unadjusted estimate) | OR derived from reported 2×2 data or crude model | 19 vs 30 | Separate unadjusted sensitivity analysis |
| Demir 2025 | Demir | Overall cohort | ORR | OR | Unadjusted | 0.980 | 0.600 | 1.600 | None (unadjusted estimate) | OR derived from reported 2×2 data or crude model | 140 vs 160 | Separate unadjusted sensitivity analysis |
| Krejčí 2025 | Krejčí | Overall cohort | ORR | OR | Unadjusted | 2.210 | 1.090 | 4.510 | None (unadjusted estimate) | OR derived from reported 2×2 data or crude model | 88 vs 66 | Separate unadjusted sensitivity analysis |
| Anami 2025 | Anami | Overall cohort | ORR | OR | Unadjusted | 1.400 | 0.740 | 2.620 | None (unadjusted estimate) | OR derived from reported 2×2 data or crude model | 65 vs 178 | Separate unadjusted sensitivity analysis |
| Cortellini 2019 | Cortellini | Overall cohort | Any-grade irAEs | OR | Unadjusted | 3.713 | 2.831 | 4.870 | None (unadjusted estimate) | OR derived from reported 2×2 data or crude model | 976 | Separate unadjusted sensitivity analysis |
| Kichenadasse 2019 | Kichenadasse | Overall cohort | Any-grade irAEs | OR | Unadjusted | 1.340 | 0.970 | 1.850 | None (unadjusted estimate) | OR derived from reported 2×2 data or crude model | 931 | Separate unadjusted sensitivity analysis |
| Labadie 2019 | Labadie | Overall cohort | Any-grade irAEs | OR | NR |  |  |  | NR | No extractable HR/OR reported in data.xlsx | 90 | Not meta-analysed |
| Rogado 2020 | Rogado | Overall cohort | Any-grade irAEs | OR | Unadjusted | 1.420 | 0.680 | 2.970 | None (unadjusted estimate) | OR derived from reported 2×2 data or crude model | 128 | Separate unadjusted sensitivity analysis |
| Bartl 2021 | Bartl | Overall cohort | Any-grade irAEs | OR | Unadjusted | 1.310 | 0.230 | 7.570 | None (unadjusted estimate) | OR derived from reported 2×2 data or crude model | 36 | Separate unadjusted sensitivity analysis |
| Collet 2021 | Collet | Overall cohort | Any-grade irAEs | OR | Unadjusted | 1.370 | 0.830 | 2.280 | None (unadjusted estimate) | OR derived from reported 2×2 data or crude model | 272 | Separate unadjusted sensitivity analysis |
| Esposito 2021 | Esposito | Overall cohort | Any-grade irAEs | OR | Unadjusted | 0.760 | 0.320 | 1.780 | None (unadjusted estimate) | OR derived from reported 2×2 data or crude model | 153 | Separate unadjusted sensitivity analysis |
| Chen 2022 | Chen | Overall cohort | Any-grade irAEs | OR | Unadjusted | 0.540 | 0.140 | 2.020 | None (unadjusted estimate) | OR derived from reported 2×2 data or crude model | 101 | Separate unadjusted sensitivity analysis |
| Vithayathil 2023 | Vithayathil | Overall cohort | Any-grade irAEs | OR | Unadjusted | 1.010 | 0.570 | 1.800 | None (unadjusted estimate) | OR derived from reported 2×2 data or crude model | 191 | Separate unadjusted sensitivity analysis |
| Zhang 2023 | Zhang | Overall cohort | Any-grade irAEs | OR | Unadjusted | 2.480 | 0.490 | 12.590 | None (unadjusted estimate) | OR derived from reported 2×2 data or crude model | NR | Separate unadjusted sensitivity analysis |
| Gómez-Banoy 2024 | Gómez-Banoy | Overall cohort | Any-grade irAEs | OR | Unadjusted | 1.870 | 1.210 | 2.910 | None (unadjusted estimate) | OR derived from reported 2×2 data or crude model | 233 vs 128 | Separate unadjusted sensitivity analysis |
| Llerena 2024 | Llerena | Overall cohort | Any-grade irAEs | OR | Adjusted | 0.700 | 0.370 | 1.310 | Age, sex, race, cardiovascular disease, nicotine dependence, ECOG PS, and pathological TNM stage | Multivariable logistic/regression model | 83 vs 83 | Primary adjusted analysis |
| Şen 2024 | Şen | Overall cohort | Any-grade irAEs | OR | Unadjusted | 2.280 | 0.910 | 5.710 | None (unadjusted estimate) | OR derived from reported 2×2 data or crude model | 66 vs 47 | Separate unadjusted sensitivity analysis |
| Mastrolonardo 2025 | Mastrolonardo | Overall cohort | Any-grade irAEs | OR | Adjusted | 1.160 | 1.100 | 1.240 | PSM: age, sex, race, ethnicity, cardiovascular disease, respiratory disease, smoking, alcohol use disorder, pathological TNM stage, and other cancers | Multivariable logistic/regression model | 9,217 vs 9,217 | Primary adjusted analysis |
| Anami 2025 | Anami | Overall cohort | Any-grade irAEs | OR | Unadjusted | 1.120 | 0.630 | 1.990 | None (unadjusted estimate) | OR derived from reported 2×2 data or crude model | 65 vs 178 | Separate unadjusted sensitivity analysis |
| Heidelberger 2017 | Heidelberger | Overall cohort | Grade ≥3 irAEs | OR | Adjusted | 4.080 | 0.950 | 17.590 | NR | Multivariable logistic/regression model | NR | Primary adjusted analysis |
| Dumenil 2018 | Dumenil | Overall cohort | Grade ≥3 irAEs | OR | Unadjusted | 2.840 | 0.540 | 14.870 | None (unadjusted estimate) | OR derived from reported 2×2 data or crude model | 67 | Separate unadjusted sensitivity analysis |
| Cortellini 2019 | Cortellini | Overall cohort | Grade ≥3 irAEs | OR | Unadjusted | 1.484 | 0.884 | 2.491 | None (unadjusted estimate) | OR derived from reported 2×2 data or crude model | 976 | Separate unadjusted sensitivity analysis |
| Bartl 2021 | Bartl | Overall cohort | Grade ≥3 irAEs | OR | Unadjusted | 3.970 | 0.150 | 104.180 | None (unadjusted estimate) | OR derived from reported 2×2 data or crude model | 36 | Separate unadjusted sensitivity analysis |
| Collet 2021 | Collet | Overall cohort | Grade ≥3 irAEs | OR | Unadjusted | 1.220 | 0.580 | 2.570 | None (unadjusted estimate) | OR derived from reported 2×2 data or crude model | 36 | Separate unadjusted sensitivity analysis |
| Esposito 2021 | Esposito | Overall cohort | Grade ≥3 irAEs | OR | Unadjusted | 1.000 | 0.250 | 4.040 | None (unadjusted estimate) | OR derived from reported 2×2 data or crude model | 153 | Separate unadjusted sensitivity analysis |
| Vithayathil 2023 | Vithayathil | Overall cohort | Grade ≥3 irAEs | OR | Unadjusted | 0.350 | 0.110 | 1.130 | None (unadjusted estimate) | OR derived from reported 2×2 data or crude model | 191 | Separate unadjusted sensitivity analysis |

**Supplementary Table S4.**Cohort definitions, potential population overlap, and handling of multiple estimates

| **Study** | **Cohort name/source** | **Cancer type** | **Independent cohort?** | **Overlap assessment** | **Potential overlap group** | **Multiple estimates?** | **Handling** |
| --- | --- | --- | --- | --- | --- | --- | --- |
| Heidelberger 2017 | Overall cohort | Melanoma | Single reported cohort | No known overlap identified from the extracted report/source information | None identified | Yes—multiple BMI categories/outcomes | Use one eligible estimate per cohort/outcome; adjusted estimate is primary, unadjusted estimate is kept separate; correlated BMI categories are modeled with covariance in dose-response analysis. |
| Kondo 2018 | Overall cohort | Melanoma | Single reported cohort | No known overlap identified from the extracted report/source information | None identified | Yes—multiple BMI categories/outcomes | Use one eligible estimate per cohort/outcome; adjusted estimate is primary, unadjusted estimate is kept separate; correlated BMI categories are modeled with covariance in dose-response analysis. |
| Mcquade 2018 | Ipilimumab + DTIC cohort | Melanoma | Yes | No known overlap identified from the extracted report/source information | None identified | Yes—independent cohorts | Retain independent cohorts as separate estimates; use adjusted estimate as primary and unadjusted estimate separately. For dose-response, combine cohort slopes within publication before between-publication pooling. |
| Mcquade 2018 | Anti-PD-1/PD-L1 cohort | Melanoma | Yes | No known overlap identified from the extracted report/source information | None identified | Yes—independent cohorts | Retain independent cohorts as separate estimates; use adjusted estimate as primary and unadjusted estimate separately. For dose-response, combine cohort slopes within publication before between-publication pooling. |
| Dumenil 2018 | Overall cohort | NSCLC | Single reported cohort | No known overlap identified from the extracted report/source information | None identified | Yes—multiple BMI categories/outcomes | Use one eligible estimate per cohort/outcome; adjusted estimate is primary, unadjusted estimate is kept separate; correlated BMI categories are modeled with covariance in dose-response analysis. |
| Shiroyama 2018 | Overall cohort | NSCLC | Single reported cohort | No known overlap identified from the extracted report/source information | None identified | Yes—multiple BMI categories/outcomes | Use one eligible estimate per cohort/outcome; adjusted estimate is primary, unadjusted estimate is kept separate; correlated BMI categories are modeled with covariance in dose-response analysis. |
| Qi 2019 | Overall cohort | Multiple | Single reported cohort | No known overlap identified from the extracted report/source information | None identified | Yes—multiple BMI categories/outcomes | Use one eligible estimate per cohort/outcome; adjusted estimate is primary, unadjusted estimate is kept separate; correlated BMI categories are modeled with covariance in dose-response analysis. |
| Martini 2019 | Overall cohort | Multiple | Single reported cohort | No known overlap identified from the extracted report/source information | None identified | Yes—multiple BMI categories/outcomes | Use one eligible estimate per cohort/outcome; adjusted estimate is primary, unadjusted estimate is kept separate; correlated BMI categories are modeled with covariance in dose-response analysis. |
| Cortellini 2019 | Overall cohort | Multiple | Single reported cohort | No known overlap identified from the extracted report/source information | None identified | Yes—multiple BMI categories/outcomes | Use one eligible estimate per cohort/outcome; adjusted estimate is primary, unadjusted estimate is kept separate; correlated BMI categories are modeled with covariance in dose-response analysis. |
| Giorgi 2019 | Overall cohort | RCC | Single reported cohort | No known overlap identified from the extracted report/source information | None identified | Yes—multiple BMI categories/outcomes | Use one eligible estimate per cohort/outcome; adjusted estimate is primary, unadjusted estimate is kept separate; correlated BMI categories are modeled with covariance in dose-response analysis. |
| Donnelly 2019 | Anti-CTLA-4 cohort | Melanoma | Yes | No known overlap identified from the extracted report/source information | None identified | Yes—independent cohorts | Retain independent cohorts as separate estimates; use adjusted estimate as primary and unadjusted estimate separately. For dose-response, combine cohort slopes within publication before between-publication pooling. |
| Donnelly 2019 | Anti-PD-1 cohort | Melanoma | Yes | No known overlap identified from the extracted report/source information | None identified | Yes—independent cohorts | Retain independent cohorts as separate estimates; use adjusted estimate as primary and unadjusted estimate separately. For dose-response, combine cohort slopes within publication before between-publication pooling. |
| Katayama 2019 | Overall cohort | NSCLC | Single reported cohort | No known overlap identified from the extracted report/source information | None identified | Yes—multiple BMI categories/outcomes | Use one eligible estimate per cohort/outcome; adjusted estimate is primary, unadjusted estimate is kept separate; correlated BMI categories are modeled with covariance in dose-response analysis. |
| Kichenadasse 2019 | Overall cohort | NSCLC | Single reported cohort | No known overlap identified from the extracted report/source information | None identified | Yes—multiple BMI categories/outcomes | Use one eligible estimate per cohort/outcome; adjusted estimate is primary, unadjusted estimate is kept separate; correlated BMI categories are modeled with covariance in dose-response analysis. |
| Labadie 2019 | Overall cohort | RCC | Single reported cohort | No known overlap identified from the extracted report/source information | None identified | Yes—multiple BMI categories/outcomes | Use one eligible estimate per cohort/outcome; adjusted estimate is primary, unadjusted estimate is kept separate; correlated BMI categories are modeled with covariance in dose-response analysis. |
| Magri 2019 | Overall cohort | NSCLC | Single reported cohort | No known overlap identified from the extracted report/source information | None identified | Yes—multiple BMI categories/outcomes | Use one eligible estimate per cohort/outcome; adjusted estimate is primary, unadjusted estimate is kept separate; correlated BMI categories are modeled with covariance in dose-response analysis. |
| Popinat 2019 | Overall cohort | NSCLC | Single reported cohort | No known overlap identified from the extracted report/source information | None identified | Yes—multiple BMI categories/outcomes | Use one eligible estimate per cohort/outcome; adjusted estimate is primary, unadjusted estimate is kept separate; correlated BMI categories are modeled with covariance in dose-response analysis. |
| Wang 2019 | Overall cohort | Multiple | Single reported cohort | No known overlap identified from the extracted report/source information | None identified | Yes—multiple BMI categories/outcomes | Use one eligible estimate per cohort/outcome; adjusted estimate is primary, unadjusted estimate is kept separate; correlated BMI categories are modeled with covariance in dose-response analysis. |
| Naik 2019 | Overall cohort | Melanoma | Single reported cohort | No known overlap identified from the extracted report/source information | None identified | Yes—multiple BMI categories/outcomes | Use one eligible estimate per cohort/outcome; adjusted estimate is primary, unadjusted estimate is kept separate; correlated BMI categories are modeled with covariance in dose-response analysis. |
| Cortellini 2020 | Overall cohort | NSCLC | Single reported cohort | No known overlap identified from the extracted report/source information | None identified | Yes—multiple BMI categories/outcomes | Use one eligible estimate per cohort/outcome; adjusted estimate is primary, unadjusted estimate is kept separate; correlated BMI categories are modeled with covariance in dose-response analysis. |
| Nitipir 2020 | Overall cohort | Multiple | Single reported cohort | No known overlap identified from the extracted report/source information | None identified | Yes—multiple BMI categories/outcomes | Use one eligible estimate per cohort/outcome; adjusted estimate is primary, unadjusted estimate is kept separate; correlated BMI categories are modeled with covariance in dose-response analysis. |
| Rutkowski 2020 | Overall cohort | Melanoma | Single reported cohort | No known overlap identified from the extracted report/source information | None identified | Yes—multiple BMI categories/outcomes | Use one eligible estimate per cohort/outcome; adjusted estimate is primary, unadjusted estimate is kept separate; correlated BMI categories are modeled with covariance in dose-response analysis. |
| Ichihara 2020 | Pembrolizumab cohort (N=84) | NSCLC | Yes | No known overlap identified from the extracted report/source information | None identified | Yes—independent cohorts | Retain independent cohorts as separate estimates; use adjusted estimate as primary and unadjusted estimate separately. For dose-response, combine cohort slopes within publication before between-publication pooling. |
| Ichihara 2020 | Mixed anti-PD-1/PD-L1 cohort (N=429) | NSCLC | Yes | No known overlap identified from the extracted report/source information | None identified | Yes—independent cohorts | Retain independent cohorts as separate estimates; use adjusted estimate as primary and unadjusted estimate separately. For dose-response, combine cohort slopes within publication before between-publication pooling. |
| Rogado 2020 | Overall cohort | Multiple | Single reported cohort | No known overlap identified from the extracted report/source information | None identified | Yes—multiple BMI categories/outcomes | Use one eligible estimate per cohort/outcome; adjusted estimate is primary, unadjusted estimate is kept separate; correlated BMI categories are modeled with covariance in dose-response analysis. |
| Minami 2020 | Overall cohort | NSCLC | Single reported cohort | No known overlap identified from the extracted report/source information | None identified | Yes—multiple BMI categories/outcomes | Use one eligible estimate per cohort/outcome; adjusted estimate is primary, unadjusted estimate is kept separate; correlated BMI categories are modeled with covariance in dose-response analysis. |
| Ahmed 2021 | Overall cohort | Multiple | Single reported cohort | No known overlap identified from the extracted report/source information | None identified | Yes—multiple BMI categories/outcomes | Use one eligible estimate per cohort/outcome; adjusted estimate is primary, unadjusted estimate is kept separate; correlated BMI categories are modeled with covariance in dose-response analysis. |
| Bartl 2021 | Overall cohort | Multiple | Single reported cohort | No known overlap identified from the extracted report/source information | None identified | Yes—multiple BMI categories/outcomes | Use one eligible estimate per cohort/outcome; adjusted estimate is primary, unadjusted estimate is kept separate; correlated BMI categories are modeled with covariance in dose-response analysis. |
| Collet 2021 | Overall cohort | Multiple | Single reported cohort | No known overlap identified from the extracted report/source information | None identified | Yes—multiple BMI categories/outcomes | Use one eligible estimate per cohort/outcome; adjusted estimate is primary, unadjusted estimate is kept separate; correlated BMI categories are modeled with covariance in dose-response analysis. |
| Imai 2021 | Overall cohort | NSCLC | Single reported cohort | No known overlap identified from the extracted report/source information | None identified | Yes—multiple BMI categories/outcomes | Use one eligible estimate per cohort/outcome; adjusted estimate is primary, unadjusted estimate is kept separate; correlated BMI categories are modeled with covariance in dose-response analysis. |
| Akce 2021 | Overall cohort | HCC | Single reported cohort | No known overlap identified from the extracted report/source information | None identified | Yes—multiple BMI categories/outcomes | Use one eligible estimate per cohort/outcome; adjusted estimate is primary, unadjusted estimate is kept separate; correlated BMI categories are modeled with covariance in dose-response analysis. |
| Esposito 2021 | Overall cohort | Multiple | Single reported cohort | No known overlap identified from the extracted report/source information | None identified | Yes—multiple BMI categories/outcomes | Use one eligible estimate per cohort/outcome; adjusted estimate is primary, unadjusted estimate is kept separate; correlated BMI categories are modeled with covariance in dose-response analysis. |
| Zhang 2021 | Overall cohort | Lung | Single reported cohort | No known overlap identified from the extracted report/source information | None identified | Yes—multiple BMI categories/outcomes | Use one eligible estimate per cohort/outcome; adjusted estimate is primary, unadjusted estimate is kept separate; correlated BMI categories are modeled with covariance in dose-response analysis. |
| Chen 2022 | Overall cohort | HCC | Single reported cohort | No known overlap identified from the extracted report/source information | None identified | Yes—multiple BMI categories/outcomes | Use one eligible estimate per cohort/outcome; adjusted estimate is primary, unadjusted estimate is kept separate; correlated BMI categories are modeled with covariance in dose-response analysis. |
| Ged 2022 | Overall cohort | RCC | Single reported cohort | No known overlap identified from the extracted report/source information | None identified | Yes—multiple BMI categories/outcomes | Use one eligible estimate per cohort/outcome; adjusted estimate is primary, unadjusted estimate is kept separate; correlated BMI categories are modeled with covariance in dose-response analysis. |
| Imai 2022 | Overall cohort | NSCLC | Single reported cohort | No known overlap identified from the extracted report/source information | None identified | Yes—multiple BMI categories/outcomes | Use one eligible estimate per cohort/outcome; adjusted estimate is primary, unadjusted estimate is kept separate; correlated BMI categories are modeled with covariance in dose-response analysis. |
| Joshi 2022 | Overall cohort | NSCLC | Single reported cohort | No known overlap identified from the extracted report/source information | None identified | Yes—multiple BMI categories/outcomes | Use one eligible estimate per cohort/outcome; adjusted estimate is primary, unadjusted estimate is kept separate; correlated BMI categories are modeled with covariance in dose-response analysis. |
| Wang 2022 | Overall cohort | RCC | Single reported cohort | No known overlap identified from the extracted report/source information | None identified | Yes—multiple BMI categories/outcomes | Use one eligible estimate per cohort/outcome; adjusted estimate is primary, unadjusted estimate is kept separate; correlated BMI categories are modeled with covariance in dose-response analysis. |
| Tateishi 2022 | Overall cohort | NSCLC | Single reported cohort | No known overlap identified from the extracted report/source information | None identified | Yes—multiple BMI categories/outcomes | Use one eligible estimate per cohort/outcome; adjusted estimate is primary, unadjusted estimate is kept separate; correlated BMI categories are modeled with covariance in dose-response analysis. |
| Wasamoto 2023 | Overall cohort | SCLC | Single reported cohort | No known overlap identified from the extracted report/source information | None identified | Yes—multiple BMI categories/outcomes | Use one eligible estimate per cohort/outcome; adjusted estimate is primary, unadjusted estimate is kept separate; correlated BMI categories are modeled with covariance in dose-response analysis. |
| Incorvaia 2023 | Overall cohort | MCC | Single reported cohort | No known overlap identified from the extracted report/source information | None identified | Yes—multiple BMI categories/outcomes | Use one eligible estimate per cohort/outcome; adjusted estimate is primary, unadjusted estimate is kept separate; correlated BMI categories are modeled with covariance in dose-response analysis. |
| Jain 2023 | Overall cohort | NSCLC | Single reported cohort | No known overlap identified from the extracted report/source information | None identified | Yes—multiple BMI categories/outcomes | Use one eligible estimate per cohort/outcome; adjusted estimate is primary, unadjusted estimate is kept separate; correlated BMI categories are modeled with covariance in dose-response analysis. |
| Vithayathil 2023 | Overall cohort | HCC | Single reported cohort | No known overlap identified from the extracted report/source information | None identified | Yes—multiple BMI categories/outcomes | Use one eligible estimate per cohort/outcome; adjusted estimate is primary, unadjusted estimate is kept separate; correlated BMI categories are modeled with covariance in dose-response analysis. |
| Wu 2023 | Overall cohort | EC | Single reported cohort | No known overlap identified from the extracted report/source information | None identified | Yes—multiple BMI categories/outcomes | Use one eligible estimate per cohort/outcome; adjusted estimate is primary, unadjusted estimate is kept separate; correlated BMI categories are modeled with covariance in dose-response analysis. |
| Zhang 2023 | Overall cohort | HNSCC | Single reported cohort | No known overlap identified from the extracted report/source information | None identified | Yes—multiple BMI categories/outcomes | Use one eligible estimate per cohort/outcome; adjusted estimate is primary, unadjusted estimate is kept separate; correlated BMI categories are modeled with covariance in dose-response analysis. |
| McManus 2023 | Overall cohort | RCC | Single reported cohort | No known overlap identified from the extracted report/source information | None identified | Yes—multiple BMI categories/outcomes | Use one eligible estimate per cohort/outcome; adjusted estimate is primary, unadjusted estimate is kept separate; correlated BMI categories are modeled with covariance in dose-response analysis. |
| Miyamoto 2023 | Overall cohort | HNSCC | Single reported cohort | No known overlap identified from the extracted report/source information | None identified | Yes—multiple BMI categories/outcomes | Use one eligible estimate per cohort/outcome; adjusted estimate is primary, unadjusted estimate is kept separate; correlated BMI categories are modeled with covariance in dose-response analysis. |
| Antoun 2023 | Melanoma cohort | Multiple | Yes | No known overlap identified from the extracted report/source information | None identified | Yes—independent cohorts | Retain independent cohorts as separate estimates; use adjusted estimate as primary and unadjusted estimate separately. For dose-response, combine cohort slopes within publication before between-publication pooling. |
| Antoun 2023 | NSCLC cohort | Multiple | Yes | No known overlap identified from the extracted report/source information | None identified | Yes—independent cohorts | Retain independent cohorts as separate estimates; use adjusted estimate as primary and unadjusted estimate separately. For dose-response, combine cohort slopes within publication before between-publication pooling. |
| Santoni 2023 | Overall cohort | RCC | Single reported cohort | No known overlap identified from the extracted report/source information | None identified | Yes—multiple BMI categories/outcomes | Use one eligible estimate per cohort/outcome; adjusted estimate is primary, unadjusted estimate is kept separate; correlated BMI categories are modeled with covariance in dose-response analysis. |
| Alam 2024 | Overall cohort | MCC | Single reported cohort | No known overlap identified from the extracted report/source information | None identified | Yes—multiple BMI categories/outcomes | Use one eligible estimate per cohort/outcome; adjusted estimate is primary, unadjusted estimate is kept separate; correlated BMI categories are modeled with covariance in dose-response analysis. |
| Bernard 2024 | Overall cohort | EA | Single reported cohort | No known overlap identified from the extracted report/source information | None identified | Yes—multiple BMI categories/outcomes | Use one eligible estimate per cohort/outcome; adjusted estimate is primary, unadjusted estimate is kept separate; correlated BMI categories are modeled with covariance in dose-response analysis. |
| Gómez-Banoy 2024 | Overall cohort | EC | Single reported cohort | No known overlap identified from the extracted report/source information | None identified | Yes—multiple BMI categories/outcomes | Use one eligible estimate per cohort/outcome; adjusted estimate is primary, unadjusted estimate is kept separate; correlated BMI categories are modeled with covariance in dose-response analysis. |
| Nie 2024 | CGDB-Immu | NSCLC | Yes | No known overlap identified from the extracted report/source information | None identified | Yes—independent cohorts | Retain independent cohorts as separate estimates; use adjusted estimate as primary and unadjusted estimate separately. For dose-response, combine cohort slopes within publication before between-publication pooling. |
| Nie 2024 | Chowell-Immu | NSCLC | Yes | No known overlap identified from the extracted report/source information | None identified | Yes—independent cohorts | Retain independent cohorts as separate estimates; use adjusted estimate as primary and unadjusted estimate separately. For dose-response, combine cohort slopes within publication before between-publication pooling. |
| Tan 2024 | Overall cohort | OTSCC | Single reported cohort | No known overlap identified from the extracted report/source information | None identified | Yes—multiple BMI categories/outcomes | Use one eligible estimate per cohort/outcome; adjusted estimate is primary, unadjusted estimate is kept separate; correlated BMI categories are modeled with covariance in dose-response analysis. |
| Wang 2024 | Overall cohort | HCC | Single reported cohort | No known overlap identified from the extracted report/source information | None identified | Yes—multiple BMI categories/outcomes | Use one eligible estimate per cohort/outcome; adjusted estimate is primary, unadjusted estimate is kept separate; correlated BMI categories are modeled with covariance in dose-response analysis. |
| Llerena 2024 | Overall cohort | HNSCC | Single reported cohort | No known overlap identified from the extracted report/source information | None identified | Yes—multiple BMI categories/outcomes | Use one eligible estimate per cohort/outcome; adjusted estimate is primary, unadjusted estimate is kept separate; correlated BMI categories are modeled with covariance in dose-response analysis. |
| Ihara 2024 | Overall cohort | NSCLC | Single reported cohort | No known overlap identified from the extracted report/source information | None identified | No | Use one eligible estimate per cohort/outcome; adjusted estimate is primary, unadjusted estimate is kept separate; correlated BMI categories are modeled with covariance in dose-response analysis. |
| Şen 2024 | Overall cohort | Multiple | Single reported cohort | No known overlap identified from the extracted report/source information | None identified | Yes—multiple BMI categories/outcomes | Use one eligible estimate per cohort/outcome; adjusted estimate is primary, unadjusted estimate is kept separate; correlated BMI categories are modeled with covariance in dose-response analysis. |
| Yamamoto 2024 | Overall cohort | HCC | Single reported cohort | No known overlap identified from the extracted report/source information | None identified | Yes—multiple BMI categories/outcomes | Use one eligible estimate per cohort/outcome; adjusted estimate is primary, unadjusted estimate is kept separate; correlated BMI categories are modeled with covariance in dose-response analysis. |
| Demir 2025 | Overall cohort | NSCLC | Single reported cohort | No known overlap identified from the extracted report/source information | None identified | Yes—multiple BMI categories/outcomes | Use one eligible estimate per cohort/outcome; adjusted estimate is primary, unadjusted estimate is kept separate; correlated BMI categories are modeled with covariance in dose-response analysis. |
| Mastrolonardo 2025 | Overall cohort | Multiple | Single reported cohort | No known overlap identified from the extracted report/source information | None identified | Yes—multiple BMI categories/outcomes | Use one eligible estimate per cohort/outcome; adjusted estimate is primary, unadjusted estimate is kept separate; correlated BMI categories are modeled with covariance in dose-response analysis. |
| Çolak 2025 | Overall cohort | RCC | Single reported cohort | No known overlap identified from the extracted report/source information | None identified | Yes—multiple BMI categories/outcomes | Use one eligible estimate per cohort/outcome; adjusted estimate is primary, unadjusted estimate is kept separate; correlated BMI categories are modeled with covariance in dose-response analysis. |
| Krejčí 2025 | Overall cohort | NSCLC | Single reported cohort | No known overlap identified from the extracted report/source information | None identified | Yes—multiple BMI categories/outcomes | Use one eligible estimate per cohort/outcome; adjusted estimate is primary, unadjusted estimate is kept separate; correlated BMI categories are modeled with covariance in dose-response analysis. |
| Anami 2025 | Overall cohort | RCC | Single reported cohort | No known overlap identified from the extracted report/source information | None identified | Yes—multiple BMI categories/outcomes | Use one eligible estimate per cohort/outcome; adjusted estimate is primary, unadjusted estimate is kept separate; correlated BMI categories are modeled with covariance in dose-response analysis. |

**Supplementary Table S5.**Category-specific data, assigned BMI values, effect estimates, and covariance or transformation methods used in the dose-response analyses

| **Study_ID** | **Outcome** | **Original BMI category** | **Final assigned BMI** | **Dose assignment method** | **Reference category?** | **Final oriented HR** | **Final lower CI** | **Final upper CI** | **Category N** | **Events/cases** | **Increment (kg/m²)** | **Study data type** | **Covariance/GLST method** | **Hamling re-reference?** |
| --- | --- | --- | --- | --- | --- | --- | --- | --- | --- | --- | --- | --- | --- | --- |
| Alam_2024 | OS | Continuous BMI |  | Direct continuous estimate | No | 0.96 | 0.790 | 1.170 | 183 |  | 5.000 | direct_continuous | Direct model | No |
| Bartl_2021 | OS | Continuous BMI |  | Direct continuous estimate | No | 7.44 | 1.620 | 34.160 | 36 |  | 5.000 | direct_continuous | Direct model | No |
| Martini_2019 | OS | Continuous BMI |  | Direct continuous estimate | No | 0.92 | 0.870 | 0.970 | 82 |  | 1.000 | direct_continuous | Direct model | No |
| Joshi_2022 | OS | Continuous BMI |  | Direct continuous estimate | No | 0.984 | 0.914 | 1.061 | 136 |  | 1.000 | direct_continuous | Direct model | No |
| Rutkowski_2020 | OS | Continuous BMI |  | Direct continuous estimate | No | 1.02 | 0.990 | 1.050 | 393 |  | 5.000 | direct_continuous | Direct model | No |
| Magri_2019 | OS | Continuous BMI |  | Direct continuous estimate | No | 1.19 | 0.930 | 1.510 | 46 |  | 1.000 | direct_continuous | Direct model | No |
| Antoun_2023 | OS | Underweight | 15.25 | Open-ended using adjacent interval width | No | 0.53 | 0.260 | 1.020 |  |  | 5.000 | categorical | Independent WLS | Required by planned method, but not feasible (category deaths/person-time NR) |
| Antoun_2023 | OS | Normal | 21.75 | Midpoint | Yes | 1 |  |  |  |  | 5.000 | categorical | Independent WLS | Required by planned method, but not feasible (category deaths/person-time NR) |
| Antoun_2023 | OS | Overweight | 27.5 | Midpoint | No | 0.8 | 0.540 | 1.160 |  |  | 5.000 | categorical | Independent WLS | Required by planned method, but not feasible (category deaths/person-time NR) |
| Antoun_2023 | OS | Obese | 32.5 | Open-ended using adjacent interval width | No | 0.85 | 0.470 | 1.560 |  |  | 5.000 | categorical | Independent WLS | Required by planned method, but not feasible (category deaths/person-time NR) |
| Antoun_2023 | OS | Underweight | 15.25 | Open-ended using adjacent interval width | No | 1.17 | 0.780 | 1.760 |  |  | 5.000 | categorical | Independent WLS | Required by planned method, but not feasible (category deaths/person-time NR) |
| Antoun_2023 | OS | Normal | 21.75 | Midpoint | Yes | 1 |  |  |  |  | 5.000 | categorical | Independent WLS | Required by planned method, but not feasible (category deaths/person-time NR) |
| Antoun_2023 | OS | Overweight | 27.5 | Midpoint | No | 1.38 | 0.890 | 2.140 |  |  | 5.000 | categorical | Independent WLS | Required by planned method, but not feasible (category deaths/person-time NR) |
| Antoun_2023 | OS | Obese | 32.5 | Open-ended using adjacent interval width | No | 1.54 | 0.730 | 3.280 |  |  | 5.000 | categorical | Independent WLS | Required by planned method, but not feasible (category deaths/person-time NR) |
| Cortellini_2020 | OS | Underweight | 15.25 | Open-ended using adjacent interval width | No | 1.22 | 0.740 | 2.010 | 35 |  | 5.000 | categorical | Independent WLS | Required by planned method, but not feasible (category deaths/person-time NR) |
| Cortellini_2020 | OS | Normal | 21.75 | Midpoint | Yes | 1 |  |  | 467 |  | 5.000 | categorical | Independent WLS | Required by planned method, but not feasible (category deaths/person-time NR) |
| Cortellini_2020 | OS | Overweight | 27.5 | Midpoint | No | 0.97 | 0.770 | 1.220 | 232 |  | 5.000 | categorical | Independent WLS | Required by planned method, but not feasible (category deaths/person-time NR) |
| Cortellini_2020 | OS | Obese | 32.5 | Open-ended using adjacent interval width | No | 0.7 | 0.490 | 0.990 | 106 |  | 5.000 | categorical | Independent WLS | Required by planned method, but not feasible (category deaths/person-time NR) |
| Donnelly_2019 | OS | Normal or underweight | 22.5 | Open-ended using adjacent interval width | Yes | 1 |  |  | 64 |  | 5.000 | categorical | Independent WLS | No |
| Donnelly_2019 | OS | Overweight | 27.5 | Midpoint | No | 0.83 | 0.470 | 1.470 | 75 |  | 5.000 | categorical | Independent WLS | No |
| Donnelly_2019 | OS | Obese | 32.5 | Open-ended using adjacent interval width | No | 1.22 | 0.750 | 2.000 | 61 |  | 5.000 | categorical | Independent WLS | No |
| Donnelly_2019 | OS | Normal or underweight | 22.5 | Open-ended using adjacent interval width | Yes | 1 |  |  | 49 |  | 5.000 | categorical | Independent WLS | No |
| Donnelly_2019 | OS | Overweight | 27.5 | Midpoint | No | 1.6 | 0.860 | 2.960 | 55 |  | 5.000 | categorical | Independent WLS | No |
| Donnelly_2019 | OS | Obese | 32.5 | Open-ended using adjacent interval width | No | 1.04 | 0.480 | 2.240 | 41 |  | 5.000 | categorical | Independent WLS | No |
| Donnelly_2019 | OS | Normal or underweight | 22.5 | Open-ended using adjacent interval width | Yes | 1 |  |  | 26 |  | 5.000 | categorical | Independent WLS | No |
| Donnelly_2019 | OS | Overweight | 27.5 | Midpoint | No | 0.7 | 0.200 | 2.470 | 35 |  | 5.000 | categorical | Independent WLS | No |
| Donnelly_2019 | OS | Obese | 32.5 | Open-ended using adjacent interval width | No | 0.92 | 0.240 | 3.490 | 17 |  | 5.000 | categorical | Independent WLS | No |
| McQuade_2018 | OS | Normal | 21.75 | Midpoint | Yes | 1 |  |  | 68 | 56 | 5.000 | categorical | Hamling GLS | No |
| McQuade_2018 | OS | Overweight | 27.5 | Midpoint | No | 0.7 | 0.480 | 1.030 | 88 | 67 | 5.000 | categorical | Hamling GLS | No |
| McQuade_2018 | OS | Obese | 32.5 | Open-ended using adjacent interval width | No | 0.54 | 0.340 | 0.860 | 51 | 35 | 5.000 | categorical | Hamling GLS | No |
| McQuade_2018 | OS | Normal | 21.75 | Midpoint | Yes | 1 |  |  | 102 | 59 | 5.000 | categorical | Hamling GLS | No |
| McQuade_2018 | OS | Overweight | 27.5 | Midpoint | No | 0.78 | 0.520 | 1.170 | 109 | 51 | 5.000 | categorical | Hamling GLS | No |
| McQuade_2018 | OS | Obese | 32.5 | Open-ended using adjacent interval width | No | 0.72 | 0.480 | 1.060 | 118 | 52 | 5.000 | categorical | Hamling GLS | No |
| Naik_2019 | OS | Normal | 22.6 | Reported mean | Yes | 1 |  |  | 35 |  | 5.000 | categorical | Independent WLS | No |
| Naik_2019 | OS | Overweight and class I obese | 29.1 | Reported mean | No | 0.26 | 0.100 | 0.710 | 86 | 27 | 5.000 | categorical | Independent WLS | No |
| Naik_2019 | OS | Class II and III obese | 40.1 | Reported mean | No | 0.42 | 0.100 | 1.770 | 15 | 9 | 5.000 | categorical | Independent WLS | No |
| Vithayathil_2023 | OS | BMI below 25 | 21.9 | Reported mean | Yes | 1 |  |  | 97 |  | 5.000 | binary | Two-group slope | No |
| Vithayathil_2023 | OS | BMI 25 or higher | 29.6 | Reported mean | No | 0.72 | 0.420 | 1.230 | 94 |  | 5.000 | binary | Two-group slope | No |
| Zhang_2023 | OS | Underweight | 16.5 | Reported median | No | 6.862 | 1.566 | 30.064 | 8 |  | 5.000 | binary | Two-group slope | Not required for two-group reciprocal slope |
| Zhang_2023 | OS | Normal | 21.6 | Reported median | Yes | 1 |  |  | 30 |  | 5.000 | binary | Two-group slope | Not required for two-group reciprocal slope |
| McQuade (2018) | PFS | Normal | 21.75 | Midpoint | Yes | 1 |  |  | 68 | 56 |  | categorical | Hamling GLS | No |
| McQuade (2018) | PFS | Overweight | 27.5 | Midpoint | No | 0.7 | 0.480 | 1.030 | 88 | 67 |  | categorical | Hamling GLS | No |
| McQuade (2018) | PFS | Obese | 32.5 | Open-ended: adjacent width | No | 0.54 | 0.340 | 0.860 | 51 | 35 |  | categorical | Hamling GLS | No |
| McQuade (2018) | PFS | Normal | 21.75 | Midpoint | Yes | 1 |  |  | 102 | 59 |  | categorical | Hamling GLS | No |
| McQuade (2018) | PFS | Overweight | 27.5 | Midpoint | No | 0.78 | 0.520 | 1.170 | 109 | 51 |  | categorical | Hamling GLS | No |
| McQuade (2018) | PFS | Obese | 32.5 | Open-ended: adjacent width | No | 0.72 | 0.480 | 1.060 | 118 | 52 |  | categorical | Hamling GLS | No |
| Martini (2019) | PFS | Continuous BMI |  | Direct continuous estimate | NR | 0.96 | 0.920 | 1.000 | 82 |  | 1.000 | direct_continuous | Direct model | No |
| Cortellini (2019) | PFS | Normal | 21.75 | Midpoint | Yes | 1 |  |  | 452 | 322 |  | categorical | Hamling GLS | No |
| Cortellini (2019) | PFS | Overweight | 27.5 | Midpoint | No | 0.68 | 0.530 | 0.870 | 377 | 223 |  | categorical | Hamling GLS | No |
| Cortellini (2019) | PFS | Obese | 32.5 | Open-ended: adjacent width | No | 0.72 | 0.490 | 1.060 | 107 | 63 |  | categorical | Hamling GLS | No |
| Donnelly (2019) | PFS | Normal/underweight | 22.5 | Open-ended: adjacent width | Yes | 1 |  |  | 64 |  |  | categorical | Independent WLS | No |
| Donnelly (2019) | PFS | Overweight | 27.5 | Midpoint | No | 0.97 | 0.560 | 1.690 | 75 |  |  | categorical | Independent WLS | No |
| Donnelly (2019) | PFS | Obese | 32.5 | Open-ended: adjacent width | No | 1.16 | 0.700 | 1.940 | 61 |  |  | categorical | Independent WLS | No |
| Donnelly (2019) | PFS | Normal/underweight | 22.5 | Open-ended: adjacent width | Yes | 1 |  |  | 49 |  |  | categorical | Independent WLS | No |
| Donnelly (2019) | PFS | Overweight | 27.5 | Midpoint | No | 2.34 | 1.050 | 5.200 | 55 |  |  | categorical | Independent WLS | No |
| Donnelly (2019) | PFS | Obese | 32.5 | Open-ended: adjacent width | No | 2.46 | 1.030 | 5.890 | 41 |  |  | categorical | Independent WLS | No |
| Donnelly (2019) | PFS | Normal/underweight | 22.5 | Open-ended: adjacent width | Yes | 1 |  |  | 26 |  |  | categorical | Independent WLS | No |
| Donnelly (2019) | PFS | Overweight | 27.5 | Midpoint | No | 0.5 | 0.150 | 1.710 | 35 |  |  | categorical | Independent WLS | No |
| Donnelly (2019) | PFS | Obese | 32.5 | Open-ended: adjacent width | No | 0.18 | 0.050 | 0.740 | 17 |  |  | categorical | Independent WLS | No |
| Naik (2019) | PFS | Normal | 22.6 | Reported mean | Yes | 1 |  |  | 35 |  |  | categorical | Independent WLS | No |
| Naik (2019) | PFS | Overweight/Class I obese | 29.1 | Reported mean | No | 0.43 | 0.190 | 0.950 | 86 |  |  | categorical | Independent WLS | No |
| Naik (2019) | PFS | Class II/III obese | 40.1 | Reported mean | No | 1 | 0.340 | 2.940 | 15 |  |  | categorical | Independent WLS | No |
| Cortellini (2020) | PFS | Underweight | 15.25 | Open-ended: adjacent width | No | 1.46 | 0.980 | 2.200 | 35 |  |  | categorical | Independent WLS | Yes |
| Cortellini (2020) | PFS | Normal | 21.75 | Midpoint | Yes | 1 |  |  | 467 |  |  | categorical | Independent WLS | Yes |
| Cortellini (2020) | PFS | Overweight | 27.5 | Midpoint | No | 1.04 | 0.850 | 1.260 | 232 |  |  | categorical | Independent WLS | Yes |
| Cortellini (2020) | PFS | Obese | 32.5 | Open-ended: adjacent width | No | 0.61 | 0.450 | 0.820 | 106 |  |  | categorical | Independent WLS | Yes |
| Rutkowski (2020) | PFS | Continuous BMI |  | Direct continuous estimate | NR | 1 | 0.980 | 1.030 | 435 | 322 | 1.000 | direct_continuous | Direct model | No |
| Bartl (2021) | PFS | Continuous BMI |  | Direct continuous estimate | NR | 0.2680965 | 0.118 | 0.613 | 36 |  | 5.000 | direct_continuous | Direct model; reciprocal | No |
| Vithayathil (2023) | PFS | BMI <25 | 21.9 | Reported mean | Yes | 1 |  |  | 97 |  |  | binary | Two-group slope | No |
| Vithayathil (2023) | PFS | BMI >=25 | 29.6 | Reported mean | No | 0.7 | 0.410 | 1.200 | 94 |  |  | binary | Two-group slope | No |
| Zhang (2023) | PFS | Underweight | 16.5 | Reported median | Yes | 1 |  |  | 8 |  |  | binary | Two-group slope; reciprocal | Yes |
| Zhang (2023) | PFS | Normal | 21.6 | Reported median | No | 0.1763047 | 0.042 | 0.733 | 30 |  |  | binary | Two-group slope; reciprocal | Yes |
| Alam (2024) | PFS | Continuous BMI |  | Direct continuous estimate | NR | 0.95 | 0.800 | 1.120 | 169 |  | 5.000 | direct_continuous | Direct model | No |

**Supplementary Code S1.**Overall survival-adjusted estimates

```
# Analysis: 01_OS_adjusted
# All analysis data are embedded. Running this file creates figures only.
rm(list = ls())
options(stringsAsFactors = FALSE, scipen = 999)
analysis_id <- "01_OS_adjusted"
script_arg <- grep('^--file=', commandArgs(trailingOnly = FALSE), value = TRUE)
script_file <- if (length(script_arg)) sub('^--file=', '', script_arg[1]) else NA_character_
if ((is.na(script_file) || !nzchar(script_file)) && interactive() && requireNamespace('rstudioapi', quietly = TRUE)) {
  p <- tryCatch(rstudioapi::getSourceEditorContext()$path, error = function(e) '')
  if (nzchar(p)) script_file <- p
}
script_dir <- if (!is.na(script_file) && nzchar(script_file)) dirname(normalizePath(script_file, winslash='/', mustWork=TRUE)) else normalizePath(getwd(), winslash='/', mustWork=TRUE)
output_dir <- file.path(script_dir, paste0(analysis_id, '_output'))
dir.create(output_dir, recursive=TRUE, showWarnings=FALSE)
setwd(output_dir)
.libPaths(unique(c(file.path(Sys.getenv('USERPROFILE'), 'R-library'), .libPaths())))
embedded_input <- structure(list(Study = c("McQuade (1) 2018", "McQuade (2) 2018",
"Qi 2019", "Martini 2019", "Cortellini 2019", "Giorgi 2019",
"Donnelly (1) 2019", "Donnelly (2) 2019", "Kichenadasse 2019",
"Magri 2019", "Wang 2019", "Naik 2019", "Cortellini 2020", "Rutkowski 2020",
"Minami 2020", "Ahmed 2021", "Bartl 2021", "Collet 2021", "Imai 2021",
"Akce 2021", "Esposito 2021", "Zhang 2021", "Ged 2022", "Imai 2022",
"Joshi 2022", "Wang 2022", "Jain 2023", "Vithayathil 2023", "Zhang 2023",
"McManus 2023", "Miyamoto 2023", "Antoun (1) 2023", "Antoun (2) 2023",
"Santoni 2023", "Alam 2024", "Gómez-Banoy 2024", "Nie (1) 2024",
"Nie (2) 2024", "Wang 2024", "Llerena 2024", "Yamamoto 2024",
"Mastrolonardo 2025"), Effect = c(0.54, 0.72, 0.496, 0.306, 0.5,
0.633, 1.22, 1.04, 0.69, 1.19, 0.594, 0.42, 0.7, 1.17, 0.55,
0.96, 0.13, 0.75, 0.5, 0.44, 0.87, 0.62, 0.75, 0.56, 0.984, 0.56,
0.83, 0.83, 0.947, 0.984, 1.08, 0.85, 1.54, 0.68, 0.94, 0.64,
0.91, 0.98, 0.22, 0.62, 0.596, 0.7), LowerCI = c(0.34, 0.48,
0.26, 0.135, 0.32, 0.439, 0.75, 0.48, 0.54, 0.93, 0.35, 0.1,
0.49, 0.82, 0.24, 0.65, 0.03, 0.51, 0.3, 0.18, 0.55, 0.44, 0.5,
0.34, 0.914, 0.07, 0.49, 0.42, 0.089, 0.498, 0.63, 0.47, 0.73,
0.48, 0.57, 0.48, 0.76, 0.51, 0.11, 0.44, 0.331, 0.67), UpperCI = c(0.86,
1.06, 0.945, 0.694, 0.79, 0.917, 2, 2.24, 0.87, 1.51, 0.99, 1.77,
0.99, 1.68, 1.27, 1.42, 0.62, 1.1, 0.83, 1.09, 1.36, 0.87, 1.12,
0.91, 1.061, 4.26, 1.42, 1.64, 10.047, 1.941, 1.82, 1.56, 3.28,
0.95, 1.56, 0.87, 1.09, 1.86, 0.45, 0.86, 1.074, 0.73)), row.names = c(NA,
-42L), class = "data.frame")


reml_meta <- function (yi, sei)
{
    keep <- is.finite(yi) & is.finite(sei) & sei > 0
    yi <- yi[keep]
    sei <- sei[keep]
    vi <- sei^2
    k <- length(yi)
    if (!k)
        stop("No valid estimates.")
    if (k == 1L) {
        z <- qnorm(0.975)
        return(list(k = 1L, mu = yi, se_mu = sei, ci_low = yi - z * sei, ci_high = yi + z * sei, p = 2 * pnorm(abs(yi/sei), lower.tail = FALSE), tau2 = NA_real_, Q = NA_real_, df_Q = 0L, Q_p = NA_real_, I2 = NA_real_, weights = 100))
    }
    wf <- 1/vi
    muf <- sum(wf * yi)/sum(wf)
    Q <- sum(wf * (yi - muf)^2)
    dfQ <- k - 1
    I2 <- if (Q > 0)
        max(0, (Q - dfQ)/Q) * 100
    else 0
    obj <- function(tau2) {
        w <- 1/(vi + tau2)
        mu <- sum(w * yi)/sum(w)
        0.5 * (sum(log(vi + tau2)) + log(sum(w)) + sum(w * (yi - mu)^2))
    }
    upper <- max(1, var(yi) * 10, max(vi) * 10)
    opt <- optimize(obj, c(0, upper), tol = 1e-12)
    tau2 <- if (opt$minimum < 1e-10)
        0
    else opt$minimum
    w <- 1/(vi + tau2)
    mu <- sum(w * yi)/sum(w)
    se <- sqrt(1/sum(w))
    ci <- mu + c(-1, 1) * qnorm(0.975) * se
    list(k = k, mu = mu, se_mu = se, ci_low = ci[1], ci_high = ci[2], p = 2 * pnorm(abs(mu/se), lower.tail = FALSE), tau2 = tau2, Q = Q, df_Q = dfQ, Q_p = pchisq(Q, dfQ, lower.tail = FALSE), I2 = I2, weights = w/sum(w) * 100)
}

prepare_effects <- function (d, effect_col = "Effect")
{
    d[[effect_col]] <- as.numeric(d[[effect_col]])
    d$LowerCI <- as.numeric(d$LowerCI)
    d$UpperCI <- as.numeric(d$UpperCI)
    ok <- is.finite(d[[effect_col]]) & is.finite(d$LowerCI) & is.finite(d$UpperCI) & d[[effect_col]] > 0 & d$LowerCI > 0 & d$UpperCI > d$LowerCI
    d <- d[ok, , drop = FALSE]
    d$Effect <- d[[effect_col]]
    d$yi <- log(d$Effect)
    d$sei <- (log(d$UpperCI) - log(d$LowerCI))/(2 * qnorm(0.975))
    d
}

fmt_p <- function (p)
if (!is.finite(p)) "NR" else if (p < 1e-04) "< 0.0001" else paste0("= ", formatC(p, format = "f", digits = 4))

leave_one_out <- function (d)
{
    if (nrow(d) < 3)
        return(data.frame())
    do.call(rbind, lapply(seq_len(nrow(d)), function(i) {
        m <- reml_meta(d$yi[-i], d$sei[-i])
        data.frame(Study = d$Study[i], Effect = exp(m$mu), LowerCI = exp(m$ci_low), UpperCI = exp(m$ci_high))
    }))
}

endpoint <- "OS"
adjustment <- "Adjusted"
d <- prepare_effects(as.data.frame(embedded_input))
m <- reml_meta(d$yi,d$sei)
forest_highlow <- function (d, m, endpoint, adjustment, file)
{
    k <- nrow(d)
    h <- max(8.5, 3.4 + 0.255 * k)
    jpeg(file, width = 11, height = h, units = "in", res = 300, quality = 100)
    on.exit(dev.off(), add = TRUE)
    par(mar = c(4.8, 0.7, 1.4, 0.7), family = "sans", xpd = NA)
    plot.new()
    plot.window(xlim = c(0, 1), ylim = c(-2.3, k + 3.2))
    xs <- 0.02
    xln <- 0.315
    xse <- 0.415
    pl <- 0.515
    pr <- 0.735
    xhr <- 0.775
    xci <- 0.86
    xw <- 0.98
    amin <- if (min(c(d$Effect, exp(m$mu))) < 0.1)
        0.01
    else 0.1
    amax <- if (max(c(d$Effect, d$UpperCI, exp(m$ci_high))) > 20)
        100
    else 10
    ticks <- if (amin == 0.01)
        c(0.01, 0.1, 0.5, 1, 2, 10)
    else c(0.1, 0.5, 1, 2, 10)
    if (amax == 100)
        ticks <- c(ticks, 100)
    map <- function(v) pl + (log(v) - log(amin))/(log(amax) - log(amin)) * (pr - pl)
    clamp <- function(v) pmin(pr, pmax(pl, map(v)))
    y <- rev(seq_len(k)) + 1
    fhr <- function(v) if (v < 0.1)
        formatC(v, format = "f", digits = 3)
    else formatC(v, format = "f", digits = 2)
    text(xs, k + 2.45, "Study", adj = 0, font = 2, cex = 0.86)
    text(xln, k + 2.45, "lnHR", adj = 1, font = 2, cex = 0.86)
    text(xse, k + 2.45, "SE(lnHR)", adj = 1, font = 2, cex = 0.86)
    text((pl + pr)/2, k + 2.45, "Hazard Ratio", font = 2, cex = 0.86)
    text(xhr, k + 2.45, "HR", adj = 1, font = 2, cex = 0.86)
    text(xci, k + 2.45, "95% CI", font = 2, cex = 0.86)
    text(xw, k + 2.45, "Weight", adj = 1, font = 2, cex = 0.86)
    segments(map(1), -0.1, map(1), k + 1.7, lwd = 0.8)
    segments(clamp(exp(m$mu)), -0.1, clamp(exp(m$mu)), k + 1.7, lty = 3, col = "#444444", lwd = 0.9)
    for (i in seq_len(k)) {
        segments(clamp(d$LowerCI[i]), y[i], clamp(d$UpperCI[i]), y[i], lwd = 0.8)
        if (d$LowerCI[i] < amin)
            arrows(pl + 0.012, y[i], pl, y[i], length = 0.055, angle = 25)
        if (d$UpperCI[i] > amax)
            arrows(pr - 0.012, y[i], pr, y[i], length = 0.055, angle = 25)
        cs <- 0.45 + 1.5 * sqrt(m$weights[i]/max(m$weights))
        points(clamp(d$Effect[i]), y[i], pch = 15, cex = cs, col = "#005AA8")
        points(clamp(d$Effect[i]), y[i], pch = 3, cex = 0.45, lwd = 0.7, col = "white")
        text(xs, y[i], d$Study[i], adj = 0, cex = 0.7)
        text(xln, y[i], formatC(d$yi[i], format = "f", digits = 4), adj = 1, cex = 0.68)
        text(xse, y[i], formatC(d$sei[i], format = "f", digits = 4), adj = 1, cex = 0.68)
        text(xhr, y[i], fhr(d$Effect[i]), adj = 1, cex = 0.7)
        text(xci, y[i], paste0("[", fhr(d$LowerCI[i]), "; ", fhr(d$UpperCI[i]), "]"), cex = 0.7)
        text(xw, y[i], sprintf("%.1f%%", m$weights[i]), adj = 1, cex = 0.7)
    }
    py <- 0.55
    polygon(c(clamp(exp(m$ci_low)), clamp(exp(m$mu)), clamp(exp(m$ci_high)), clamp(exp(m$mu))), c(py, py + 0.24, py, py - 0.24), col = "#FF1A12", border = "black", lwd = 0.8)
    text(xs, py, "Random effects model", adj = 0, font = 2, cex = 0.78)
    text(xhr, py, sprintf("%.2f", exp(m$mu)), adj = 1, font = 2, cex = 0.76)
    text(xci, py, sprintf("[%.2f; %.2f]", exp(m$ci_low), exp(m$ci_high)), font = 2, cex = 0.76)
    text(xw, py, "100.0%", adj = 1, font = 2, cex = 0.76)
    ay <- -0.25
    segments(pl, ay, pr, ay)
    for (t in ticks) {
        segments(map(t), ay, map(t), ay - 0.18)
        text(map(t), ay - 0.38, format(t, trim = TRUE), cex = 0.68)
    }
    text((pl + map(1))/2, ay - 1, "High BMI", font = 2, cex = 0.66)
    text((map(1) + pr)/2, ay - 1, "Low BMI", font = 2, cex = 0.66)
    text(xs, -1.92, sprintf("Heterogeneity: I² = %.1f%%, τ² = %.4f, Q = %.2f (df = %d), p %s", m$I2, m$tau2, m$Q, m$df_Q, fmt_p(m$Q_p)), adj = 0, cex = 0.69)
    mtext(sprintf("High BMI vs Low BMI — %s — %s estimates", endpoint, adjustment), side = 3, line = 0.1, cex = 0.76, font = 2)
}
sensitivity_highlow <- function (loo, endpoint, adjustment, file)
{
    k <- nrow(loo)
    jpeg(file, width = 10, height = max(7.5, 3.6 + 0.25 * k), units = "in", res = 300, quality = 100)
    on.exit(dev.off(), add = TRUE)
    par(mar = c(4.8, 0.8, 2.2, 0.8), family = "sans", xpd = NA)
    plot.new()
    plot.window(xlim = c(0, 1), ylim = c(-1.7, k + 2.5))
    xs <- 0.03
    pl <- 0.42
    pr <- 0.7
    xhr <- 0.76
    xci <- 0.89
    amin <- 10^floor(log10(min(0.5, min(loo$LowerCI) * 0.9)))
    amax <- 10^ceiling(log10(max(2, max(loo$UpperCI) * 1.1)))
    ticks <- c(0.01, 0.05, 0.1, 0.2, 0.5, 1, 2, 5, 10, 100)
    ticks <- ticks[ticks >= amin & ticks <= amax]
    map <- function(v) pl + (log(v) - log(amin))/(log(amax) - log(amin)) * (pr - pl)
    y <- rev(seq_len(k)) + 0.6
    text(xs, k + 1.7, "Omitted study", adj = 0, font = 2, cex = 0.83)
    text((pl + pr)/2, k + 1.7, "Pooled Hazard Ratio", font = 2, cex = 0.83)
    text(xhr, k + 1.7, "HR", adj = 1, font = 2, cex = 0.83)
    text(xci, k + 1.7, "95% CI", font = 2, cex = 0.83)
    segments(map(1), -0.1, map(1), k + 1.3, lwd = 0.8)
    for (i in seq_len(k)) {
        segments(map(loo$LowerCI[i]), y[i], map(loo$UpperCI[i]), y[i], lwd = 0.85)
        points(map(loo$Effect[i]), y[i], pch = 15, cex = 1.15, col = "#005AA8")
        text(xs, y[i], loo$Study[i], adj = 0, cex = 0.69)
        text(xhr, y[i], sprintf("%.2f", loo$Effect[i]), adj = 1, cex = 0.7)
        text(xci, y[i], sprintf("[%.2f; %.2f]", loo$LowerCI[i], loo$UpperCI[i]), cex = 0.7)
    }
    ay <- -0.15
    segments(pl, ay, pr, ay)
    for (t in ticks) {
        segments(map(t), ay, map(t), ay - 0.15)
        text(map(t), ay - 0.38, format(t, trim = TRUE), cex = 0.67)
    }
    text((pl + map(1))/2, ay - 1, "High BMI", font = 2, cex = 0.65)
    text((map(1) + pr)/2, ay - 1, "Low BMI", font = 2, cex = 0.65)
    mtext(sprintf("High BMI vs Low BMI — leave-one-out sensitivity analysis — %s, %s estimates", endpoint, tolower(adjustment)), side = 3, line = 0.5, font = 2, cex = 0.86)
}
funnel_plot <- function (d, m, title, xlab, file, note = TRUE)
{
    jpeg(file, width = 8, height = 7, units = "in", res = 300, quality = 100)
    on.exit(dev.off(), add = TRUE)
    par(mar = c(5, 5, 3.3, 1.2), family = "sans", las = 1)
    ym <- max(d$sei) * 1.08
    xc <- c(d$yi, m$mu - 1.96 * ym, m$mu + 1.96 * ym)
    pad <- diff(range(xc)) * 0.08
    plot(d$yi, d$sei, xlim = range(xc) + c(-pad, pad), ylim = c(ym, 0), pch = 21, bg = if (grepl("hazard", tolower(xlab)))
        "#2F80C0"
    else "#D0D0D0", col = if (grepl("hazard", tolower(xlab)))
        "#004C99"
    else "black", cex = 1.15, xlab = xlab, ylab = "Standard error", main = title, bty = "l")
    s <- seq(0, ym, length.out = 200)
    lines(m$mu - 1.96 * s, s, lty = 2, col = "#707070")
    lines(m$mu + 1.96 * s, s, lty = 2, col = "#707070")
    abline(v = m$mu, col = if (grepl("hazard", tolower(xlab)))
        "#D73027"
    else "#333333", lty = if (grepl("hazard", tolower(xlab)))
        1
    else 3, lwd = 1.2)
    if (note) {
        if (nrow(d) < 10)
            mtext(sprintf("Exploratory only; Egger test not performed (k = %d < 10).", nrow(d)), side = 1, line = 3.7, cex = 0.78)
    }
}
forest_highlow(d,m,endpoint,adjustment,'forest.jpg')
loo<-leave_one_out(d);sensitivity_highlow(loo,endpoint,adjustment,'sensitivity_leave_one_out.jpg')
funnel_plot(d,m,paste0('Funnel plot — ',endpoint,', ',tolower(adjustment),' estimates'),'Log hazard ratio','funnel.jpg',TRUE)
```

**Supplementary Code S2.**Overall survival-unadjusted estimates

```
# Analysis: 02_OS_unadjusted
# All analysis data are embedded. Running this file creates figures only.
rm(list = ls())
options(stringsAsFactors = FALSE, scipen = 999)
analysis_id <- "02_OS_unadjusted"
script_arg <- grep('^--file=', commandArgs(trailingOnly = FALSE), value = TRUE)
script_file <- if (length(script_arg)) sub('^--file=', '', script_arg[1]) else NA_character_
if ((is.na(script_file) || !nzchar(script_file)) && interactive() && requireNamespace('rstudioapi', quietly = TRUE)) {
  p <- tryCatch(rstudioapi::getSourceEditorContext()$path, error = function(e) '')
  if (nzchar(p)) script_file <- p
}
script_dir <- if (!is.na(script_file) && nzchar(script_file)) dirname(normalizePath(script_file, winslash='/', mustWork=TRUE)) else normalizePath(getwd(), winslash='/', mustWork=TRUE)
output_dir <- file.path(script_dir, paste0(analysis_id, '_output'))
dir.create(output_dir, recursive=TRUE, showWarnings=FALSE)
setwd(output_dir)
.libPaths(unique(c(file.path(Sys.getenv('USERPROFILE'), 'R-library'), .libPaths())))
embedded_input <- structure(list(Study = c("McQuade (1) 2018", "McQuade (2) 2018",
"Dumenil 2018", "Qi 2019", "Cortellini 2019", "Giorgi 2019",
"Donnelly (1) 2019", "Donnelly (2) 2019", "Katayama 2019", "Kichenadasse 2019",
"Labadie 2019", "Magri 2019", "Naik 2019", "Ichihara (1) 2020",
"Ichihara (2) 2020", "Ahmed 2021", "Bartl 2021", "Collet 2021",
"Imai 2021", "Esposito 2021", "Ged 2022", "Imai 2022", "Joshi 2022",
"Wang 2022", "Tateishi 2022", "Wasamoto 2023", "Incorvaia 2023",
"Vithayathil 2023", "Wu 2023", "Zhang 2023", "McManus 2023",
"Miyamoto 2023", "Antoun (1) 2023", "Antoun (2) 2023", "Santoni 2023",
"Bernard 2024", "Gómez-Banoy 2024", "Nie (1) 2024", "Nie (2) 2024",
"Wang 2024", "?en 2024", "Yamamoto 2024", "Demir 2025", "Krej?í 2025",
"Anami 2025"), Effect = c(0.64, 0.7, 0.775, 0.496, 0.37, 0.667,
1.07, 0.88, 0.92, 0.64, 0.28, 1.01, 0.88, 0.67, 0.28, 0.77, 0.53,
0.63, 0.51, 0.8, 0.66, 0.49, 0.952, 1.09, 1.03, 0.66, 0.61, 1.29,
0.62, 0.438, 0.916, 1.01, 0.77, 0.69, 0.52, 0.71, 0.65, 0.91,
1.02, 0.39, 1.06, 0.486, 1.01, 0.66, 0.63), LowerCI = c(0.42,
0.48, 0.328, 0.261, 0.27, 0.465, 0.63, 0.42, 0.26, 0.51, 0.09,
0.94, 0.41, 0.32, 0.085, 0.57, 0.3, 0.44, 0.3, 0.53, 0.45, 0.3,
0.911, 0.38, 0.68, 0.38, 0.11, 0.7, 0.32, 0.056, 0.489, 0.6,
0.52, 0.43, 0.38, 0.26, 0.49, 0.76, 0.59, 0.21, 0.69, 0.283,
0.64, 0.42, 0.41), UpperCI = c(0.97, 1.01, 1.818, 0.943, 0.51,
0.952, 1.8, 1.85, 3.25, 0.81, 0.91, 1.09, 1.91, 1.4, 0.89, 1.04,
0.93, 0.92, 0.83, 1.21, 0.97, 0.78, 0.996, 3.13, 1.54, 1.14,
3.37, 2.39, 1.2, 3.401, 1.718, 1.69, 1.16, 1.11, 0.73, 1.98,
0.86, 1.09, 1.75, 0.7, 1.64, 0.836, 1.56, 1.05, 0.98)), row.names = c(NA,
-45L), class = "data.frame")


reml_meta <- function (yi, sei)
{
    keep <- is.finite(yi) & is.finite(sei) & sei > 0
    yi <- yi[keep]
    sei <- sei[keep]
    vi <- sei^2
    k <- length(yi)
    if (!k)
        stop("No valid estimates.")
    if (k == 1L) {
        z <- qnorm(0.975)
        return(list(k = 1L, mu = yi, se_mu = sei, ci_low = yi - z * sei, ci_high = yi + z * sei, p = 2 * pnorm(abs(yi/sei), lower.tail = FALSE), tau2 = NA_real_, Q = NA_real_, df_Q = 0L, Q_p = NA_real_, I2 = NA_real_, weights = 100))
    }
    wf <- 1/vi
    muf <- sum(wf * yi)/sum(wf)
    Q <- sum(wf * (yi - muf)^2)
    dfQ <- k - 1
    I2 <- if (Q > 0)
        max(0, (Q - dfQ)/Q) * 100
    else 0
    obj <- function(tau2) {
        w <- 1/(vi + tau2)
        mu <- sum(w * yi)/sum(w)
        0.5 * (sum(log(vi + tau2)) + log(sum(w)) + sum(w * (yi - mu)^2))
    }
    upper <- max(1, var(yi) * 10, max(vi) * 10)
    opt <- optimize(obj, c(0, upper), tol = 1e-12)
    tau2 <- if (opt$minimum < 1e-10)
        0
    else opt$minimum
    w <- 1/(vi + tau2)
    mu <- sum(w * yi)/sum(w)
    se <- sqrt(1/sum(w))
    ci <- mu + c(-1, 1) * qnorm(0.975) * se
    list(k = k, mu = mu, se_mu = se, ci_low = ci[1], ci_high = ci[2], p = 2 * pnorm(abs(mu/se), lower.tail = FALSE), tau2 = tau2, Q = Q, df_Q = dfQ, Q_p = pchisq(Q, dfQ, lower.tail = FALSE), I2 = I2, weights = w/sum(w) * 100)
}

prepare_effects <- function (d, effect_col = "Effect")
{
    d[[effect_col]] <- as.numeric(d[[effect_col]])
    d$LowerCI <- as.numeric(d$LowerCI)
    d$UpperCI <- as.numeric(d$UpperCI)
    ok <- is.finite(d[[effect_col]]) & is.finite(d$LowerCI) & is.finite(d$UpperCI) & d[[effect_col]] > 0 & d$LowerCI > 0 & d$UpperCI > d$LowerCI
    d <- d[ok, , drop = FALSE]
    d$Effect <- d[[effect_col]]
    d$yi <- log(d$Effect)
    d$sei <- (log(d$UpperCI) - log(d$LowerCI))/(2 * qnorm(0.975))
    d
}

fmt_p <- function (p)
if (!is.finite(p)) "NR" else if (p < 1e-04) "< 0.0001" else paste0("= ", formatC(p, format = "f", digits = 4))

leave_one_out <- function (d)
{
    if (nrow(d) < 3)
        return(data.frame())
    do.call(rbind, lapply(seq_len(nrow(d)), function(i) {
        m <- reml_meta(d$yi[-i], d$sei[-i])
        data.frame(Study = d$Study[i], Effect = exp(m$mu), LowerCI = exp(m$ci_low), UpperCI = exp(m$ci_high))
    }))
}

endpoint <- "OS"
adjustment <- "Unadjusted"
d <- prepare_effects(as.data.frame(embedded_input))
m <- reml_meta(d$yi,d$sei)
forest_highlow <- function (d, m, endpoint, adjustment, file)
{
    k <- nrow(d)
    h <- max(8.5, 3.4 + 0.255 * k)
    jpeg(file, width = 11, height = h, units = "in", res = 300, quality = 100)
    on.exit(dev.off(), add = TRUE)
    par(mar = c(4.8, 0.7, 1.4, 0.7), family = "sans", xpd = NA)
    plot.new()
    plot.window(xlim = c(0, 1), ylim = c(-2.3, k + 3.2))
    xs <- 0.02
    xln <- 0.315
    xse <- 0.415
    pl <- 0.515
    pr <- 0.735
    xhr <- 0.775
    xci <- 0.86
    xw <- 0.98
    amin <- if (min(c(d$Effect, exp(m$mu))) < 0.1)
        0.01
    else 0.1
    amax <- if (max(c(d$Effect, d$UpperCI, exp(m$ci_high))) > 20)
        100
    else 10
    ticks <- if (amin == 0.01)
        c(0.01, 0.1, 0.5, 1, 2, 10)
    else c(0.1, 0.5, 1, 2, 10)
    if (amax == 100)
        ticks <- c(ticks, 100)
    map <- function(v) pl + (log(v) - log(amin))/(log(amax) - log(amin)) * (pr - pl)
    clamp <- function(v) pmin(pr, pmax(pl, map(v)))
    y <- rev(seq_len(k)) + 1
    fhr <- function(v) if (v < 0.1)
        formatC(v, format = "f", digits = 3)
    else formatC(v, format = "f", digits = 2)
    text(xs, k + 2.45, "Study", adj = 0, font = 2, cex = 0.86)
    text(xln, k + 2.45, "lnHR", adj = 1, font = 2, cex = 0.86)
    text(xse, k + 2.45, "SE(lnHR)", adj = 1, font = 2, cex = 0.86)
    text((pl + pr)/2, k + 2.45, "Hazard Ratio", font = 2, cex = 0.86)
    text(xhr, k + 2.45, "HR", adj = 1, font = 2, cex = 0.86)
    text(xci, k + 2.45, "95% CI", font = 2, cex = 0.86)
    text(xw, k + 2.45, "Weight", adj = 1, font = 2, cex = 0.86)
    segments(map(1), -0.1, map(1), k + 1.7, lwd = 0.8)
    segments(clamp(exp(m$mu)), -0.1, clamp(exp(m$mu)), k + 1.7, lty = 3, col = "#444444", lwd = 0.9)
    for (i in seq_len(k)) {
        segments(clamp(d$LowerCI[i]), y[i], clamp(d$UpperCI[i]), y[i], lwd = 0.8)
        if (d$LowerCI[i] < amin)
            arrows(pl + 0.012, y[i], pl, y[i], length = 0.055, angle = 25)
        if (d$UpperCI[i] > amax)
            arrows(pr - 0.012, y[i], pr, y[i], length = 0.055, angle = 25)
        cs <- 0.45 + 1.5 * sqrt(m$weights[i]/max(m$weights))
        points(clamp(d$Effect[i]), y[i], pch = 15, cex = cs, col = "#005AA8")
        points(clamp(d$Effect[i]), y[i], pch = 3, cex = 0.45, lwd = 0.7, col = "white")
        text(xs, y[i], d$Study[i], adj = 0, cex = 0.7)
        text(xln, y[i], formatC(d$yi[i], format = "f", digits = 4), adj = 1, cex = 0.68)
        text(xse, y[i], formatC(d$sei[i], format = "f", digits = 4), adj = 1, cex = 0.68)
        text(xhr, y[i], fhr(d$Effect[i]), adj = 1, cex = 0.7)
        text(xci, y[i], paste0("[", fhr(d$LowerCI[i]), "; ", fhr(d$UpperCI[i]), "]"), cex = 0.7)
        text(xw, y[i], sprintf("%.1f%%", m$weights[i]), adj = 1, cex = 0.7)
    }
    py <- 0.55
    polygon(c(clamp(exp(m$ci_low)), clamp(exp(m$mu)), clamp(exp(m$ci_high)), clamp(exp(m$mu))), c(py, py + 0.24, py, py - 0.24), col = "#FF1A12", border = "black", lwd = 0.8)
    text(xs, py, "Random effects model", adj = 0, font = 2, cex = 0.78)
    text(xhr, py, sprintf("%.2f", exp(m$mu)), adj = 1, font = 2, cex = 0.76)
    text(xci, py, sprintf("[%.2f; %.2f]", exp(m$ci_low), exp(m$ci_high)), font = 2, cex = 0.76)
    text(xw, py, "100.0%", adj = 1, font = 2, cex = 0.76)
    ay <- -0.25
    segments(pl, ay, pr, ay)
    for (t in ticks) {
        segments(map(t), ay, map(t), ay - 0.18)
        text(map(t), ay - 0.38, format(t, trim = TRUE), cex = 0.68)
    }
    text((pl + map(1))/2, ay - 1, "High BMI", font = 2, cex = 0.66)
    text((map(1) + pr)/2, ay - 1, "Low BMI", font = 2, cex = 0.66)
    text(xs, -1.92, sprintf("Heterogeneity: I² = %.1f%%, τ² = %.4f, Q = %.2f (df = %d), p %s", m$I2, m$tau2, m$Q, m$df_Q, fmt_p(m$Q_p)), adj = 0, cex = 0.69)
    mtext(sprintf("High BMI vs Low BMI — %s — %s estimates", endpoint, adjustment), side = 3, line = 0.1, cex = 0.76, font = 2)
}
sensitivity_highlow <- function (loo, endpoint, adjustment, file)
{
    k <- nrow(loo)
    jpeg(file, width = 10, height = max(7.5, 3.6 + 0.25 * k), units = "in", res = 300, quality = 100)
    on.exit(dev.off(), add = TRUE)
    par(mar = c(4.8, 0.8, 2.2, 0.8), family = "sans", xpd = NA)
    plot.new()
    plot.window(xlim = c(0, 1), ylim = c(-1.7, k + 2.5))
    xs <- 0.03
    pl <- 0.42
    pr <- 0.7
    xhr <- 0.76
    xci <- 0.89
    amin <- 10^floor(log10(min(0.5, min(loo$LowerCI) * 0.9)))
    amax <- 10^ceiling(log10(max(2, max(loo$UpperCI) * 1.1)))
    ticks <- c(0.01, 0.05, 0.1, 0.2, 0.5, 1, 2, 5, 10, 100)
    ticks <- ticks[ticks >= amin & ticks <= amax]
    map <- function(v) pl + (log(v) - log(amin))/(log(amax) - log(amin)) * (pr - pl)
    y <- rev(seq_len(k)) + 0.6
    text(xs, k + 1.7, "Omitted study", adj = 0, font = 2, cex = 0.83)
    text((pl + pr)/2, k + 1.7, "Pooled Hazard Ratio", font = 2, cex = 0.83)
    text(xhr, k + 1.7, "HR", adj = 1, font = 2, cex = 0.83)
    text(xci, k + 1.7, "95% CI", font = 2, cex = 0.83)
    segments(map(1), -0.1, map(1), k + 1.3, lwd = 0.8)
    for (i in seq_len(k)) {
        segments(map(loo$LowerCI[i]), y[i], map(loo$UpperCI[i]), y[i], lwd = 0.85)
        points(map(loo$Effect[i]), y[i], pch = 15, cex = 1.15, col = "#005AA8")
        text(xs, y[i], loo$Study[i], adj = 0, cex = 0.69)
        text(xhr, y[i], sprintf("%.2f", loo$Effect[i]), adj = 1, cex = 0.7)
        text(xci, y[i], sprintf("[%.2f; %.2f]", loo$LowerCI[i], loo$UpperCI[i]), cex = 0.7)
    }
    ay <- -0.15
    segments(pl, ay, pr, ay)
    for (t in ticks) {
        segments(map(t), ay, map(t), ay - 0.15)
        text(map(t), ay - 0.38, format(t, trim = TRUE), cex = 0.67)
    }
    text((pl + map(1))/2, ay - 1, "High BMI", font = 2, cex = 0.65)
    text((map(1) + pr)/2, ay - 1, "Low BMI", font = 2, cex = 0.65)
    mtext(sprintf("High BMI vs Low BMI — leave-one-out sensitivity analysis — %s, %s estimates", endpoint, tolower(adjustment)), side = 3, line = 0.5, font = 2, cex = 0.86)
}
funnel_plot <- function (d, m, title, xlab, file, note = TRUE)
{
    jpeg(file, width = 8, height = 7, units = "in", res = 300, quality = 100)
    on.exit(dev.off(), add = TRUE)
    par(mar = c(5, 5, 3.3, 1.2), family = "sans", las = 1)
    ym <- max(d$sei) * 1.08
    xc <- c(d$yi, m$mu - 1.96 * ym, m$mu + 1.96 * ym)
    pad <- diff(range(xc)) * 0.08
    plot(d$yi, d$sei, xlim = range(xc) + c(-pad, pad), ylim = c(ym, 0), pch = 21, bg = if (grepl("hazard", tolower(xlab)))
        "#2F80C0"
    else "#D0D0D0", col = if (grepl("hazard", tolower(xlab)))
        "#004C99"
    else "black", cex = 1.15, xlab = xlab, ylab = "Standard error", main = title, bty = "l")
    s <- seq(0, ym, length.out = 200)
    lines(m$mu - 1.96 * s, s, lty = 2, col = "#707070")
    lines(m$mu + 1.96 * s, s, lty = 2, col = "#707070")
    abline(v = m$mu, col = if (grepl("hazard", tolower(xlab)))
        "#D73027"
    else "#333333", lty = if (grepl("hazard", tolower(xlab)))
        1
    else 3, lwd = 1.2)
    if (note) {
        if (nrow(d) < 10)
            mtext(sprintf("Exploratory only; Egger test not performed (k = %d < 10).", nrow(d)), side = 1, line = 3.7, cex = 0.78)
    }
}
forest_highlow(d,m,endpoint,adjustment,'forest.jpg')
loo<-leave_one_out(d);sensitivity_highlow(loo,endpoint,adjustment,'sensitivity_leave_one_out.jpg')
funnel_plot(d,m,paste0('Funnel plot — ',endpoint,', ',tolower(adjustment),' estimates'),'Log hazard ratio','funnel.jpg',TRUE)
```

**Supplementary Code S3.**Progression-free survival-adjusted estimates

```
# Analysis: 03_PFS_adjusted
# All analysis data are embedded. Running this file creates figures only.
rm(list = ls())
options(stringsAsFactors = FALSE, scipen = 999)
analysis_id <- "03_PFS_adjusted"
script_arg <- grep('^--file=', commandArgs(trailingOnly = FALSE), value = TRUE)
script_file <- if (length(script_arg)) sub('^--file=', '', script_arg[1]) else NA_character_
if ((is.na(script_file) || !nzchar(script_file)) && interactive() && requireNamespace('rstudioapi', quietly = TRUE)) {
  p <- tryCatch(rstudioapi::getSourceEditorContext()$path, error = function(e) '')
  if (nzchar(p)) script_file <- p
}
script_dir <- if (!is.na(script_file) && nzchar(script_file)) dirname(normalizePath(script_file, winslash='/', mustWork=TRUE)) else normalizePath(getwd(), winslash='/', mustWork=TRUE)
output_dir <- file.path(script_dir, paste0(analysis_id, '_output'))
dir.create(output_dir, recursive=TRUE, showWarnings=FALSE)
setwd(output_dir)
.libPaths(unique(c(file.path(Sys.getenv('USERPROFILE'), 'R-library'), .libPaths())))
embedded_input <- structure(list(Study = c("McQuade (1) 2018", "McQuade (2) 2018",
"Martini 2019", "Cortellini 2019", "Donnelly (1) 2019", "Donnelly (2) 2019",
"Kichenadasse 2019", "Wang 2019", "Naik 2019", "Cortellini 2020",
"Rutkowski 2020", "Rogado 2020", "Minami 2020", "Ahmed 2021",
"Bartl 2021", "Akce 2021", "Esposito 2021", "Zhang 2021", "Chen 2022",
"Imai 2022", "Wang 2022", "Tateishi 2022", "Incorvaia 2023",
"Jain 2023", "Vithayathil 2023", "Zhang 2023", "McManus 2023",
"Miyamoto 2023", "Alam 2024", "Gómez-Banoy 2024", "Nie (1) 2024",
"Nie (2) 2024", "Tan 2024", "Wang 2024"), Effect = c(0.63, 0.85,
0.543, 0.72, 1.16, 2.46, 0.87, 0.61, 1, 0.61, 1.01, 0.58, 0.9,
0.87, 0.27, 1.04, 0.8, 0.76, 0.47, 0.78, 0.44, 1, 0.18, 0.7,
0.8, 0.863, 1.859, 1.18, 0.94, 0.54, 1.01, 1.14, 0.022, 0.32),
    LowerCI = c(0.41, 0.61, 0.278, 0.49, 0.7, 1.03, 0.72, 0.42,
    0.34, 0.45, 0.74, 0.38, 0.45, 0.59, 0.12, 0.56, 0.54, 0.55,
    0.22, 0.51, 0.17, 0.4, 0.03, 0.42, 0.41, 0.091, 1.156, 0.76,
    0.62, 0.42, 0.84, 0.68, 0.001, 0.17), UpperCI = c(0.95, 1.19,
    1.064, 1.06, 1.94, 5.89, 1.04, 0.89, 2.94, 0.82, 1.39, 0.89,
    1.82, 1.28, 0.61, 1.92, 1.19, 1.04, 0.98, 1.19, 1.13, 2.45,
    0.93, 1.14, 1.56, 8.156, 2.989, 1.82, 1.44, 0.71, 1.21, 1.92,
    0.326, 0.59)), row.names = c(NA, -34L), class = "data.frame")


reml_meta <- function (yi, sei)
{
    keep <- is.finite(yi) & is.finite(sei) & sei > 0
    yi <- yi[keep]
    sei <- sei[keep]
    vi <- sei^2
    k <- length(yi)
    if (!k)
        stop("No valid estimates.")
    if (k == 1L) {
        z <- qnorm(0.975)
        return(list(k = 1L, mu = yi, se_mu = sei, ci_low = yi - z * sei, ci_high = yi + z * sei, p = 2 * pnorm(abs(yi/sei), lower.tail = FALSE), tau2 = NA_real_, Q = NA_real_, df_Q = 0L, Q_p = NA_real_, I2 = NA_real_, weights = 100))
    }
    wf <- 1/vi
    muf <- sum(wf * yi)/sum(wf)
    Q <- sum(wf * (yi - muf)^2)
    dfQ <- k - 1
    I2 <- if (Q > 0)
        max(0, (Q - dfQ)/Q) * 100
    else 0
    obj <- function(tau2) {
        w <- 1/(vi + tau2)
        mu <- sum(w * yi)/sum(w)
        0.5 * (sum(log(vi + tau2)) + log(sum(w)) + sum(w * (yi - mu)^2))
    }
    upper <- max(1, var(yi) * 10, max(vi) * 10)
    opt <- optimize(obj, c(0, upper), tol = 1e-12)
    tau2 <- if (opt$minimum < 1e-10)
        0
    else opt$minimum
    w <- 1/(vi + tau2)
    mu <- sum(w * yi)/sum(w)
    se <- sqrt(1/sum(w))
    ci <- mu + c(-1, 1) * qnorm(0.975) * se
    list(k = k, mu = mu, se_mu = se, ci_low = ci[1], ci_high = ci[2], p = 2 * pnorm(abs(mu/se), lower.tail = FALSE), tau2 = tau2, Q = Q, df_Q = dfQ, Q_p = pchisq(Q, dfQ, lower.tail = FALSE), I2 = I2, weights = w/sum(w) * 100)
}

prepare_effects <- function (d, effect_col = "Effect")
{
    d[[effect_col]] <- as.numeric(d[[effect_col]])
    d$LowerCI <- as.numeric(d$LowerCI)
    d$UpperCI <- as.numeric(d$UpperCI)
    ok <- is.finite(d[[effect_col]]) & is.finite(d$LowerCI) & is.finite(d$UpperCI) & d[[effect_col]] > 0 & d$LowerCI > 0 & d$UpperCI > d$LowerCI
    d <- d[ok, , drop = FALSE]
    d$Effect <- d[[effect_col]]
    d$yi <- log(d$Effect)
    d$sei <- (log(d$UpperCI) - log(d$LowerCI))/(2 * qnorm(0.975))
    d
}

fmt_p <- function (p)
if (!is.finite(p)) "NR" else if (p < 1e-04) "< 0.0001" else paste0("= ", formatC(p, format = "f", digits = 4))

leave_one_out <- function (d)
{
    if (nrow(d) < 3)
        return(data.frame())
    do.call(rbind, lapply(seq_len(nrow(d)), function(i) {
        m <- reml_meta(d$yi[-i], d$sei[-i])
        data.frame(Study = d$Study[i], Effect = exp(m$mu), LowerCI = exp(m$ci_low), UpperCI = exp(m$ci_high))
    }))
}

endpoint <- "PFS"
adjustment <- "Adjusted"
d <- prepare_effects(as.data.frame(embedded_input))
m <- reml_meta(d$yi,d$sei)
forest_highlow <- function (d, m, endpoint, adjustment, file)
{
    k <- nrow(d)
    h <- max(8.5, 3.4 + 0.255 * k)
    jpeg(file, width = 11, height = h, units = "in", res = 300, quality = 100)
    on.exit(dev.off(), add = TRUE)
    par(mar = c(4.8, 0.7, 1.4, 0.7), family = "sans", xpd = NA)
    plot.new()
    plot.window(xlim = c(0, 1), ylim = c(-2.3, k + 3.2))
    xs <- 0.02
    xln <- 0.315
    xse <- 0.415
    pl <- 0.515
    pr <- 0.735
    xhr <- 0.775
    xci <- 0.86
    xw <- 0.98
    amin <- if (min(c(d$Effect, exp(m$mu))) < 0.1)
        0.01
    else 0.1
    amax <- if (max(c(d$Effect, d$UpperCI, exp(m$ci_high))) > 20)
        100
    else 10
    ticks <- if (amin == 0.01)
        c(0.01, 0.1, 0.5, 1, 2, 10)
    else c(0.1, 0.5, 1, 2, 10)
    if (amax == 100)
        ticks <- c(ticks, 100)
    map <- function(v) pl + (log(v) - log(amin))/(log(amax) - log(amin)) * (pr - pl)
    clamp <- function(v) pmin(pr, pmax(pl, map(v)))
    y <- rev(seq_len(k)) + 1
    fhr <- function(v) if (v < 0.1)
        formatC(v, format = "f", digits = 3)
    else formatC(v, format = "f", digits = 2)
    text(xs, k + 2.45, "Study", adj = 0, font = 2, cex = 0.86)
    text(xln, k + 2.45, "lnHR", adj = 1, font = 2, cex = 0.86)
    text(xse, k + 2.45, "SE(lnHR)", adj = 1, font = 2, cex = 0.86)
    text((pl + pr)/2, k + 2.45, "Hazard Ratio", font = 2, cex = 0.86)
    text(xhr, k + 2.45, "HR", adj = 1, font = 2, cex = 0.86)
    text(xci, k + 2.45, "95% CI", font = 2, cex = 0.86)
    text(xw, k + 2.45, "Weight", adj = 1, font = 2, cex = 0.86)
    segments(map(1), -0.1, map(1), k + 1.7, lwd = 0.8)
    segments(clamp(exp(m$mu)), -0.1, clamp(exp(m$mu)), k + 1.7, lty = 3, col = "#444444", lwd = 0.9)
    for (i in seq_len(k)) {
        segments(clamp(d$LowerCI[i]), y[i], clamp(d$UpperCI[i]), y[i], lwd = 0.8)
        if (d$LowerCI[i] < amin)
            arrows(pl + 0.012, y[i], pl, y[i], length = 0.055, angle = 25)
        if (d$UpperCI[i] > amax)
            arrows(pr - 0.012, y[i], pr, y[i], length = 0.055, angle = 25)
        cs <- 0.45 + 1.5 * sqrt(m$weights[i]/max(m$weights))
        points(clamp(d$Effect[i]), y[i], pch = 15, cex = cs, col = "#005AA8")
        points(clamp(d$Effect[i]), y[i], pch = 3, cex = 0.45, lwd = 0.7, col = "white")
        text(xs, y[i], d$Study[i], adj = 0, cex = 0.7)
        text(xln, y[i], formatC(d$yi[i], format = "f", digits = 4), adj = 1, cex = 0.68)
        text(xse, y[i], formatC(d$sei[i], format = "f", digits = 4), adj = 1, cex = 0.68)
        text(xhr, y[i], fhr(d$Effect[i]), adj = 1, cex = 0.7)
        text(xci, y[i], paste0("[", fhr(d$LowerCI[i]), "; ", fhr(d$UpperCI[i]), "]"), cex = 0.7)
        text(xw, y[i], sprintf("%.1f%%", m$weights[i]), adj = 1, cex = 0.7)
    }
    py <- 0.55
    polygon(c(clamp(exp(m$ci_low)), clamp(exp(m$mu)), clamp(exp(m$ci_high)), clamp(exp(m$mu))), c(py, py + 0.24, py, py - 0.24), col = "#FF1A12", border = "black", lwd = 0.8)
    text(xs, py, "Random effects model", adj = 0, font = 2, cex = 0.78)
    text(xhr, py, sprintf("%.2f", exp(m$mu)), adj = 1, font = 2, cex = 0.76)
    text(xci, py, sprintf("[%.2f; %.2f]", exp(m$ci_low), exp(m$ci_high)), font = 2, cex = 0.76)
    text(xw, py, "100.0%", adj = 1, font = 2, cex = 0.76)
    ay <- -0.25
    segments(pl, ay, pr, ay)
    for (t in ticks) {
        segments(map(t), ay, map(t), ay - 0.18)
        text(map(t), ay - 0.38, format(t, trim = TRUE), cex = 0.68)
    }
    text((pl + map(1))/2, ay - 1, "High BMI", font = 2, cex = 0.66)
    text((map(1) + pr)/2, ay - 1, "Low BMI", font = 2, cex = 0.66)
    text(xs, -1.92, sprintf("Heterogeneity: I² = %.1f%%, τ² = %.4f, Q = %.2f (df = %d), p %s", m$I2, m$tau2, m$Q, m$df_Q, fmt_p(m$Q_p)), adj = 0, cex = 0.69)
    mtext(sprintf("High BMI vs Low BMI — %s — %s estimates", endpoint, adjustment), side = 3, line = 0.1, cex = 0.76, font = 2)
}
sensitivity_highlow <- function (loo, endpoint, adjustment, file)
{
    k <- nrow(loo)
    jpeg(file, width = 10, height = max(7.5, 3.6 + 0.25 * k), units = "in", res = 300, quality = 100)
    on.exit(dev.off(), add = TRUE)
    par(mar = c(4.8, 0.8, 2.2, 0.8), family = "sans", xpd = NA)
    plot.new()
    plot.window(xlim = c(0, 1), ylim = c(-1.7, k + 2.5))
    xs <- 0.03
    pl <- 0.42
    pr <- 0.7
    xhr <- 0.76
    xci <- 0.89
    amin <- 10^floor(log10(min(0.5, min(loo$LowerCI) * 0.9)))
    amax <- 10^ceiling(log10(max(2, max(loo$UpperCI) * 1.1)))
    ticks <- c(0.01, 0.05, 0.1, 0.2, 0.5, 1, 2, 5, 10, 100)
    ticks <- ticks[ticks >= amin & ticks <= amax]
    map <- function(v) pl + (log(v) - log(amin))/(log(amax) - log(amin)) * (pr - pl)
    y <- rev(seq_len(k)) + 0.6
    text(xs, k + 1.7, "Omitted study", adj = 0, font = 2, cex = 0.83)
    text((pl + pr)/2, k + 1.7, "Pooled Hazard Ratio", font = 2, cex = 0.83)
    text(xhr, k + 1.7, "HR", adj = 1, font = 2, cex = 0.83)
    text(xci, k + 1.7, "95% CI", font = 2, cex = 0.83)
    segments(map(1), -0.1, map(1), k + 1.3, lwd = 0.8)
    for (i in seq_len(k)) {
        segments(map(loo$LowerCI[i]), y[i], map(loo$UpperCI[i]), y[i], lwd = 0.85)
        points(map(loo$Effect[i]), y[i], pch = 15, cex = 1.15, col = "#005AA8")
        text(xs, y[i], loo$Study[i], adj = 0, cex = 0.69)
        text(xhr, y[i], sprintf("%.2f", loo$Effect[i]), adj = 1, cex = 0.7)
        text(xci, y[i], sprintf("[%.2f; %.2f]", loo$LowerCI[i], loo$UpperCI[i]), cex = 0.7)
    }
    ay <- -0.15
    segments(pl, ay, pr, ay)
    for (t in ticks) {
        segments(map(t), ay, map(t), ay - 0.15)
        text(map(t), ay - 0.38, format(t, trim = TRUE), cex = 0.67)
    }
    text((pl + map(1))/2, ay - 1, "High BMI", font = 2, cex = 0.65)
    text((map(1) + pr)/2, ay - 1, "Low BMI", font = 2, cex = 0.65)
    mtext(sprintf("High BMI vs Low BMI — leave-one-out sensitivity analysis — %s, %s estimates", endpoint, tolower(adjustment)), side = 3, line = 0.5, font = 2, cex = 0.86)
}
funnel_plot <- function (d, m, title, xlab, file, note = TRUE)
{
    jpeg(file, width = 8, height = 7, units = "in", res = 300, quality = 100)
    on.exit(dev.off(), add = TRUE)
    par(mar = c(5, 5, 3.3, 1.2), family = "sans", las = 1)
    ym <- max(d$sei) * 1.08
    xc <- c(d$yi, m$mu - 1.96 * ym, m$mu + 1.96 * ym)
    pad <- diff(range(xc)) * 0.08
    plot(d$yi, d$sei, xlim = range(xc) + c(-pad, pad), ylim = c(ym, 0), pch = 21, bg = if (grepl("hazard", tolower(xlab)))
        "#2F80C0"
    else "#D0D0D0", col = if (grepl("hazard", tolower(xlab)))
        "#004C99"
    else "black", cex = 1.15, xlab = xlab, ylab = "Standard error", main = title, bty = "l")
    s <- seq(0, ym, length.out = 200)
    lines(m$mu - 1.96 * s, s, lty = 2, col = "#707070")
    lines(m$mu + 1.96 * s, s, lty = 2, col = "#707070")
    abline(v = m$mu, col = if (grepl("hazard", tolower(xlab)))
        "#D73027"
    else "#333333", lty = if (grepl("hazard", tolower(xlab)))
        1
    else 3, lwd = 1.2)
    if (note) {
        if (nrow(d) < 10)
            mtext(sprintf("Exploratory only; Egger test not performed (k = %d < 10).", nrow(d)), side = 1, line = 3.7, cex = 0.78)
    }
}
forest_highlow(d,m,endpoint,adjustment,'forest.jpg')
loo<-leave_one_out(d);sensitivity_highlow(loo,endpoint,adjustment,'sensitivity_leave_one_out.jpg')
funnel_plot(d,m,paste0('Funnel plot — ',endpoint,', ',tolower(adjustment),' estimates'),'Log hazard ratio','funnel.jpg',TRUE)
```

**Supplementary Code S4.**Progression-free survival -unadjusted estimates

```
# Analysis: 04_PFS_unadjusted
# All analysis data are embedded. Running this file creates figures only.
rm(list = ls())
options(stringsAsFactors = FALSE, scipen = 999)
analysis_id <- "04_PFS_unadjusted"
script_arg <- grep('^--file=', commandArgs(trailingOnly = FALSE), value = TRUE)
script_file <- if (length(script_arg)) sub('^--file=', '', script_arg[1]) else NA_character_
if ((is.na(script_file) || !nzchar(script_file)) && interactive() && requireNamespace('rstudioapi', quietly = TRUE)) {
  p <- tryCatch(rstudioapi::getSourceEditorContext()$path, error = function(e) '')
  if (nzchar(p)) script_file <- p
}
script_dir <- if (!is.na(script_file) && nzchar(script_file)) dirname(normalizePath(script_file, winslash='/', mustWork=TRUE)) else normalizePath(getwd(), winslash='/', mustWork=TRUE)
output_dir <- file.path(script_dir, paste0(analysis_id, '_output'))
dir.create(output_dir, recursive=TRUE, showWarnings=FALSE)
setwd(output_dir)
.libPaths(unique(c(file.path(Sys.getenv('USERPROFILE'), 'R-library'), .libPaths())))
embedded_input <- structure(list(Study = c("Kondo 2018", "McQuade (1) 2018", "McQuade (2) 2018",
"Dumenil 2018", "Shiroyama 2018", "Qi 2019", "Cortellini 2019",
"Donnelly (1) 2019", "Donnelly (2) 2019", "Katayama 2019", "Kichenadasse 2019",
"Labadie 2019", "Naik 2019", "Ichihara (1) 2020", "Ichihara (2) 2020",
"Ahmed 2021", "Bartl 2021", "Imai 2021", "Esposito 2021", "Ged 2022",
"Imai 2022", "Wang 2022", "Tateishi 2022", "Wasamoto 2023", "Incorvaia 2023",
"Vithayathil 2023", "Wu 2023", "Zhang 2023", "McManus 2023",
"Miyamoto 2023", "Santoni 2023", "Bernard 2024", "Gómez-Banoy 2024",
"Nie (1) 2024", "Nie (2) 2024", "Tan 2024", "Wang 2024", "?en 2024",
"Demir 2025", "Anami 2025"), Effect = c(0.243, 0.67, 0.8, 0.935,
0.877, 0.751, 0.48, 0.98, 1.49, 0.54, 0.86, 0.39, 1.03, 0.94,
0.58, 0.69, 0.65, 0.69, 0.79, 0.99, 0.72, 0.61, 0.86, 0.73, 0.12,
1.11, 0.72, 0.489, 1.765, 1.08, 0.79, 0.38, 0.61, 1.05, 1.05,
0.099, 0.28, 1.16, 0.82, 0.85), LowerCI = c(0.108, 0.45, 0.58,
0.366, 0.61, 0.458, 0.37, 0.59, 0.71, 0.19, 0.73, 0.19, 0.53,
0.53, 0.27, 0.51, 0.43, 0.45, 0.55, 0.71, 0.47, 0.27, 0.6, 0.45,
0.03, 0.6, 0.39, 0.064, 1.119, 0.71, 0.61, 0.148, 0.47, 0.88,
0.68, 0.013, 0.15, 0.76, 0.59, 0.63), UpperCI = c(0.543, 0.99,
1.1, 2.381, 1.25, 1.232, 0.64, 1.63, 3.13, 1.59, 1.01, 0.82,
2, 1.65, 1.28, 0.94, 0.97, 1.03, 1.14, 1.38, 1.1, 1.42, 1.25,
1.16, 0.53, 2.06, 1.32, 3.737, 2.783, 1.64, 1.02, 0.96, 0.77,
1.26, 1.63, 0.768, 0.52, 1.75, 1.31, 1.14)), row.names = c(NA,
-40L), class = "data.frame")


reml_meta <- function (yi, sei)
{
    keep <- is.finite(yi) & is.finite(sei) & sei > 0
    yi <- yi[keep]
    sei <- sei[keep]
    vi <- sei^2
    k <- length(yi)
    if (!k)
        stop("No valid estimates.")
    if (k == 1L) {
        z <- qnorm(0.975)
        return(list(k = 1L, mu = yi, se_mu = sei, ci_low = yi - z * sei, ci_high = yi + z * sei, p = 2 * pnorm(abs(yi/sei), lower.tail = FALSE), tau2 = NA_real_, Q = NA_real_, df_Q = 0L, Q_p = NA_real_, I2 = NA_real_, weights = 100))
    }
    wf <- 1/vi
    muf <- sum(wf * yi)/sum(wf)
    Q <- sum(wf * (yi - muf)^2)
    dfQ <- k - 1
    I2 <- if (Q > 0)
        max(0, (Q - dfQ)/Q) * 100
    else 0
    obj <- function(tau2) {
        w <- 1/(vi + tau2)
        mu <- sum(w * yi)/sum(w)
        0.5 * (sum(log(vi + tau2)) + log(sum(w)) + sum(w * (yi - mu)^2))
    }
    upper <- max(1, var(yi) * 10, max(vi) * 10)
    opt <- optimize(obj, c(0, upper), tol = 1e-12)
    tau2 <- if (opt$minimum < 1e-10)
        0
    else opt$minimum
    w <- 1/(vi + tau2)
    mu <- sum(w * yi)/sum(w)
    se <- sqrt(1/sum(w))
    ci <- mu + c(-1, 1) * qnorm(0.975) * se
    list(k = k, mu = mu, se_mu = se, ci_low = ci[1], ci_high = ci[2], p = 2 * pnorm(abs(mu/se), lower.tail = FALSE), tau2 = tau2, Q = Q, df_Q = dfQ, Q_p = pchisq(Q, dfQ, lower.tail = FALSE), I2 = I2, weights = w/sum(w) * 100)
}

prepare_effects <- function (d, effect_col = "Effect")
{
    d[[effect_col]] <- as.numeric(d[[effect_col]])
    d$LowerCI <- as.numeric(d$LowerCI)
    d$UpperCI <- as.numeric(d$UpperCI)
    ok <- is.finite(d[[effect_col]]) & is.finite(d$LowerCI) & is.finite(d$UpperCI) & d[[effect_col]] > 0 & d$LowerCI > 0 & d$UpperCI > d$LowerCI
    d <- d[ok, , drop = FALSE]
    d$Effect <- d[[effect_col]]
    d$yi <- log(d$Effect)
    d$sei <- (log(d$UpperCI) - log(d$LowerCI))/(2 * qnorm(0.975))
    d
}

fmt_p <- function (p)
if (!is.finite(p)) "NR" else if (p < 1e-04) "< 0.0001" else paste0("= ", formatC(p, format = "f", digits = 4))

leave_one_out <- function (d)
{
    if (nrow(d) < 3)
        return(data.frame())
    do.call(rbind, lapply(seq_len(nrow(d)), function(i) {
        m <- reml_meta(d$yi[-i], d$sei[-i])
        data.frame(Study = d$Study[i], Effect = exp(m$mu), LowerCI = exp(m$ci_low), UpperCI = exp(m$ci_high))
    }))
}

endpoint <- "PFS"
adjustment <- "Unadjusted"
d <- prepare_effects(as.data.frame(embedded_input))
m <- reml_meta(d$yi,d$sei)
forest_highlow <- function (d, m, endpoint, adjustment, file)
{
    k <- nrow(d)
    h <- max(8.5, 3.4 + 0.255 * k)
    jpeg(file, width = 11, height = h, units = "in", res = 300, quality = 100)
    on.exit(dev.off(), add = TRUE)
    par(mar = c(4.8, 0.7, 1.4, 0.7), family = "sans", xpd = NA)
    plot.new()
    plot.window(xlim = c(0, 1), ylim = c(-2.3, k + 3.2))
    xs <- 0.02
    xln <- 0.315
    xse <- 0.415
    pl <- 0.515
    pr <- 0.735
    xhr <- 0.775
    xci <- 0.86
    xw <- 0.98
    amin <- if (min(c(d$Effect, exp(m$mu))) < 0.1)
        0.01
    else 0.1
    amax <- if (max(c(d$Effect, d$UpperCI, exp(m$ci_high))) > 20)
        100
    else 10
    ticks <- if (amin == 0.01)
        c(0.01, 0.1, 0.5, 1, 2, 10)
    else c(0.1, 0.5, 1, 2, 10)
    if (amax == 100)
        ticks <- c(ticks, 100)
    map <- function(v) pl + (log(v) - log(amin))/(log(amax) - log(amin)) * (pr - pl)
    clamp <- function(v) pmin(pr, pmax(pl, map(v)))
    y <- rev(seq_len(k)) + 1
    fhr <- function(v) if (v < 0.1)
        formatC(v, format = "f", digits = 3)
    else formatC(v, format = "f", digits = 2)
    text(xs, k + 2.45, "Study", adj = 0, font = 2, cex = 0.86)
    text(xln, k + 2.45, "lnHR", adj = 1, font = 2, cex = 0.86)
    text(xse, k + 2.45, "SE(lnHR)", adj = 1, font = 2, cex = 0.86)
    text((pl + pr)/2, k + 2.45, "Hazard Ratio", font = 2, cex = 0.86)
    text(xhr, k + 2.45, "HR", adj = 1, font = 2, cex = 0.86)
    text(xci, k + 2.45, "95% CI", font = 2, cex = 0.86)
    text(xw, k + 2.45, "Weight", adj = 1, font = 2, cex = 0.86)
    segments(map(1), -0.1, map(1), k + 1.7, lwd = 0.8)
    segments(clamp(exp(m$mu)), -0.1, clamp(exp(m$mu)), k + 1.7, lty = 3, col = "#444444", lwd = 0.9)
    for (i in seq_len(k)) {
        segments(clamp(d$LowerCI[i]), y[i], clamp(d$UpperCI[i]), y[i], lwd = 0.8)
        if (d$LowerCI[i] < amin)
            arrows(pl + 0.012, y[i], pl, y[i], length = 0.055, angle = 25)
        if (d$UpperCI[i] > amax)
            arrows(pr - 0.012, y[i], pr, y[i], length = 0.055, angle = 25)
        cs <- 0.45 + 1.5 * sqrt(m$weights[i]/max(m$weights))
        points(clamp(d$Effect[i]), y[i], pch = 15, cex = cs, col = "#005AA8")
        points(clamp(d$Effect[i]), y[i], pch = 3, cex = 0.45, lwd = 0.7, col = "white")
        text(xs, y[i], d$Study[i], adj = 0, cex = 0.7)
        text(xln, y[i], formatC(d$yi[i], format = "f", digits = 4), adj = 1, cex = 0.68)
        text(xse, y[i], formatC(d$sei[i], format = "f", digits = 4), adj = 1, cex = 0.68)
        text(xhr, y[i], fhr(d$Effect[i]), adj = 1, cex = 0.7)
        text(xci, y[i], paste0("[", fhr(d$LowerCI[i]), "; ", fhr(d$UpperCI[i]), "]"), cex = 0.7)
        text(xw, y[i], sprintf("%.1f%%", m$weights[i]), adj = 1, cex = 0.7)
    }
    py <- 0.55
    polygon(c(clamp(exp(m$ci_low)), clamp(exp(m$mu)), clamp(exp(m$ci_high)), clamp(exp(m$mu))), c(py, py + 0.24, py, py - 0.24), col = "#FF1A12", border = "black", lwd = 0.8)
    text(xs, py, "Random effects model", adj = 0, font = 2, cex = 0.78)
    text(xhr, py, sprintf("%.2f", exp(m$mu)), adj = 1, font = 2, cex = 0.76)
    text(xci, py, sprintf("[%.2f; %.2f]", exp(m$ci_low), exp(m$ci_high)), font = 2, cex = 0.76)
    text(xw, py, "100.0%", adj = 1, font = 2, cex = 0.76)
    ay <- -0.25
    segments(pl, ay, pr, ay)
    for (t in ticks) {
        segments(map(t), ay, map(t), ay - 0.18)
        text(map(t), ay - 0.38, format(t, trim = TRUE), cex = 0.68)
    }
    text((pl + map(1))/2, ay - 1, "High BMI", font = 2, cex = 0.66)
    text((map(1) + pr)/2, ay - 1, "Low BMI", font = 2, cex = 0.66)
    text(xs, -1.92, sprintf("Heterogeneity: I² = %.1f%%, τ² = %.4f, Q = %.2f (df = %d), p %s", m$I2, m$tau2, m$Q, m$df_Q, fmt_p(m$Q_p)), adj = 0, cex = 0.69)
    mtext(sprintf("High BMI vs Low BMI — %s — %s estimates", endpoint, adjustment), side = 3, line = 0.1, cex = 0.76, font = 2)
}
sensitivity_highlow <- function (loo, endpoint, adjustment, file)
{
    k <- nrow(loo)
    jpeg(file, width = 10, height = max(7.5, 3.6 + 0.25 * k), units = "in", res = 300, quality = 100)
    on.exit(dev.off(), add = TRUE)
    par(mar = c(4.8, 0.8, 2.2, 0.8), family = "sans", xpd = NA)
    plot.new()
    plot.window(xlim = c(0, 1), ylim = c(-1.7, k + 2.5))
    xs <- 0.03
    pl <- 0.42
    pr <- 0.7
    xhr <- 0.76
    xci <- 0.89
    amin <- 10^floor(log10(min(0.5, min(loo$LowerCI) * 0.9)))
    amax <- 10^ceiling(log10(max(2, max(loo$UpperCI) * 1.1)))
    ticks <- c(0.01, 0.05, 0.1, 0.2, 0.5, 1, 2, 5, 10, 100)
    ticks <- ticks[ticks >= amin & ticks <= amax]
    map <- function(v) pl + (log(v) - log(amin))/(log(amax) - log(amin)) * (pr - pl)
    y <- rev(seq_len(k)) + 0.6
    text(xs, k + 1.7, "Omitted study", adj = 0, font = 2, cex = 0.83)
    text((pl + pr)/2, k + 1.7, "Pooled Hazard Ratio", font = 2, cex = 0.83)
    text(xhr, k + 1.7, "HR", adj = 1, font = 2, cex = 0.83)
    text(xci, k + 1.7, "95% CI", font = 2, cex = 0.83)
    segments(map(1), -0.1, map(1), k + 1.3, lwd = 0.8)
    for (i in seq_len(k)) {
        segments(map(loo$LowerCI[i]), y[i], map(loo$UpperCI[i]), y[i], lwd = 0.85)
        points(map(loo$Effect[i]), y[i], pch = 15, cex = 1.15, col = "#005AA8")
        text(xs, y[i], loo$Study[i], adj = 0, cex = 0.69)
        text(xhr, y[i], sprintf("%.2f", loo$Effect[i]), adj = 1, cex = 0.7)
        text(xci, y[i], sprintf("[%.2f; %.2f]", loo$LowerCI[i], loo$UpperCI[i]), cex = 0.7)
    }
    ay <- -0.15
    segments(pl, ay, pr, ay)
    for (t in ticks) {
        segments(map(t), ay, map(t), ay - 0.15)
        text(map(t), ay - 0.38, format(t, trim = TRUE), cex = 0.67)
    }
    text((pl + map(1))/2, ay - 1, "High BMI", font = 2, cex = 0.65)
    text((map(1) + pr)/2, ay - 1, "Low BMI", font = 2, cex = 0.65)
    mtext(sprintf("High BMI vs Low BMI — leave-one-out sensitivity analysis — %s, %s estimates", endpoint, tolower(adjustment)), side = 3, line = 0.5, font = 2, cex = 0.86)
}
funnel_plot <- function (d, m, title, xlab, file, note = TRUE)
{
    jpeg(file, width = 8, height = 7, units = "in", res = 300, quality = 100)
    on.exit(dev.off(), add = TRUE)
    par(mar = c(5, 5, 3.3, 1.2), family = "sans", las = 1)
    ym <- max(d$sei) * 1.08
    xc <- c(d$yi, m$mu - 1.96 * ym, m$mu + 1.96 * ym)
    pad <- diff(range(xc)) * 0.08
    plot(d$yi, d$sei, xlim = range(xc) + c(-pad, pad), ylim = c(ym, 0), pch = 21, bg = if (grepl("hazard", tolower(xlab)))
        "#2F80C0"
    else "#D0D0D0", col = if (grepl("hazard", tolower(xlab)))
        "#004C99"
    else "black", cex = 1.15, xlab = xlab, ylab = "Standard error", main = title, bty = "l")
    s <- seq(0, ym, length.out = 200)
    lines(m$mu - 1.96 * s, s, lty = 2, col = "#707070")
    lines(m$mu + 1.96 * s, s, lty = 2, col = "#707070")
    abline(v = m$mu, col = if (grepl("hazard", tolower(xlab)))
        "#D73027"
    else "#333333", lty = if (grepl("hazard", tolower(xlab)))
        1
    else 3, lwd = 1.2)
    if (note) {
        if (nrow(d) < 10)
            mtext(sprintf("Exploratory only; Egger test not performed (k = %d < 10).", nrow(d)), side = 1, line = 3.7, cex = 0.78)
    }
}
forest_highlow(d,m,endpoint,adjustment,'forest.jpg')
loo<-leave_one_out(d);sensitivity_highlow(loo,endpoint,adjustment,'sensitivity_leave_one_out.jpg')
funnel_plot(d,m,paste0('Funnel plot — ',endpoint,', ',tolower(adjustment),' estimates'),'Log hazard ratio','funnel.jpg',TRUE)
```

**Supplementary Code S5.**Objective response rate-adjusted estimates

```
# Analysis: 05_ORR_adjusted
# All analysis data are embedded. Running this file creates figures only.
rm(list = ls())
options(stringsAsFactors = FALSE, scipen = 999)
analysis_id <- "05_ORR_adjusted"
script_arg <- grep('^--file=', commandArgs(trailingOnly = FALSE), value = TRUE)
script_file <- if (length(script_arg)) sub('^--file=', '', script_arg[1]) else NA_character_
if ((is.na(script_file) || !nzchar(script_file)) && interactive() && requireNamespace('rstudioapi', quietly = TRUE)) {
  p <- tryCatch(rstudioapi::getSourceEditorContext()$path, error = function(e) '')
  if (nzchar(p)) script_file <- p
}
script_dir <- if (!is.na(script_file) && nzchar(script_file)) dirname(normalizePath(script_file, winslash='/', mustWork=TRUE)) else normalizePath(getwd(), winslash='/', mustWork=TRUE)
output_dir <- file.path(script_dir, paste0(analysis_id, '_output'))
dir.create(output_dir, recursive=TRUE, showWarnings=FALSE)
setwd(output_dir)
.libPaths(unique(c(file.path(Sys.getenv('USERPROFILE'), 'R-library'), .libPaths())))
embedded_input <- structure(list(Study = c("Cortellini 2019", "Cortellini 2020",
"Esposito 2021", "Jain 2023", "McManus 2023", "Alam 2024", "Bernard 2024",
"Wang 2024"), Effect = c(1.56, 1.61, 0.96, 2.67, 0.268, 1.18,
2.62, 6.22), LowerCI = c(1.061, 1.04, 0.3, 1.11, 0.078, 0.81,
0.66, 1.75), UpperCI = c(2.295, 2.5, 3.1, 6.4, 0.773, 1.73, 10.78,
22.17)), row.names = c(NA, -8L), class = "data.frame")


reml_meta <- function (yi, sei)
{
    keep <- is.finite(yi) & is.finite(sei) & sei > 0
    yi <- yi[keep]
    sei <- sei[keep]
    vi <- sei^2
    k <- length(yi)
    if (!k)
        stop("No valid estimates.")
    if (k == 1L) {
        z <- qnorm(0.975)
        return(list(k = 1L, mu = yi, se_mu = sei, ci_low = yi - z * sei, ci_high = yi + z * sei, p = 2 * pnorm(abs(yi/sei), lower.tail = FALSE), tau2 = NA_real_, Q = NA_real_, df_Q = 0L, Q_p = NA_real_, I2 = NA_real_, weights = 100))
    }
    wf <- 1/vi
    muf <- sum(wf * yi)/sum(wf)
    Q <- sum(wf * (yi - muf)^2)
    dfQ <- k - 1
    I2 <- if (Q > 0)
        max(0, (Q - dfQ)/Q) * 100
    else 0
    obj <- function(tau2) {
        w <- 1/(vi + tau2)
        mu <- sum(w * yi)/sum(w)
        0.5 * (sum(log(vi + tau2)) + log(sum(w)) + sum(w * (yi - mu)^2))
    }
    upper <- max(1, var(yi) * 10, max(vi) * 10)
    opt <- optimize(obj, c(0, upper), tol = 1e-12)
    tau2 <- if (opt$minimum < 1e-10)
        0
    else opt$minimum
    w <- 1/(vi + tau2)
    mu <- sum(w * yi)/sum(w)
    se <- sqrt(1/sum(w))
    ci <- mu + c(-1, 1) * qnorm(0.975) * se
    list(k = k, mu = mu, se_mu = se, ci_low = ci[1], ci_high = ci[2], p = 2 * pnorm(abs(mu/se), lower.tail = FALSE), tau2 = tau2, Q = Q, df_Q = dfQ, Q_p = pchisq(Q, dfQ, lower.tail = FALSE), I2 = I2, weights = w/sum(w) * 100)
}

prepare_effects <- function (d, effect_col = "Effect")
{
    d[[effect_col]] <- as.numeric(d[[effect_col]])
    d$LowerCI <- as.numeric(d$LowerCI)
    d$UpperCI <- as.numeric(d$UpperCI)
    ok <- is.finite(d[[effect_col]]) & is.finite(d$LowerCI) & is.finite(d$UpperCI) & d[[effect_col]] > 0 & d$LowerCI > 0 & d$UpperCI > d$LowerCI
    d <- d[ok, , drop = FALSE]
    d$Effect <- d[[effect_col]]
    d$yi <- log(d$Effect)
    d$sei <- (log(d$UpperCI) - log(d$LowerCI))/(2 * qnorm(0.975))
    d
}

fmt_p <- function (p)
if (!is.finite(p)) "NR" else if (p < 1e-04) "< 0.0001" else paste0("= ", formatC(p, format = "f", digits = 4))

leave_one_out <- function (d)
{
    if (nrow(d) < 3)
        return(data.frame())
    do.call(rbind, lapply(seq_len(nrow(d)), function(i) {
        m <- reml_meta(d$yi[-i], d$sei[-i])
        data.frame(Study = d$Study[i], Effect = exp(m$mu), LowerCI = exp(m$ci_low), UpperCI = exp(m$ci_high))
    }))
}

endpoint <- "ORR"
adjustment <- "Adjusted"
d <- prepare_effects(as.data.frame(embedded_input))
m <- reml_meta(d$yi,d$sei)
forest_binary <- function (d, m, title, subtitle, file)
{
    k <- nrow(d)
    h <- max(6.5, 5.2 + 0.33 * k)
    jpeg(file, width = 12, height = h, units = "in", res = 300, quality = 100)
    on.exit(dev.off(), add = TRUE)
    par(mar = c(5.3, 0.65, 3.5, 0.65), family = "sans", xpd = NA)
    plot.new()
    plot.window(xlim = c(0, 1), ylim = c(-3, k + 3.4))
    xs <- 0.02
    xln <- 0.3
    xse <- 0.4
    pl <- 0.51
    pr <- 0.72
    xo <- 0.77
    xci <- 0.875
    xw <- 0.985
    amin <- 0.1
    amax <- 10
    ticks <- c(0.1, 0.5, 1, 2, 10)
    map <- function(v) pl + (log(v) - log(amin))/(log(amax) - log(amin)) * (pr - pl)
    clamp <- function(v) pmin(pr, pmax(pl, map(v)))
    y <- rev(seq_len(k)) + 1.1
    text(xs, k + 2.5, "Study", adj = 0, font = 2, cex = 0.86)
    text(xln, k + 2.5, "lnOR", adj = 1, font = 2, cex = 0.86)
    text(xse, k + 2.5, "SE(lnOR)", adj = 1, font = 2, cex = 0.86)
    text((pl + pr)/2, k + 2.5, "Odds Ratio", font = 2, cex = 0.86)
    text(xo, k + 2.5, "OR", adj = 1, font = 2, cex = 0.86)
    text(xci, k + 2.5, "95% CI", font = 2, cex = 0.86)
    text(xw, k + 2.5, "Weight", adj = 1, font = 2, cex = 0.86)
    segments(map(1), -0.1, map(1), k + 1.8, lwd = 0.8)
    if (k > 1)
        segments(clamp(exp(m$mu)), -0.1, clamp(exp(m$mu)), k + 1.8, lty = 3, col = "#555555")
    for (i in seq_len(k)) {
        segments(clamp(d$LowerCI[i]), y[i], clamp(d$UpperCI[i]), y[i], lwd = 0.8)
        if (d$LowerCI[i] < amin)
            arrows(pl + 0.012, y[i], pl, y[i], length = 0.05, angle = 25)
        if (d$UpperCI[i] > amax)
            arrows(pr - 0.012, y[i], pr, y[i], length = 0.05, angle = 25)
        cs <- 0.45 + 1.5 * sqrt(m$weights[i]/max(m$weights))
        points(clamp(d$Effect[i]), y[i], pch = 15, col = "#BDBDBD", cex = cs)
        points(clamp(d$Effect[i]), y[i], pch = 3, cex = 0.42, lwd = 0.7, col = "#333333")
        text(xs, y[i], d$Study[i], adj = 0, cex = 0.7)
        text(xln, y[i], formatC(d$yi[i], format = "f", digits = 4), adj = 1, cex = 0.68)
        text(xse, y[i], formatC(d$sei[i], format = "f", digits = 4), adj = 1, cex = 0.68)
        text(xo, y[i], sprintf("%.2f", d$Effect[i]), adj = 1, cex = 0.7)
        text(xci, y[i], sprintf("[%.2f; %.2f]", d$LowerCI[i], d$UpperCI[i]), cex = 0.7)
        text(xw, y[i], sprintf("%.1f%%", m$weights[i]), adj = 1, cex = 0.7)
    }
    if (k > 1) {
        py <- 0.45
        polygon(c(clamp(exp(m$ci_low)), clamp(exp(m$mu)), clamp(exp(m$ci_high)), clamp(exp(m$mu))), c(py, py + 0.25, py, py - 0.25), col = "#C9C9C9", border = "black")
        text(xs, py, "Random effects model", adj = 0, font = 2, cex = 0.78)
        text(xo, py, sprintf("%.2f", exp(m$mu)), adj = 1, font = 2, cex = 0.76)
        text(xci, py, sprintf("[%.2f; %.2f]", exp(m$ci_low), exp(m$ci_high)), font = 2, cex = 0.76)
        text(xw, py, "100.0%", adj = 1, font = 2, cex = 0.76)
    }
    ay <- -1.1
    segments(pl, ay, pr, ay)
    for (t in ticks) {
        segments(map(t), ay, map(t), ay - 0.16)
        text(map(t), ay - 0.38, format(t, trim = TRUE), cex = 0.68)
    }
    text((pl + pr)/2, ay - 0.85, "Odds Ratio", font = 2, cex = 0.72)
    if (k > 1) {
        pi_se <- sqrt(m$tau2 + m$se_mu^2)
        crit <- qt(0.975, df = max(1, k - 2))
        pi <- exp(m$mu + c(-1, 1) * crit * pi_se)
        text(xs, -2.25, sprintf("Pooled-effect P = %.3f; Heterogeneity: I² = %.1f%%, tau² = %.4f, Q = %.2f (df = %d), P_heterogeneity = %.3f", m$p, m$I2, m$tau2, m$Q, m$df_Q, m$Q_p), adj = 0, cex = 0.65)
        text(xs, -2.7, sprintf("95%% prediction interval: [%.2f; %.2f]", pi[1], pi[2]), adj = 0, cex = 0.65)
    }
    mtext(title, side = 3, line = 1.25, font = 2, cex = 0.95)
    mtext(subtitle, side = 3, line = 0.1, cex = 0.68, col = "#444444")
}
sensitivity_binary <- function (loo, title, file)
{
    k <- nrow(loo)
    jpeg(file, width = 11.5, height = max(6.3, 4.3 + 0.33 * k), units = "in", res = 300, quality = 100)
    on.exit(dev.off(), add = TRUE)
    par(mar = c(5.2, 7.2, 3.3, 1.2), family = "sans", las = 1)
    y <- rev(seq_len(k))
    xl <- range(c(0.1, 10, loo$LowerCI, loo$UpperCI))
    plot(loo$Effect, y, log = "x", xlim = xl, yaxt = "n", pch = 15, col = "#888888", xlab = "Odds Ratio", ylab = "", main = title, bty = "n")
    segments(loo$LowerCI, y, loo$UpperCI, y)
    abline(v = 1, lty = 2, col = "#555555")
    axis(2, at = y, labels = paste("Omit", loo$Study), las = 1, tick = TRUE, cex.axis = 0.8)
    mtext("Each row is a REML random-effects estimate after omitting the named study.", side = 1, line = 3.7, cex = 0.72)
}
funnel_plot <- function (d, m, title, xlab, file, note = TRUE)
{
    jpeg(file, width = 8, height = 7, units = "in", res = 300, quality = 100)
    on.exit(dev.off(), add = TRUE)
    par(mar = c(5, 5, 3.3, 1.2), family = "sans", las = 1)
    ym <- max(d$sei) * 1.08
    xc <- c(d$yi, m$mu - 1.96 * ym, m$mu + 1.96 * ym)
    pad <- diff(range(xc)) * 0.08
    plot(d$yi, d$sei, xlim = range(xc) + c(-pad, pad), ylim = c(ym, 0), pch = 21, bg = if (grepl("hazard", tolower(xlab)))
        "#2F80C0"
    else "#D0D0D0", col = if (grepl("hazard", tolower(xlab)))
        "#004C99"
    else "black", cex = 1.15, xlab = xlab, ylab = "Standard error", main = title, bty = "l")
    s <- seq(0, ym, length.out = 200)
    lines(m$mu - 1.96 * s, s, lty = 2, col = "#707070")
    lines(m$mu + 1.96 * s, s, lty = 2, col = "#707070")
    abline(v = m$mu, col = if (grepl("hazard", tolower(xlab)))
        "#D73027"
    else "#333333", lty = if (grepl("hazard", tolower(xlab)))
        1
    else 3, lwd = 1.2)
    if (note) {
        if (nrow(d) < 10)
            mtext(sprintf("Exploratory only; Egger test not performed (k = %d < 10).", nrow(d)), side = 1, line = 3.7, cex = 0.78)
    }
}
forest_binary(d,m,paste0('High BMI vs Low BMI — ',"Objective response rate",' — ',adjustment,' estimates'),"OR >1 indicates greater odds of response in the high-BMI group.",'forest.jpg')
if(nrow(d)>=3){loo<-leave_one_out(d);sensitivity_binary(loo,paste0("Objective response rate",' — ',adjustment,' leave-one-out analysis'),'sensitivity_leave_one_out.jpg')}
if(nrow(d)>=3)funnel_plot(d,m,paste0("Objective response rate",' — ',adjustment,' funnel plot'),'Log odds ratio','funnel.jpg',TRUE)
```

**Supplementary Code S6.**Objective response rate-unadjusted estimates

```
# Analysis: 06_ORR_unadjusted
# All analysis data are embedded. Running this file creates figures only.
rm(list = ls())
options(stringsAsFactors = FALSE, scipen = 999)
analysis_id <- "06_ORR_unadjusted"
script_arg <- grep('^--file=', commandArgs(trailingOnly = FALSE), value = TRUE)
script_file <- if (length(script_arg)) sub('^--file=', '', script_arg[1]) else NA_character_
if ((is.na(script_file) || !nzchar(script_file)) && interactive() && requireNamespace('rstudioapi', quietly = TRUE)) {
  p <- tryCatch(rstudioapi::getSourceEditorContext()$path, error = function(e) '')
  if (nzchar(p)) script_file <- p
}
script_dir <- if (!is.na(script_file) && nzchar(script_file)) dirname(normalizePath(script_file, winslash='/', mustWork=TRUE)) else normalizePath(getwd(), winslash='/', mustWork=TRUE)
output_dir <- file.path(script_dir, paste0(analysis_id, '_output'))
dir.create(output_dir, recursive=TRUE, showWarnings=FALSE)
setwd(output_dir)
.libPaths(unique(c(file.path(Sys.getenv('USERPROFILE'), 'R-library'), .libPaths())))
embedded_input <- structure(list(Study = c("Cortellini 2019", "Giorgi 2019", "Rogado 2020",
"Minami 2020", "Imai 2021", "Esposito 2021", "Chen 2022", "Ged 2022",
"Imai 2022", "Tateishi 2022", "Wasamoto 2023", "Incorvaia 2023",
"Vithayathil 2023", "McManus 2023", "Miyamoto 2023", "Bernard 2024",
"Nie 2024", "?en 2024", "Yamamoto 2024", "Demir 2025", "Krej?í 2025",
"Anami 2025"), Effect = c(2.668, 1.168, 2.4, 1.06, 3.37, 0.93,
2.29, 0.89, 1.06, 0.87, 1.61, 0.48, 1.33, 0.364, 0.72, 3.2, 1.52,
0.84, 1.17, 0.98, 2.21, 1.4), LowerCI = c(1.99, 0.675, 0.81,
0.2, 1.65, 0.31, 0.6, 0.44, 0.39, 0.43, 0.61, 0.11, 0.65, 0.132,
0.32, 0.9, 0.7, 0.28, 0.35, 0.6, 1.09, 0.74), UpperCI = c(3.577,
2.022, 7.13, 5.55, 6.86, 2.79, 8.67, 1.8, 2.88, 1.73, 4.27, 2.04,
2.71, 1.005, 1.64, 12.3, 3.3, 2.56, 3.88, 1.6, 4.51, 2.62)), row.names = c(NA,
-22L), class = "data.frame")


reml_meta <- function (yi, sei)
{
    keep <- is.finite(yi) & is.finite(sei) & sei > 0
    yi <- yi[keep]
    sei <- sei[keep]
    vi <- sei^2
    k <- length(yi)
    if (!k)
        stop("No valid estimates.")
    if (k == 1L) {
        z <- qnorm(0.975)
        return(list(k = 1L, mu = yi, se_mu = sei, ci_low = yi - z * sei, ci_high = yi + z * sei, p = 2 * pnorm(abs(yi/sei), lower.tail = FALSE), tau2 = NA_real_, Q = NA_real_, df_Q = 0L, Q_p = NA_real_, I2 = NA_real_, weights = 100))
    }
    wf <- 1/vi
    muf <- sum(wf * yi)/sum(wf)
    Q <- sum(wf * (yi - muf)^2)
    dfQ <- k - 1
    I2 <- if (Q > 0)
        max(0, (Q - dfQ)/Q) * 100
    else 0
    obj <- function(tau2) {
        w <- 1/(vi + tau2)
        mu <- sum(w * yi)/sum(w)
        0.5 * (sum(log(vi + tau2)) + log(sum(w)) + sum(w * (yi - mu)^2))
    }
    upper <- max(1, var(yi) * 10, max(vi) * 10)
    opt <- optimize(obj, c(0, upper), tol = 1e-12)
    tau2 <- if (opt$minimum < 1e-10)
        0
    else opt$minimum
    w <- 1/(vi + tau2)
    mu <- sum(w * yi)/sum(w)
    se <- sqrt(1/sum(w))
    ci <- mu + c(-1, 1) * qnorm(0.975) * se
    list(k = k, mu = mu, se_mu = se, ci_low = ci[1], ci_high = ci[2], p = 2 * pnorm(abs(mu/se), lower.tail = FALSE), tau2 = tau2, Q = Q, df_Q = dfQ, Q_p = pchisq(Q, dfQ, lower.tail = FALSE), I2 = I2, weights = w/sum(w) * 100)
}

prepare_effects <- function (d, effect_col = "Effect")
{
    d[[effect_col]] <- as.numeric(d[[effect_col]])
    d$LowerCI <- as.numeric(d$LowerCI)
    d$UpperCI <- as.numeric(d$UpperCI)
    ok <- is.finite(d[[effect_col]]) & is.finite(d$LowerCI) & is.finite(d$UpperCI) & d[[effect_col]] > 0 & d$LowerCI > 0 & d$UpperCI > d$LowerCI
    d <- d[ok, , drop = FALSE]
    d$Effect <- d[[effect_col]]
    d$yi <- log(d$Effect)
    d$sei <- (log(d$UpperCI) - log(d$LowerCI))/(2 * qnorm(0.975))
    d
}

fmt_p <- function (p)
if (!is.finite(p)) "NR" else if (p < 1e-04) "< 0.0001" else paste0("= ", formatC(p, format = "f", digits = 4))

leave_one_out <- function (d)
{
    if (nrow(d) < 3)
        return(data.frame())
    do.call(rbind, lapply(seq_len(nrow(d)), function(i) {
        m <- reml_meta(d$yi[-i], d$sei[-i])
        data.frame(Study = d$Study[i], Effect = exp(m$mu), LowerCI = exp(m$ci_low), UpperCI = exp(m$ci_high))
    }))
}

endpoint <- "ORR"
adjustment <- "Unadjusted"
d <- prepare_effects(as.data.frame(embedded_input))
m <- reml_meta(d$yi,d$sei)
forest_binary <- function (d, m, title, subtitle, file)
{
    k <- nrow(d)
    h <- max(6.5, 5.2 + 0.33 * k)
    jpeg(file, width = 12, height = h, units = "in", res = 300, quality = 100)
    on.exit(dev.off(), add = TRUE)
    par(mar = c(5.3, 0.65, 3.5, 0.65), family = "sans", xpd = NA)
    plot.new()
    plot.window(xlim = c(0, 1), ylim = c(-3, k + 3.4))
    xs <- 0.02
    xln <- 0.3
    xse <- 0.4
    pl <- 0.51
    pr <- 0.72
    xo <- 0.77
    xci <- 0.875
    xw <- 0.985
    amin <- 0.1
    amax <- 10
    ticks <- c(0.1, 0.5, 1, 2, 10)
    map <- function(v) pl + (log(v) - log(amin))/(log(amax) - log(amin)) * (pr - pl)
    clamp <- function(v) pmin(pr, pmax(pl, map(v)))
    y <- rev(seq_len(k)) + 1.1
    text(xs, k + 2.5, "Study", adj = 0, font = 2, cex = 0.86)
    text(xln, k + 2.5, "lnOR", adj = 1, font = 2, cex = 0.86)
    text(xse, k + 2.5, "SE(lnOR)", adj = 1, font = 2, cex = 0.86)
    text((pl + pr)/2, k + 2.5, "Odds Ratio", font = 2, cex = 0.86)
    text(xo, k + 2.5, "OR", adj = 1, font = 2, cex = 0.86)
    text(xci, k + 2.5, "95% CI", font = 2, cex = 0.86)
    text(xw, k + 2.5, "Weight", adj = 1, font = 2, cex = 0.86)
    segments(map(1), -0.1, map(1), k + 1.8, lwd = 0.8)
    if (k > 1)
        segments(clamp(exp(m$mu)), -0.1, clamp(exp(m$mu)), k + 1.8, lty = 3, col = "#555555")
    for (i in seq_len(k)) {
        segments(clamp(d$LowerCI[i]), y[i], clamp(d$UpperCI[i]), y[i], lwd = 0.8)
        if (d$LowerCI[i] < amin)
            arrows(pl + 0.012, y[i], pl, y[i], length = 0.05, angle = 25)
        if (d$UpperCI[i] > amax)
            arrows(pr - 0.012, y[i], pr, y[i], length = 0.05, angle = 25)
        cs <- 0.45 + 1.5 * sqrt(m$weights[i]/max(m$weights))
        points(clamp(d$Effect[i]), y[i], pch = 15, col = "#BDBDBD", cex = cs)
        points(clamp(d$Effect[i]), y[i], pch = 3, cex = 0.42, lwd = 0.7, col = "#333333")
        text(xs, y[i], d$Study[i], adj = 0, cex = 0.7)
        text(xln, y[i], formatC(d$yi[i], format = "f", digits = 4), adj = 1, cex = 0.68)
        text(xse, y[i], formatC(d$sei[i], format = "f", digits = 4), adj = 1, cex = 0.68)
        text(xo, y[i], sprintf("%.2f", d$Effect[i]), adj = 1, cex = 0.7)
        text(xci, y[i], sprintf("[%.2f; %.2f]", d$LowerCI[i], d$UpperCI[i]), cex = 0.7)
        text(xw, y[i], sprintf("%.1f%%", m$weights[i]), adj = 1, cex = 0.7)
    }
    if (k > 1) {
        py <- 0.45
        polygon(c(clamp(exp(m$ci_low)), clamp(exp(m$mu)), clamp(exp(m$ci_high)), clamp(exp(m$mu))), c(py, py + 0.25, py, py - 0.25), col = "#C9C9C9", border = "black")
        text(xs, py, "Random effects model", adj = 0, font = 2, cex = 0.78)
        text(xo, py, sprintf("%.2f", exp(m$mu)), adj = 1, font = 2, cex = 0.76)
        text(xci, py, sprintf("[%.2f; %.2f]", exp(m$ci_low), exp(m$ci_high)), font = 2, cex = 0.76)
        text(xw, py, "100.0%", adj = 1, font = 2, cex = 0.76)
    }
    ay <- -1.1
    segments(pl, ay, pr, ay)
    for (t in ticks) {
        segments(map(t), ay, map(t), ay - 0.16)
        text(map(t), ay - 0.38, format(t, trim = TRUE), cex = 0.68)
    }
    text((pl + pr)/2, ay - 0.85, "Odds Ratio", font = 2, cex = 0.72)
    if (k > 1) {
        pi_se <- sqrt(m$tau2 + m$se_mu^2)
        crit <- qt(0.975, df = max(1, k - 2))
        pi <- exp(m$mu + c(-1, 1) * crit * pi_se)
        text(xs, -2.25, sprintf("Pooled-effect P = %.3f; Heterogeneity: I² = %.1f%%, tau² = %.4f, Q = %.2f (df = %d), P_heterogeneity = %.3f", m$p, m$I2, m$tau2, m$Q, m$df_Q, m$Q_p), adj = 0, cex = 0.65)
        text(xs, -2.7, sprintf("95%% prediction interval: [%.2f; %.2f]", pi[1], pi[2]), adj = 0, cex = 0.65)
    }
    mtext(title, side = 3, line = 1.25, font = 2, cex = 0.95)
    mtext(subtitle, side = 3, line = 0.1, cex = 0.68, col = "#444444")
}
sensitivity_binary <- function (loo, title, file)
{
    k <- nrow(loo)
    jpeg(file, width = 11.5, height = max(6.3, 4.3 + 0.33 * k), units = "in", res = 300, quality = 100)
    on.exit(dev.off(), add = TRUE)
    par(mar = c(5.2, 7.2, 3.3, 1.2), family = "sans", las = 1)
    y <- rev(seq_len(k))
    xl <- range(c(0.1, 10, loo$LowerCI, loo$UpperCI))
    plot(loo$Effect, y, log = "x", xlim = xl, yaxt = "n", pch = 15, col = "#888888", xlab = "Odds Ratio", ylab = "", main = title, bty = "n")
    segments(loo$LowerCI, y, loo$UpperCI, y)
    abline(v = 1, lty = 2, col = "#555555")
    axis(2, at = y, labels = paste("Omit", loo$Study), las = 1, tick = TRUE, cex.axis = 0.8)
    mtext("Each row is a REML random-effects estimate after omitting the named study.", side = 1, line = 3.7, cex = 0.72)
}
funnel_plot <- function (d, m, title, xlab, file, note = TRUE)
{
    jpeg(file, width = 8, height = 7, units = "in", res = 300, quality = 100)
    on.exit(dev.off(), add = TRUE)
    par(mar = c(5, 5, 3.3, 1.2), family = "sans", las = 1)
    ym <- max(d$sei) * 1.08
    xc <- c(d$yi, m$mu - 1.96 * ym, m$mu + 1.96 * ym)
    pad <- diff(range(xc)) * 0.08
    plot(d$yi, d$sei, xlim = range(xc) + c(-pad, pad), ylim = c(ym, 0), pch = 21, bg = if (grepl("hazard", tolower(xlab)))
        "#2F80C0"
    else "#D0D0D0", col = if (grepl("hazard", tolower(xlab)))
        "#004C99"
    else "black", cex = 1.15, xlab = xlab, ylab = "Standard error", main = title, bty = "l")
    s <- seq(0, ym, length.out = 200)
    lines(m$mu - 1.96 * s, s, lty = 2, col = "#707070")
    lines(m$mu + 1.96 * s, s, lty = 2, col = "#707070")
    abline(v = m$mu, col = if (grepl("hazard", tolower(xlab)))
        "#D73027"
    else "#333333", lty = if (grepl("hazard", tolower(xlab)))
        1
    else 3, lwd = 1.2)
    if (note) {
        if (nrow(d) < 10)
            mtext(sprintf("Exploratory only; Egger test not performed (k = %d < 10).", nrow(d)), side = 1, line = 3.7, cex = 0.78)
    }
}
forest_binary(d,m,paste0('High BMI vs Low BMI — ',"Objective response rate",' — ',adjustment,' estimates'),"OR >1 indicates greater odds of response in the high-BMI group.",'forest.jpg')
if(nrow(d)>=3){loo<-leave_one_out(d);sensitivity_binary(loo,paste0("Objective response rate",' — ',adjustment,' leave-one-out analysis'),'sensitivity_leave_one_out.jpg')}
if(nrow(d)>=3)funnel_plot(d,m,paste0("Objective response rate",' — ',adjustment,' funnel plot'),'Log odds ratio','funnel.jpg',TRUE)
```

**Supplementary Code S7.**Any-grade irAEs-adjusted estimates

```
# Analysis: 07_any_grade_irAEs_adjusted
# All analysis data are embedded. Running this file creates figures only.
rm(list = ls())
options(stringsAsFactors = FALSE, scipen = 999)
analysis_id <- "07_any_grade_irAEs_adjusted"
script_arg <- grep('^--file=', commandArgs(trailingOnly = FALSE), value = TRUE)
script_file <- if (length(script_arg)) sub('^--file=', '', script_arg[1]) else NA_character_
if ((is.na(script_file) || !nzchar(script_file)) && interactive() && requireNamespace('rstudioapi', quietly = TRUE)) {
  p <- tryCatch(rstudioapi::getSourceEditorContext()$path, error = function(e) '')
  if (nzchar(p)) script_file <- p
}
script_dir <- if (!is.na(script_file) && nzchar(script_file)) dirname(normalizePath(script_file, winslash='/', mustWork=TRUE)) else normalizePath(getwd(), winslash='/', mustWork=TRUE)
output_dir <- file.path(script_dir, paste0(analysis_id, '_output'))
dir.create(output_dir, recursive=TRUE, showWarnings=FALSE)
setwd(output_dir)
.libPaths(unique(c(file.path(Sys.getenv('USERPROFILE'), 'R-library'), .libPaths())))
embedded_input <- structure(list(Study = c("Llerena 2024", "Mastrolonardo 2025"
), Effect = c(0.7, 1.16), LowerCI = c(0.37, 1.1), UpperCI = c(1.31,
1.24)), row.names = c(NA, -2L), class = "data.frame")


reml_meta <- function (yi, sei)
{
    keep <- is.finite(yi) & is.finite(sei) & sei > 0
    yi <- yi[keep]
    sei <- sei[keep]
    vi <- sei^2
    k <- length(yi)
    if (!k)
        stop("No valid estimates.")
    if (k == 1L) {
        z <- qnorm(0.975)
        return(list(k = 1L, mu = yi, se_mu = sei, ci_low = yi - z * sei, ci_high = yi + z * sei, p = 2 * pnorm(abs(yi/sei), lower.tail = FALSE), tau2 = NA_real_, Q = NA_real_, df_Q = 0L, Q_p = NA_real_, I2 = NA_real_, weights = 100))
    }
    wf <- 1/vi
    muf <- sum(wf * yi)/sum(wf)
    Q <- sum(wf * (yi - muf)^2)
    dfQ <- k - 1
    I2 <- if (Q > 0)
        max(0, (Q - dfQ)/Q) * 100
    else 0
    obj <- function(tau2) {
        w <- 1/(vi + tau2)
        mu <- sum(w * yi)/sum(w)
        0.5 * (sum(log(vi + tau2)) + log(sum(w)) + sum(w * (yi - mu)^2))
    }
    upper <- max(1, var(yi) * 10, max(vi) * 10)
    opt <- optimize(obj, c(0, upper), tol = 1e-12)
    tau2 <- if (opt$minimum < 1e-10)
        0
    else opt$minimum
    w <- 1/(vi + tau2)
    mu <- sum(w * yi)/sum(w)
    se <- sqrt(1/sum(w))
    ci <- mu + c(-1, 1) * qnorm(0.975) * se
    list(k = k, mu = mu, se_mu = se, ci_low = ci[1], ci_high = ci[2], p = 2 * pnorm(abs(mu/se), lower.tail = FALSE), tau2 = tau2, Q = Q, df_Q = dfQ, Q_p = pchisq(Q, dfQ, lower.tail = FALSE), I2 = I2, weights = w/sum(w) * 100)
}

prepare_effects <- function (d, effect_col = "Effect")
{
    d[[effect_col]] <- as.numeric(d[[effect_col]])
    d$LowerCI <- as.numeric(d$LowerCI)
    d$UpperCI <- as.numeric(d$UpperCI)
    ok <- is.finite(d[[effect_col]]) & is.finite(d$LowerCI) & is.finite(d$UpperCI) & d[[effect_col]] > 0 & d$LowerCI > 0 & d$UpperCI > d$LowerCI
    d <- d[ok, , drop = FALSE]
    d$Effect <- d[[effect_col]]
    d$yi <- log(d$Effect)
    d$sei <- (log(d$UpperCI) - log(d$LowerCI))/(2 * qnorm(0.975))
    d
}

fmt_p <- function (p)
if (!is.finite(p)) "NR" else if (p < 1e-04) "< 0.0001" else paste0("= ", formatC(p, format = "f", digits = 4))

leave_one_out <- function (d)
{
    if (nrow(d) < 3)
        return(data.frame())
    do.call(rbind, lapply(seq_len(nrow(d)), function(i) {
        m <- reml_meta(d$yi[-i], d$sei[-i])
        data.frame(Study = d$Study[i], Effect = exp(m$mu), LowerCI = exp(m$ci_low), UpperCI = exp(m$ci_high))
    }))
}

endpoint <- "Any-grade irAEs"
adjustment <- "Adjusted"
d <- prepare_effects(as.data.frame(embedded_input))
m <- reml_meta(d$yi,d$sei)
forest_binary <- function (d, m, title, subtitle, file)
{
    k <- nrow(d)
    h <- max(6.5, 5.2 + 0.33 * k)
    jpeg(file, width = 12, height = h, units = "in", res = 300, quality = 100)
    on.exit(dev.off(), add = TRUE)
    par(mar = c(5.3, 0.65, 3.5, 0.65), family = "sans", xpd = NA)
    plot.new()
    plot.window(xlim = c(0, 1), ylim = c(-3, k + 3.4))
    xs <- 0.02
    xln <- 0.3
    xse <- 0.4
    pl <- 0.51
    pr <- 0.72
    xo <- 0.77
    xci <- 0.875
    xw <- 0.985
    amin <- 0.1
    amax <- 10
    ticks <- c(0.1, 0.5, 1, 2, 10)
    map <- function(v) pl + (log(v) - log(amin))/(log(amax) - log(amin)) * (pr - pl)
    clamp <- function(v) pmin(pr, pmax(pl, map(v)))
    y <- rev(seq_len(k)) + 1.1
    text(xs, k + 2.5, "Study", adj = 0, font = 2, cex = 0.86)
    text(xln, k + 2.5, "lnOR", adj = 1, font = 2, cex = 0.86)
    text(xse, k + 2.5, "SE(lnOR)", adj = 1, font = 2, cex = 0.86)
    text((pl + pr)/2, k + 2.5, "Odds Ratio", font = 2, cex = 0.86)
    text(xo, k + 2.5, "OR", adj = 1, font = 2, cex = 0.86)
    text(xci, k + 2.5, "95% CI", font = 2, cex = 0.86)
    text(xw, k + 2.5, "Weight", adj = 1, font = 2, cex = 0.86)
    segments(map(1), -0.1, map(1), k + 1.8, lwd = 0.8)
    if (k > 1)
        segments(clamp(exp(m$mu)), -0.1, clamp(exp(m$mu)), k + 1.8, lty = 3, col = "#555555")
    for (i in seq_len(k)) {
        segments(clamp(d$LowerCI[i]), y[i], clamp(d$UpperCI[i]), y[i], lwd = 0.8)
        if (d$LowerCI[i] < amin)
            arrows(pl + 0.012, y[i], pl, y[i], length = 0.05, angle = 25)
        if (d$UpperCI[i] > amax)
            arrows(pr - 0.012, y[i], pr, y[i], length = 0.05, angle = 25)
        cs <- 0.45 + 1.5 * sqrt(m$weights[i]/max(m$weights))
        points(clamp(d$Effect[i]), y[i], pch = 15, col = "#BDBDBD", cex = cs)
        points(clamp(d$Effect[i]), y[i], pch = 3, cex = 0.42, lwd = 0.7, col = "#333333")
        text(xs, y[i], d$Study[i], adj = 0, cex = 0.7)
        text(xln, y[i], formatC(d$yi[i], format = "f", digits = 4), adj = 1, cex = 0.68)
        text(xse, y[i], formatC(d$sei[i], format = "f", digits = 4), adj = 1, cex = 0.68)
        text(xo, y[i], sprintf("%.2f", d$Effect[i]), adj = 1, cex = 0.7)
        text(xci, y[i], sprintf("[%.2f; %.2f]", d$LowerCI[i], d$UpperCI[i]), cex = 0.7)
        text(xw, y[i], sprintf("%.1f%%", m$weights[i]), adj = 1, cex = 0.7)
    }
    if (k > 1) {
        py <- 0.45
        polygon(c(clamp(exp(m$ci_low)), clamp(exp(m$mu)), clamp(exp(m$ci_high)), clamp(exp(m$mu))), c(py, py + 0.25, py, py - 0.25), col = "#C9C9C9", border = "black")
        text(xs, py, "Random effects model", adj = 0, font = 2, cex = 0.78)
        text(xo, py, sprintf("%.2f", exp(m$mu)), adj = 1, font = 2, cex = 0.76)
        text(xci, py, sprintf("[%.2f; %.2f]", exp(m$ci_low), exp(m$ci_high)), font = 2, cex = 0.76)
        text(xw, py, "100.0%", adj = 1, font = 2, cex = 0.76)
    }
    ay <- -1.1
    segments(pl, ay, pr, ay)
    for (t in ticks) {
        segments(map(t), ay, map(t), ay - 0.16)
        text(map(t), ay - 0.38, format(t, trim = TRUE), cex = 0.68)
    }
    text((pl + pr)/2, ay - 0.85, "Odds Ratio", font = 2, cex = 0.72)
    if (k > 1) {
        pi_se <- sqrt(m$tau2 + m$se_mu^2)
        crit <- qt(0.975, df = max(1, k - 2))
        pi <- exp(m$mu + c(-1, 1) * crit * pi_se)
        text(xs, -2.25, sprintf("Pooled-effect P = %.3f; Heterogeneity: I² = %.1f%%, tau² = %.4f, Q = %.2f (df = %d), P_heterogeneity = %.3f", m$p, m$I2, m$tau2, m$Q, m$df_Q, m$Q_p), adj = 0, cex = 0.65)
        text(xs, -2.7, sprintf("95%% prediction interval: [%.2f; %.2f]", pi[1], pi[2]), adj = 0, cex = 0.65)
    }
    mtext(title, side = 3, line = 1.25, font = 2, cex = 0.95)
    mtext(subtitle, side = 3, line = 0.1, cex = 0.68, col = "#444444")
}
sensitivity_binary <- function (loo, title, file)
{
    k <- nrow(loo)
    jpeg(file, width = 11.5, height = max(6.3, 4.3 + 0.33 * k), units = "in", res = 300, quality = 100)
    on.exit(dev.off(), add = TRUE)
    par(mar = c(5.2, 7.2, 3.3, 1.2), family = "sans", las = 1)
    y <- rev(seq_len(k))
    xl <- range(c(0.1, 10, loo$LowerCI, loo$UpperCI))
    plot(loo$Effect, y, log = "x", xlim = xl, yaxt = "n", pch = 15, col = "#888888", xlab = "Odds Ratio", ylab = "", main = title, bty = "n")
    segments(loo$LowerCI, y, loo$UpperCI, y)
    abline(v = 1, lty = 2, col = "#555555")
    axis(2, at = y, labels = paste("Omit", loo$Study), las = 1, tick = TRUE, cex.axis = 0.8)
    mtext("Each row is a REML random-effects estimate after omitting the named study.", side = 1, line = 3.7, cex = 0.72)
}
funnel_plot <- function (d, m, title, xlab, file, note = TRUE)
{
    jpeg(file, width = 8, height = 7, units = "in", res = 300, quality = 100)
    on.exit(dev.off(), add = TRUE)
    par(mar = c(5, 5, 3.3, 1.2), family = "sans", las = 1)
    ym <- max(d$sei) * 1.08
    xc <- c(d$yi, m$mu - 1.96 * ym, m$mu + 1.96 * ym)
    pad <- diff(range(xc)) * 0.08
    plot(d$yi, d$sei, xlim = range(xc) + c(-pad, pad), ylim = c(ym, 0), pch = 21, bg = if (grepl("hazard", tolower(xlab)))
        "#2F80C0"
    else "#D0D0D0", col = if (grepl("hazard", tolower(xlab)))
        "#004C99"
    else "black", cex = 1.15, xlab = xlab, ylab = "Standard error", main = title, bty = "l")
    s <- seq(0, ym, length.out = 200)
    lines(m$mu - 1.96 * s, s, lty = 2, col = "#707070")
    lines(m$mu + 1.96 * s, s, lty = 2, col = "#707070")
    abline(v = m$mu, col = if (grepl("hazard", tolower(xlab)))
        "#D73027"
    else "#333333", lty = if (grepl("hazard", tolower(xlab)))
        1
    else 3, lwd = 1.2)
    if (note) {
        if (nrow(d) < 10)
            mtext(sprintf("Exploratory only; Egger test not performed (k = %d < 10).", nrow(d)), side = 1, line = 3.7, cex = 0.78)
    }
}
forest_binary(d,m,paste0('High BMI vs Low BMI — ',"Any-grade immune-related adverse events",' — ',adjustment,' estimates'),"OR >1 indicates greater odds of the event in the high-BMI group.",'forest.jpg')
if(nrow(d)>=3){loo<-leave_one_out(d);sensitivity_binary(loo,paste0("Any-grade immune-related adverse events",' — ',adjustment,' leave-one-out analysis'),'sensitivity_leave_one_out.jpg')}
if(nrow(d)>=3)funnel_plot(d,m,paste0("Any-grade immune-related adverse events",' — ',adjustment,' funnel plot'),'Log odds ratio','funnel.jpg',TRUE)
```

**Supplementary Code S8.**Any-grade irAEs — unadjusted estimates

```
# Analysis: 08_any_grade_irAEs_unadjusted
# All analysis data are embedded. Running this file creates figures only.
rm(list = ls())
options(stringsAsFactors = FALSE, scipen = 999)
analysis_id <- "08_any_grade_irAEs_unadjusted"
script_arg <- grep('^--file=', commandArgs(trailingOnly = FALSE), value = TRUE)
script_file <- if (length(script_arg)) sub('^--file=', '', script_arg[1]) else NA_character_
if ((is.na(script_file) || !nzchar(script_file)) && interactive() && requireNamespace('rstudioapi', quietly = TRUE)) {
  p <- tryCatch(rstudioapi::getSourceEditorContext()$path, error = function(e) '')
  if (nzchar(p)) script_file <- p
}
script_dir <- if (!is.na(script_file) && nzchar(script_file)) dirname(normalizePath(script_file, winslash='/', mustWork=TRUE)) else normalizePath(getwd(), winslash='/', mustWork=TRUE)
output_dir <- file.path(script_dir, paste0(analysis_id, '_output'))
dir.create(output_dir, recursive=TRUE, showWarnings=FALSE)
setwd(output_dir)
.libPaths(unique(c(file.path(Sys.getenv('USERPROFILE'), 'R-library'), .libPaths())))
embedded_input <- structure(list(Study = c("Cortellini 2019", "Kichenadasse 2019",
"Rogado 2020", "Bartl 2021", "Collet 2021", "Esposito 2021",
"Chen 2022", "Vithayathil 2023", "Zhang 2023", "Gómez-Banoy 2024",
"?en 2024", "Anami 2025"), Effect = c(3.713, 1.34, 1.42, 1.31,
1.37, 0.76, 0.54, 1.01, 2.48, 1.87, 2.28, 1.12), LowerCI = c(2.831,
0.97, 0.68, 0.23, 0.83, 0.32, 0.14, 0.57, 0.49, 1.21, 0.91, 0.63
), UpperCI = c(4.87, 1.85, 2.97, 7.57, 2.28, 1.78, 2.02, 1.8,
12.59, 2.91, 5.71, 1.99)), row.names = c(NA, -12L), class = "data.frame")


reml_meta <- function (yi, sei)
{
    keep <- is.finite(yi) & is.finite(sei) & sei > 0
    yi <- yi[keep]
    sei <- sei[keep]
    vi <- sei^2
    k <- length(yi)
    if (!k)
        stop("No valid estimates.")
    if (k == 1L) {
        z <- qnorm(0.975)
        return(list(k = 1L, mu = yi, se_mu = sei, ci_low = yi - z * sei, ci_high = yi + z * sei, p = 2 * pnorm(abs(yi/sei), lower.tail = FALSE), tau2 = NA_real_, Q = NA_real_, df_Q = 0L, Q_p = NA_real_, I2 = NA_real_, weights = 100))
    }
    wf <- 1/vi
    muf <- sum(wf * yi)/sum(wf)
    Q <- sum(wf * (yi - muf)^2)
    dfQ <- k - 1
    I2 <- if (Q > 0)
        max(0, (Q - dfQ)/Q) * 100
    else 0
    obj <- function(tau2) {
        w <- 1/(vi + tau2)
        mu <- sum(w * yi)/sum(w)
        0.5 * (sum(log(vi + tau2)) + log(sum(w)) + sum(w * (yi - mu)^2))
    }
    upper <- max(1, var(yi) * 10, max(vi) * 10)
    opt <- optimize(obj, c(0, upper), tol = 1e-12)
    tau2 <- if (opt$minimum < 1e-10)
        0
    else opt$minimum
    w <- 1/(vi + tau2)
    mu <- sum(w * yi)/sum(w)
    se <- sqrt(1/sum(w))
    ci <- mu + c(-1, 1) * qnorm(0.975) * se
    list(k = k, mu = mu, se_mu = se, ci_low = ci[1], ci_high = ci[2], p = 2 * pnorm(abs(mu/se), lower.tail = FALSE), tau2 = tau2, Q = Q, df_Q = dfQ, Q_p = pchisq(Q, dfQ, lower.tail = FALSE), I2 = I2, weights = w/sum(w) * 100)
}

prepare_effects <- function (d, effect_col = "Effect")
{
    d[[effect_col]] <- as.numeric(d[[effect_col]])
    d$LowerCI <- as.numeric(d$LowerCI)
    d$UpperCI <- as.numeric(d$UpperCI)
    ok <- is.finite(d[[effect_col]]) & is.finite(d$LowerCI) & is.finite(d$UpperCI) & d[[effect_col]] > 0 & d$LowerCI > 0 & d$UpperCI > d$LowerCI
    d <- d[ok, , drop = FALSE]
    d$Effect <- d[[effect_col]]
    d$yi <- log(d$Effect)
    d$sei <- (log(d$UpperCI) - log(d$LowerCI))/(2 * qnorm(0.975))
    d
}

fmt_p <- function (p)
if (!is.finite(p)) "NR" else if (p < 1e-04) "< 0.0001" else paste0("= ", formatC(p, format = "f", digits = 4))

leave_one_out <- function (d)
{
    if (nrow(d) < 3)
        return(data.frame())
    do.call(rbind, lapply(seq_len(nrow(d)), function(i) {
        m <- reml_meta(d$yi[-i], d$sei[-i])
        data.frame(Study = d$Study[i], Effect = exp(m$mu), LowerCI = exp(m$ci_low), UpperCI = exp(m$ci_high))
    }))
}

endpoint <- "Any-grade irAEs"
adjustment <- "Unadjusted"
d <- prepare_effects(as.data.frame(embedded_input))
m <- reml_meta(d$yi,d$sei)
forest_binary <- function (d, m, title, subtitle, file)
{
    k <- nrow(d)
    h <- max(6.5, 5.2 + 0.33 * k)
    jpeg(file, width = 12, height = h, units = "in", res = 300, quality = 100)
    on.exit(dev.off(), add = TRUE)
    par(mar = c(5.3, 0.65, 3.5, 0.65), family = "sans", xpd = NA)
    plot.new()
    plot.window(xlim = c(0, 1), ylim = c(-3, k + 3.4))
    xs <- 0.02
    xln <- 0.3
    xse <- 0.4
    pl <- 0.51
    pr <- 0.72
    xo <- 0.77
    xci <- 0.875
    xw <- 0.985
    amin <- 0.1
    amax <- 10
    ticks <- c(0.1, 0.5, 1, 2, 10)
    map <- function(v) pl + (log(v) - log(amin))/(log(amax) - log(amin)) * (pr - pl)
    clamp <- function(v) pmin(pr, pmax(pl, map(v)))
    y <- rev(seq_len(k)) + 1.1
    text(xs, k + 2.5, "Study", adj = 0, font = 2, cex = 0.86)
    text(xln, k + 2.5, "lnOR", adj = 1, font = 2, cex = 0.86)
    text(xse, k + 2.5, "SE(lnOR)", adj = 1, font = 2, cex = 0.86)
    text((pl + pr)/2, k + 2.5, "Odds Ratio", font = 2, cex = 0.86)
    text(xo, k + 2.5, "OR", adj = 1, font = 2, cex = 0.86)
    text(xci, k + 2.5, "95% CI", font = 2, cex = 0.86)
    text(xw, k + 2.5, "Weight", adj = 1, font = 2, cex = 0.86)
    segments(map(1), -0.1, map(1), k + 1.8, lwd = 0.8)
    if (k > 1)
        segments(clamp(exp(m$mu)), -0.1, clamp(exp(m$mu)), k + 1.8, lty = 3, col = "#555555")
    for (i in seq_len(k)) {
        segments(clamp(d$LowerCI[i]), y[i], clamp(d$UpperCI[i]), y[i], lwd = 0.8)
        if (d$LowerCI[i] < amin)
            arrows(pl + 0.012, y[i], pl, y[i], length = 0.05, angle = 25)
        if (d$UpperCI[i] > amax)
            arrows(pr - 0.012, y[i], pr, y[i], length = 0.05, angle = 25)
        cs <- 0.45 + 1.5 * sqrt(m$weights[i]/max(m$weights))
        points(clamp(d$Effect[i]), y[i], pch = 15, col = "#BDBDBD", cex = cs)
        points(clamp(d$Effect[i]), y[i], pch = 3, cex = 0.42, lwd = 0.7, col = "#333333")
        text(xs, y[i], d$Study[i], adj = 0, cex = 0.7)
        text(xln, y[i], formatC(d$yi[i], format = "f", digits = 4), adj = 1, cex = 0.68)
        text(xse, y[i], formatC(d$sei[i], format = "f", digits = 4), adj = 1, cex = 0.68)
        text(xo, y[i], sprintf("%.2f", d$Effect[i]), adj = 1, cex = 0.7)
        text(xci, y[i], sprintf("[%.2f; %.2f]", d$LowerCI[i], d$UpperCI[i]), cex = 0.7)
        text(xw, y[i], sprintf("%.1f%%", m$weights[i]), adj = 1, cex = 0.7)
    }
    if (k > 1) {
        py <- 0.45
        polygon(c(clamp(exp(m$ci_low)), clamp(exp(m$mu)), clamp(exp(m$ci_high)), clamp(exp(m$mu))), c(py, py + 0.25, py, py - 0.25), col = "#C9C9C9", border = "black")
        text(xs, py, "Random effects model", adj = 0, font = 2, cex = 0.78)
        text(xo, py, sprintf("%.2f", exp(m$mu)), adj = 1, font = 2, cex = 0.76)
        text(xci, py, sprintf("[%.2f; %.2f]", exp(m$ci_low), exp(m$ci_high)), font = 2, cex = 0.76)
        text(xw, py, "100.0%", adj = 1, font = 2, cex = 0.76)
    }
    ay <- -1.1
    segments(pl, ay, pr, ay)
    for (t in ticks) {
        segments(map(t), ay, map(t), ay - 0.16)
        text(map(t), ay - 0.38, format(t, trim = TRUE), cex = 0.68)
    }
    text((pl + pr)/2, ay - 0.85, "Odds Ratio", font = 2, cex = 0.72)
    if (k > 1) {
        pi_se <- sqrt(m$tau2 + m$se_mu^2)
        crit <- qt(0.975, df = max(1, k - 2))
        pi <- exp(m$mu + c(-1, 1) * crit * pi_se)
        text(xs, -2.25, sprintf("Pooled-effect P = %.3f; Heterogeneity: I² = %.1f%%, tau² = %.4f, Q = %.2f (df = %d), P_heterogeneity = %.3f", m$p, m$I2, m$tau2, m$Q, m$df_Q, m$Q_p), adj = 0, cex = 0.65)
        text(xs, -2.7, sprintf("95%% prediction interval: [%.2f; %.2f]", pi[1], pi[2]), adj = 0, cex = 0.65)
    }
    mtext(title, side = 3, line = 1.25, font = 2, cex = 0.95)
    mtext(subtitle, side = 3, line = 0.1, cex = 0.68, col = "#444444")
}
sensitivity_binary <- function (loo, title, file)
{
    k <- nrow(loo)
    jpeg(file, width = 11.5, height = max(6.3, 4.3 + 0.33 * k), units = "in", res = 300, quality = 100)
    on.exit(dev.off(), add = TRUE)
    par(mar = c(5.2, 7.2, 3.3, 1.2), family = "sans", las = 1)
    y <- rev(seq_len(k))
    xl <- range(c(0.1, 10, loo$LowerCI, loo$UpperCI))
    plot(loo$Effect, y, log = "x", xlim = xl, yaxt = "n", pch = 15, col = "#888888", xlab = "Odds Ratio", ylab = "", main = title, bty = "n")
    segments(loo$LowerCI, y, loo$UpperCI, y)
    abline(v = 1, lty = 2, col = "#555555")
    axis(2, at = y, labels = paste("Omit", loo$Study), las = 1, tick = TRUE, cex.axis = 0.8)
    mtext("Each row is a REML random-effects estimate after omitting the named study.", side = 1, line = 3.7, cex = 0.72)
}
funnel_plot <- function (d, m, title, xlab, file, note = TRUE)
{
    jpeg(file, width = 8, height = 7, units = "in", res = 300, quality = 100)
    on.exit(dev.off(), add = TRUE)
    par(mar = c(5, 5, 3.3, 1.2), family = "sans", las = 1)
    ym <- max(d$sei) * 1.08
    xc <- c(d$yi, m$mu - 1.96 * ym, m$mu + 1.96 * ym)
    pad <- diff(range(xc)) * 0.08
    plot(d$yi, d$sei, xlim = range(xc) + c(-pad, pad), ylim = c(ym, 0), pch = 21, bg = if (grepl("hazard", tolower(xlab)))
        "#2F80C0"
    else "#D0D0D0", col = if (grepl("hazard", tolower(xlab)))
        "#004C99"
    else "black", cex = 1.15, xlab = xlab, ylab = "Standard error", main = title, bty = "l")
    s <- seq(0, ym, length.out = 200)
    lines(m$mu - 1.96 * s, s, lty = 2, col = "#707070")
    lines(m$mu + 1.96 * s, s, lty = 2, col = "#707070")
    abline(v = m$mu, col = if (grepl("hazard", tolower(xlab)))
        "#D73027"
    else "#333333", lty = if (grepl("hazard", tolower(xlab)))
        1
    else 3, lwd = 1.2)
    if (note) {
        if (nrow(d) < 10)
            mtext(sprintf("Exploratory only; Egger test not performed (k = %d < 10).", nrow(d)), side = 1, line = 3.7, cex = 0.78)
    }
}
forest_binary(d,m,paste0('High BMI vs Low BMI — ',"Any-grade immune-related adverse events",' — ',adjustment,' estimates'),"OR >1 indicates greater odds of the event in the high-BMI group.",'forest.jpg')
if(nrow(d)>=3){loo<-leave_one_out(d);sensitivity_binary(loo,paste0("Any-grade immune-related adverse events",' — ',adjustment,' leave-one-out analysis'),'sensitivity_leave_one_out.jpg')}
if(nrow(d)>=3)funnel_plot(d,m,paste0("Any-grade immune-related adverse events",' — ',adjustment,' funnel plot'),'Log odds ratio','funnel.jpg',TRUE)
```

**Supplementary Code S9.**Grade ≥3 irAEs — adjusted estimates

```
# Analysis: 09_grade3_irAEs_adjusted
# All analysis data are embedded. Running this file creates figures only.
rm(list = ls())
options(stringsAsFactors = FALSE, scipen = 999)
analysis_id <- "09_grade3_irAEs_adjusted"
script_arg <- grep('^--file=', commandArgs(trailingOnly = FALSE), value = TRUE)
script_file <- if (length(script_arg)) sub('^--file=', '', script_arg[1]) else NA_character_
if ((is.na(script_file) || !nzchar(script_file)) && interactive() && requireNamespace('rstudioapi', quietly = TRUE)) {
  p <- tryCatch(rstudioapi::getSourceEditorContext()$path, error = function(e) '')
  if (nzchar(p)) script_file <- p
}
script_dir <- if (!is.na(script_file) && nzchar(script_file)) dirname(normalizePath(script_file, winslash='/', mustWork=TRUE)) else normalizePath(getwd(), winslash='/', mustWork=TRUE)
output_dir <- file.path(script_dir, paste0(analysis_id, '_output'))
dir.create(output_dir, recursive=TRUE, showWarnings=FALSE)
setwd(output_dir)
.libPaths(unique(c(file.path(Sys.getenv('USERPROFILE'), 'R-library'), .libPaths())))
embedded_input <- structure(list(Study = "Heidelberger 2017", Effect = 4.08, LowerCI = 0.95,
    UpperCI = 17.59), row.names = c(NA, -1L), class = "data.frame")


reml_meta <- function (yi, sei)
{
    keep <- is.finite(yi) & is.finite(sei) & sei > 0
    yi <- yi[keep]
    sei <- sei[keep]
    vi <- sei^2
    k <- length(yi)
    if (!k)
        stop("No valid estimates.")
    if (k == 1L) {
        z <- qnorm(0.975)
        return(list(k = 1L, mu = yi, se_mu = sei, ci_low = yi - z * sei, ci_high = yi + z * sei, p = 2 * pnorm(abs(yi/sei), lower.tail = FALSE), tau2 = NA_real_, Q = NA_real_, df_Q = 0L, Q_p = NA_real_, I2 = NA_real_, weights = 100))
    }
    wf <- 1/vi
    muf <- sum(wf * yi)/sum(wf)
    Q <- sum(wf * (yi - muf)^2)
    dfQ <- k - 1
    I2 <- if (Q > 0)
        max(0, (Q - dfQ)/Q) * 100
    else 0
    obj <- function(tau2) {
        w <- 1/(vi + tau2)
        mu <- sum(w * yi)/sum(w)
        0.5 * (sum(log(vi + tau2)) + log(sum(w)) + sum(w * (yi - mu)^2))
    }
    upper <- max(1, var(yi) * 10, max(vi) * 10)
    opt <- optimize(obj, c(0, upper), tol = 1e-12)
    tau2 <- if (opt$minimum < 1e-10)
        0
    else opt$minimum
    w <- 1/(vi + tau2)
    mu <- sum(w * yi)/sum(w)
    se <- sqrt(1/sum(w))
    ci <- mu + c(-1, 1) * qnorm(0.975) * se
    list(k = k, mu = mu, se_mu = se, ci_low = ci[1], ci_high = ci[2], p = 2 * pnorm(abs(mu/se), lower.tail = FALSE), tau2 = tau2, Q = Q, df_Q = dfQ, Q_p = pchisq(Q, dfQ, lower.tail = FALSE), I2 = I2, weights = w/sum(w) * 100)
}

prepare_effects <- function (d, effect_col = "Effect")
{
    d[[effect_col]] <- as.numeric(d[[effect_col]])
    d$LowerCI <- as.numeric(d$LowerCI)
    d$UpperCI <- as.numeric(d$UpperCI)
    ok <- is.finite(d[[effect_col]]) & is.finite(d$LowerCI) & is.finite(d$UpperCI) & d[[effect_col]] > 0 & d$LowerCI > 0 & d$UpperCI > d$LowerCI
    d <- d[ok, , drop = FALSE]
    d$Effect <- d[[effect_col]]
    d$yi <- log(d$Effect)
    d$sei <- (log(d$UpperCI) - log(d$LowerCI))/(2 * qnorm(0.975))
    d
}

fmt_p <- function (p)
if (!is.finite(p)) "NR" else if (p < 1e-04) "< 0.0001" else paste0("= ", formatC(p, format = "f", digits = 4))

leave_one_out <- function (d)
{
    if (nrow(d) < 3)
        return(data.frame())
    do.call(rbind, lapply(seq_len(nrow(d)), function(i) {
        m <- reml_meta(d$yi[-i], d$sei[-i])
        data.frame(Study = d$Study[i], Effect = exp(m$mu), LowerCI = exp(m$ci_low), UpperCI = exp(m$ci_high))
    }))
}

endpoint <- "Grade ≥3 irAEs"
adjustment <- "Adjusted"
d <- prepare_effects(as.data.frame(embedded_input))
m <- reml_meta(d$yi,d$sei)
forest_binary <- function (d, m, title, subtitle, file)
{
    k <- nrow(d)
    h <- max(6.5, 5.2 + 0.33 * k)
    jpeg(file, width = 12, height = h, units = "in", res = 300, quality = 100)
    on.exit(dev.off(), add = TRUE)
    par(mar = c(5.3, 0.65, 3.5, 0.65), family = "sans", xpd = NA)
    plot.new()
    plot.window(xlim = c(0, 1), ylim = c(-3, k + 3.4))
    xs <- 0.02
    xln <- 0.3
    xse <- 0.4
    pl <- 0.51
    pr <- 0.72
    xo <- 0.77
    xci <- 0.875
    xw <- 0.985
    amin <- 0.1
    amax <- 10
    ticks <- c(0.1, 0.5, 1, 2, 10)
    map <- function(v) pl + (log(v) - log(amin))/(log(amax) - log(amin)) * (pr - pl)
    clamp <- function(v) pmin(pr, pmax(pl, map(v)))
    y <- rev(seq_len(k)) + 1.1
    text(xs, k + 2.5, "Study", adj = 0, font = 2, cex = 0.86)
    text(xln, k + 2.5, "lnOR", adj = 1, font = 2, cex = 0.86)
    text(xse, k + 2.5, "SE(lnOR)", adj = 1, font = 2, cex = 0.86)
    text((pl + pr)/2, k + 2.5, "Odds Ratio", font = 2, cex = 0.86)
    text(xo, k + 2.5, "OR", adj = 1, font = 2, cex = 0.86)
    text(xci, k + 2.5, "95% CI", font = 2, cex = 0.86)
    text(xw, k + 2.5, "Weight", adj = 1, font = 2, cex = 0.86)
    segments(map(1), -0.1, map(1), k + 1.8, lwd = 0.8)
    if (k > 1)
        segments(clamp(exp(m$mu)), -0.1, clamp(exp(m$mu)), k + 1.8, lty = 3, col = "#555555")
    for (i in seq_len(k)) {
        segments(clamp(d$LowerCI[i]), y[i], clamp(d$UpperCI[i]), y[i], lwd = 0.8)
        if (d$LowerCI[i] < amin)
            arrows(pl + 0.012, y[i], pl, y[i], length = 0.05, angle = 25)
        if (d$UpperCI[i] > amax)
            arrows(pr - 0.012, y[i], pr, y[i], length = 0.05, angle = 25)
        cs <- 0.45 + 1.5 * sqrt(m$weights[i]/max(m$weights))
        points(clamp(d$Effect[i]), y[i], pch = 15, col = "#BDBDBD", cex = cs)
        points(clamp(d$Effect[i]), y[i], pch = 3, cex = 0.42, lwd = 0.7, col = "#333333")
        text(xs, y[i], d$Study[i], adj = 0, cex = 0.7)
        text(xln, y[i], formatC(d$yi[i], format = "f", digits = 4), adj = 1, cex = 0.68)
        text(xse, y[i], formatC(d$sei[i], format = "f", digits = 4), adj = 1, cex = 0.68)
        text(xo, y[i], sprintf("%.2f", d$Effect[i]), adj = 1, cex = 0.7)
        text(xci, y[i], sprintf("[%.2f; %.2f]", d$LowerCI[i], d$UpperCI[i]), cex = 0.7)
        text(xw, y[i], sprintf("%.1f%%", m$weights[i]), adj = 1, cex = 0.7)
    }
    if (k > 1) {
        py <- 0.45
        polygon(c(clamp(exp(m$ci_low)), clamp(exp(m$mu)), clamp(exp(m$ci_high)), clamp(exp(m$mu))), c(py, py + 0.25, py, py - 0.25), col = "#C9C9C9", border = "black")
        text(xs, py, "Random effects model", adj = 0, font = 2, cex = 0.78)
        text(xo, py, sprintf("%.2f", exp(m$mu)), adj = 1, font = 2, cex = 0.76)
        text(xci, py, sprintf("[%.2f; %.2f]", exp(m$ci_low), exp(m$ci_high)), font = 2, cex = 0.76)
        text(xw, py, "100.0%", adj = 1, font = 2, cex = 0.76)
    }
    ay <- -1.1
    segments(pl, ay, pr, ay)
    for (t in ticks) {
        segments(map(t), ay, map(t), ay - 0.16)
        text(map(t), ay - 0.38, format(t, trim = TRUE), cex = 0.68)
    }
    text((pl + pr)/2, ay - 0.85, "Odds Ratio", font = 2, cex = 0.72)
    if (k > 1) {
        pi_se <- sqrt(m$tau2 + m$se_mu^2)
        crit <- qt(0.975, df = max(1, k - 2))
        pi <- exp(m$mu + c(-1, 1) * crit * pi_se)
        text(xs, -2.25, sprintf("Pooled-effect P = %.3f; Heterogeneity: I² = %.1f%%, tau² = %.4f, Q = %.2f (df = %d), P_heterogeneity = %.3f", m$p, m$I2, m$tau2, m$Q, m$df_Q, m$Q_p), adj = 0, cex = 0.65)
        text(xs, -2.7, sprintf("95%% prediction interval: [%.2f; %.2f]", pi[1], pi[2]), adj = 0, cex = 0.65)
    }
    mtext(title, side = 3, line = 1.25, font = 2, cex = 0.95)
    mtext(subtitle, side = 3, line = 0.1, cex = 0.68, col = "#444444")
}
sensitivity_binary <- function (loo, title, file)
{
    k <- nrow(loo)
    jpeg(file, width = 11.5, height = max(6.3, 4.3 + 0.33 * k), units = "in", res = 300, quality = 100)
    on.exit(dev.off(), add = TRUE)
    par(mar = c(5.2, 7.2, 3.3, 1.2), family = "sans", las = 1)
    y <- rev(seq_len(k))
    xl <- range(c(0.1, 10, loo$LowerCI, loo$UpperCI))
    plot(loo$Effect, y, log = "x", xlim = xl, yaxt = "n", pch = 15, col = "#888888", xlab = "Odds Ratio", ylab = "", main = title, bty = "n")
    segments(loo$LowerCI, y, loo$UpperCI, y)
    abline(v = 1, lty = 2, col = "#555555")
    axis(2, at = y, labels = paste("Omit", loo$Study), las = 1, tick = TRUE, cex.axis = 0.8)
    mtext("Each row is a REML random-effects estimate after omitting the named study.", side = 1, line = 3.7, cex = 0.72)
}
funnel_plot <- function (d, m, title, xlab, file, note = TRUE)
{
    jpeg(file, width = 8, height = 7, units = "in", res = 300, quality = 100)
    on.exit(dev.off(), add = TRUE)
    par(mar = c(5, 5, 3.3, 1.2), family = "sans", las = 1)
    ym <- max(d$sei) * 1.08
    xc <- c(d$yi, m$mu - 1.96 * ym, m$mu + 1.96 * ym)
    pad <- diff(range(xc)) * 0.08
    plot(d$yi, d$sei, xlim = range(xc) + c(-pad, pad), ylim = c(ym, 0), pch = 21, bg = if (grepl("hazard", tolower(xlab)))
        "#2F80C0"
    else "#D0D0D0", col = if (grepl("hazard", tolower(xlab)))
        "#004C99"
    else "black", cex = 1.15, xlab = xlab, ylab = "Standard error", main = title, bty = "l")
    s <- seq(0, ym, length.out = 200)
    lines(m$mu - 1.96 * s, s, lty = 2, col = "#707070")
    lines(m$mu + 1.96 * s, s, lty = 2, col = "#707070")
    abline(v = m$mu, col = if (grepl("hazard", tolower(xlab)))
        "#D73027"
    else "#333333", lty = if (grepl("hazard", tolower(xlab)))
        1
    else 3, lwd = 1.2)
    if (note) {
        if (nrow(d) < 10)
            mtext(sprintf("Exploratory only; Egger test not performed (k = %d < 10).", nrow(d)), side = 1, line = 3.7, cex = 0.78)
    }
}
forest_binary(d,m,paste0('High BMI vs Low BMI — ',"Grade 3 or higher immune-related adverse events",' — ',adjustment,' estimates'),"OR >1 indicates greater odds of the event in the high-BMI group.",'forest.jpg')
if(nrow(d)>=3){loo<-leave_one_out(d);sensitivity_binary(loo,paste0("Grade 3 or higher immune-related adverse events",' — ',adjustment,' leave-one-out analysis'),'sensitivity_leave_one_out.jpg')}
if(nrow(d)>=3)funnel_plot(d,m,paste0("Grade 3 or higher immune-related adverse events",' — ',adjustment,' funnel plot'),'Log odds ratio','funnel.jpg',TRUE)
```

**Supplementary Code S10.**Grade ≥3 irAEs — unadjusted estimates

```
# Analysis: 10_grade3_irAEs_unadjusted
# All analysis data are embedded. Running this file creates figures only.
rm(list = ls())
options(stringsAsFactors = FALSE, scipen = 999)
analysis_id <- "10_grade3_irAEs_unadjusted"
script_arg <- grep('^--file=', commandArgs(trailingOnly = FALSE), value = TRUE)
script_file <- if (length(script_arg)) sub('^--file=', '', script_arg[1]) else NA_character_
if ((is.na(script_file) || !nzchar(script_file)) && interactive() && requireNamespace('rstudioapi', quietly = TRUE)) {
  p <- tryCatch(rstudioapi::getSourceEditorContext()$path, error = function(e) '')
  if (nzchar(p)) script_file <- p
}
script_dir <- if (!is.na(script_file) && nzchar(script_file)) dirname(normalizePath(script_file, winslash='/', mustWork=TRUE)) else normalizePath(getwd(), winslash='/', mustWork=TRUE)
output_dir <- file.path(script_dir, paste0(analysis_id, '_output'))
dir.create(output_dir, recursive=TRUE, showWarnings=FALSE)
setwd(output_dir)
.libPaths(unique(c(file.path(Sys.getenv('USERPROFILE'), 'R-library'), .libPaths())))
embedded_input <- structure(list(Study = c("Dumenil 2018", "Cortellini 2019", "Bartl 2021",
"Collet 2021", "Esposito 2021", "Vithayathil 2023"), Effect = c(2.84,
1.484, 3.97, 1.222619, 1, 0.35), LowerCI = c(0.54, 0.884, 0.15,
0.58208, 0.25, 0.11), UpperCI = c(14.87, 2.491, 104.18, 2.568026,
4.04, 1.13)), row.names = c(NA, -6L), class = "data.frame")


reml_meta <- function (yi, sei)
{
    keep <- is.finite(yi) & is.finite(sei) & sei > 0
    yi <- yi[keep]
    sei <- sei[keep]
    vi <- sei^2
    k <- length(yi)
    if (!k)
        stop("No valid estimates.")
    if (k == 1L) {
        z <- qnorm(0.975)
        return(list(k = 1L, mu = yi, se_mu = sei, ci_low = yi - z * sei, ci_high = yi + z * sei, p = 2 * pnorm(abs(yi/sei), lower.tail = FALSE), tau2 = NA_real_, Q = NA_real_, df_Q = 0L, Q_p = NA_real_, I2 = NA_real_, weights = 100))
    }
    wf <- 1/vi
    muf <- sum(wf * yi)/sum(wf)
    Q <- sum(wf * (yi - muf)^2)
    dfQ <- k - 1
    I2 <- if (Q > 0)
        max(0, (Q - dfQ)/Q) * 100
    else 0
    obj <- function(tau2) {
        w <- 1/(vi + tau2)
        mu <- sum(w * yi)/sum(w)
        0.5 * (sum(log(vi + tau2)) + log(sum(w)) + sum(w * (yi - mu)^2))
    }
    upper <- max(1, var(yi) * 10, max(vi) * 10)
    opt <- optimize(obj, c(0, upper), tol = 1e-12)
    tau2 <- if (opt$minimum < 1e-10)
        0
    else opt$minimum
    w <- 1/(vi + tau2)
    mu <- sum(w * yi)/sum(w)
    se <- sqrt(1/sum(w))
    ci <- mu + c(-1, 1) * qnorm(0.975) * se
    list(k = k, mu = mu, se_mu = se, ci_low = ci[1], ci_high = ci[2], p = 2 * pnorm(abs(mu/se), lower.tail = FALSE), tau2 = tau2, Q = Q, df_Q = dfQ, Q_p = pchisq(Q, dfQ, lower.tail = FALSE), I2 = I2, weights = w/sum(w) * 100)
}

prepare_effects <- function (d, effect_col = "Effect")
{
    d[[effect_col]] <- as.numeric(d[[effect_col]])
    d$LowerCI <- as.numeric(d$LowerCI)
    d$UpperCI <- as.numeric(d$UpperCI)
    ok <- is.finite(d[[effect_col]]) & is.finite(d$LowerCI) & is.finite(d$UpperCI) & d[[effect_col]] > 0 & d$LowerCI > 0 & d$UpperCI > d$LowerCI
    d <- d[ok, , drop = FALSE]
    d$Effect <- d[[effect_col]]
    d$yi <- log(d$Effect)
    d$sei <- (log(d$UpperCI) - log(d$LowerCI))/(2 * qnorm(0.975))
    d
}

fmt_p <- function (p)
if (!is.finite(p)) "NR" else if (p < 1e-04) "< 0.0001" else paste0("= ", formatC(p, format = "f", digits = 4))

leave_one_out <- function (d)
{
    if (nrow(d) < 3)
        return(data.frame())
    do.call(rbind, lapply(seq_len(nrow(d)), function(i) {
        m <- reml_meta(d$yi[-i], d$sei[-i])
        data.frame(Study = d$Study[i], Effect = exp(m$mu), LowerCI = exp(m$ci_low), UpperCI = exp(m$ci_high))
    }))
}

endpoint <- "Grade ≥3 irAEs"
adjustment <- "Unadjusted"
d <- prepare_effects(as.data.frame(embedded_input))
m <- reml_meta(d$yi,d$sei)
forest_binary <- function (d, m, title, subtitle, file)
{
    k <- nrow(d)
    h <- max(6.5, 5.2 + 0.33 * k)
    jpeg(file, width = 12, height = h, units = "in", res = 300, quality = 100)
    on.exit(dev.off(), add = TRUE)
    par(mar = c(5.3, 0.65, 3.5, 0.65), family = "sans", xpd = NA)
    plot.new()
    plot.window(xlim = c(0, 1), ylim = c(-3, k + 3.4))
    xs <- 0.02
    xln <- 0.3
    xse <- 0.4
    pl <- 0.51
    pr <- 0.72
    xo <- 0.77
    xci <- 0.875
    xw <- 0.985
    amin <- 0.1
    amax <- 10
    ticks <- c(0.1, 0.5, 1, 2, 10)
    map <- function(v) pl + (log(v) - log(amin))/(log(amax) - log(amin)) * (pr - pl)
    clamp <- function(v) pmin(pr, pmax(pl, map(v)))
    y <- rev(seq_len(k)) + 1.1
    text(xs, k + 2.5, "Study", adj = 0, font = 2, cex = 0.86)
    text(xln, k + 2.5, "lnOR", adj = 1, font = 2, cex = 0.86)
    text(xse, k + 2.5, "SE(lnOR)", adj = 1, font = 2, cex = 0.86)
    text((pl + pr)/2, k + 2.5, "Odds Ratio", font = 2, cex = 0.86)
    text(xo, k + 2.5, "OR", adj = 1, font = 2, cex = 0.86)
    text(xci, k + 2.5, "95% CI", font = 2, cex = 0.86)
    text(xw, k + 2.5, "Weight", adj = 1, font = 2, cex = 0.86)
    segments(map(1), -0.1, map(1), k + 1.8, lwd = 0.8)
    if (k > 1)
        segments(clamp(exp(m$mu)), -0.1, clamp(exp(m$mu)), k + 1.8, lty = 3, col = "#555555")
    for (i in seq_len(k)) {
        segments(clamp(d$LowerCI[i]), y[i], clamp(d$UpperCI[i]), y[i], lwd = 0.8)
        if (d$LowerCI[i] < amin)
            arrows(pl + 0.012, y[i], pl, y[i], length = 0.05, angle = 25)
        if (d$UpperCI[i] > amax)
            arrows(pr - 0.012, y[i], pr, y[i], length = 0.05, angle = 25)
        cs <- 0.45 + 1.5 * sqrt(m$weights[i]/max(m$weights))
        points(clamp(d$Effect[i]), y[i], pch = 15, col = "#BDBDBD", cex = cs)
        points(clamp(d$Effect[i]), y[i], pch = 3, cex = 0.42, lwd = 0.7, col = "#333333")
        text(xs, y[i], d$Study[i], adj = 0, cex = 0.7)
        text(xln, y[i], formatC(d$yi[i], format = "f", digits = 4), adj = 1, cex = 0.68)
        text(xse, y[i], formatC(d$sei[i], format = "f", digits = 4), adj = 1, cex = 0.68)
        text(xo, y[i], sprintf("%.2f", d$Effect[i]), adj = 1, cex = 0.7)
        text(xci, y[i], sprintf("[%.2f; %.2f]", d$LowerCI[i], d$UpperCI[i]), cex = 0.7)
        text(xw, y[i], sprintf("%.1f%%", m$weights[i]), adj = 1, cex = 0.7)
    }
    if (k > 1) {
        py <- 0.45
        polygon(c(clamp(exp(m$ci_low)), clamp(exp(m$mu)), clamp(exp(m$ci_high)), clamp(exp(m$mu))), c(py, py + 0.25, py, py - 0.25), col = "#C9C9C9", border = "black")
        text(xs, py, "Random effects model", adj = 0, font = 2, cex = 0.78)
        text(xo, py, sprintf("%.2f", exp(m$mu)), adj = 1, font = 2, cex = 0.76)
        text(xci, py, sprintf("[%.2f; %.2f]", exp(m$ci_low), exp(m$ci_high)), font = 2, cex = 0.76)
        text(xw, py, "100.0%", adj = 1, font = 2, cex = 0.76)
    }
    ay <- -1.1
    segments(pl, ay, pr, ay)
    for (t in ticks) {
        segments(map(t), ay, map(t), ay - 0.16)
        text(map(t), ay - 0.38, format(t, trim = TRUE), cex = 0.68)
    }
    text((pl + pr)/2, ay - 0.85, "Odds Ratio", font = 2, cex = 0.72)
    if (k > 1) {
        pi_se <- sqrt(m$tau2 + m$se_mu^2)
        crit <- qt(0.975, df = max(1, k - 2))
        pi <- exp(m$mu + c(-1, 1) * crit * pi_se)
        text(xs, -2.25, sprintf("Pooled-effect P = %.3f; Heterogeneity: I² = %.1f%%, tau² = %.4f, Q = %.2f (df = %d), P_heterogeneity = %.3f", m$p, m$I2, m$tau2, m$Q, m$df_Q, m$Q_p), adj = 0, cex = 0.65)
        text(xs, -2.7, sprintf("95%% prediction interval: [%.2f; %.2f]", pi[1], pi[2]), adj = 0, cex = 0.65)
    }
    mtext(title, side = 3, line = 1.25, font = 2, cex = 0.95)
    mtext(subtitle, side = 3, line = 0.1, cex = 0.68, col = "#444444")
}
sensitivity_binary <- function (loo, title, file)
{
    k <- nrow(loo)
    jpeg(file, width = 11.5, height = max(6.3, 4.3 + 0.33 * k), units = "in", res = 300, quality = 100)
    on.exit(dev.off(), add = TRUE)
    par(mar = c(5.2, 7.2, 3.3, 1.2), family = "sans", las = 1)
    y <- rev(seq_len(k))
    xl <- range(c(0.1, 10, loo$LowerCI, loo$UpperCI))
    plot(loo$Effect, y, log = "x", xlim = xl, yaxt = "n", pch = 15, col = "#888888", xlab = "Odds Ratio", ylab = "", main = title, bty = "n")
    segments(loo$LowerCI, y, loo$UpperCI, y)
    abline(v = 1, lty = 2, col = "#555555")
    axis(2, at = y, labels = paste("Omit", loo$Study), las = 1, tick = TRUE, cex.axis = 0.8)
    mtext("Each row is a REML random-effects estimate after omitting the named study.", side = 1, line = 3.7, cex = 0.72)
}
funnel_plot <- function (d, m, title, xlab, file, note = TRUE)
{
    jpeg(file, width = 8, height = 7, units = "in", res = 300, quality = 100)
    on.exit(dev.off(), add = TRUE)
    par(mar = c(5, 5, 3.3, 1.2), family = "sans", las = 1)
    ym <- max(d$sei) * 1.08
    xc <- c(d$yi, m$mu - 1.96 * ym, m$mu + 1.96 * ym)
    pad <- diff(range(xc)) * 0.08
    plot(d$yi, d$sei, xlim = range(xc) + c(-pad, pad), ylim = c(ym, 0), pch = 21, bg = if (grepl("hazard", tolower(xlab)))
        "#2F80C0"
    else "#D0D0D0", col = if (grepl("hazard", tolower(xlab)))
        "#004C99"
    else "black", cex = 1.15, xlab = xlab, ylab = "Standard error", main = title, bty = "l")
    s <- seq(0, ym, length.out = 200)
    lines(m$mu - 1.96 * s, s, lty = 2, col = "#707070")
    lines(m$mu + 1.96 * s, s, lty = 2, col = "#707070")
    abline(v = m$mu, col = if (grepl("hazard", tolower(xlab)))
        "#D73027"
    else "#333333", lty = if (grepl("hazard", tolower(xlab)))
        1
    else 3, lwd = 1.2)
    if (note) {
        if (nrow(d) < 10)
            mtext(sprintf("Exploratory only; Egger test not performed (k = %d < 10).", nrow(d)), side = 1, line = 3.7, cex = 0.78)
    }
}
forest_binary(d,m,paste0('High BMI vs Low BMI — ',"Grade 3 or higher immune-related adverse events",' — ',adjustment,' estimates'),"OR >1 indicates greater odds of the event in the high-BMI group.",'forest.jpg')
if(nrow(d)>=3){loo<-leave_one_out(d);sensitivity_binary(loo,paste0("Grade 3 or higher immune-related adverse events",' — ',adjustment,' leave-one-out analysis'),'sensitivity_leave_one_out.jpg')}
if(nrow(d)>=3)funnel_plot(d,m,paste0("Grade 3 or higher immune-related adverse events",' — ',adjustment,' funnel plot'),'Log odds ratio','funnel.jpg',TRUE)
```

**Supplementary Code S11.**BMI categories and OS — adjusted estimates

```
# Analysis: 11_BMI_categories_OS_adjusted
# All analysis data are embedded. Running this file creates figures only.
rm(list = ls())
options(stringsAsFactors = FALSE, scipen = 999)
analysis_id <- "11_BMI_categories_OS_adjusted"
script_arg <- grep('^--file=', commandArgs(trailingOnly = FALSE), value = TRUE)
script_file <- if (length(script_arg)) sub('^--file=', '', script_arg[1]) else NA_character_
if ((is.na(script_file) || !nzchar(script_file)) && interactive() && requireNamespace('rstudioapi', quietly = TRUE)) {
  p <- tryCatch(rstudioapi::getSourceEditorContext()$path, error = function(e) '')
  if (nzchar(p)) script_file <- p
}
script_dir <- if (!is.na(script_file) && nzchar(script_file)) dirname(normalizePath(script_file, winslash='/', mustWork=TRUE)) else normalizePath(getwd(), winslash='/', mustWork=TRUE)
output_dir <- file.path(script_dir, paste0(analysis_id, '_output'))
dir.create(output_dir, recursive=TRUE, showWarnings=FALSE)
setwd(output_dir)
.libPaths(unique(c(file.path(Sys.getenv('USERPROFILE'), 'R-library'), .libPaths())))
embedded_input <- structure(list(ContrastOrder = c(1, 1, 1, 1, 1, 1, 1, 1, 1, 1,
1, 1, 2, 2, 2, 2, 2, 2, 2, 2, 2, 2, 2, 2, 3, 3, 3, 3, 3, 3, 4,
4, 4, 4, 4, 4, 4, 4, 4, 4, 4, 4), Contrast = c("Overweight vs normal weight",
"Overweight vs normal weight", "Overweight vs normal weight",
"Overweight vs normal weight", "Overweight vs normal weight",
"Overweight vs normal weight", "Overweight vs normal weight",
"Overweight vs normal weight", "Overweight vs normal weight",
"Overweight vs normal weight", "Overweight vs normal weight",
"Overweight vs normal weight", "Obese vs normal weight", "Obese vs normal weight",
"Obese vs normal weight", "Obese vs normal weight", "Obese vs normal weight",
"Obese vs normal weight", "Obese vs normal weight", "Obese vs normal weight",
"Obese vs normal weight", "Obese vs normal weight", "Obese vs normal weight",
"Obese vs normal weight", "Underweight vs normal weight", "Underweight vs normal weight",
"Underweight vs normal weight", "Underweight vs normal weight",
"Underweight vs normal weight", "Underweight vs normal weight",
"Obese/Overweight vs normal/underweight", "Obese/Overweight vs normal/underweight",
"Obese/Overweight vs normal/underweight", "Obese/Overweight vs normal/underweight",
"Obese/Overweight vs normal/underweight", "Obese/Overweight vs normal/underweight",
"Obese/Overweight vs normal/underweight", "Obese/Overweight vs normal/underweight",
"Obese/Overweight vs normal/underweight", "Obese/Overweight vs normal/underweight",
"Obese/Overweight vs normal/underweight", "Obese/Overweight vs normal/underweight"
), Study = c("McQuade 2018 (1)", "McQuade 2018 (2)", "Cortellini 2020",
"Antoun 2023 (1)", "Antoun 2023 (2)", "Wang 2022", "Zhang 2023",
"Vithayathil 2023", "Llerena 2024", "Gomez-Banoy 2024", "Nie 2024 (1)",
"Nie 2024 (2)", "McQuade 2018 (1)", "McQuade 2018 (2)", "Cortellini 2020",
"Antoun 2023 (1)", "Antoun 2023 (2)", "Wang 2022", "Zhang 2023",
"Vithayathil 2023", "Gomez-Banoy 2024", "Nie 2024 (1)", "Nie 2024 (2)",
"Mastrolonardo 2025", "Cortellini 2020", "Antoun 2023 (1)", "Antoun 2023 (2)",
"Zhang 2023", "Vithayathil 2023", "Llerena 2024", "Qi 2019",
"Cortellini 2019", "Ahmed 2021", "Collet 2021", "Akce 2021",
"Esposito 2021", "Zhang 2021", "Ged 2022", "Vithayathil 2023",
"Santoni 2023", "Alam 2024", "Wang 2024"), Effect = c(0.7, 0.78,
0.97, 0.8, 1.38, 1.65, 0.229, 0.73, 0.95, 0.64, 0.94, 1.39, 0.54,
0.72, 0.7, 0.85, 1.54, 0.56, 0.947, 0.83, 0.64, 0.91, 0.98, 0.7,
1.21995852141027, 0.529941706412295, 1.16959064327485, 6.66666666666667,
2.17391304347826, 1.02040816326531, 0.496, 0.49, 0.96, 0.75,
0.44, 0.87, 0.62, 0.75, 0.72, 0.68, 0.9, 0.22), LowerCI = c(0.48,
0.52, 0.77, 0.54, 0.89, 0.95, 0.028, 0.38, 0.69, 0.47, 0.8, 0.81,
0.34, 0.48, 0.49, 0.47, 0.73, 0.07, 0.089, 0.42, 0.48, 0.76,
0.51, 0.67, 0.739973360959006, 0.260010400416017, 0.78003120124805,
1.5625, 0.719424460431655, 0.78125, 0.26, 0.38, 0.65, 0.51, 0.18,
0.55, 0.44, 0.5, 0.42, 0.48, 0.49, 0.11), UpperCI = c(1.03, 1.17,
1.22, 1.16, 2.14, 2.86, 1.887, 1.4, 1.32, 0.89, 1.1, 2.39, 0.86,
1.06, 0.99, 1.56, 3.28, 4.26, 10.047, 1.64, 0.87, 1.09, 1.86,
0.73, 2.01005025125628, 1.02040816326531, 1.76056338028169, 33.3333333333333,
6.66666666666667, 1.33333333333333, 0.945, 0.64, 1.42, 1.1, 1.09,
1.36, 0.87, 1.12, 1.23, 0.95, 1.67, 0.45)), row.names = c(NA,
-42L), class = "data.frame")


reml_meta <- function (yi, sei)
{
    keep <- is.finite(yi) & is.finite(sei) & sei > 0
    yi <- yi[keep]
    sei <- sei[keep]
    vi <- sei^2
    k <- length(yi)
    if (!k)
        stop("No valid estimates.")
    if (k == 1L) {
        z <- qnorm(0.975)
        return(list(k = 1L, mu = yi, se_mu = sei, ci_low = yi - z * sei, ci_high = yi + z * sei, p = 2 * pnorm(abs(yi/sei), lower.tail = FALSE), tau2 = NA_real_, Q = NA_real_, df_Q = 0L, Q_p = NA_real_, I2 = NA_real_, weights = 100))
    }
    wf <- 1/vi
    muf <- sum(wf * yi)/sum(wf)
    Q <- sum(wf * (yi - muf)^2)
    dfQ <- k - 1
    I2 <- if (Q > 0)
        max(0, (Q - dfQ)/Q) * 100
    else 0
    obj <- function(tau2) {
        w <- 1/(vi + tau2)
        mu <- sum(w * yi)/sum(w)
        0.5 * (sum(log(vi + tau2)) + log(sum(w)) + sum(w * (yi - mu)^2))
    }
    upper <- max(1, var(yi) * 10, max(vi) * 10)
    opt <- optimize(obj, c(0, upper), tol = 1e-12)
    tau2 <- if (opt$minimum < 1e-10)
        0
    else opt$minimum
    w <- 1/(vi + tau2)
    mu <- sum(w * yi)/sum(w)
    se <- sqrt(1/sum(w))
    ci <- mu + c(-1, 1) * qnorm(0.975) * se
    list(k = k, mu = mu, se_mu = se, ci_low = ci[1], ci_high = ci[2], p = 2 * pnorm(abs(mu/se), lower.tail = FALSE), tau2 = tau2, Q = Q, df_Q = dfQ, Q_p = pchisq(Q, dfQ, lower.tail = FALSE), I2 = I2, weights = w/sum(w) * 100)
}
prepare_effects <- function (d, effect_col = "Effect")
{
    d[[effect_col]] <- as.numeric(d[[effect_col]])
    d$LowerCI <- as.numeric(d$LowerCI)
    d$UpperCI <- as.numeric(d$UpperCI)
    ok <- is.finite(d[[effect_col]]) & is.finite(d$LowerCI) & is.finite(d$UpperCI) & d[[effect_col]] > 0 & d$LowerCI > 0 & d$UpperCI > d$LowerCI
    d <- d[ok, , drop = FALSE]
    d$Effect <- d[[effect_col]]
    d$yi <- log(d$Effect)
    d$sei <- (log(d$UpperCI) - log(d$LowerCI))/(2 * qnorm(0.975))
    d
}
fmt_p <- function (p)
if (!is.finite(p)) "NR" else if (p < 1e-04) "< 0.0001" else paste0("= ", formatC(p, format = "f", digits = 4))
funnel_plot <- function (d, m, title, xlab, file, note = TRUE)
{
    jpeg(file, width = 8, height = 7, units = "in", res = 300, quality = 100)
    on.exit(dev.off(), add = TRUE)
    par(mar = c(5, 5, 3.3, 1.2), family = "sans", las = 1)
    ym <- max(d$sei) * 1.08
    xc <- c(d$yi, m$mu - 1.96 * ym, m$mu + 1.96 * ym)
    pad <- diff(range(xc)) * 0.08
    plot(d$yi, d$sei, xlim = range(xc) + c(-pad, pad), ylim = c(ym, 0), pch = 21, bg = if (grepl("hazard", tolower(xlab)))
        "#2F80C0"
    else "#D0D0D0", col = if (grepl("hazard", tolower(xlab)))
        "#004C99"
    else "black", cex = 1.15, xlab = xlab, ylab = "Standard error", main = title, bty = "l")
    s <- seq(0, ym, length.out = 200)
    lines(m$mu - 1.96 * s, s, lty = 2, col = "#707070")
    lines(m$mu + 1.96 * s, s, lty = 2, col = "#707070")
    abline(v = m$mu, col = if (grepl("hazard", tolower(xlab)))
        "#D73027"
    else "#333333", lty = if (grepl("hazard", tolower(xlab)))
        1
    else 3, lwd = 1.2)
    if (note) {
        if (nrow(d) < 10)
            mtext(sprintf("Exploratory only; Egger test not performed (k = %d < 10).", nrow(d)), side = 1, line = 3.7, cex = 0.78)
    }
}
category_forest <- function (d, endpoint, file)
{
    if (!requireNamespace("metafor", quietly = TRUE))
        stop("Package 'metafor' is required.")
    labels <- unique(d$Contrast[order(d$ContrastOrder)])
    d$yi <- log(d$Effect)
    d$sei <- (log(d$UpperCI) - log(d$LowerCI))/(2 * 1.96)
    d$vi <- d$sei^2
    models <- lapply(labels, function(g) metafor::rma.uni(yi, vi, data = d[d$Contrast == g, ], method = "REML", test = "knha"))
    names(models) <- labels
    rows <- list()
    for (g in labels) {
        rows[[length(rows) + 1]] <- data.frame(type = "title", g = g, i = NA)
        for (i in which(d$Contrast == g)) rows[[length(rows) + 1]] <- data.frame(type = "study", g = g, i = i)
        rows[[length(rows) + 1]] <- data.frame(type = "sum", g = g, i = NA)
        rows[[length(rows) + 1]] <- data.frame(type = "het", g = g, i = NA)
        rows[[length(rows) + 1]] <- data.frame(type = "space", g = g, i = NA)
    }
    rp <- do.call(rbind, rows)
    rp$y <- rev(seq_len(nrow(rp))) + 3
    h <- if (endpoint == "OS")
        19.3
    else 16.2
    jpeg(file, width = 11, height = h, units = "in", res = 300, quality = 100)
    on.exit(dev.off(), add = TRUE)
    par(mar = c(2.4, 0.6, 2, 0.6), family = "sans", xpd = NA)
    plot.new()
    plot.window(xlim = c(0, 1), ylim = c(0, max(rp$y) + 4.8))
    xs <- 0.025
    xln <- 0.315
    xse <- 0.4
    pl <- 0.475
    pr <- 0.715
    xhr <- 0.755
    xci <- 0.825
    xw <- 0.965
    map <- function(v) pl + (log10(v) + 1)/2 * (pr - pl)
    maxw <- 0
    for (g in labels) {
        z <- d[d$Contrast == g, ]
        m <- models[[g]]
        w <- as.numeric(weights(m))
        d$w[d$Contrast == g] <- 100 * w/sum(w)
        maxw <- max(maxw, d$w[d$Contrast == g])
    }
    title_txt <- if (endpoint == "OS")
        "Association between BMI categories and overall survival"
    else "Association between BMI categories and progression-free survival"
    text(0.5, max(rp$y) + 3.9, title_txt, font = 2, cex = 1.02)
    hy <- max(rp$y) + 2.2
    text(xs, hy, "Study", adj = 0, font = 2, cex = 0.74)
    text(xln, hy, "lnHR", adj = 1, font = 2, cex = 0.74)
    text(xse, hy, "SE(lnHR)", adj = 1, font = 2, cex = 0.74)
    text((pl + pr)/2, hy, "Hazard Ratio", font = 2, cex = 0.74)
    text(xhr, hy, "HR", adj = 1, font = 2, cex = 0.74)
    text(xci, hy, "95% CI", adj = 0, font = 2, cex = 0.74)
    text(xw, hy, "Weight", adj = 1, font = 2, cex = 0.74)
    text(xw, hy - 0.55, "(within comparison)", adj = 1, cex = 0.55)
    segments(map(1), 2.7, map(1), max(rp$y) + 1, lwd = 0.8)
    num <- setNames(seq_along(labels), labels)
    for (r in seq_len(nrow(rp))) {
        z <- rp[r, ]
        y <- z$y
        if (z$type == "title")
            text(xs, y, paste0(num[z$g], ". ", z$g), adj = 0, font = 2, cex = 0.76)
        if (z$type == "study") {
            i <- z$i
            segments(map(max(0.1, d$LowerCI[i])), y, map(min(10, d$UpperCI[i])), y, col = "#333333", lwd = 0.8)
            if (d$LowerCI[i] < 0.1)
                arrows(map(0.12), y, map(0.1), y, length = 0.04)
            if (d$UpperCI[i] > 10)
                arrows(map(8.5), y, map(10), y, length = 0.04)
            points(map(d$Effect[i]), y, pch = 15, col = "#A7C1D0", cex = 0.55 + 1.1 * sqrt(d$w[i]/maxw))
            text(xs, y, d$Study[i], adj = 0, cex = 0.67)
            text(xln, y, formatC(d$yi[i], format = "f", digits = 4), adj = 1, cex = 0.64)
            text(xse, y, formatC(d$sei[i], format = "f", digits = 4), adj = 1, cex = 0.64)
            text(xhr, y, sprintf("%.2f", d$Effect[i]), adj = 1, cex = 0.67)
            text(xci, y, sprintf("[%.2f; %.2f]", d$LowerCI[i], d$UpperCI[i]), adj = 0, cex = 0.67)
            text(xw, y, sprintf("%.2f%%", d$w[i]), adj = 1, cex = 0.67)
        }
        if (z$type == "sum") {
            m <- models[[z$g]]
            est <- exp(as.numeric(m$b))
            lb <- exp(m$ci.lb)
            ub <- exp(m$ci.ub)
            polygon(c(map(lb), map(est), map(ub), map(est)), c(y, y + 0.34, y, y - 0.34), col = "#E25353", border = "#333333")
            text(xs, y, "Random-effects model", adj = 0, font = 2, cex = 0.68)
            text(xhr, y, sprintf("%.2f", est), adj = 1, font = 2, cex = 0.68)
            text(xci, y, sprintf("[%.2f; %.2f]", lb, ub), adj = 0, font = 2, cex = 0.68)
            text(xw, y, "100.00%", adj = 1, font = 2, cex = 0.68)
        }
        if (z$type == "het") {
            m <- models[[z$g]]
            text(xs, y, sprintf("Heterogeneity: I² = %.1f%%, τ² = %.4f, p %s", m$I2, m$tau2, fmt_p(m$QEp)), adj = 0, cex = 0.64)
        }
    }
    ay <- 2.8
    segments(pl, ay, pr, ay)
    for (t in c(0.1, 0.2, 0.5, 1, 2, 5, 10)) {
        segments(map(t), ay, map(t), ay - 0.28)
        text(map(t), ay - 0.52, t, cex = 0.66)
    }
    text((pl + pr)/2, ay - 1.05, "Hazard Ratio", cex = 0.7)
}
category_sensitivity <- function (d, label, file)
{
    if (!requireNamespace("metafor", quietly = TRUE))
        stop("Package 'metafor' is required.")
    d$yi <- log(d$Effect)
    d$sei <- (log(d$UpperCI) - log(d$LowerCI))/(2 * 1.96)
    m <- metafor::rma.uni(d$yi, sei = d$sei, method = "REML", test = "knha")
    loo <- metafor::leave1out(m)
    k <- nrow(d)
    est <- exp(loo$estimate)
    lb <- exp(loo$ci.lb)
    ub <- exp(loo$ci.ub)
    full <- exp(as.numeric(m$b))
    fl <- exp(m$ci.lb)
    fu <- exp(m$ci.ub)
    num <- unique(d$ContrastOrder)[1]
    jpeg(file, width = 10, height = max(6.2, 4.24 + 0.385 * k), units = "in", res = 300, quality = 100)
    on.exit(dev.off(), add = TRUE)
    par(mar = c(3.9, 0.6, 2.7, 0.6), family = "sans", xpd = NA)
    plot.new()
    plot.window(xlim = c(0, 1), ylim = c(-1.9, k + 3.1))
    xs <- 0.025
    pl <- 0.46
    pr <- 0.75
    xhr <- 0.81
    xci <- 0.9
    map <- function(v) pl + (log(v) - log(0.1))/(log(10) - log(0.1)) * (pr - pl)
    y <- rev(seq_len(k)) + 1.1
    for (i in seq_len(k)) if (i%%2 == 0)
        rect(0.015, y[i] - 0.45, 0.985, y[i] + 0.45, col = "#F4F4F4", border = NA)
    text(xs, k + 2.25, "Study omitted", adj = 0, font = 2, cex = 0.78)
    text((pl + pr)/2, k + 2.25, "Pooled Hazard Ratio", font = 2, cex = 0.78)
    text(xhr, k + 2.25, "HR", adj = 1, font = 2, cex = 0.78)
    text(xci, k + 2.25, "95% CI", font = 2, cex = 0.78)
    segments(map(1), -0.2, map(1), k + 1.5)
    segments(map(full), -0.2, map(full), k + 1.5, lty = 3, col = "red")
    for (i in seq_len(k)) {
        segments(map(max(0.1, lb[i])), y[i], map(min(10, ub[i])), y[i], lwd = 0.8)
        points(map(est[i]), y[i], pch = 15, col = "#AFAFAF", cex = 0.9)
        text(xs, y[i], paste("Omit", d$Study[i]), adj = 0, cex = 0.7)
        text(xhr, y[i], sprintf("%.2f", est[i]), adj = 1, cex = 0.7)
        text(xci, y[i], sprintf("[%.2f; %.2f]", lb[i], ub[i]), cex = 0.7)
    }
    py <- 0.05
    polygon(c(map(fl), map(full), map(fu), map(full)), c(py, py + 0.28, py, py - 0.28), col = "red", border = "black")
    text(xs, py, "Full random-effects model", adj = 0, font = 2, cex = 0.72)
    text(xhr, py, sprintf("%.2f", full), adj = 1, font = 2, cex = 0.72)
    text(xci, py, sprintf("[%.2f; %.2f]", fl, fu), font = 2, cex = 0.72)
    ay <- -0.65
    segments(pl, ay, pr, ay)
    for (t in c(0.1, 0.2, 0.5, 1, 2, 5, 10)) {
        segments(map(t), ay, map(t), ay - 0.15)
        text(map(t), ay - 0.35, t, cex = 0.66)
    }
    text((pl + pr)/2, ay - 0.72, "Hazard Ratio", cex = 0.7)
    mtext(paste0("Leave-one-out sensitivity analysis: ", num, ". ", label), side = 3, line = 0.7, font = 2, cex = 0.86)
    text(0.5, -1.65, sprintf("Dashed red line = full-model pooled HR; REML with Knapp-Hartung; full-model I2=%.2f%%", m$I2), cex = 0.6, col = "#555555")
}
category_funnel <- function (d, label, file)
{
    d$yi <- log(d$Effect)
    d$sei <- (log(d$UpperCI) - log(d$LowerCI))/(2 * 1.96)
    m <- reml_meta(d$yi, d$sei)
    k <- nrow(d)
    ym <- max(d$sei) * 1.08
    s <- seq(0, ym, length.out = 200)
    xl <- range(c(d$yi, m$mu - 1.96 * ym, m$mu + 1.96 * ym))
    pad <- diff(xl) * 0.08
    num <- unique(d$ContrastOrder)[1]
    ep <- if (k >= 10) {
        fit <- metafor::rma.uni(yi = d$yi, sei = d$sei, method = "REML")
        eg <- metafor::regtest(fit, model = "rma", predictor = "sei")
        sprintf("k=%d; Egger regression p=%.3f", k, eg$pval)
    }
    else sprintf("k=%d; Egger regression not performed", k)
    jpeg(file, width = 8, height = 7, units = "in", res = 300, quality = 100)
    on.exit(dev.off(), add = TRUE)
    par(mar = c(5, 5, 4.2, 1.2), family = "sans", las = 1)
    plot(d$yi, d$sei, xlim = xl + c(-pad, pad), ylim = c(ym, 0), pch = 15, col = "#B5B5B5", cex = 1.15, xlab = "Log Hazard Ratio", ylab = "Standard Error", main = paste0("Funnel plot:  ", num, ". ", label), bty = "l")
    polygon(c(m$mu - 1.96 * ym, m$mu, m$mu + 1.96 * ym), c(ym, 0, ym), col = "#EFEFEF", border = NA)
    lines(m$mu - 1.96 * s, s, lty = 2, col = "#777777")
    lines(m$mu + 1.96 * s, s, lty = 2, col = "#777777")
    abline(v = m$mu, col = "red", lwd = 1.4)
    points(d$yi, d$sei, pch = 15, col = "#B5B5B5", cex = 1.15)
    mtext(ep, side = 3, line = 0.65, cex = 0.78, col = "#444444")
}
d<-prepare_effects(as.data.frame(embedded_input))
category_forest(d,"OS",'forest.jpg')
for(g in unique(d$Contrast[order(d$ContrastOrder)])){z<-d[d$Contrast==g,,drop=FALSE];stem<-gsub('[^A-Za-z0-9]+','_',g);if(nrow(z)>=3)category_sensitivity(z,g,paste0('sensitivity_',stem,'.jpg'));category_funnel(z,g,paste0('funnel_',stem,'.jpg'))}
```

**Supplementary Code S12.**BMI categories and OS — unadjusted estimates

```
# Analysis: 12_BMI_categories_OS_unadjusted
# All analysis data are embedded. Running this file creates figures only.
rm(list = ls())
options(stringsAsFactors = FALSE, scipen = 999)
analysis_id <- "12_BMI_categories_OS_unadjusted"
script_arg <- grep('^--file=', commandArgs(trailingOnly = FALSE), value = TRUE)
script_file <- if (length(script_arg)) sub('^--file=', '', script_arg[1]) else NA_character_
if ((is.na(script_file) || !nzchar(script_file)) && interactive() && requireNamespace('rstudioapi', quietly = TRUE)) {
  p <- tryCatch(rstudioapi::getSourceEditorContext()$path, error = function(e) '')
  if (nzchar(p)) script_file <- p
}
script_dir <- if (!is.na(script_file) && nzchar(script_file)) dirname(normalizePath(script_file, winslash='/', mustWork=TRUE)) else normalizePath(getwd(), winslash='/', mustWork=TRUE)
output_dir <- file.path(script_dir, paste0(analysis_id, '_output'))
dir.create(output_dir, recursive=TRUE, showWarnings=FALSE)
setwd(output_dir)
.libPaths(unique(c(file.path(Sys.getenv('USERPROFILE'), 'R-library'), .libPaths())))
embedded_input <- structure(list(ContrastOrder = c(1, 1, 1, 1, 1, 2, 2, 2, 2, 2,
3, 3, 3, 4, 4, 4, 4, 4, 4, 4, 4, 4, 4, 4), Contrast = c("Overweight vs normal weight",
"Overweight vs normal weight", "Overweight vs normal weight",
"Overweight vs normal weight", "Overweight vs normal weight",
"Obese vs normal weight", "Obese vs normal weight", "Obese vs normal weight",
"Obese vs normal weight", "Obese vs normal weight", "Underweight vs normal weight",
"Underweight vs normal weight", "Underweight vs normal weight",
"Obese/Overweight vs normal/underweight", "Obese/Overweight vs normal/underweight",
"Obese/Overweight vs normal/underweight", "Obese/Overweight vs normal/underweight",
"Obese/Overweight vs normal/underweight", "Obese/Overweight vs normal/underweight",
"Obese/Overweight vs normal/underweight", "Obese/Overweight vs normal/underweight",
"Obese/Overweight vs normal/underweight", "Obese/Overweight vs normal/underweight",
"Obese/Overweight vs normal/underweight"), Study = c("Labadie 2019",
"Kichenadasse 2019", "Åžen 2024", "Demir 2025", "Wang 2024",
"Labadie 2019", "Kichenadasse 2019", "Åžen 2024", "Demir 2025",
"Wang 2024", "Cortellini 2019", "Labadie 2019", "Ichihara 2020",
"Qi 2019", "Ahmed 2021", "Collet 2021", "Esposito 2021", "Ged 2022",
"Tateishi 2022", "Vithayathil 2023", "Santoni 2023", "Wang 2024",
"Åžen 2024", "Demir 2025"), Effect = c(0.38, 0.81, 1.22, 1.08,
0.48, 0.28, 0.64, 0.84, 1.01, 0.28, 3.03030303030303, 2.02839756592292,
1.47058823529412, 0.496, 0.77, 0.63, 0.8, 0.66, 1.03, 1, 0.52,
0.39, 1.06, 1.06), LowerCI = c(0.15, 0.68, 0.69, 0.8, 0.25, 0.09,
0.51, 0.45, 0.64, 0.14, 2.08333333333333, 0.260010400416017,
1.0752688172043, 0.261, 0.57, 0.44, 0.53, 0.45, 0.68, 0.6, 0.38,
0.21, 0.69, 0.8), UpperCI = c(0.97, 0.95, 1.82, 1.46, 0.9, 0.91,
0.81, 1.56, 1.56, 0.57, 4.34782608695652, 15.8730158730159, 2,
0.943, 1.04, 0.92, 1.21, 0.97, 1.54, 1.64, 0.73, 0.7, 1.64, 1.4
)), row.names = c(NA, -24L), class = "data.frame")


reml_meta <- function (yi, sei)
{
    keep <- is.finite(yi) & is.finite(sei) & sei > 0
    yi <- yi[keep]
    sei <- sei[keep]
    vi <- sei^2
    k <- length(yi)
    if (!k)
        stop("No valid estimates.")
    if (k == 1L) {
        z <- qnorm(0.975)
        return(list(k = 1L, mu = yi, se_mu = sei, ci_low = yi - z * sei, ci_high = yi + z * sei, p = 2 * pnorm(abs(yi/sei), lower.tail = FALSE), tau2 = NA_real_, Q = NA_real_, df_Q = 0L, Q_p = NA_real_, I2 = NA_real_, weights = 100))
    }
    wf <- 1/vi
    muf <- sum(wf * yi)/sum(wf)
    Q <- sum(wf * (yi - muf)^2)
    dfQ <- k - 1
    I2 <- if (Q > 0)
        max(0, (Q - dfQ)/Q) * 100
    else 0
    obj <- function(tau2) {
        w <- 1/(vi + tau2)
        mu <- sum(w * yi)/sum(w)
        0.5 * (sum(log(vi + tau2)) + log(sum(w)) + sum(w * (yi - mu)^2))
    }
    upper <- max(1, var(yi) * 10, max(vi) * 10)
    opt <- optimize(obj, c(0, upper), tol = 1e-12)
    tau2 <- if (opt$minimum < 1e-10)
        0
    else opt$minimum
    w <- 1/(vi + tau2)
    mu <- sum(w * yi)/sum(w)
    se <- sqrt(1/sum(w))
    ci <- mu + c(-1, 1) * qnorm(0.975) * se
    list(k = k, mu = mu, se_mu = se, ci_low = ci[1], ci_high = ci[2], p = 2 * pnorm(abs(mu/se), lower.tail = FALSE), tau2 = tau2, Q = Q, df_Q = dfQ, Q_p = pchisq(Q, dfQ, lower.tail = FALSE), I2 = I2, weights = w/sum(w) * 100)
}
prepare_effects <- function (d, effect_col = "Effect")
{
    d[[effect_col]] <- as.numeric(d[[effect_col]])
    d$LowerCI <- as.numeric(d$LowerCI)
    d$UpperCI <- as.numeric(d$UpperCI)
    ok <- is.finite(d[[effect_col]]) & is.finite(d$LowerCI) & is.finite(d$UpperCI) & d[[effect_col]] > 0 & d$LowerCI > 0 & d$UpperCI > d$LowerCI
    d <- d[ok, , drop = FALSE]
    d$Effect <- d[[effect_col]]
    d$yi <- log(d$Effect)
    d$sei <- (log(d$UpperCI) - log(d$LowerCI))/(2 * qnorm(0.975))
    d
}
fmt_p <- function (p)
if (!is.finite(p)) "NR" else if (p < 1e-04) "< 0.0001" else paste0("= ", formatC(p, format = "f", digits = 4))
funnel_plot <- function (d, m, title, xlab, file, note = TRUE)
{
    jpeg(file, width = 8, height = 7, units = "in", res = 300, quality = 100)
    on.exit(dev.off(), add = TRUE)
    par(mar = c(5, 5, 3.3, 1.2), family = "sans", las = 1)
    ym <- max(d$sei) * 1.08
    xc <- c(d$yi, m$mu - 1.96 * ym, m$mu + 1.96 * ym)
    pad <- diff(range(xc)) * 0.08
    plot(d$yi, d$sei, xlim = range(xc) + c(-pad, pad), ylim = c(ym, 0), pch = 21, bg = if (grepl("hazard", tolower(xlab)))
        "#2F80C0"
    else "#D0D0D0", col = if (grepl("hazard", tolower(xlab)))
        "#004C99"
    else "black", cex = 1.15, xlab = xlab, ylab = "Standard error", main = title, bty = "l")
    s <- seq(0, ym, length.out = 200)
    lines(m$mu - 1.96 * s, s, lty = 2, col = "#707070")
    lines(m$mu + 1.96 * s, s, lty = 2, col = "#707070")
    abline(v = m$mu, col = if (grepl("hazard", tolower(xlab)))
        "#D73027"
    else "#333333", lty = if (grepl("hazard", tolower(xlab)))
        1
    else 3, lwd = 1.2)
    if (note) {
        if (nrow(d) < 10)
            mtext(sprintf("Exploratory only; Egger test not performed (k = %d < 10).", nrow(d)), side = 1, line = 3.7, cex = 0.78)
    }
}
category_forest <- function (d, endpoint, file)
{
    if (!requireNamespace("metafor", quietly = TRUE))
        stop("Package 'metafor' is required.")
    labels <- unique(d$Contrast[order(d$ContrastOrder)])
    d$yi <- log(d$Effect)
    d$sei <- (log(d$UpperCI) - log(d$LowerCI))/(2 * 1.96)
    d$vi <- d$sei^2
    models <- lapply(labels, function(g) metafor::rma.uni(yi, vi, data = d[d$Contrast == g, ], method = "REML", test = "knha"))
    names(models) <- labels
    rows <- list()
    for (g in labels) {
        rows[[length(rows) + 1]] <- data.frame(type = "title", g = g, i = NA)
        for (i in which(d$Contrast == g)) rows[[length(rows) + 1]] <- data.frame(type = "study", g = g, i = i)
        rows[[length(rows) + 1]] <- data.frame(type = "sum", g = g, i = NA)
        rows[[length(rows) + 1]] <- data.frame(type = "het", g = g, i = NA)
        rows[[length(rows) + 1]] <- data.frame(type = "space", g = g, i = NA)
    }
    rp <- do.call(rbind, rows)
    rp$y <- rev(seq_len(nrow(rp))) + 3
    h <- if (endpoint == "OS")
        19.3
    else 16.2
    jpeg(file, width = 11, height = h, units = "in", res = 300, quality = 100)
    on.exit(dev.off(), add = TRUE)
    par(mar = c(2.4, 0.6, 2, 0.6), family = "sans", xpd = NA)
    plot.new()
    plot.window(xlim = c(0, 1), ylim = c(0, max(rp$y) + 4.8))
    xs <- 0.025
    xln <- 0.315
    xse <- 0.4
    pl <- 0.475
    pr <- 0.715
    xhr <- 0.755
    xci <- 0.825
    xw <- 0.965
    map <- function(v) pl + (log10(v) + 1)/2 * (pr - pl)
    maxw <- 0
    for (g in labels) {
        z <- d[d$Contrast == g, ]
        m <- models[[g]]
        w <- as.numeric(weights(m))
        d$w[d$Contrast == g] <- 100 * w/sum(w)
        maxw <- max(maxw, d$w[d$Contrast == g])
    }
    title_txt <- if (endpoint == "OS")
        "Association between BMI categories and overall survival"
    else "Association between BMI categories and progression-free survival"
    text(0.5, max(rp$y) + 3.9, title_txt, font = 2, cex = 1.02)
    hy <- max(rp$y) + 2.2
    text(xs, hy, "Study", adj = 0, font = 2, cex = 0.74)
    text(xln, hy, "lnHR", adj = 1, font = 2, cex = 0.74)
    text(xse, hy, "SE(lnHR)", adj = 1, font = 2, cex = 0.74)
    text((pl + pr)/2, hy, "Hazard Ratio", font = 2, cex = 0.74)
    text(xhr, hy, "HR", adj = 1, font = 2, cex = 0.74)
    text(xci, hy, "95% CI", adj = 0, font = 2, cex = 0.74)
    text(xw, hy, "Weight", adj = 1, font = 2, cex = 0.74)
    text(xw, hy - 0.55, "(within comparison)", adj = 1, cex = 0.55)
    segments(map(1), 2.7, map(1), max(rp$y) + 1, lwd = 0.8)
    num <- setNames(seq_along(labels), labels)
    for (r in seq_len(nrow(rp))) {
        z <- rp[r, ]
        y <- z$y
        if (z$type == "title")
            text(xs, y, paste0(num[z$g], ". ", z$g), adj = 0, font = 2, cex = 0.76)
        if (z$type == "study") {
            i <- z$i
            segments(map(max(0.1, d$LowerCI[i])), y, map(min(10, d$UpperCI[i])), y, col = "#333333", lwd = 0.8)
            if (d$LowerCI[i] < 0.1)
                arrows(map(0.12), y, map(0.1), y, length = 0.04)
            if (d$UpperCI[i] > 10)
                arrows(map(8.5), y, map(10), y, length = 0.04)
            points(map(d$Effect[i]), y, pch = 15, col = "#A7C1D0", cex = 0.55 + 1.1 * sqrt(d$w[i]/maxw))
            text(xs, y, d$Study[i], adj = 0, cex = 0.67)
            text(xln, y, formatC(d$yi[i], format = "f", digits = 4), adj = 1, cex = 0.64)
            text(xse, y, formatC(d$sei[i], format = "f", digits = 4), adj = 1, cex = 0.64)
            text(xhr, y, sprintf("%.2f", d$Effect[i]), adj = 1, cex = 0.67)
            text(xci, y, sprintf("[%.2f; %.2f]", d$LowerCI[i], d$UpperCI[i]), adj = 0, cex = 0.67)
            text(xw, y, sprintf("%.2f%%", d$w[i]), adj = 1, cex = 0.67)
        }
        if (z$type == "sum") {
            m <- models[[z$g]]
            est <- exp(as.numeric(m$b))
            lb <- exp(m$ci.lb)
            ub <- exp(m$ci.ub)
            polygon(c(map(lb), map(est), map(ub), map(est)), c(y, y + 0.34, y, y - 0.34), col = "#E25353", border = "#333333")
            text(xs, y, "Random-effects model", adj = 0, font = 2, cex = 0.68)
            text(xhr, y, sprintf("%.2f", est), adj = 1, font = 2, cex = 0.68)
            text(xci, y, sprintf("[%.2f; %.2f]", lb, ub), adj = 0, font = 2, cex = 0.68)
            text(xw, y, "100.00%", adj = 1, font = 2, cex = 0.68)
        }
        if (z$type == "het") {
            m <- models[[z$g]]
            text(xs, y, sprintf("Heterogeneity: I² = %.1f%%, τ² = %.4f, p %s", m$I2, m$tau2, fmt_p(m$QEp)), adj = 0, cex = 0.64)
        }
    }
    ay <- 2.8
    segments(pl, ay, pr, ay)
    for (t in c(0.1, 0.2, 0.5, 1, 2, 5, 10)) {
        segments(map(t), ay, map(t), ay - 0.28)
        text(map(t), ay - 0.52, t, cex = 0.66)
    }
    text((pl + pr)/2, ay - 1.05, "Hazard Ratio", cex = 0.7)
}
category_sensitivity <- function (d, label, file)
{
    if (!requireNamespace("metafor", quietly = TRUE))
        stop("Package 'metafor' is required.")
    d$yi <- log(d$Effect)
    d$sei <- (log(d$UpperCI) - log(d$LowerCI))/(2 * 1.96)
    m <- metafor::rma.uni(d$yi, sei = d$sei, method = "REML", test = "knha")
    loo <- metafor::leave1out(m)
    k <- nrow(d)
    est <- exp(loo$estimate)
    lb <- exp(loo$ci.lb)
    ub <- exp(loo$ci.ub)
    full <- exp(as.numeric(m$b))
    fl <- exp(m$ci.lb)
    fu <- exp(m$ci.ub)
    num <- unique(d$ContrastOrder)[1]
    jpeg(file, width = 10, height = max(6.2, 4.24 + 0.385 * k), units = "in", res = 300, quality = 100)
    on.exit(dev.off(), add = TRUE)
    par(mar = c(3.9, 0.6, 2.7, 0.6), family = "sans", xpd = NA)
    plot.new()
    plot.window(xlim = c(0, 1), ylim = c(-1.9, k + 3.1))
    xs <- 0.025
    pl <- 0.46
    pr <- 0.75
    xhr <- 0.81
    xci <- 0.9
    map <- function(v) pl + (log(v) - log(0.1))/(log(10) - log(0.1)) * (pr - pl)
    y <- rev(seq_len(k)) + 1.1
    for (i in seq_len(k)) if (i%%2 == 0)
        rect(0.015, y[i] - 0.45, 0.985, y[i] + 0.45, col = "#F4F4F4", border = NA)
    text(xs, k + 2.25, "Study omitted", adj = 0, font = 2, cex = 0.78)
    text((pl + pr)/2, k + 2.25, "Pooled Hazard Ratio", font = 2, cex = 0.78)
    text(xhr, k + 2.25, "HR", adj = 1, font = 2, cex = 0.78)
    text(xci, k + 2.25, "95% CI", font = 2, cex = 0.78)
    segments(map(1), -0.2, map(1), k + 1.5)
    segments(map(full), -0.2, map(full), k + 1.5, lty = 3, col = "red")
    for (i in seq_len(k)) {
        segments(map(max(0.1, lb[i])), y[i], map(min(10, ub[i])), y[i], lwd = 0.8)
        points(map(est[i]), y[i], pch = 15, col = "#AFAFAF", cex = 0.9)
        text(xs, y[i], paste("Omit", d$Study[i]), adj = 0, cex = 0.7)
        text(xhr, y[i], sprintf("%.2f", est[i]), adj = 1, cex = 0.7)
        text(xci, y[i], sprintf("[%.2f; %.2f]", lb[i], ub[i]), cex = 0.7)
    }
    py <- 0.05
    polygon(c(map(fl), map(full), map(fu), map(full)), c(py, py + 0.28, py, py - 0.28), col = "red", border = "black")
    text(xs, py, "Full random-effects model", adj = 0, font = 2, cex = 0.72)
    text(xhr, py, sprintf("%.2f", full), adj = 1, font = 2, cex = 0.72)
    text(xci, py, sprintf("[%.2f; %.2f]", fl, fu), font = 2, cex = 0.72)
    ay <- -0.65
    segments(pl, ay, pr, ay)
    for (t in c(0.1, 0.2, 0.5, 1, 2, 5, 10)) {
        segments(map(t), ay, map(t), ay - 0.15)
        text(map(t), ay - 0.35, t, cex = 0.66)
    }
    text((pl + pr)/2, ay - 0.72, "Hazard Ratio", cex = 0.7)
    mtext(paste0("Leave-one-out sensitivity analysis: ", num, ". ", label), side = 3, line = 0.7, font = 2, cex = 0.86)
    text(0.5, -1.65, sprintf("Dashed red line = full-model pooled HR; REML with Knapp-Hartung; full-model I2=%.2f%%", m$I2), cex = 0.6, col = "#555555")
}
category_funnel <- function (d, label, file)
{
    d$yi <- log(d$Effect)
    d$sei <- (log(d$UpperCI) - log(d$LowerCI))/(2 * 1.96)
    m <- reml_meta(d$yi, d$sei)
    k <- nrow(d)
    ym <- max(d$sei) * 1.08
    s <- seq(0, ym, length.out = 200)
    xl <- range(c(d$yi, m$mu - 1.96 * ym, m$mu + 1.96 * ym))
    pad <- diff(xl) * 0.08
    num <- unique(d$ContrastOrder)[1]
    ep <- if (k >= 10) {
        fit <- metafor::rma.uni(yi = d$yi, sei = d$sei, method = "REML")
        eg <- metafor::regtest(fit, model = "rma", predictor = "sei")
        sprintf("k=%d; Egger regression p=%.3f", k, eg$pval)
    }
    else sprintf("k=%d; Egger regression not performed", k)
    jpeg(file, width = 8, height = 7, units = "in", res = 300, quality = 100)
    on.exit(dev.off(), add = TRUE)
    par(mar = c(5, 5, 4.2, 1.2), family = "sans", las = 1)
    plot(d$yi, d$sei, xlim = xl + c(-pad, pad), ylim = c(ym, 0), pch = 15, col = "#B5B5B5", cex = 1.15, xlab = "Log Hazard Ratio", ylab = "Standard Error", main = paste0("Funnel plot:  ", num, ". ", label), bty = "l")
    polygon(c(m$mu - 1.96 * ym, m$mu, m$mu + 1.96 * ym), c(ym, 0, ym), col = "#EFEFEF", border = NA)
    lines(m$mu - 1.96 * s, s, lty = 2, col = "#777777")
    lines(m$mu + 1.96 * s, s, lty = 2, col = "#777777")
    abline(v = m$mu, col = "red", lwd = 1.4)
    points(d$yi, d$sei, pch = 15, col = "#B5B5B5", cex = 1.15)
    mtext(ep, side = 3, line = 0.65, cex = 0.78, col = "#444444")
}
d<-prepare_effects(as.data.frame(embedded_input))
category_forest(d,"OS",'forest.jpg')
for(g in unique(d$Contrast[order(d$ContrastOrder)])){z<-d[d$Contrast==g,,drop=FALSE];stem<-gsub('[^A-Za-z0-9]+','_',g);if(nrow(z)>=3)category_sensitivity(z,g,paste0('sensitivity_',stem,'.jpg'));category_funnel(z,g,paste0('funnel_',stem,'.jpg'))}
```

**Supplementary Code S13.**BMI categories and PFS — adjusted estimates

```
# Analysis: 13_BMI_categories_PFS_adjusted
# All analysis data are embedded. Running this file creates figures only.
rm(list = ls())
options(stringsAsFactors = FALSE, scipen = 999)
analysis_id <- "13_BMI_categories_PFS_adjusted"
script_arg <- grep('^--file=', commandArgs(trailingOnly = FALSE), value = TRUE)
script_file <- if (length(script_arg)) sub('^--file=', '', script_arg[1]) else NA_character_
if ((is.na(script_file) || !nzchar(script_file)) && interactive() && requireNamespace('rstudioapi', quietly = TRUE)) {
  p <- tryCatch(rstudioapi::getSourceEditorContext()$path, error = function(e) '')
  if (nzchar(p)) script_file <- p
}
script_dir <- if (!is.na(script_file) && nzchar(script_file)) dirname(normalizePath(script_file, winslash='/', mustWork=TRUE)) else normalizePath(getwd(), winslash='/', mustWork=TRUE)
output_dir <- file.path(script_dir, paste0(analysis_id, '_output'))
dir.create(output_dir, recursive=TRUE, showWarnings=FALSE)
setwd(output_dir)
.libPaths(unique(c(file.path(Sys.getenv('USERPROFILE'), 'R-library'), .libPaths())))
embedded_input <- structure(list(ContrastOrder = c(1, 1, 1, 1, 1, 1, 1, 1, 1, 2,
2, 2, 2, 2, 2, 2, 2, 2, 3, 3, 3, 4, 4, 4, 4, 4, 4, 4, 4, 4, 4
), Contrast = c("Overweight vs normal weight", "Overweight vs normal weight",
"Overweight vs normal weight", "Overweight vs normal weight",
"Overweight vs normal weight", "Overweight vs normal weight",
"Overweight vs normal weight", "Overweight vs normal weight",
"Overweight vs normal weight", "Obese vs normal weight", "Obese vs normal weight",
"Obese vs normal weight", "Obese vs normal weight", "Obese vs normal weight",
"Obese vs normal weight", "Obese vs normal weight", "Obese vs normal weight",
"Obese vs normal weight", "Underweight vs normal weight", "Underweight vs normal weight",
"Underweight vs normal weight", "Obese/Overweight vs normal/underweight",
"Obese/Overweight vs normal/underweight", "Obese/Overweight vs normal/underweight",
"Obese/Overweight vs normal/underweight", "Obese/Overweight vs normal/underweight",
"Obese/Overweight vs normal/underweight", "Obese/Overweight vs normal/underweight",
"Obese/Overweight vs normal/underweight", "Obese/Overweight vs normal/underweight",
"Obese/Overweight vs normal/underweight"), Study = c("McQuade 2018 (1)",
"McQuade 2018 (2)", "Cortellini 2020", "Wang 2022", "Zhang 2023",
"Vithayathil 2023", "Gomez-Banoy 2024", "Nie 2024 (1)", "Nie 2024 (2)",
"McQuade 2018 (1)", "McQuade 2018 (2)", "Cortellini 2020", "Wang 2022",
"Zhang 2023", "Vithayathil 2023", "Gomez-Banoy 2024", "Nie 2024 (1)",
"Nie 2024 (2)", "Cortellini 2020", "Zhang 2023", "Vithayathil 2023",
"Cortellini 2019", "Ahmed 2021", "Akce 2021", "Esposito 2021",
"Zhang 2021", "Chen 2022", "Tateishi 2022", "Vithayathil 2023",
"Alam 2024", "Wang 2024"), Effect = c(0.88, 0.82, 1.04, 0.74,
0.26, 0.69, 0.71, 0.97, 1.33, 0.63, 0.85, 0.61, 0.44, 0.863,
0.8, 0.54, 1.01, 1.14, 1.4600671630895, 5.55555555555556, 1.85185185185185,
0.71, 0.87, 1.04, 0.8, 0.76, 0.47, 1, 0.7, 0.82, 0.32), LowerCI = c(0.61,
0.58, 0.85, 0.41, 0.032, 0.36, 0.54, 0.83, 0.85, 0.41, 0.61,
0.45, 0.17, 0.091, 0.41, 0.42, 0.84, 0.68, 0.980007840062721,
1.36986301369863, 0.609756097560976, 0.56, 0.59, 0.56, 0.54,
0.55, 0.22, 0.4, 0.41, 0.49, 0.17), UpperCI = c(1.26, 1.16, 1.26,
1.36, 2.135, 1.34, 0.93, 1.15, 2.09, 0.95, 1.19, 0.82, 1.13,
8.156, 1.56, 0.71, 1.21, 1.92, 2.2002200220022, 25, 5.55555555555556,
0.9, 1.28, 1.92, 1.19, 1.04, 0.98, 2.45, 1.2, 1.38, 0.59)), row.names = c(NA,
-31L), class = "data.frame")


reml_meta <- function (yi, sei)
{
    keep <- is.finite(yi) & is.finite(sei) & sei > 0
    yi <- yi[keep]
    sei <- sei[keep]
    vi <- sei^2
    k <- length(yi)
    if (!k)
        stop("No valid estimates.")
    if (k == 1L) {
        z <- qnorm(0.975)
        return(list(k = 1L, mu = yi, se_mu = sei, ci_low = yi - z * sei, ci_high = yi + z * sei, p = 2 * pnorm(abs(yi/sei), lower.tail = FALSE), tau2 = NA_real_, Q = NA_real_, df_Q = 0L, Q_p = NA_real_, I2 = NA_real_, weights = 100))
    }
    wf <- 1/vi
    muf <- sum(wf * yi)/sum(wf)
    Q <- sum(wf * (yi - muf)^2)
    dfQ <- k - 1
    I2 <- if (Q > 0)
        max(0, (Q - dfQ)/Q) * 100
    else 0
    obj <- function(tau2) {
        w <- 1/(vi + tau2)
        mu <- sum(w * yi)/sum(w)
        0.5 * (sum(log(vi + tau2)) + log(sum(w)) + sum(w * (yi - mu)^2))
    }
    upper <- max(1, var(yi) * 10, max(vi) * 10)
    opt <- optimize(obj, c(0, upper), tol = 1e-12)
    tau2 <- if (opt$minimum < 1e-10)
        0
    else opt$minimum
    w <- 1/(vi + tau2)
    mu <- sum(w * yi)/sum(w)
    se <- sqrt(1/sum(w))
    ci <- mu + c(-1, 1) * qnorm(0.975) * se
    list(k = k, mu = mu, se_mu = se, ci_low = ci[1], ci_high = ci[2], p = 2 * pnorm(abs(mu/se), lower.tail = FALSE), tau2 = tau2, Q = Q, df_Q = dfQ, Q_p = pchisq(Q, dfQ, lower.tail = FALSE), I2 = I2, weights = w/sum(w) * 100)
}
prepare_effects <- function (d, effect_col = "Effect")
{
    d[[effect_col]] <- as.numeric(d[[effect_col]])
    d$LowerCI <- as.numeric(d$LowerCI)
    d$UpperCI <- as.numeric(d$UpperCI)
    ok <- is.finite(d[[effect_col]]) & is.finite(d$LowerCI) & is.finite(d$UpperCI) & d[[effect_col]] > 0 & d$LowerCI > 0 & d$UpperCI > d$LowerCI
    d <- d[ok, , drop = FALSE]
    d$Effect <- d[[effect_col]]
    d$yi <- log(d$Effect)
    d$sei <- (log(d$UpperCI) - log(d$LowerCI))/(2 * qnorm(0.975))
    d
}
fmt_p <- function (p)
if (!is.finite(p)) "NR" else if (p < 1e-04) "< 0.0001" else paste0("= ", formatC(p, format = "f", digits = 4))
funnel_plot <- function (d, m, title, xlab, file, note = TRUE)
{
    jpeg(file, width = 8, height = 7, units = "in", res = 300, quality = 100)
    on.exit(dev.off(), add = TRUE)
    par(mar = c(5, 5, 3.3, 1.2), family = "sans", las = 1)
    ym <- max(d$sei) * 1.08
    xc <- c(d$yi, m$mu - 1.96 * ym, m$mu + 1.96 * ym)
    pad <- diff(range(xc)) * 0.08
    plot(d$yi, d$sei, xlim = range(xc) + c(-pad, pad), ylim = c(ym, 0), pch = 21, bg = if (grepl("hazard", tolower(xlab)))
        "#2F80C0"
    else "#D0D0D0", col = if (grepl("hazard", tolower(xlab)))
        "#004C99"
    else "black", cex = 1.15, xlab = xlab, ylab = "Standard error", main = title, bty = "l")
    s <- seq(0, ym, length.out = 200)
    lines(m$mu - 1.96 * s, s, lty = 2, col = "#707070")
    lines(m$mu + 1.96 * s, s, lty = 2, col = "#707070")
    abline(v = m$mu, col = if (grepl("hazard", tolower(xlab)))
        "#D73027"
    else "#333333", lty = if (grepl("hazard", tolower(xlab)))
        1
    else 3, lwd = 1.2)
    if (note) {
        if (nrow(d) < 10)
            mtext(sprintf("Exploratory only; Egger test not performed (k = %d < 10).", nrow(d)), side = 1, line = 3.7, cex = 0.78)
    }
}
category_forest <- function (d, endpoint, file)
{
    if (!requireNamespace("metafor", quietly = TRUE))
        stop("Package 'metafor' is required.")
    labels <- unique(d$Contrast[order(d$ContrastOrder)])
    d$yi <- log(d$Effect)
    d$sei <- (log(d$UpperCI) - log(d$LowerCI))/(2 * 1.96)
    d$vi <- d$sei^2
    models <- lapply(labels, function(g) metafor::rma.uni(yi, vi, data = d[d$Contrast == g, ], method = "REML", test = "knha"))
    names(models) <- labels
    rows <- list()
    for (g in labels) {
        rows[[length(rows) + 1]] <- data.frame(type = "title", g = g, i = NA)
        for (i in which(d$Contrast == g)) rows[[length(rows) + 1]] <- data.frame(type = "study", g = g, i = i)
        rows[[length(rows) + 1]] <- data.frame(type = "sum", g = g, i = NA)
        rows[[length(rows) + 1]] <- data.frame(type = "het", g = g, i = NA)
        rows[[length(rows) + 1]] <- data.frame(type = "space", g = g, i = NA)
    }
    rp <- do.call(rbind, rows)
    rp$y <- rev(seq_len(nrow(rp))) + 3
    h <- if (endpoint == "OS")
        19.3
    else 16.2
    jpeg(file, width = 11, height = h, units = "in", res = 300, quality = 100)
    on.exit(dev.off(), add = TRUE)
    par(mar = c(2.4, 0.6, 2, 0.6), family = "sans", xpd = NA)
    plot.new()
    plot.window(xlim = c(0, 1), ylim = c(0, max(rp$y) + 4.8))
    xs <- 0.025
    xln <- 0.315
    xse <- 0.4
    pl <- 0.475
    pr <- 0.715
    xhr <- 0.755
    xci <- 0.825
    xw <- 0.965
    map <- function(v) pl + (log10(v) + 1)/2 * (pr - pl)
    maxw <- 0
    for (g in labels) {
        z <- d[d$Contrast == g, ]
        m <- models[[g]]
        w <- as.numeric(weights(m))
        d$w[d$Contrast == g] <- 100 * w/sum(w)
        maxw <- max(maxw, d$w[d$Contrast == g])
    }
    title_txt <- if (endpoint == "OS")
        "Association between BMI categories and overall survival"
    else "Association between BMI categories and progression-free survival"
    text(0.5, max(rp$y) + 3.9, title_txt, font = 2, cex = 1.02)
    hy <- max(rp$y) + 2.2
    text(xs, hy, "Study", adj = 0, font = 2, cex = 0.74)
    text(xln, hy, "lnHR", adj = 1, font = 2, cex = 0.74)
    text(xse, hy, "SE(lnHR)", adj = 1, font = 2, cex = 0.74)
    text((pl + pr)/2, hy, "Hazard Ratio", font = 2, cex = 0.74)
    text(xhr, hy, "HR", adj = 1, font = 2, cex = 0.74)
    text(xci, hy, "95% CI", adj = 0, font = 2, cex = 0.74)
    text(xw, hy, "Weight", adj = 1, font = 2, cex = 0.74)
    text(xw, hy - 0.55, "(within comparison)", adj = 1, cex = 0.55)
    segments(map(1), 2.7, map(1), max(rp$y) + 1, lwd = 0.8)
    num <- setNames(seq_along(labels), labels)
    for (r in seq_len(nrow(rp))) {
        z <- rp[r, ]
        y <- z$y
        if (z$type == "title")
            text(xs, y, paste0(num[z$g], ". ", z$g), adj = 0, font = 2, cex = 0.76)
        if (z$type == "study") {
            i <- z$i
            segments(map(max(0.1, d$LowerCI[i])), y, map(min(10, d$UpperCI[i])), y, col = "#333333", lwd = 0.8)
            if (d$LowerCI[i] < 0.1)
                arrows(map(0.12), y, map(0.1), y, length = 0.04)
            if (d$UpperCI[i] > 10)
                arrows(map(8.5), y, map(10), y, length = 0.04)
            points(map(d$Effect[i]), y, pch = 15, col = "#A7C1D0", cex = 0.55 + 1.1 * sqrt(d$w[i]/maxw))
            text(xs, y, d$Study[i], adj = 0, cex = 0.67)
            text(xln, y, formatC(d$yi[i], format = "f", digits = 4), adj = 1, cex = 0.64)
            text(xse, y, formatC(d$sei[i], format = "f", digits = 4), adj = 1, cex = 0.64)
            text(xhr, y, sprintf("%.2f", d$Effect[i]), adj = 1, cex = 0.67)
            text(xci, y, sprintf("[%.2f; %.2f]", d$LowerCI[i], d$UpperCI[i]), adj = 0, cex = 0.67)
            text(xw, y, sprintf("%.2f%%", d$w[i]), adj = 1, cex = 0.67)
        }
        if (z$type == "sum") {
            m <- models[[z$g]]
            est <- exp(as.numeric(m$b))
            lb <- exp(m$ci.lb)
            ub <- exp(m$ci.ub)
            polygon(c(map(lb), map(est), map(ub), map(est)), c(y, y + 0.34, y, y - 0.34), col = "#E25353", border = "#333333")
            text(xs, y, "Random-effects model", adj = 0, font = 2, cex = 0.68)
            text(xhr, y, sprintf("%.2f", est), adj = 1, font = 2, cex = 0.68)
            text(xci, y, sprintf("[%.2f; %.2f]", lb, ub), adj = 0, font = 2, cex = 0.68)
            text(xw, y, "100.00%", adj = 1, font = 2, cex = 0.68)
        }
        if (z$type == "het") {
            m <- models[[z$g]]
            text(xs, y, sprintf("Heterogeneity: I² = %.1f%%, τ² = %.4f, p %s", m$I2, m$tau2, fmt_p(m$QEp)), adj = 0, cex = 0.64)
        }
    }
    ay <- 2.8
    segments(pl, ay, pr, ay)
    for (t in c(0.1, 0.2, 0.5, 1, 2, 5, 10)) {
        segments(map(t), ay, map(t), ay - 0.28)
        text(map(t), ay - 0.52, t, cex = 0.66)
    }
    text((pl + pr)/2, ay - 1.05, "Hazard Ratio", cex = 0.7)
}
category_sensitivity <- function (d, label, file)
{
    if (!requireNamespace("metafor", quietly = TRUE))
        stop("Package 'metafor' is required.")
    d$yi <- log(d$Effect)
    d$sei <- (log(d$UpperCI) - log(d$LowerCI))/(2 * 1.96)
    m <- metafor::rma.uni(d$yi, sei = d$sei, method = "REML", test = "knha")
    loo <- metafor::leave1out(m)
    k <- nrow(d)
    est <- exp(loo$estimate)
    lb <- exp(loo$ci.lb)
    ub <- exp(loo$ci.ub)
    full <- exp(as.numeric(m$b))
    fl <- exp(m$ci.lb)
    fu <- exp(m$ci.ub)
    num <- unique(d$ContrastOrder)[1]
    jpeg(file, width = 10, height = max(6.2, 4.24 + 0.385 * k), units = "in", res = 300, quality = 100)
    on.exit(dev.off(), add = TRUE)
    par(mar = c(3.9, 0.6, 2.7, 0.6), family = "sans", xpd = NA)
    plot.new()
    plot.window(xlim = c(0, 1), ylim = c(-1.9, k + 3.1))
    xs <- 0.025
    pl <- 0.46
    pr <- 0.75
    xhr <- 0.81
    xci <- 0.9
    map <- function(v) pl + (log(v) - log(0.1))/(log(10) - log(0.1)) * (pr - pl)
    y <- rev(seq_len(k)) + 1.1
    for (i in seq_len(k)) if (i%%2 == 0)
        rect(0.015, y[i] - 0.45, 0.985, y[i] + 0.45, col = "#F4F4F4", border = NA)
    text(xs, k + 2.25, "Study omitted", adj = 0, font = 2, cex = 0.78)
    text((pl + pr)/2, k + 2.25, "Pooled Hazard Ratio", font = 2, cex = 0.78)
    text(xhr, k + 2.25, "HR", adj = 1, font = 2, cex = 0.78)
    text(xci, k + 2.25, "95% CI", font = 2, cex = 0.78)
    segments(map(1), -0.2, map(1), k + 1.5)
    segments(map(full), -0.2, map(full), k + 1.5, lty = 3, col = "red")
    for (i in seq_len(k)) {
        segments(map(max(0.1, lb[i])), y[i], map(min(10, ub[i])), y[i], lwd = 0.8)
        points(map(est[i]), y[i], pch = 15, col = "#AFAFAF", cex = 0.9)
        text(xs, y[i], paste("Omit", d$Study[i]), adj = 0, cex = 0.7)
        text(xhr, y[i], sprintf("%.2f", est[i]), adj = 1, cex = 0.7)
        text(xci, y[i], sprintf("[%.2f; %.2f]", lb[i], ub[i]), cex = 0.7)
    }
    py <- 0.05
    polygon(c(map(fl), map(full), map(fu), map(full)), c(py, py + 0.28, py, py - 0.28), col = "red", border = "black")
    text(xs, py, "Full random-effects model", adj = 0, font = 2, cex = 0.72)
    text(xhr, py, sprintf("%.2f", full), adj = 1, font = 2, cex = 0.72)
    text(xci, py, sprintf("[%.2f; %.2f]", fl, fu), font = 2, cex = 0.72)
    ay <- -0.65
    segments(pl, ay, pr, ay)
    for (t in c(0.1, 0.2, 0.5, 1, 2, 5, 10)) {
        segments(map(t), ay, map(t), ay - 0.15)
        text(map(t), ay - 0.35, t, cex = 0.66)
    }
    text((pl + pr)/2, ay - 0.72, "Hazard Ratio", cex = 0.7)
    mtext(paste0("Leave-one-out sensitivity analysis: ", num, ". ", label), side = 3, line = 0.7, font = 2, cex = 0.86)
    text(0.5, -1.65, sprintf("Dashed red line = full-model pooled HR; REML with Knapp-Hartung; full-model I2=%.2f%%", m$I2), cex = 0.6, col = "#555555")
}
category_funnel <- function (d, label, file)
{
    d$yi <- log(d$Effect)
    d$sei <- (log(d$UpperCI) - log(d$LowerCI))/(2 * 1.96)
    m <- reml_meta(d$yi, d$sei)
    k <- nrow(d)
    ym <- max(d$sei) * 1.08
    s <- seq(0, ym, length.out = 200)
    xl <- range(c(d$yi, m$mu - 1.96 * ym, m$mu + 1.96 * ym))
    pad <- diff(xl) * 0.08
    num <- unique(d$ContrastOrder)[1]
    ep <- if (k >= 10) {
        fit <- metafor::rma.uni(yi = d$yi, sei = d$sei, method = "REML")
        eg <- metafor::regtest(fit, model = "rma", predictor = "sei")
        sprintf("k=%d; Egger regression p=%.3f", k, eg$pval)
    }
    else sprintf("k=%d; Egger regression not performed", k)
    jpeg(file, width = 8, height = 7, units = "in", res = 300, quality = 100)
    on.exit(dev.off(), add = TRUE)
    par(mar = c(5, 5, 4.2, 1.2), family = "sans", las = 1)
    plot(d$yi, d$sei, xlim = xl + c(-pad, pad), ylim = c(ym, 0), pch = 15, col = "#B5B5B5", cex = 1.15, xlab = "Log Hazard Ratio", ylab = "Standard Error", main = paste0("Funnel plot:  ", num, ". ", label), bty = "l")
    polygon(c(m$mu - 1.96 * ym, m$mu, m$mu + 1.96 * ym), c(ym, 0, ym), col = "#EFEFEF", border = NA)
    lines(m$mu - 1.96 * s, s, lty = 2, col = "#777777")
    lines(m$mu + 1.96 * s, s, lty = 2, col = "#777777")
    abline(v = m$mu, col = "red", lwd = 1.4)
    points(d$yi, d$sei, pch = 15, col = "#B5B5B5", cex = 1.15)
    mtext(ep, side = 3, line = 0.65, cex = 0.78, col = "#444444")
}
d<-prepare_effects(as.data.frame(embedded_input))
category_forest(d,"PFS",'forest.jpg')
for(g in unique(d$Contrast[order(d$ContrastOrder)])){z<-d[d$Contrast==g,,drop=FALSE];stem<-gsub('[^A-Za-z0-9]+','_',g);if(nrow(z)>=3)category_sensitivity(z,g,paste0('sensitivity_',stem,'.jpg'));category_funnel(z,g,paste0('funnel_',stem,'.jpg'))}
```

**Supplementary Code S14.**BMI categories and PFS — unadjusted estimates

```
# Analysis: 14_BMI_categories_PFS_unadjusted
# All analysis data are embedded. Running this file creates figures only.
rm(list = ls())
options(stringsAsFactors = FALSE, scipen = 999)
analysis_id <- "14_BMI_categories_PFS_unadjusted"
script_arg <- grep('^--file=', commandArgs(trailingOnly = FALSE), value = TRUE)
script_file <- if (length(script_arg)) sub('^--file=', '', script_arg[1]) else NA_character_
if ((is.na(script_file) || !nzchar(script_file)) && interactive() && requireNamespace('rstudioapi', quietly = TRUE)) {
  p <- tryCatch(rstudioapi::getSourceEditorContext()$path, error = function(e) '')
  if (nzchar(p)) script_file <- p
}
script_dir <- if (!is.na(script_file) && nzchar(script_file)) dirname(normalizePath(script_file, winslash='/', mustWork=TRUE)) else normalizePath(getwd(), winslash='/', mustWork=TRUE)
output_dir <- file.path(script_dir, paste0(analysis_id, '_output'))
dir.create(output_dir, recursive=TRUE, showWarnings=FALSE)
setwd(output_dir)
.libPaths(unique(c(file.path(Sys.getenv('USERPROFILE'), 'R-library'), .libPaths())))
embedded_input <- structure(list(ContrastOrder = c(1, 1, 1, 1, 1, 2, 2, 2, 2, 2,
3, 3, 3, 4, 4, 4, 4, 4, 4, 4, 4, 4, 4), Contrast = c("Overweight vs normal weight",
"Overweight vs normal weight", "Overweight vs normal weight",
"Overweight vs normal weight", "Overweight vs normal weight",
"Obese vs normal weight", "Obese vs normal weight", "Obese vs normal weight",
"Obese vs normal weight", "Obese vs normal weight", "Underweight vs normal weight",
"Underweight vs normal weight", "Underweight vs normal weight",
"Obese/Overweight vs normal/underweight", "Obese/Overweight vs normal/underweight",
"Obese/Overweight vs normal/underweight", "Obese/Overweight vs normal/underweight",
"Obese/Overweight vs normal/underweight", "Obese/Overweight vs normal/underweight",
"Obese/Overweight vs normal/underweight", "Obese/Overweight vs normal/underweight",
"Obese/Overweight vs normal/underweight", "Obese/Overweight vs normal/underweight"
), Study = c("Labadie 2019", "Kichenadasse 2019", "Åžen 2024",
"Demir 2025", "Wang 2024", "Labadie 2019", "Kichenadasse 2019",
"Åžen 2024", "Demir 2025", "Wang 2024", "Cortellini 2019",
"Labadie 2019", "Ichihara 2020", "Qi 2019", "Ahmed 2021", "Esposito 2021",
"Ged 2022", "Tateishi 2022", "Vithayathil 2023", "Santoni 2023",
"Wang 2024", "Åžen 2024", "Demir 2025"), Effect = c(0.5, 0.89,
1.1, 0.86, 0.36, 0.39, 0.86, 1.04, 0.82, 0.13, 2.22222222222222,
3.03030303030303, 1.2987012987013, 0.751, 0.69, 0.79, 0.99, 0.86,
0.9, 0.79, 0.28, 1.16, 0.86), LowerCI = c(0.28, 0.78, 0.69, 0.65,
0.19, 0.19, 0.73, 0.58, 0.59, 0.06, 1.5625, 0.4, 1, 0.458, 0.51,
0.55, 0.71, 0.6, 0.55, 0.61, 0.15, 0.76, 0.67), UpperCI = c(0.88,
1.01, 1.75, 1.13, 0.69, 0.82, 1.01, 1.85, 1.31, 0.31, 3.125,
23.2558139534884, 1.69491525423729, 1.232, 0.94, 1.14, 1.38,
1.25, 1.49, 1.02, 0.52, 1.75, 1.11)), row.names = c(NA, -23L), class = "data.frame")


reml_meta <- function (yi, sei)
{
    keep <- is.finite(yi) & is.finite(sei) & sei > 0
    yi <- yi[keep]
    sei <- sei[keep]
    vi <- sei^2
    k <- length(yi)
    if (!k)
        stop("No valid estimates.")
    if (k == 1L) {
        z <- qnorm(0.975)
        return(list(k = 1L, mu = yi, se_mu = sei, ci_low = yi - z * sei, ci_high = yi + z * sei, p = 2 * pnorm(abs(yi/sei), lower.tail = FALSE), tau2 = NA_real_, Q = NA_real_, df_Q = 0L, Q_p = NA_real_, I2 = NA_real_, weights = 100))
    }
    wf <- 1/vi
    muf <- sum(wf * yi)/sum(wf)
    Q <- sum(wf * (yi - muf)^2)
    dfQ <- k - 1
    I2 <- if (Q > 0)
        max(0, (Q - dfQ)/Q) * 100
    else 0
    obj <- function(tau2) {
        w <- 1/(vi + tau2)
        mu <- sum(w * yi)/sum(w)
        0.5 * (sum(log(vi + tau2)) + log(sum(w)) + sum(w * (yi - mu)^2))
    }
    upper <- max(1, var(yi) * 10, max(vi) * 10)
    opt <- optimize(obj, c(0, upper), tol = 1e-12)
    tau2 <- if (opt$minimum < 1e-10)
        0
    else opt$minimum
    w <- 1/(vi + tau2)
    mu <- sum(w * yi)/sum(w)
    se <- sqrt(1/sum(w))
    ci <- mu + c(-1, 1) * qnorm(0.975) * se
    list(k = k, mu = mu, se_mu = se, ci_low = ci[1], ci_high = ci[2], p = 2 * pnorm(abs(mu/se), lower.tail = FALSE), tau2 = tau2, Q = Q, df_Q = dfQ, Q_p = pchisq(Q, dfQ, lower.tail = FALSE), I2 = I2, weights = w/sum(w) * 100)
}
prepare_effects <- function (d, effect_col = "Effect")
{
    d[[effect_col]] <- as.numeric(d[[effect_col]])
    d$LowerCI <- as.numeric(d$LowerCI)
    d$UpperCI <- as.numeric(d$UpperCI)
    ok <- is.finite(d[[effect_col]]) & is.finite(d$LowerCI) & is.finite(d$UpperCI) & d[[effect_col]] > 0 & d$LowerCI > 0 & d$UpperCI > d$LowerCI
    d <- d[ok, , drop = FALSE]
    d$Effect <- d[[effect_col]]
    d$yi <- log(d$Effect)
    d$sei <- (log(d$UpperCI) - log(d$LowerCI))/(2 * qnorm(0.975))
    d
}
fmt_p <- function (p)
if (!is.finite(p)) "NR" else if (p < 1e-04) "< 0.0001" else paste0("= ", formatC(p, format = "f", digits = 4))
funnel_plot <- function (d, m, title, xlab, file, note = TRUE)
{
    jpeg(file, width = 8, height = 7, units = "in", res = 300, quality = 100)
    on.exit(dev.off(), add = TRUE)
    par(mar = c(5, 5, 3.3, 1.2), family = "sans", las = 1)
    ym <- max(d$sei) * 1.08
    xc <- c(d$yi, m$mu - 1.96 * ym, m$mu + 1.96 * ym)
    pad <- diff(range(xc)) * 0.08
    plot(d$yi, d$sei, xlim = range(xc) + c(-pad, pad), ylim = c(ym, 0), pch = 21, bg = if (grepl("hazard", tolower(xlab)))
        "#2F80C0"
    else "#D0D0D0", col = if (grepl("hazard", tolower(xlab)))
        "#004C99"
    else "black", cex = 1.15, xlab = xlab, ylab = "Standard error", main = title, bty = "l")
    s <- seq(0, ym, length.out = 200)
    lines(m$mu - 1.96 * s, s, lty = 2, col = "#707070")
    lines(m$mu + 1.96 * s, s, lty = 2, col = "#707070")
    abline(v = m$mu, col = if (grepl("hazard", tolower(xlab)))
        "#D73027"
    else "#333333", lty = if (grepl("hazard", tolower(xlab)))
        1
    else 3, lwd = 1.2)
    if (note) {
        if (nrow(d) < 10)
            mtext(sprintf("Exploratory only; Egger test not performed (k = %d < 10).", nrow(d)), side = 1, line = 3.7, cex = 0.78)
    }
}
category_forest <- function (d, endpoint, file)
{
    if (!requireNamespace("metafor", quietly = TRUE))
        stop("Package 'metafor' is required.")
    labels <- unique(d$Contrast[order(d$ContrastOrder)])
    d$yi <- log(d$Effect)
    d$sei <- (log(d$UpperCI) - log(d$LowerCI))/(2 * 1.96)
    d$vi <- d$sei^2
    models <- lapply(labels, function(g) metafor::rma.uni(yi, vi, data = d[d$Contrast == g, ], method = "REML", test = "knha"))
    names(models) <- labels
    rows <- list()
    for (g in labels) {
        rows[[length(rows) + 1]] <- data.frame(type = "title", g = g, i = NA)
        for (i in which(d$Contrast == g)) rows[[length(rows) + 1]] <- data.frame(type = "study", g = g, i = i)
        rows[[length(rows) + 1]] <- data.frame(type = "sum", g = g, i = NA)
        rows[[length(rows) + 1]] <- data.frame(type = "het", g = g, i = NA)
        rows[[length(rows) + 1]] <- data.frame(type = "space", g = g, i = NA)
    }
    rp <- do.call(rbind, rows)
    rp$y <- rev(seq_len(nrow(rp))) + 3
    h <- if (endpoint == "OS")
        19.3
    else 16.2
    jpeg(file, width = 11, height = h, units = "in", res = 300, quality = 100)
    on.exit(dev.off(), add = TRUE)
    par(mar = c(2.4, 0.6, 2, 0.6), family = "sans", xpd = NA)
    plot.new()
    plot.window(xlim = c(0, 1), ylim = c(0, max(rp$y) + 4.8))
    xs <- 0.025
    xln <- 0.315
    xse <- 0.4
    pl <- 0.475
    pr <- 0.715
    xhr <- 0.755
    xci <- 0.825
    xw <- 0.965
    map <- function(v) pl + (log10(v) + 1)/2 * (pr - pl)
    maxw <- 0
    for (g in labels) {
        z <- d[d$Contrast == g, ]
        m <- models[[g]]
        w <- as.numeric(weights(m))
        d$w[d$Contrast == g] <- 100 * w/sum(w)
        maxw <- max(maxw, d$w[d$Contrast == g])
    }
    title_txt <- if (endpoint == "OS")
        "Association between BMI categories and overall survival"
    else "Association between BMI categories and progression-free survival"
    text(0.5, max(rp$y) + 3.9, title_txt, font = 2, cex = 1.02)
    hy <- max(rp$y) + 2.2
    text(xs, hy, "Study", adj = 0, font = 2, cex = 0.74)
    text(xln, hy, "lnHR", adj = 1, font = 2, cex = 0.74)
    text(xse, hy, "SE(lnHR)", adj = 1, font = 2, cex = 0.74)
    text((pl + pr)/2, hy, "Hazard Ratio", font = 2, cex = 0.74)
    text(xhr, hy, "HR", adj = 1, font = 2, cex = 0.74)
    text(xci, hy, "95% CI", adj = 0, font = 2, cex = 0.74)
    text(xw, hy, "Weight", adj = 1, font = 2, cex = 0.74)
    text(xw, hy - 0.55, "(within comparison)", adj = 1, cex = 0.55)
    segments(map(1), 2.7, map(1), max(rp$y) + 1, lwd = 0.8)
    num <- setNames(seq_along(labels), labels)
    for (r in seq_len(nrow(rp))) {
        z <- rp[r, ]
        y <- z$y
        if (z$type == "title")
            text(xs, y, paste0(num[z$g], ". ", z$g), adj = 0, font = 2, cex = 0.76)
        if (z$type == "study") {
            i <- z$i
            segments(map(max(0.1, d$LowerCI[i])), y, map(min(10, d$UpperCI[i])), y, col = "#333333", lwd = 0.8)
            if (d$LowerCI[i] < 0.1)
                arrows(map(0.12), y, map(0.1), y, length = 0.04)
            if (d$UpperCI[i] > 10)
                arrows(map(8.5), y, map(10), y, length = 0.04)
            points(map(d$Effect[i]), y, pch = 15, col = "#A7C1D0", cex = 0.55 + 1.1 * sqrt(d$w[i]/maxw))
            text(xs, y, d$Study[i], adj = 0, cex = 0.67)
            text(xln, y, formatC(d$yi[i], format = "f", digits = 4), adj = 1, cex = 0.64)
            text(xse, y, formatC(d$sei[i], format = "f", digits = 4), adj = 1, cex = 0.64)
            text(xhr, y, sprintf("%.2f", d$Effect[i]), adj = 1, cex = 0.67)
            text(xci, y, sprintf("[%.2f; %.2f]", d$LowerCI[i], d$UpperCI[i]), adj = 0, cex = 0.67)
            text(xw, y, sprintf("%.2f%%", d$w[i]), adj = 1, cex = 0.67)
        }
        if (z$type == "sum") {
            m <- models[[z$g]]
            est <- exp(as.numeric(m$b))
            lb <- exp(m$ci.lb)
            ub <- exp(m$ci.ub)
            polygon(c(map(lb), map(est), map(ub), map(est)), c(y, y + 0.34, y, y - 0.34), col = "#E25353", border = "#333333")
            text(xs, y, "Random-effects model", adj = 0, font = 2, cex = 0.68)
            text(xhr, y, sprintf("%.2f", est), adj = 1, font = 2, cex = 0.68)
            text(xci, y, sprintf("[%.2f; %.2f]", lb, ub), adj = 0, font = 2, cex = 0.68)
            text(xw, y, "100.00%", adj = 1, font = 2, cex = 0.68)
        }
        if (z$type == "het") {
            m <- models[[z$g]]
            text(xs, y, sprintf("Heterogeneity: I² = %.1f%%, τ² = %.4f, p %s", m$I2, m$tau2, fmt_p(m$QEp)), adj = 0, cex = 0.64)
        }
    }
    ay <- 2.8
    segments(pl, ay, pr, ay)
    for (t in c(0.1, 0.2, 0.5, 1, 2, 5, 10)) {
        segments(map(t), ay, map(t), ay - 0.28)
        text(map(t), ay - 0.52, t, cex = 0.66)
    }
    text((pl + pr)/2, ay - 1.05, "Hazard Ratio", cex = 0.7)
}
category_sensitivity <- function (d, label, file)
{
    if (!requireNamespace("metafor", quietly = TRUE))
        stop("Package 'metafor' is required.")
    d$yi <- log(d$Effect)
    d$sei <- (log(d$UpperCI) - log(d$LowerCI))/(2 * 1.96)
    m <- metafor::rma.uni(d$yi, sei = d$sei, method = "REML", test = "knha")
    loo <- metafor::leave1out(m)
    k <- nrow(d)
    est <- exp(loo$estimate)
    lb <- exp(loo$ci.lb)
    ub <- exp(loo$ci.ub)
    full <- exp(as.numeric(m$b))
    fl <- exp(m$ci.lb)
    fu <- exp(m$ci.ub)
    num <- unique(d$ContrastOrder)[1]
    jpeg(file, width = 10, height = max(6.2, 4.24 + 0.385 * k), units = "in", res = 300, quality = 100)
    on.exit(dev.off(), add = TRUE)
    par(mar = c(3.9, 0.6, 2.7, 0.6), family = "sans", xpd = NA)
    plot.new()
    plot.window(xlim = c(0, 1), ylim = c(-1.9, k + 3.1))
    xs <- 0.025
    pl <- 0.46
    pr <- 0.75
    xhr <- 0.81
    xci <- 0.9
    map <- function(v) pl + (log(v) - log(0.1))/(log(10) - log(0.1)) * (pr - pl)
    y <- rev(seq_len(k)) + 1.1
    for (i in seq_len(k)) if (i%%2 == 0)
        rect(0.015, y[i] - 0.45, 0.985, y[i] + 0.45, col = "#F4F4F4", border = NA)
    text(xs, k + 2.25, "Study omitted", adj = 0, font = 2, cex = 0.78)
    text((pl + pr)/2, k + 2.25, "Pooled Hazard Ratio", font = 2, cex = 0.78)
    text(xhr, k + 2.25, "HR", adj = 1, font = 2, cex = 0.78)
    text(xci, k + 2.25, "95% CI", font = 2, cex = 0.78)
    segments(map(1), -0.2, map(1), k + 1.5)
    segments(map(full), -0.2, map(full), k + 1.5, lty = 3, col = "red")
    for (i in seq_len(k)) {
        segments(map(max(0.1, lb[i])), y[i], map(min(10, ub[i])), y[i], lwd = 0.8)
        points(map(est[i]), y[i], pch = 15, col = "#AFAFAF", cex = 0.9)
        text(xs, y[i], paste("Omit", d$Study[i]), adj = 0, cex = 0.7)
        text(xhr, y[i], sprintf("%.2f", est[i]), adj = 1, cex = 0.7)
        text(xci, y[i], sprintf("[%.2f; %.2f]", lb[i], ub[i]), cex = 0.7)
    }
    py <- 0.05
    polygon(c(map(fl), map(full), map(fu), map(full)), c(py, py + 0.28, py, py - 0.28), col = "red", border = "black")
    text(xs, py, "Full random-effects model", adj = 0, font = 2, cex = 0.72)
    text(xhr, py, sprintf("%.2f", full), adj = 1, font = 2, cex = 0.72)
    text(xci, py, sprintf("[%.2f; %.2f]", fl, fu), font = 2, cex = 0.72)
    ay <- -0.65
    segments(pl, ay, pr, ay)
    for (t in c(0.1, 0.2, 0.5, 1, 2, 5, 10)) {
        segments(map(t), ay, map(t), ay - 0.15)
        text(map(t), ay - 0.35, t, cex = 0.66)
    }
    text((pl + pr)/2, ay - 0.72, "Hazard Ratio", cex = 0.7)
    mtext(paste0("Leave-one-out sensitivity analysis: ", num, ". ", label), side = 3, line = 0.7, font = 2, cex = 0.86)
    text(0.5, -1.65, sprintf("Dashed red line = full-model pooled HR; REML with Knapp-Hartung; full-model I2=%.2f%%", m$I2), cex = 0.6, col = "#555555")
}
category_funnel <- function (d, label, file)
{
    d$yi <- log(d$Effect)
    d$sei <- (log(d$UpperCI) - log(d$LowerCI))/(2 * 1.96)
    m <- reml_meta(d$yi, d$sei)
    k <- nrow(d)
    ym <- max(d$sei) * 1.08
    s <- seq(0, ym, length.out = 200)
    xl <- range(c(d$yi, m$mu - 1.96 * ym, m$mu + 1.96 * ym))
    pad <- diff(xl) * 0.08
    num <- unique(d$ContrastOrder)[1]
    ep <- if (k >= 10) {
        fit <- metafor::rma.uni(yi = d$yi, sei = d$sei, method = "REML")
        eg <- metafor::regtest(fit, model = "rma", predictor = "sei")
        sprintf("k=%d; Egger regression p=%.3f", k, eg$pval)
    }
    else sprintf("k=%d; Egger regression not performed", k)
    jpeg(file, width = 8, height = 7, units = "in", res = 300, quality = 100)
    on.exit(dev.off(), add = TRUE)
    par(mar = c(5, 5, 4.2, 1.2), family = "sans", las = 1)
    plot(d$yi, d$sei, xlim = xl + c(-pad, pad), ylim = c(ym, 0), pch = 15, col = "#B5B5B5", cex = 1.15, xlab = "Log Hazard Ratio", ylab = "Standard Error", main = paste0("Funnel plot:  ", num, ". ", label), bty = "l")
    polygon(c(m$mu - 1.96 * ym, m$mu, m$mu + 1.96 * ym), c(ym, 0, ym), col = "#EFEFEF", border = NA)
    lines(m$mu - 1.96 * s, s, lty = 2, col = "#777777")
    lines(m$mu + 1.96 * s, s, lty = 2, col = "#777777")
    abline(v = m$mu, col = "red", lwd = 1.4)
    points(d$yi, d$sei, pch = 15, col = "#B5B5B5", cex = 1.15)
    mtext(ep, side = 3, line = 0.65, cex = 0.78, col = "#444444")
}
d<-prepare_effects(as.data.frame(embedded_input))
category_forest(d,"PFS",'forest.jpg')
for(g in unique(d$Contrast[order(d$ContrastOrder)])){z<-d[d$Contrast==g,,drop=FALSE];stem<-gsub('[^A-Za-z0-9]+','_',g);if(nrow(z)>=3)category_sensitivity(z,g,paste0('sensitivity_',stem,'.jpg'));category_funnel(z,g,paste0('funnel_',stem,'.jpg'))}
```

**Supplementary Code S15.**BMI-cutoff meta-regression — OS

```
# Analysis: 15_cutoff_meta_regression_OS
# All analysis data are embedded. Running this file creates figures only.
rm(list = ls())
options(stringsAsFactors = FALSE, scipen = 999)
analysis_id <- "15_cutoff_meta_regression_OS"
script_arg <- grep('^--file=', commandArgs(trailingOnly = FALSE), value = TRUE)
script_file <- if (length(script_arg)) sub('^--file=', '', script_arg[1]) else NA_character_
if ((is.na(script_file) || !nzchar(script_file)) && interactive() && requireNamespace('rstudioapi', quietly = TRUE)) {
  p <- tryCatch(rstudioapi::getSourceEditorContext()$path, error = function(e) '')
  if (nzchar(p)) script_file <- p
}
script_dir <- if (!is.na(script_file) && nzchar(script_file)) dirname(normalizePath(script_file, winslash='/', mustWork=TRUE)) else normalizePath(getwd(), winslash='/', mustWork=TRUE)
output_dir <- file.path(script_dir, paste0(analysis_id, '_output'))
dir.create(output_dir, recursive=TRUE, showWarnings=FALSE)
setwd(output_dir)
.libPaths(unique(c(file.path(Sys.getenv('USERPROFILE'), 'R-library'), .libPaths())))
embedded_input <- structure(list(Study = c("Qi 2019", "Wang 2019", "Minami 2020",
"Ahmed 2021", "Collet 2021", "Imai 2021", "Akce 2021", "Esposito 2021",
"Zhang 2021", "Ged 2022", "Imai 2022", "McManus 2023", "Miyamoto 2023",
"Santoni 2023", "Alam 2024", "Wang 2024", "Yamamoto 2024"), Cutoff = c(24,
30, 18.5, 25, 25, 21.4, 25, 25, 25, 25, 22.1, 30, 20, 25, 30,
25, 25), Effect = c(0.496, 0.594, 0.55, 0.96, 0.75, 0.5, 0.44,
0.87, 0.62, 0.75, 0.56, 0.984, 1.08, 0.68, 0.94, 0.22, 0.596),
    LowerCI = c(0.26, 0.35, 0.24, 0.65, 0.51, 0.3, 0.18, 0.55,
    0.44, 0.5, 0.34, 0.498, 0.63, 0.48, 0.57, 0.11, 0.331), UpperCI = c(0.945,
    0.99, 1.27, 1.42, 1.1, 0.83, 1.09, 1.36, 0.87, 1.12, 0.91,
    1.941, 1.82, 0.95, 1.56, 0.45, 1.074)), row.names = c(NA,
-17L), class = "data.frame")


reml_meta <- function (yi, sei)
{
    keep <- is.finite(yi) & is.finite(sei) & sei > 0
    yi <- yi[keep]
    sei <- sei[keep]
    vi <- sei^2
    k <- length(yi)
    if (!k)
        stop("No valid estimates.")
    if (k == 1L) {
        z <- qnorm(0.975)
        return(list(k = 1L, mu = yi, se_mu = sei, ci_low = yi - z * sei, ci_high = yi + z * sei, p = 2 * pnorm(abs(yi/sei), lower.tail = FALSE), tau2 = NA_real_, Q = NA_real_, df_Q = 0L, Q_p = NA_real_, I2 = NA_real_, weights = 100))
    }
    wf <- 1/vi
    muf <- sum(wf * yi)/sum(wf)
    Q <- sum(wf * (yi - muf)^2)
    dfQ <- k - 1
    I2 <- if (Q > 0)
        max(0, (Q - dfQ)/Q) * 100
    else 0
    obj <- function(tau2) {
        w <- 1/(vi + tau2)
        mu <- sum(w * yi)/sum(w)
        0.5 * (sum(log(vi + tau2)) + log(sum(w)) + sum(w * (yi - mu)^2))
    }
    upper <- max(1, var(yi) * 10, max(vi) * 10)
    opt <- optimize(obj, c(0, upper), tol = 1e-12)
    tau2 <- if (opt$minimum < 1e-10)
        0
    else opt$minimum
    w <- 1/(vi + tau2)
    mu <- sum(w * yi)/sum(w)
    se <- sqrt(1/sum(w))
    ci <- mu + c(-1, 1) * qnorm(0.975) * se
    list(k = k, mu = mu, se_mu = se, ci_low = ci[1], ci_high = ci[2], p = 2 * pnorm(abs(mu/se), lower.tail = FALSE), tau2 = tau2, Q = Q, df_Q = dfQ, Q_p = pchisq(Q, dfQ, lower.tail = FALSE), I2 = I2, weights = w/sum(w) * 100)
}
prepare_effects <- function (d, effect_col = "Effect")
{
    d[[effect_col]] <- as.numeric(d[[effect_col]])
    d$LowerCI <- as.numeric(d$LowerCI)
    d$UpperCI <- as.numeric(d$UpperCI)
    ok <- is.finite(d[[effect_col]]) & is.finite(d$LowerCI) & is.finite(d$UpperCI) & d[[effect_col]] > 0 & d$LowerCI > 0 & d$UpperCI > d$LowerCI
    d <- d[ok, , drop = FALSE]
    d$Effect <- d[[effect_col]]
    d$yi <- log(d$Effect)
    d$sei <- (log(d$UpperCI) - log(d$LowerCI))/(2 * qnorm(0.975))
    d
}
meta_reg_plot <- function (d, endpoint, file)
{
    d$yi <- log(d$Effect)
    d$sei <- (log(d$UpperCI) - log(d$LowerCI))/(2 * qnorm(0.975))
    vi <- d$sei^2
    tau2 <- reml_meta(d$yi, d$sei)$tau2
    w <- 1/(vi + tau2)
    X <- cbind(1, (d$Cutoff - mean(d$Cutoff))/5)
    b <- solve(crossprod(X, w * X), crossprod(X, w * d$yi))
    V <- solve(crossprod(X, w * X))
    x <- seq(min(d$Cutoff), max(d$Cutoff), length.out = 200)
    Xp <- cbind(1, (x - mean(d$Cutoff))/5)
    mu <- as.vector(Xp %*% b)
    se <- sqrt(rowSums((Xp %*% V) * Xp))
    jpeg(file, width = 7.2, height = 5.3, units = "in", res = 300, quality = 100)
    on.exit(dev.off(), add = TRUE)
    par(mar = c(4.8, 5, 3.1, 1.2), family = "sans", las = 1)
    plot(d$Cutoff, exp(d$yi), type = "n", log = "y", xlab = expression("BMI cutoff (kg/m"^2 * ")"), ylab = "Hazard ratio", main = paste("BMI cutoff meta-regression -", endpoint), bty = "l")
    polygon(c(x, rev(x)), c(exp(mu - 1.96 * se), rev(exp(mu + 1.96 * se))), col = "#CFE6F2", border = NA)
    lines(x, exp(mu), col = "#4E8FB5", lwd = 2.5)
    points(d$Cutoff, exp(d$yi), pch = 21, bg = "#9CC5DB", col = "#315B73", cex = 0.8 + 2 * sqrt(w/max(w)))
    abline(h = 1, lty = 2, col = "#6B7280")
}
d<-prepare_effects(as.data.frame(embedded_input));meta_reg_plot(d,"OS",'meta_regression.jpg')
```

**Supplementary Code S16.**BMI-cutoff meta-regression — PFS

```
# Analysis: 16_cutoff_meta_regression_PFS
# All analysis data are embedded. Running this file creates figures only.
rm(list = ls())
options(stringsAsFactors = FALSE, scipen = 999)
analysis_id <- "16_cutoff_meta_regression_PFS"
script_arg <- grep('^--file=', commandArgs(trailingOnly = FALSE), value = TRUE)
script_file <- if (length(script_arg)) sub('^--file=', '', script_arg[1]) else NA_character_
if ((is.na(script_file) || !nzchar(script_file)) && interactive() && requireNamespace('rstudioapi', quietly = TRUE)) {
  p <- tryCatch(rstudioapi::getSourceEditorContext()$path, error = function(e) '')
  if (nzchar(p)) script_file <- p
}
script_dir <- if (!is.na(script_file) && nzchar(script_file)) dirname(normalizePath(script_file, winslash='/', mustWork=TRUE)) else normalizePath(getwd(), winslash='/', mustWork=TRUE)
output_dir <- file.path(script_dir, paste0(analysis_id, '_output'))
dir.create(output_dir, recursive=TRUE, showWarnings=FALSE)
setwd(output_dir)
.libPaths(unique(c(file.path(Sys.getenv('USERPROFILE'), 'R-library'), .libPaths())))
embedded_input <- structure(list(Study = c("Wang 2019", "Minami 2020", "Ahmed 2021",
"Akce 2021", "Esposito 2021", "Zhang 2021", "Chen 2022", "Imai 2022",
"Tateishi 2022", "Incorvaia 2023", "McManus 2023", "Miyamoto 2023",
"Alam 2024", "Tan 2024", "Wang 2024"), Cutoff = c(30, 18.5, 25,
25, 25, 25, 24.9, 22.1, 25, 30, 30, 20, 30, 24, 25), Effect = c(0.61,
0.9, 0.87, 1.04, 0.8, 0.76, 0.47, 0.78, 1, 0.18, 1.859, 1.18,
0.94, 0.022, 0.32), LowerCI = c(0.42, 0.45, 0.59, 0.56, 0.54,
0.55, 0.22, 0.51, 0.4, 0.03, 1.156, 0.76, 0.62, 0.001, 0.17),
    UpperCI = c(0.89, 1.82, 1.28, 1.92, 1.19, 1.04, 0.98, 1.19,
    2.45, 0.93, 2.989, 1.82, 1.44, 0.326, 0.59)), row.names = c(NA,
-15L), class = "data.frame")


reml_meta <- function (yi, sei)
{
    keep <- is.finite(yi) & is.finite(sei) & sei > 0
    yi <- yi[keep]
    sei <- sei[keep]
    vi <- sei^2
    k <- length(yi)
    if (!k)
        stop("No valid estimates.")
    if (k == 1L) {
        z <- qnorm(0.975)
        return(list(k = 1L, mu = yi, se_mu = sei, ci_low = yi - z * sei, ci_high = yi + z * sei, p = 2 * pnorm(abs(yi/sei), lower.tail = FALSE), tau2 = NA_real_, Q = NA_real_, df_Q = 0L, Q_p = NA_real_, I2 = NA_real_, weights = 100))
    }
    wf <- 1/vi
    muf <- sum(wf * yi)/sum(wf)
    Q <- sum(wf * (yi - muf)^2)
    dfQ <- k - 1
    I2 <- if (Q > 0)
        max(0, (Q - dfQ)/Q) * 100
    else 0
    obj <- function(tau2) {
        w <- 1/(vi + tau2)
        mu <- sum(w * yi)/sum(w)
        0.5 * (sum(log(vi + tau2)) + log(sum(w)) + sum(w * (yi - mu)^2))
    }
    upper <- max(1, var(yi) * 10, max(vi) * 10)
    opt <- optimize(obj, c(0, upper), tol = 1e-12)
    tau2 <- if (opt$minimum < 1e-10)
        0
    else opt$minimum
    w <- 1/(vi + tau2)
    mu <- sum(w * yi)/sum(w)
    se <- sqrt(1/sum(w))
    ci <- mu + c(-1, 1) * qnorm(0.975) * se
    list(k = k, mu = mu, se_mu = se, ci_low = ci[1], ci_high = ci[2], p = 2 * pnorm(abs(mu/se), lower.tail = FALSE), tau2 = tau2, Q = Q, df_Q = dfQ, Q_p = pchisq(Q, dfQ, lower.tail = FALSE), I2 = I2, weights = w/sum(w) * 100)
}
prepare_effects <- function (d, effect_col = "Effect")
{
    d[[effect_col]] <- as.numeric(d[[effect_col]])
    d$LowerCI <- as.numeric(d$LowerCI)
    d$UpperCI <- as.numeric(d$UpperCI)
    ok <- is.finite(d[[effect_col]]) & is.finite(d$LowerCI) & is.finite(d$UpperCI) & d[[effect_col]] > 0 & d$LowerCI > 0 & d$UpperCI > d$LowerCI
    d <- d[ok, , drop = FALSE]
    d$Effect <- d[[effect_col]]
    d$yi <- log(d$Effect)
    d$sei <- (log(d$UpperCI) - log(d$LowerCI))/(2 * qnorm(0.975))
    d
}
meta_reg_plot <- function (d, endpoint, file)
{
    d$yi <- log(d$Effect)
    d$sei <- (log(d$UpperCI) - log(d$LowerCI))/(2 * qnorm(0.975))
    vi <- d$sei^2
    tau2 <- reml_meta(d$yi, d$sei)$tau2
    w <- 1/(vi + tau2)
    X <- cbind(1, (d$Cutoff - mean(d$Cutoff))/5)
    b <- solve(crossprod(X, w * X), crossprod(X, w * d$yi))
    V <- solve(crossprod(X, w * X))
    x <- seq(min(d$Cutoff), max(d$Cutoff), length.out = 200)
    Xp <- cbind(1, (x - mean(d$Cutoff))/5)
    mu <- as.vector(Xp %*% b)
    se <- sqrt(rowSums((Xp %*% V) * Xp))
    jpeg(file, width = 7.2, height = 5.3, units = "in", res = 300, quality = 100)
    on.exit(dev.off(), add = TRUE)
    par(mar = c(4.8, 5, 3.1, 1.2), family = "sans", las = 1)
    plot(d$Cutoff, exp(d$yi), type = "n", log = "y", xlab = expression("BMI cutoff (kg/m"^2 * ")"), ylab = "Hazard ratio", main = paste("BMI cutoff meta-regression -", endpoint), bty = "l")
    polygon(c(x, rev(x)), c(exp(mu - 1.96 * se), rev(exp(mu + 1.96 * se))), col = "#CFE6F2", border = NA)
    lines(x, exp(mu), col = "#4E8FB5", lwd = 2.5)
    points(d$Cutoff, exp(d$yi), pch = 21, bg = "#9CC5DB", col = "#315B73", cex = 0.8 + 2 * sqrt(w/max(w)))
    abline(h = 1, lty = 2, col = "#6B7280")
}
d<-prepare_effects(as.data.frame(embedded_input));meta_reg_plot(d,"PFS",'meta_regression.jpg')
```

**Supplementary Code S17.**Linear dose-response analysis — adjusted OS

```
# Analysis: 17_dose_response_OS_adjusted
# All analysis data are embedded. Running this file creates figures only.
rm(list = ls())
options(stringsAsFactors = FALSE, scipen = 999)
analysis_id <- "17_dose_response_OS_adjusted"
script_arg <- grep('^--file=', commandArgs(trailingOnly = FALSE), value = TRUE)
script_file <- if (length(script_arg)) sub('^--file=', '', script_arg[1]) else NA_character_
if ((is.na(script_file) || !nzchar(script_file)) && interactive() && requireNamespace('rstudioapi', quietly = TRUE)) {
  p <- tryCatch(rstudioapi::getSourceEditorContext()$path, error = function(e) '')
  if (nzchar(p)) script_file <- p
}
script_dir <- if (!is.na(script_file) && nzchar(script_file)) dirname(normalizePath(script_file, winslash='/', mustWork=TRUE)) else normalizePath(getwd(), winslash='/', mustWork=TRUE)
output_dir <- file.path(script_dir, paste0(analysis_id, '_output'))
dir.create(output_dir, recursive=TRUE, showWarnings=FALSE)
setwd(output_dir)
.libPaths(unique(c(file.path(Sys.getenv('USERPROFILE'), 'R-library'), .libPaths())))
embedded_category_input <- structure(list(Study = c("Alam", "Bartl", "Martini", "Joshi",
"Rutkowski", "Magri", "Antoun", "Antoun", "Antoun", "Antoun",
"Antoun", "Antoun", "Antoun", "Antoun", "Cortellini", "Cortellini",
"Cortellini", "Cortellini", "Donnelly", "Donnelly", "Donnelly",
"Donnelly", "Donnelly", "Donnelly", "Donnelly", "Donnelly", "Donnelly",
"McQuade", "McQuade", "McQuade", "McQuade", "McQuade", "McQuade",
"Naik", "Naik", "Naik", "Vithayathil", "Vithayathil", "Zhang",
"Zhang"), Year = c(2024, 2021, 2019, 2022, 2020, 2019, 2023,
2023, 2023, 2023, 2023, 2023, 2023, 2023, 2020, 2020, 2020, 2020,
2019, 2019, 2019, 2019, 2019, 2019, 2019, 2019, 2019, 2018, 2018,
2018, 2018, 2018, 2018, 2019, 2019, 2019, 2023, 2023, 2023, 2023
), Cohort = c("MCC cohort", "Gynecologic cohort", "Phase I cohort",
"First-line pembrolizumab cohort", "First-line ICI cohort", "Nivolumab cohort",
"Melanoma", "Melanoma", "Melanoma", "Melanoma", "NSCLC", "NSCLC",
"NSCLC", "NSCLC", "Pembrolizumab cohort", "Pembrolizumab cohort",
"Pembrolizumab cohort", "Pembrolizumab cohort", "Anti-CTLA-4",
"Anti-CTLA-4", "Anti-CTLA-4", "Anti-PD-1", "Anti-PD-1", "Anti-PD-1",
"Combination ICI", "Combination ICI", "Combination ICI", "Ipilimumab plus dacarbazine",
"Ipilimumab plus dacarbazine", "Ipilimumab plus dacarbazine",
"Pembrolizumab nivolumab or atezolizumab", "Pembrolizumab nivolumab or atezolizumab",
"Pembrolizumab nivolumab or atezolizumab", "Anti-PD-1 cohort",
"Anti-PD-1 cohort", "Anti-PD-1 cohort", "Atezolizumab plus bevacizumab",
"Atezolizumab plus bevacizumab", "HNSCC cohort", "HNSCC cohort"
), DataType = c("direct_continuous", "direct_continuous", "direct_continuous",
"direct_continuous", "direct_continuous", "direct_continuous",
"categorical", "categorical", "categorical", "categorical", "categorical",
"categorical", "categorical", "categorical", "categorical", "categorical",
"categorical", "categorical", "categorical", "categorical", "categorical",
"categorical", "categorical", "categorical", "categorical", "categorical",
"categorical", "categorical", "categorical", "categorical", "categorical",
"categorical", "categorical", "categorical", "categorical", "categorical",
"binary", "binary", "binary", "binary"), Method = c("Direct model",
"Direct model", "Direct model", "Direct model", "Direct model",
"Direct model", "Independent WLS", "Independent WLS", "Independent WLS",
"Independent WLS", "Independent WLS", "Independent WLS", "Independent WLS",
"Independent WLS", "Independent WLS", "Independent WLS", "Independent WLS",
"Independent WLS", "Independent WLS", "Independent WLS", "Independent WLS",
"Independent WLS", "Independent WLS", "Independent WLS", "Independent WLS",
"Independent WLS", "Independent WLS", "Hamling GLS", "Hamling GLS",
"Hamling GLS", "Hamling GLS", "Hamling GLS", "Hamling GLS", "Independent WLS",
"Independent WLS", "Independent WLS", "Two-group slope", "Two-group slope",
"Two-group slope", "Two-group slope"), Dose = c(NA, NA, NA, NA,
NA, NA, 15.25, 21.75, 27.5, 32.5, 15.25, 21.75, 27.5, 32.5, 15.25,
21.75, 27.5, 32.5, 22.5, 27.5, 32.5, 22.5, 27.5, 32.5, 22.5,
27.5, 32.5, 21.75, 27.5, 32.5, 21.75, 27.5, 32.5, 22.6, 29.1,
40.1, 21.9, 29.6, 16.5, 21.6), Reference = c("No", "No", "No",
"No", "No", "No", "No", "Yes", "No", "No", "No", "Yes", "No",
"No", "No", "Yes", "No", "No", "Yes", "No", "No", "Yes", "No",
"No", "Yes", "No", "No", "Yes", "No", "No", "Yes", "No", "No",
"Yes", "No", "No", "Yes", "No", "No", "Yes"), HR = c(0.96, 7.44,
0.92, 0.984, 1.02, 1.19, 0.53, 1, 0.8, 0.85, 1.17, 1, 1.38, 1.54,
1.22, 1, 0.97, 0.7, 1, 0.83, 1.22, 1, 1.6, 1.04, 1, 0.7, 0.92,
1, 0.7, 0.54, 1, 0.78, 0.72, 1, 0.26, 0.42, 1, 0.72, 6.862, 1
), LowerCI = c(0.79, 1.62, 0.87, 0.914, 0.99, 0.93, 0.26, NA,
0.54, 0.47, 0.78, NA, 0.89, 0.73, 0.74, NA, 0.77, 0.49, NA, 0.47,
0.75, NA, 0.86, 0.48, NA, 0.2, 0.24, NA, 0.48, 0.34, NA, 0.52,
0.48, NA, 0.1, 0.1, NA, 0.42, 1.566, NA), UpperCI = c(1.17, 34.16,
0.97, 1.061, 1.05, 1.51, 1.02, NA, 1.16, 1.56, 1.76, NA, 2.14,
3.28, 2.01, NA, 1.22, 0.99, NA, 1.47, 2, NA, 2.96, 2.24, NA,
2.47, 3.49, NA, 1.03, 0.86, NA, 1.17, 1.06, NA, 0.71, 1.77, NA,
1.23, 30.064, NA), N = c(183, 36, 82, 136, 393, 46, NA, NA, NA,
NA, NA, NA, NA, NA, 35, 467, 232, 106, 64, 75, 61, 49, 55, 41,
26, 35, 17, 68, 88, 51, 102, 109, 118, 35, 86, 15, 97, 94, 8,
30), Events = c(NA, NA, NA, NA, NA, NA, NA, NA, NA, NA, NA, NA,
NA, NA, NA, NA, NA, NA, NA, NA, NA, NA, NA, NA, NA, NA, NA, 56,
67, 35, 59, 51, 52, NA, 27, 9, NA, NA, NA, NA), Increment = c(5,
5, 1, 1, 1, 1, NA, NA, NA, NA, NA, NA, NA, NA, NA, NA, NA, NA,
NA, NA, NA, NA, NA, NA, NA, NA, NA, NA, NA, NA, NA, NA, NA, NA,
NA, NA, NA, NA, NA, NA)), row.names = c(NA, -40L), class = "data.frame")

embedded_gls_reconstruction <- structure(list(Study = c("McQuade", "McQuade"), Year = c(2018,
2018), Cohort = c("Ipilimumab plus dacarbazine", "Pembrolizumab nivolumab or atezolizumab"
), logHR5 = c(-0.293679818852964, -0.158240679433613), SE5 = c(0.0914804815048405,
0.0928745445452891), Method = c("Hamling covariance reconstruction",
"Hamling covariance reconstruction")), row.names = c(NA, -2L), class = "data.frame")


if(!requireNamespace('metafor',quietly=TRUE))stop("Package 'metafor' is required.")
reml_meta <- function (yi, sei)
{
    keep <- is.finite(yi) & is.finite(sei) & sei > 0
    yi <- yi[keep]
    sei <- sei[keep]
    vi <- sei^2
    k <- length(yi)
    if (!k)
        stop("No valid estimates.")
    if (k == 1L) {
        z <- qnorm(0.975)
        return(list(k = 1L, mu = yi, se_mu = sei, ci_low = yi - z * sei, ci_high = yi + z * sei, p = 2 * pnorm(abs(yi/sei), lower.tail = FALSE), tau2 = NA_real_, Q = NA_real_, df_Q = 0L, Q_p = NA_real_, I2 = NA_real_, weights = 100))
    }
    wf <- 1/vi
    muf <- sum(wf * yi)/sum(wf)
    Q <- sum(wf * (yi - muf)^2)
    dfQ <- k - 1
    I2 <- if (Q > 0)
        max(0, (Q - dfQ)/Q) * 100
    else 0
    obj <- function(tau2) {
        w <- 1/(vi + tau2)
        mu <- sum(w * yi)/sum(w)
        0.5 * (sum(log(vi + tau2)) + log(sum(w)) + sum(w * (yi - mu)^2))
    }
    upper <- max(1, var(yi) * 10, max(vi) * 10)
    opt <- optimize(obj, c(0, upper), tol = 1e-12)
    tau2 <- if (opt$minimum < 1e-10)
        0
    else opt$minimum
    w <- 1/(vi + tau2)
    mu <- sum(w * yi)/sum(w)
    se <- sqrt(1/sum(w))
    ci <- mu + c(-1, 1) * qnorm(0.975) * se
    list(k = k, mu = mu, se_mu = se, ci_low = ci[1], ci_high = ci[2], p = 2 * pnorm(abs(mu/se), lower.tail = FALSE), tau2 = tau2, Q = Q, df_Q = dfQ, Q_p = pchisq(Q, dfQ, lower.tail = FALSE), I2 = I2, weights = w/sum(w) * 100)
}
leave_one_out <- function (d)
{
    if (nrow(d) < 3)
        return(data.frame())
    do.call(rbind, lapply(seq_len(nrow(d)), function(i) {
        m <- reml_meta(d$yi[-i], d$sei[-i])
        data.frame(Study = d$Study[i], Effect = exp(m$mu), LowerCI = exp(m$ci_low), UpperCI = exp(m$ci_high))
    }))
}
sensitivity_highlow <- function (loo, endpoint, adjustment, file)
{
    k <- nrow(loo)
    jpeg(file, width = 10, height = max(7.5, 3.6 + 0.25 * k), units = "in", res = 300, quality = 100)
    on.exit(dev.off(), add = TRUE)
    par(mar = c(4.8, 0.8, 2.2, 0.8), family = "sans", xpd = NA)
    plot.new()
    plot.window(xlim = c(0, 1), ylim = c(-1.7, k + 2.5))
    xs <- 0.03
    pl <- 0.42
    pr <- 0.7
    xhr <- 0.76
    xci <- 0.89
    amin <- 10^floor(log10(min(0.5, min(loo$LowerCI) * 0.9)))
    amax <- 10^ceiling(log10(max(2, max(loo$UpperCI) * 1.1)))
    ticks <- c(0.01, 0.05, 0.1, 0.2, 0.5, 1, 2, 5, 10, 100)
    ticks <- ticks[ticks >= amin & ticks <= amax]
    map <- function(v) pl + (log(v) - log(amin))/(log(amax) - log(amin)) * (pr - pl)
    y <- rev(seq_len(k)) + 0.6
    text(xs, k + 1.7, "Omitted study", adj = 0, font = 2, cex = 0.83)
    text((pl + pr)/2, k + 1.7, "Pooled Hazard Ratio", font = 2, cex = 0.83)
    text(xhr, k + 1.7, "HR", adj = 1, font = 2, cex = 0.83)
    text(xci, k + 1.7, "95% CI", font = 2, cex = 0.83)
    segments(map(1), -0.1, map(1), k + 1.3, lwd = 0.8)
    for (i in seq_len(k)) {
        segments(map(loo$LowerCI[i]), y[i], map(loo$UpperCI[i]), y[i], lwd = 0.85)
        points(map(loo$Effect[i]), y[i], pch = 15, cex = 1.15, col = "#005AA8")
        text(xs, y[i], loo$Study[i], adj = 0, cex = 0.69)
        text(xhr, y[i], sprintf("%.2f", loo$Effect[i]), adj = 1, cex = 0.7)
        text(xci, y[i], sprintf("[%.2f; %.2f]", loo$LowerCI[i], loo$UpperCI[i]), cex = 0.7)
    }
    ay <- -0.15
    segments(pl, ay, pr, ay)
    for (t in ticks) {
        segments(map(t), ay, map(t), ay - 0.15)
        text(map(t), ay - 0.38, format(t, trim = TRUE), cex = 0.67)
    }
    text((pl + map(1))/2, ay - 1, "High BMI", font = 2, cex = 0.65)
    text((map(1) + pr)/2, ay - 1, "Low BMI", font = 2, cex = 0.65)
    mtext(sprintf("High BMI vs Low BMI — leave-one-out sensitivity analysis — %s, %s estimates", endpoint, tolower(adjustment)), side = 3, line = 0.5, font = 2, cex = 0.86)
}
funnel_plot <- function (d, m, title, xlab, file, note = TRUE)
{
    jpeg(file, width = 8, height = 7, units = "in", res = 300, quality = 100)
    on.exit(dev.off(), add = TRUE)
    par(mar = c(5, 5, 3.3, 1.2), family = "sans", las = 1)
    ym <- max(d$sei) * 1.08
    xc <- c(d$yi, m$mu - 1.96 * ym, m$mu + 1.96 * ym)
    pad <- diff(range(xc)) * 0.08
    plot(d$yi, d$sei, xlim = range(xc) + c(-pad, pad), ylim = c(ym, 0), pch = 21, bg = if (grepl("hazard", tolower(xlab)))
        "#2F80C0"
    else "#D0D0D0", col = if (grepl("hazard", tolower(xlab)))
        "#004C99"
    else "black", cex = 1.15, xlab = xlab, ylab = "Standard error", main = title, bty = "l")
    s <- seq(0, ym, length.out = 200)
    lines(m$mu - 1.96 * s, s, lty = 2, col = "#707070")
    lines(m$mu + 1.96 * s, s, lty = 2, col = "#707070")
    abline(v = m$mu, col = if (grepl("hazard", tolower(xlab)))
        "#D73027"
    else "#333333", lty = if (grepl("hazard", tolower(xlab)))
        1
    else 3, lwd = 1.2)
    if (note) {
        if (nrow(d) < 10)
            mtext(sprintf("Exploratory only; Egger test not performed (k = %d < 10).", nrow(d)), side = 1, line = 3.7, cex = 0.78)
    }
}
endpoint<-"OS";d<-as.data.frame(embedded_category_input);gls<-as.data.frame(embedded_gls_reconstruction);z975<-qnorm(.975);for(n in c('Dose','HR','LowerCI','UpperCI','Increment'))d[[n]]<-as.numeric(d[[n]])
calc<-function(z){method<-z$Method[1];study<-z$Study[1];year<-z$Year[1];cohort<-z$Cohort[1];if(grepl('Hamling',method)){g<-gls[gls$Study==study&gls$Year==year&gls$Cohort==cohort,,drop=FALSE];return(data.frame(Study=study,Year=year,Cohort=cohort,logHR5=g$logHR5,SE5=g$SE5))};if(z$DataType[1]=='direct_continuous'){r<-z[1,];fac<-5/r$Increment;return(data.frame(Study=study,Year=year,Cohort=cohort,logHR5=log(r$HR)*fac,SE5=(log(r$UpperCI)-log(r$LowerCI))/(2*z975)*fac))};ref<-which(tolower(z$Reference)=='yes');non<-setdiff(seq_len(nrow(z)),ref);xx<-(z$Dose[non]-z$Dose[ref])/5;yy<-log(z$HR[non]);ss<-(log(z$UpperCI[non])-log(z$LowerCI[non]))/(2*z975);ww<-1/ss^2;data.frame(Study=study,Year=year,Cohort=cohort,logHR5=sum(ww*xx*yy)/sum(ww*xx^2),SE5=sqrt(1/sum(ww*xx^2)))}
cohort<-do.call(rbind,lapply(split(d,interaction(d$Study,d$Year,d$Cohort,drop=TRUE)),calc));combine<-function(z){w<-1/z$SE5^2;data.frame(Study=z$Study[1],Year=z$Year[1],logHR5=sum(w*z$logHR5)/sum(w),SE5=sqrt(1/sum(w)))};study<-do.call(rbind,lapply(split(cohort,interaction(cohort$Study,cohort$Year,drop=TRUE)),combine));study<-study[order(study$Year,study$Study),];fit<-metafor::rma.uni(yi=study$logHR5,sei=study$SE5,method='REML',test='z')
jpeg('forest.jpg',width=7.6,height=max(7,3.4+.43*nrow(study)),units='in',res=300,quality=100);par(family='sans',mar=c(4.8,4.2,3.2,1.5));metafor::forest(fit,slab=paste(study$Study,study$Year),atransf=exp,xlab=expression('Hazard ratio per 5 kg/m'^2*' increase in BMI'),mlab='Random-effects model (REML)',header=c('Study','HR [95% CI]'),col='#4E8FB5',border='#315B73',cex=.84);title(paste('Linear dose–response association between BMI and',endpoint),line=1);dev.off()
bmi<-seq(15,40,.1);xx<-(bmi-25)/5;b<-as.numeric(fit$b);se<-fit$se;hr<-exp(b*xx);a<-exp((b-z975*se)*xx);zz<-exp((b+z975*se)*xx);lo<-pmin(a,zz);hi<-pmax(a,zz);jpeg('linear_dose_response.jpg',width=7.2,height=5.2,units='in',res=300,quality=100);par(mar=c(4.8,5,3.1,1.2),family='sans',las=1);plot(bmi,hr,type='n',log='y',ylim=range(c(lo,hi)),xlab=expression('BMI (kg/m'^2*')'),ylab=if(endpoint=='OS')'Relative hazard of death (reference BMI = 25)' else 'Relative hazard of progression or death (reference BMI = 25)',main=paste('Linear dose–response association between BMI and',endpoint),bty='l');polygon(c(bmi,rev(bmi)),c(lo,rev(hi)),col='#BFDDEA99',border=NA);lines(bmi,hr,col='#4E8FB5',lwd=2.6);abline(h=1,lty=2,col='#6B7280');abline(v=25,lty=3,col='#94A3B8');dev.off()
loo<-metafor::leave1out(fit);lr<-data.frame(Study=study$Study,Effect=exp(loo$estimate),LowerCI=exp(loo$ci.lb),UpperCI=exp(loo$ci.ub));sensitivity_highlow(lr,endpoint,'Adjusted dose-response','sensitivity_leave_one_out.jpg');fd<-data.frame(yi=study$logHR5,sei=study$SE5);funnel_plot(fd,list(mu=as.numeric(fit$b)),paste(endpoint,'dose-response funnel plot'),'Log hazard ratio per 5-unit BMI increase','funnel.jpg',TRUE)
```

**Supplementary Code S18.**Linear dose-response analysis — adjusted PFS

```
# Analysis: 18_dose_response_PFS_adjusted
# All analysis data are embedded. Running this file creates figures only.
rm(list = ls())
options(stringsAsFactors = FALSE, scipen = 999)
analysis_id <- "18_dose_response_PFS_adjusted"
script_arg <- grep('^--file=', commandArgs(trailingOnly = FALSE), value = TRUE)
script_file <- if (length(script_arg)) sub('^--file=', '', script_arg[1]) else NA_character_
if ((is.na(script_file) || !nzchar(script_file)) && interactive() && requireNamespace('rstudioapi', quietly = TRUE)) {
  p <- tryCatch(rstudioapi::getSourceEditorContext()$path, error = function(e) '')
  if (nzchar(p)) script_file <- p
}
script_dir <- if (!is.na(script_file) && nzchar(script_file)) dirname(normalizePath(script_file, winslash='/', mustWork=TRUE)) else normalizePath(getwd(), winslash='/', mustWork=TRUE)
output_dir <- file.path(script_dir, paste0(analysis_id, '_output'))
dir.create(output_dir, recursive=TRUE, showWarnings=FALSE)
setwd(output_dir)
.libPaths(unique(c(file.path(Sys.getenv('USERPROFILE'), 'R-library'), .libPaths())))
embedded_category_input <- structure(list(Study = c("McQuade", "McQuade", "McQuade", "McQuade",
"McQuade", "McQuade", "Martini", "Cortellini", "Cortellini",
"Cortellini", "Donnelly", "Donnelly", "Donnelly", "Donnelly",
"Donnelly", "Donnelly", "Donnelly", "Donnelly", "Donnelly", "Naik",
"Naik", "Naik", "Cortellini", "Cortellini", "Cortellini", "Cortellini",
"Rutkowski", "Bartl", "Vithayathil", "Vithayathil", "Zhang",
"Zhang", "Alam"), Year = c(2018, 2018, 2018, 2018, 2018, 2018,
2019, 2019, 2019, 2019, 2019, 2019, 2019, 2019, 2019, 2019, 2019,
2019, 2019, 2019, 2019, 2019, 2020, 2020, 2020, 2020, 2020, 2021,
2023, 2023, 2023, 2023, 2024), Cohort = c("Ipilimumab plus dacarbazine",
"Ipilimumab plus dacarbazine", "Ipilimumab plus dacarbazine",
"Pembrolizumab, nivolumab, or atezolizumab", "Pembrolizumab, nivolumab, or atezolizumab",
"Pembrolizumab, nivolumab, or atezolizumab", "Phase I cohort",
"Pan-cancer anti-PD-1/PD-L1 cohort", "Pan-cancer anti-PD-1/PD-L1 cohort",
"Pan-cancer anti-PD-1/PD-L1 cohort", "Anti-CTLA-4", "Anti-CTLA-4",
"Anti-CTLA-4", "Anti-PD-1", "Anti-PD-1", "Anti-PD-1", "Combination ICI",
"Combination ICI", "Combination ICI", "Anti-PD-1 cohort", "Anti-PD-1 cohort",
"Anti-PD-1 cohort", "First-line pembrolizumab cohort", "First-line pembrolizumab cohort",
"First-line pembrolizumab cohort", "First-line pembrolizumab cohort",
"First-line ICI cohort", "Gynecologic cohort", "Atezolizumab plus bevacizumab",
"Atezolizumab plus bevacizumab", "HNSCC cohort", "HNSCC cohort",
"Merkel cell carcinoma cohort"), DataType = c("categorical",
"categorical", "categorical", "categorical", "categorical", "categorical",
"direct_continuous", "categorical", "categorical", "categorical",
"categorical", "categorical", "categorical", "categorical", "categorical",
"categorical", "categorical", "categorical", "categorical", "categorical",
"categorical", "categorical", "categorical", "categorical", "categorical",
"categorical", "direct_continuous", "direct_continuous", "binary",
"binary", "binary", "binary", "direct_continuous"), Method = c("Hamling GLS",
"Hamling GLS", "Hamling GLS", "Hamling GLS", "Hamling GLS", "Hamling GLS",
"Direct model", "Hamling GLS", "Hamling GLS", "Hamling GLS",
"Independent WLS", "Independent WLS", "Independent WLS", "Independent WLS",
"Independent WLS", "Independent WLS", "Independent WLS", "Independent WLS",
"Independent WLS", "Independent WLS", "Independent WLS", "Independent WLS",
"Independent WLS", "Independent WLS", "Independent WLS", "Independent WLS",
"Direct model", "Direct model; reciprocal", "Two-group slope",
"Two-group slope", "Two-group slope; reciprocal", "Two-group slope; reciprocal",
"Direct model"), Dose = c(21.75, 27.5, 32.5, 21.75, 27.5, 32.5,
NA, 21.75, 27.5, 32.5, 22.5, 27.5, 32.5, 22.5, 27.5, 32.5, 22.5,
27.5, 32.5, 22.6, 29.1, 40.1, 15.25, 21.75, 27.5, 32.5, NA, NA,
21.9, 29.6, 16.5, 21.6, NA), Reference = c("Yes", "No", "No",
"Yes", "No", "No", NA, "Yes", "No", "No", "Yes", "No", "No",
"Yes", "No", "No", "Yes", "No", "No", "Yes", "No", "No", "No",
"Yes", "No", "No", NA, NA, "Yes", "No", "Yes", "No", NA), HR = c(1,
0.7, 0.54, 1, 0.78, 0.72, 0.96, 1, 0.68, 0.72, 1, 0.97, 1.16,
1, 2.34, 2.46, 1, 0.5, 0.18, 1, 0.43, 1, 1.46, 1, 1.04, 0.61,
1, 0.2680965, 1, 0.7, 1, 0.1763047, 0.95), LowerCI = c(NA, 0.48,
0.34, NA, 0.52, 0.48, 0.92, NA, 0.53, 0.49, NA, 0.56, 0.7, NA,
1.05, 1.03, NA, 0.15, 0.05, NA, 0.19, 0.34, 0.98, NA, 0.85, 0.45,
0.98, 0.1176471, NA, 0.41, NA, 0.042398, 0.8), UpperCI = c(NA,
1.03, 0.86, NA, 1.17, 1.06, 1, NA, 0.87, 1.06, NA, 1.69, 1.94,
NA, 5.2, 5.89, NA, 1.71, 0.74, NA, 0.95, 2.94, 2.2, NA, 1.26,
0.82, 1.03, 0.6134969, NA, 1.2, NA, 0.7331378, 1.12), N = c(68,
88, 51, 102, 109, 118, 82, 452, 377, 107, 64, 75, 61, 49, 55,
41, 26, 35, 17, 35, 86, 15, 35, 467, 232, 106, 435, 36, 97, 94,
8, 30, 169), Events = c(56, 67, 35, 59, 51, 52, NA, 322, 223,
63, NA, NA, NA, NA, NA, NA, NA, NA, NA, NA, NA, NA, NA, NA, NA,
NA, 322, NA, NA, NA, NA, NA, NA), Increment = c(NA, NA, NA, NA,
NA, NA, 1, NA, NA, NA, NA, NA, NA, NA, NA, NA, NA, NA, NA, NA,
NA, NA, NA, NA, NA, NA, 1, 5, NA, NA, NA, NA, 5)), row.names = c(NA,
-33L), class = "data.frame")

embedded_gls_reconstruction <- structure(list(Study = c("McQuade", "McQuade", "Cortellini"),
    Year = c(2018, 2018, 2019), Cohort = c("Ipilimumab plus dacarbazine",
    "Pembrolizumab, nivolumab, or atezolizumab", "Pan-cancer anti-PD-1/PD-L1 cohort"
    ), logHR5 = c(-0.293679818852964, -0.158240679433613, -0.225095854528703
    ), SE5 = c(0.0914804815048405, 0.0928745445452891, 0.0747497894153837
    ), Method = c("Hamling covariance reconstruction", "Hamling covariance reconstruction",
    "Hamling covariance reconstruction")), row.names = c(NA,
-3L), class = "data.frame")


if(!requireNamespace('metafor',quietly=TRUE))stop("Package 'metafor' is required.")
reml_meta <- function (yi, sei)
{
    keep <- is.finite(yi) & is.finite(sei) & sei > 0
    yi <- yi[keep]
    sei <- sei[keep]
    vi <- sei^2
    k <- length(yi)
    if (!k)
        stop("No valid estimates.")
    if (k == 1L) {
        z <- qnorm(0.975)
        return(list(k = 1L, mu = yi, se_mu = sei, ci_low = yi - z * sei, ci_high = yi + z * sei, p = 2 * pnorm(abs(yi/sei), lower.tail = FALSE), tau2 = NA_real_, Q = NA_real_, df_Q = 0L, Q_p = NA_real_, I2 = NA_real_, weights = 100))
    }
    wf <- 1/vi
    muf <- sum(wf * yi)/sum(wf)
    Q <- sum(wf * (yi - muf)^2)
    dfQ <- k - 1
    I2 <- if (Q > 0)
        max(0, (Q - dfQ)/Q) * 100
    else 0
    obj <- function(tau2) {
        w <- 1/(vi + tau2)
        mu <- sum(w * yi)/sum(w)
        0.5 * (sum(log(vi + tau2)) + log(sum(w)) + sum(w * (yi - mu)^2))
    }
    upper <- max(1, var(yi) * 10, max(vi) * 10)
    opt <- optimize(obj, c(0, upper), tol = 1e-12)
    tau2 <- if (opt$minimum < 1e-10)
        0
    else opt$minimum
    w <- 1/(vi + tau2)
    mu <- sum(w * yi)/sum(w)
    se <- sqrt(1/sum(w))
    ci <- mu + c(-1, 1) * qnorm(0.975) * se
    list(k = k, mu = mu, se_mu = se, ci_low = ci[1], ci_high = ci[2], p = 2 * pnorm(abs(mu/se), lower.tail = FALSE), tau2 = tau2, Q = Q, df_Q = dfQ, Q_p = pchisq(Q, dfQ, lower.tail = FALSE), I2 = I2, weights = w/sum(w) * 100)
}
leave_one_out <- function (d)
{
    if (nrow(d) < 3)
        return(data.frame())
    do.call(rbind, lapply(seq_len(nrow(d)), function(i) {
        m <- reml_meta(d$yi[-i], d$sei[-i])
        data.frame(Study = d$Study[i], Effect = exp(m$mu), LowerCI = exp(m$ci_low), UpperCI = exp(m$ci_high))
    }))
}
sensitivity_highlow <- function (loo, endpoint, adjustment, file)
{
    k <- nrow(loo)
    jpeg(file, width = 10, height = max(7.5, 3.6 + 0.25 * k), units = "in", res = 300, quality = 100)
    on.exit(dev.off(), add = TRUE)
    par(mar = c(4.8, 0.8, 2.2, 0.8), family = "sans", xpd = NA)
    plot.new()
    plot.window(xlim = c(0, 1), ylim = c(-1.7, k + 2.5))
    xs <- 0.03
    pl <- 0.42
    pr <- 0.7
    xhr <- 0.76
    xci <- 0.89
    amin <- 10^floor(log10(min(0.5, min(loo$LowerCI) * 0.9)))
    amax <- 10^ceiling(log10(max(2, max(loo$UpperCI) * 1.1)))
    ticks <- c(0.01, 0.05, 0.1, 0.2, 0.5, 1, 2, 5, 10, 100)
    ticks <- ticks[ticks >= amin & ticks <= amax]
    map <- function(v) pl + (log(v) - log(amin))/(log(amax) - log(amin)) * (pr - pl)
    y <- rev(seq_len(k)) + 0.6
    text(xs, k + 1.7, "Omitted study", adj = 0, font = 2, cex = 0.83)
    text((pl + pr)/2, k + 1.7, "Pooled Hazard Ratio", font = 2, cex = 0.83)
    text(xhr, k + 1.7, "HR", adj = 1, font = 2, cex = 0.83)
    text(xci, k + 1.7, "95% CI", font = 2, cex = 0.83)
    segments(map(1), -0.1, map(1), k + 1.3, lwd = 0.8)
    for (i in seq_len(k)) {
        segments(map(loo$LowerCI[i]), y[i], map(loo$UpperCI[i]), y[i], lwd = 0.85)
        points(map(loo$Effect[i]), y[i], pch = 15, cex = 1.15, col = "#005AA8")
        text(xs, y[i], loo$Study[i], adj = 0, cex = 0.69)
        text(xhr, y[i], sprintf("%.2f", loo$Effect[i]), adj = 1, cex = 0.7)
        text(xci, y[i], sprintf("[%.2f; %.2f]", loo$LowerCI[i], loo$UpperCI[i]), cex = 0.7)
    }
    ay <- -0.15
    segments(pl, ay, pr, ay)
    for (t in ticks) {
        segments(map(t), ay, map(t), ay - 0.15)
        text(map(t), ay - 0.38, format(t, trim = TRUE), cex = 0.67)
    }
    text((pl + map(1))/2, ay - 1, "High BMI", font = 2, cex = 0.65)
    text((map(1) + pr)/2, ay - 1, "Low BMI", font = 2, cex = 0.65)
    mtext(sprintf("High BMI vs Low BMI — leave-one-out sensitivity analysis — %s, %s estimates", endpoint, tolower(adjustment)), side = 3, line = 0.5, font = 2, cex = 0.86)
}
funnel_plot <- function (d, m, title, xlab, file, note = TRUE)
{
    jpeg(file, width = 8, height = 7, units = "in", res = 300, quality = 100)
    on.exit(dev.off(), add = TRUE)
    par(mar = c(5, 5, 3.3, 1.2), family = "sans", las = 1)
    ym <- max(d$sei) * 1.08
    xc <- c(d$yi, m$mu - 1.96 * ym, m$mu + 1.96 * ym)
    pad <- diff(range(xc)) * 0.08
    plot(d$yi, d$sei, xlim = range(xc) + c(-pad, pad), ylim = c(ym, 0), pch = 21, bg = if (grepl("hazard", tolower(xlab)))
        "#2F80C0"
    else "#D0D0D0", col = if (grepl("hazard", tolower(xlab)))
        "#004C99"
    else "black", cex = 1.15, xlab = xlab, ylab = "Standard error", main = title, bty = "l")
    s <- seq(0, ym, length.out = 200)
    lines(m$mu - 1.96 * s, s, lty = 2, col = "#707070")
    lines(m$mu + 1.96 * s, s, lty = 2, col = "#707070")
    abline(v = m$mu, col = if (grepl("hazard", tolower(xlab)))
        "#D73027"
    else "#333333", lty = if (grepl("hazard", tolower(xlab)))
        1
    else 3, lwd = 1.2)
    if (note) {
        if (nrow(d) < 10)
            mtext(sprintf("Exploratory only; Egger test not performed (k = %d < 10).", nrow(d)), side = 1, line = 3.7, cex = 0.78)
    }
}
endpoint<-"PFS";d<-as.data.frame(embedded_category_input);gls<-as.data.frame(embedded_gls_reconstruction);z975<-qnorm(.975);for(n in c('Dose','HR','LowerCI','UpperCI','Increment'))d[[n]]<-as.numeric(d[[n]])
calc<-function(z){method<-z$Method[1];study<-z$Study[1];year<-z$Year[1];cohort<-z$Cohort[1];if(grepl('Hamling',method)){g<-gls[gls$Study==study&gls$Year==year&gls$Cohort==cohort,,drop=FALSE];return(data.frame(Study=study,Year=year,Cohort=cohort,logHR5=g$logHR5,SE5=g$SE5))};if(z$DataType[1]=='direct_continuous'){r<-z[1,];fac<-5/r$Increment;return(data.frame(Study=study,Year=year,Cohort=cohort,logHR5=log(r$HR)*fac,SE5=(log(r$UpperCI)-log(r$LowerCI))/(2*z975)*fac))};ref<-which(tolower(z$Reference)=='yes');non<-setdiff(seq_len(nrow(z)),ref);xx<-(z$Dose[non]-z$Dose[ref])/5;yy<-log(z$HR[non]);ss<-(log(z$UpperCI[non])-log(z$LowerCI[non]))/(2*z975);ww<-1/ss^2;data.frame(Study=study,Year=year,Cohort=cohort,logHR5=sum(ww*xx*yy)/sum(ww*xx^2),SE5=sqrt(1/sum(ww*xx^2)))}
cohort<-do.call(rbind,lapply(split(d,interaction(d$Study,d$Year,d$Cohort,drop=TRUE)),calc));combine<-function(z){w<-1/z$SE5^2;data.frame(Study=z$Study[1],Year=z$Year[1],logHR5=sum(w*z$logHR5)/sum(w),SE5=sqrt(1/sum(w)))};study<-do.call(rbind,lapply(split(cohort,interaction(cohort$Study,cohort$Year,drop=TRUE)),combine));study<-study[order(study$Year,study$Study),];fit<-metafor::rma.uni(yi=study$logHR5,sei=study$SE5,method='REML',test='z')
jpeg('forest.jpg',width=7.6,height=max(7,3.4+.43*nrow(study)),units='in',res=300,quality=100);par(family='sans',mar=c(4.8,4.2,3.2,1.5));metafor::forest(fit,slab=paste(study$Study,study$Year),atransf=exp,xlab=expression('Hazard ratio per 5 kg/m'^2*' increase in BMI'),mlab='Random-effects model (REML)',header=c('Study','HR [95% CI]'),col='#4E8FB5',border='#315B73',cex=.84);title(paste('Linear dose–response association between BMI and',endpoint),line=1);dev.off()
bmi<-seq(15,40,.1);xx<-(bmi-25)/5;b<-as.numeric(fit$b);se<-fit$se;hr<-exp(b*xx);a<-exp((b-z975*se)*xx);zz<-exp((b+z975*se)*xx);lo<-pmin(a,zz);hi<-pmax(a,zz);jpeg('linear_dose_response.jpg',width=7.2,height=5.2,units='in',res=300,quality=100);par(mar=c(4.8,5,3.1,1.2),family='sans',las=1);plot(bmi,hr,type='n',log='y',ylim=range(c(lo,hi)),xlab=expression('BMI (kg/m'^2*')'),ylab=if(endpoint=='OS')'Relative hazard of death (reference BMI = 25)' else 'Relative hazard of progression or death (reference BMI = 25)',main=paste('Linear dose–response association between BMI and',endpoint),bty='l');polygon(c(bmi,rev(bmi)),c(lo,rev(hi)),col='#BFDDEA99',border=NA);lines(bmi,hr,col='#4E8FB5',lwd=2.6);abline(h=1,lty=2,col='#6B7280');abline(v=25,lty=3,col='#94A3B8');dev.off()
loo<-metafor::leave1out(fit);lr<-data.frame(Study=study$Study,Effect=exp(loo$estimate),LowerCI=exp(loo$ci.lb),UpperCI=exp(loo$ci.ub));sensitivity_highlow(lr,endpoint,'Adjusted dose-response','sensitivity_leave_one_out.jpg');fd<-data.frame(yi=study$logHR5,sei=study$SE5);funnel_plot(fd,list(mu=as.numeric(fit$b)),paste(endpoint,'dose-response funnel plot'),'Log hazard ratio per 5-unit BMI increase','funnel.jpg',TRUE)
```

**Supplementary Code S19.**Subgroup analysis — adjusted OS

```
# Analysis: 19_subgroup_OS_adjusted
# All analysis data are embedded. Running this file creates figures only.
rm(list = ls())
options(stringsAsFactors = FALSE, scipen = 999)
analysis_id <- "19_subgroup_OS_adjusted"
script_arg <- grep('^--file=', commandArgs(trailingOnly = FALSE), value = TRUE)
script_file <- if (length(script_arg)) sub('^--file=', '', script_arg[1]) else NA_character_
if ((is.na(script_file) || !nzchar(script_file)) && interactive() && requireNamespace('rstudioapi', quietly = TRUE)) {
  p <- tryCatch(rstudioapi::getSourceEditorContext()$path, error = function(e) '')
  if (nzchar(p)) script_file <- p
}
script_dir <- if (!is.na(script_file) && nzchar(script_file)) dirname(normalizePath(script_file, winslash='/', mustWork=TRUE)) else normalizePath(getwd(), winslash='/', mustWork=TRUE)
output_dir <- file.path(script_dir, paste0(analysis_id, '_output'))
dir.create(output_dir, recursive=TRUE, showWarnings=FALSE)
setwd(output_dir)
.libPaths(unique(c(file.path(Sys.getenv('USERPROFILE'), 'R-library'), .libPaths())))
embedded_input <- structure(list(Study = c("McQuade (1) 2018", "McQuade (2) 2018",
"Qi 2019", "Martini 2019", "Cortellini 2019", "Giorgi 2019",
"Donnelly (1) 2019", "Donnelly (2) 2019", "Kichenadasse 2019",
"Magri 2019", "Wang 2019", "Naik 2019", "Cortellini 2020", "Rutkowski 2020",
"Minami 2020", "Ahmed 2021", "Bartl 2021", "Collet 2021", "Imai 2021",
"Akce 2021", "Esposito 2021", "Zhang 2021", "Ged 2022", "Imai 2022",
"Joshi 2022", "Wang 2022", "Jain 2023", "Vithayathil 2023", "Zhang 2023",
"McManus 2023", "Miyamoto 2023", "Antoun (1) 2023", "Antoun (2) 2023",
"Santoni 2023", "Alam 2024", "Gómez-Banoy 2024", "Nie (1) 2024",
"Nie (2) 2024", "Wang 2024", "Llerena 2024", "Yamamoto 2024",
"Mastrolonardo 2025"), CancerType = c("Melanoma", "Melanoma",
"Multiple/Other", "NSCLC", "Multiple/Other", "RCC", "Melanoma",
"Melanoma", "NSCLC", "NSCLC", "Multiple/Other", "Melanoma", "NSCLC",
"Melanoma", "NSCLC", "Multiple/Other", "Multiple/Other", "Multiple/Other",
"NSCLC", "HCC", "Multiple/Other", "Multiple/Other", "RCC", "NSCLC",
"NSCLC", "RCC", "NSCLC", "HCC", "HNSCC", "RCC", "HNSCC", "Multiple/Other",
"Multiple/Other", "RCC", "Multiple/Other", "Multiple/Other",
"NSCLC", "NSCLC", "HCC", "HNSCC", "HCC", "Multiple/Other"), StudyRegion = c("Others",
"Others", "Asia", "USA", "Europe", "Europe", "USA", "USA", "Others",
"Europe", "USA", "USA", "Others", "Others", "Asia", "USA", "Europe",
"Europe", "Asia", "USA", "Europe", "USA", "USA", "Asia", "USA",
"Asia", "USA", "Others", "Asia", "USA", "Asia", "Europe", "Europe",
"Others", "USA", "USA", "Others", "Others", "Asia", "USA", "Asia",
"USA"), ICIClass = c("Anti-CTLA-4", "Anti-PD-1/PD-L1", "Anti-PD-1/PD-L1",
"Anti-PD-1/PD-L1", "Anti-PD-1/PD-L1", "Anti-PD-1/PD-L1", "Anti-CTLA-4",
"Anti-PD-1/PD-L1", "Anti-PD-1/PD-L1", "Anti-PD-1/PD-L1", "Anti-PD-1/PD-L1",
"Anti-PD-1/PD-L1 + Anti-CTLA-4", "Anti-PD-1/PD-L1", "Anti-PD-1/PD-L1 + Anti-CTLA-4",
"Anti-PD-1/PD-L1", "Anti-PD-1/PD-L1 + Anti-CTLA-4", "Anti-PD-1/PD-L1",
"Anti-PD-1/PD-L1 + Anti-CTLA-4", "Anti-PD-1/PD-L1", "Anti-PD-1/PD-L1",
"Anti-PD-1/PD-L1", "Anti-PD-1/PD-L1", "Anti-PD-1/PD-L1 + Anti-CTLA-4",
"Anti-PD-1/PD-L1", "Anti-PD-1/PD-L1", "Anti-PD-1/PD-L1", "Anti-PD-1/PD-L1",
"Anti-PD-1/PD-L1", "Anti-PD-1/PD-L1", "Anti-PD-1/PD-L1 + Anti-CTLA-4",
"Anti-PD-1/PD-L1", "Anti-PD-1/PD-L1", "Anti-PD-1/PD-L1", "Anti-PD-1/PD-L1 + Anti-CTLA-4",
"Anti-PD-1/PD-L1 + Anti-CTLA-4", "Anti-PD-1/PD-L1", "Anti-PD-1/PD-L1",
"Anti-PD-1/PD-L1", "Anti-PD-1/PD-L1", "Anti-PD-1/PD-L1", "Anti-PD-1/PD-L1",
"Anti-PD-1/PD-L1"), Effect = c(0.54, 0.72, 0.496, 0.306, 0.5,
0.633, 1.22, 1.04, 0.69, 1.19, 0.594, 0.42, 0.7, 1.17, 0.55,
0.96, 0.13, 0.75, 0.5, 0.44, 0.87, 0.62, 0.75, 0.56, 0.984, 0.56,
0.83, 0.83, 0.947, 0.984, 1.08, 0.85, 1.54, 0.68, 0.94, 0.64,
0.91, 0.98, 0.22, 0.62, 0.596, 0.7), LowerCI = c(0.34, 0.48,
0.26, 0.135, 0.32, 0.439, 0.75, 0.48, 0.54, 0.93, 0.35, 0.1,
0.49, 0.82, 0.24, 0.65, 0.03, 0.51, 0.3, 0.18, 0.55, 0.44, 0.5,
0.34, 0.914, 0.07, 0.49, 0.42, 0.089, 0.498, 0.63, 0.47, 0.73,
0.48, 0.57, 0.48, 0.76, 0.51, 0.11, 0.44, 0.331, 0.67), UpperCI = c(0.86,
1.06, 0.945, 0.694, 0.79, 0.917, 2, 2.24, 0.87, 1.51, 0.99, 1.77,
0.99, 1.68, 1.27, 1.42, 0.62, 1.1, 0.83, 1.09, 1.36, 0.87, 1.12,
0.91, 1.061, 4.26, 1.42, 1.64, 10.047, 1.941, 1.82, 1.56, 3.28,
0.95, 1.56, 0.87, 1.09, 1.86, 0.45, 0.86, 1.074, 0.73)), row.names = c(NA,
-42L), class = "data.frame")


reml_meta <- function (yi, sei)
{
    keep <- is.finite(yi) & is.finite(sei) & sei > 0
    yi <- yi[keep]
    sei <- sei[keep]
    vi <- sei^2
    k <- length(yi)
    if (!k)
        stop("No valid estimates.")
    if (k == 1L) {
        z <- qnorm(0.975)
        return(list(k = 1L, mu = yi, se_mu = sei, ci_low = yi - z * sei, ci_high = yi + z * sei, p = 2 * pnorm(abs(yi/sei), lower.tail = FALSE), tau2 = NA_real_, Q = NA_real_, df_Q = 0L, Q_p = NA_real_, I2 = NA_real_, weights = 100))
    }
    wf <- 1/vi
    muf <- sum(wf * yi)/sum(wf)
    Q <- sum(wf * (yi - muf)^2)
    dfQ <- k - 1
    I2 <- if (Q > 0)
        max(0, (Q - dfQ)/Q) * 100
    else 0
    obj <- function(tau2) {
        w <- 1/(vi + tau2)
        mu <- sum(w * yi)/sum(w)
        0.5 * (sum(log(vi + tau2)) + log(sum(w)) + sum(w * (yi - mu)^2))
    }
    upper <- max(1, var(yi) * 10, max(vi) * 10)
    opt <- optimize(obj, c(0, upper), tol = 1e-12)
    tau2 <- if (opt$minimum < 1e-10)
        0
    else opt$minimum
    w <- 1/(vi + tau2)
    mu <- sum(w * yi)/sum(w)
    se <- sqrt(1/sum(w))
    ci <- mu + c(-1, 1) * qnorm(0.975) * se
    list(k = k, mu = mu, se_mu = se, ci_low = ci[1], ci_high = ci[2], p = 2 * pnorm(abs(mu/se), lower.tail = FALSE), tau2 = tau2, Q = Q, df_Q = dfQ, Q_p = pchisq(Q, dfQ, lower.tail = FALSE), I2 = I2, weights = w/sum(w) * 100)
}
prepare_effects <- function (d, effect_col = "Effect")
{
    d[[effect_col]] <- as.numeric(d[[effect_col]])
    d$LowerCI <- as.numeric(d$LowerCI)
    d$UpperCI <- as.numeric(d$UpperCI)
    ok <- is.finite(d[[effect_col]]) & is.finite(d$LowerCI) & is.finite(d$UpperCI) & d[[effect_col]] > 0 & d$LowerCI > 0 & d$UpperCI > d$LowerCI
    d <- d[ok, , drop = FALSE]
    d$Effect <- d[[effect_col]]
    d$yi <- log(d$Effect)
    d$sei <- (log(d$UpperCI) - log(d$LowerCI))/(2 * qnorm(0.975))
    d
}
fmt_p <- function (p)
if (!is.finite(p)) "NR" else if (p < 1e-04) "< 0.0001" else paste0("= ", formatC(p, format = "f", digits = 4))
subgroup_forest <- function (d, var, label, endpoint, measure, file)
{
    preferred <- if (var == "CancerType")
        c("Melanoma", "NSCLC", "RCC", "HCC", "HNSCC", "Multiple/Other", "Multiple/other")
    else if (var == "StudyRegion")
        c("America", "United States", "Europe", "Asia", "Others", "Multiple regions")
    else c("Anti-PD-1/PD-L1", "Anti-CTLA-4", "Anti-PD-1/PD-L1 + Anti-CTLA-4")
    levels <- c(preferred[preferred %in% unique(d[[var]])], setdiff(unique(d[[var]]), preferred))
    rows <- list()
    models <- list()
    for (g in levels) {
        z <- d[d[[var]] == g, , drop = FALSE]
        m <- reml_meta(z$yi, z$sei)
        models[[g]] <- m
        rows[[length(rows) + 1]] <- data.frame(type = "title", g = g, i = NA)
        for (i in which(d[[var]] == g)) rows[[length(rows) + 1]] <- data.frame(type = "study", g = g, i = i)
        rows[[length(rows) + 1]] <- data.frame(type = "sum", g = g, i = NA)
        rows[[length(rows) + 1]] <- data.frame(type = "het", g = g, i = NA)
        rows[[length(rows) + 1]] <- data.frame(type = "space", g = g, i = NA)
    }
    rp <- do.call(rbind, rows)
    rp$y <- rev(seq_len(nrow(rp))) + 5
    overall <- reml_meta(d$yi, d$sei)
    h <- max(8, 3.7 + 0.255 * nrow(rp))
    jpeg(file, width = 11, height = h, units = "in", res = 300, quality = 100)
    on.exit(dev.off(), add = TRUE)
    par(mar = c(4.7, 0.65, 1.8, 0.65), family = "sans", xpd = NA)
    plot.new()
    plot.window(xlim = c(0, 1), ylim = c(-2.1, max(rp$y) + 3))
    xs <- 0.02
    xln <- 0.31
    xse <- 0.41
    pl <- 0.515
    pr <- 0.7
    xe <- 0.775
    xci <- 0.89
    xw <- 0.98
    amin <- 0.1
    amax <- 10
    map <- function(v) pl + (log(v) - log(amin))/(log(amax) - log(amin)) * (pr - pl)
    clamp <- function(v) pmin(pr, pmax(pl, map(v)))
    hy <- max(rp$y) + 2
    text(xs, hy, "Subgroup / Study", adj = 0, font = 2, cex = 0.84)
    text(xln, hy, paste0("log", measure), adj = 1, font = 2, cex = 0.84)
    text(xse, hy, paste0("SE(log", measure, ")"), adj = 1, font = 2, cex = 0.84)
    text((pl + pr)/2, hy, if (measure == "HR")
        "Hazard Ratio"
    else "Odds Ratio", font = 2, cex = 0.84)
    text(xe, hy, measure, adj = 1, font = 2, cex = 0.84)
    text(xci, hy, "95% CI", font = 2, cex = 0.84)
    text(xw, hy, "Weight", adj = 1, font = 2, cex = 0.84)
    segments(map(1), -0.4, map(1), max(rp$y) + 1.2)
    segments(clamp(exp(overall$mu)), -0.4, clamp(exp(overall$mu)), max(rp$y) + 1.2, lty = 3)
    for (r in seq_len(nrow(rp))) {
        z <- rp[r, ]
        y <- z$y
        if (z$type == "title")
            text(xs, y, paste0(z$g, " (k = ", sum(d[[var]] == z$g), ")"), adj = 0, font = 2, col = "#16836F", cex = 0.8)
        if (z$type == "study") {
            i <- z$i
            segments(clamp(d$LowerCI[i]), y, clamp(d$UpperCI[i]), y)
            if (d$LowerCI[i] < amin)
                arrows(pl + 0.012, y, pl, y, length = 0.04)
            if (d$UpperCI[i] > amax)
                arrows(pr - 0.012, y, pr, y, length = 0.04)
            points(clamp(d$Effect[i]), y, pch = 15, col = "#0A4F78", cex = 0.55 + 1.1 * sqrt(overall$weights[i]/max(overall$weights)))
            text(xs, y, d$Study[i], adj = 0, cex = 0.68)
            text(xln, y, formatC(d$yi[i], format = "f", digits = 4), adj = 1, cex = 0.65)
            text(xse, y, formatC(d$sei[i], format = "f", digits = 4), adj = 1, cex = 0.65)
            text(xe, y, sprintf("%.2f", d$Effect[i]), adj = 1, cex = 0.68)
            text(xci, y, sprintf("[%.2f; %.2f]", d$LowerCI[i], d$UpperCI[i]), cex = 0.68)
            text(xw, y, sprintf("%.1f%%", overall$weights[i]), adj = 1, cex = 0.68)
        }
        if (z$type == "sum") {
            m <- models[[z$g]]
            polygon(c(clamp(exp(m$ci_low)), clamp(exp(m$mu)), clamp(exp(m$ci_high)), clamp(exp(m$mu))), c(y, y + 0.24, y, y - 0.24), col = "red", border = "black")
            text(xs, y, "Random effects model", adj = 0, font = 2, col = "#16836F", cex = 0.7)
            text(xe, y, sprintf("%.2f", exp(m$mu)), adj = 1, font = 2, col = "#16836F", cex = 0.7)
            text(xci, y, sprintf("[%.2f; %.2f]", exp(m$ci_low), exp(m$ci_high)), font = 2, col = "#16836F", cex = 0.7)
        }
        if (z$type == "het") {
            m <- models[[z$g]]
            text(xs, y, sprintf("Heterogeneity: I² = %.1f%%, τ² = %.4f, p %s", m$I2, m$tau2, fmt_p(m$Q_p)), adj = 0, col = "#16836F", cex = 0.65)
        }
    }
    py <- 0.15
    polygon(c(clamp(exp(overall$ci_low)), clamp(exp(overall$mu)), clamp(exp(overall$ci_high)), clamp(exp(overall$mu))), c(py, py + 0.22, py, py - 0.22), col = "red", border = "black")
    text(xs, py, "Overall random effects model", adj = 0, font = 2, cex = 0.74)
    text(xe, py, sprintf("%.2f", exp(overall$mu)), adj = 1, font = 2, cex = 0.74)
    text(xci, py, sprintf("[%.2f; %.2f]", exp(overall$ci_low), exp(overall$ci_high)), font = 2, cex = 0.74)
    text(xw, py, "100.0%", adj = 1, font = 2, cex = 0.74)
    text(xs, py - 0.65, sprintf("Overall heterogeneity: I² = %.1f%%, τ² = %.4f, p %s", overall$I2, overall$tau2, fmt_p(overall$Q_p)), adj = 0, cex = 0.64)
    vi <- d$sei^2
    tau2 <- overall$tau2
    w <- 1/(vi + tau2)
    g <- factor(d[[var]], levels = levels)
    X <- model.matrix(~g)
    A <- crossprod(X, w * X)
    bb <- solve(A, crossprod(X, w * d$yi))
    VV <- solve(A)
    Qm <- if (ncol(X) > 1)
        as.numeric(crossprod(bb[-1], solve(VV[-1, -1, drop = FALSE], bb[-1])))
    else NA
    df <- ncol(X) - 1
    pm <- if (df > 0)
        pchisq(Qm, df, lower.tail = FALSE)
    else NA
    text(xs, -1.55, sprintf("Test for subgroup differences: Qm = %.2f, df = %d (p = %.4f)", Qm, df, pm), adj = 0, cex = 0.62)
    text(xs, -1.9, "Random-effects model (REML; normal 95% CI). Subgroups with k < 3 are exploratory.", adj = 0, cex = 0.58, col = "#555555")
    ay <- -0.75
    segments(pl, ay, pr, ay)
    for (t in c(0.1, 0.5, 1, 2, 10)) {
        segments(map(t), ay, map(t), ay - 0.15)
        text(map(t), ay - 0.35, t, cex = 0.66)
    }
    text((pl + pr)/2, ay - 0.75, if (measure == "HR")
        endpoint
    else "Odds Ratio", cex = 0.72)
}
d<-prepare_effects(as.data.frame(embedded_input));subgroup_forest(d,'CancerType','Cancer type',"OS","HR",'subgroup_CancerType.jpg');subgroup_forest(d,'StudyRegion','Study region',"OS","HR",'subgroup_StudyRegion.jpg');subgroup_forest(d,'ICIClass','ICI class',"OS","HR",'subgroup_ICIClass.jpg')
```

**Supplementary Code S20.**Subgroup analysis — adjusted PFS

```
# Analysis: 20_subgroup_PFS_adjusted
# All analysis data are embedded. Running this file creates figures only.
rm(list = ls())
options(stringsAsFactors = FALSE, scipen = 999)
analysis_id <- "20_subgroup_PFS_adjusted"
script_arg <- grep('^--file=', commandArgs(trailingOnly = FALSE), value = TRUE)
script_file <- if (length(script_arg)) sub('^--file=', '', script_arg[1]) else NA_character_
if ((is.na(script_file) || !nzchar(script_file)) && interactive() && requireNamespace('rstudioapi', quietly = TRUE)) {
  p <- tryCatch(rstudioapi::getSourceEditorContext()$path, error = function(e) '')
  if (nzchar(p)) script_file <- p
}
script_dir <- if (!is.na(script_file) && nzchar(script_file)) dirname(normalizePath(script_file, winslash='/', mustWork=TRUE)) else normalizePath(getwd(), winslash='/', mustWork=TRUE)
output_dir <- file.path(script_dir, paste0(analysis_id, '_output'))
dir.create(output_dir, recursive=TRUE, showWarnings=FALSE)
setwd(output_dir)
.libPaths(unique(c(file.path(Sys.getenv('USERPROFILE'), 'R-library'), .libPaths())))
embedded_input <- structure(list(Study = c("McQuade (1) 2018", "McQuade (2) 2018",
"Martini 2019", "Cortellini 2019", "Donnelly (1) 2019", "Donnelly (2) 2019",
"Kichenadasse 2019", "Wang 2019", "Naik 2019", "Cortellini 2020",
"Rutkowski 2020", "Rogado 2020", "Minami 2020", "Ahmed 2021",
"Bartl 2021", "Akce 2021", "Esposito 2021", "Zhang 2021", "Chen 2022",
"Imai 2022", "Wang 2022", "Tateishi 2022", "Incorvaia 2023",
"Jain 2023", "Vithayathil 2023", "Zhang 2023", "McManus 2023",
"Miyamoto 2023", "Alam 2024", "Gómez-Banoy 2024", "Nie (1) 2024",
"Nie (2) 2024", "Tan 2024", "Wang 2024"), CancerType = c("Melanoma",
"Melanoma", "NSCLC", "Multiple/Other", "Melanoma", "Melanoma",
"NSCLC", "Multiple/Other", "Melanoma", "NSCLC", "Melanoma", "Multiple/Other",
"NSCLC", "Multiple/Other", "Multiple/Other", "HCC", "Multiple/Other",
"Multiple/Other", "HCC", "NSCLC", "RCC", "NSCLC", "Multiple/Other",
"NSCLC", "HCC", "HNSCC", "RCC", "HNSCC", "Multiple/Other", "Multiple/Other",
"NSCLC", "NSCLC", "Multiple/Other", "HCC"), StudyRegion = c("Others",
"Others", "USA", "Europe", "USA", "USA", "Others", "USA", "USA",
"Others", "Others", "Europe", "Asia", "USA", "Europe", "USA",
"Europe", "USA", "Asia", "Asia", "Asia", "Asia", "Europe", "USA",
"Others", "Asia", "USA", "Asia", "USA", "USA", "Others", "Others",
"Asia", "Asia"), ICIClass = c("Anti-CTLA-4", "Anti-PD-1/PD-L1",
"Anti-PD-1/PD-L1", "Anti-PD-1/PD-L1", "Anti-CTLA-4", "Anti-PD-1/PD-L1",
"Anti-PD-1/PD-L1", "Anti-PD-1/PD-L1", "Anti-PD-1/PD-L1 + Anti-CTLA-4",
"Anti-PD-1/PD-L1", "Anti-PD-1/PD-L1 + Anti-CTLA-4", "Anti-PD-1/PD-L1",
"Anti-PD-1/PD-L1", "Anti-PD-1/PD-L1 + Anti-CTLA-4", "Anti-PD-1/PD-L1",
"Anti-PD-1/PD-L1", "Anti-PD-1/PD-L1", "Anti-PD-1/PD-L1", "Anti-PD-1/PD-L1",
"Anti-PD-1/PD-L1", "Anti-PD-1/PD-L1", "Anti-PD-1/PD-L1", "Anti-PD-1/PD-L1",
"Anti-PD-1/PD-L1", "Anti-PD-1/PD-L1", "Anti-PD-1/PD-L1", "Anti-PD-1/PD-L1 + Anti-CTLA-4",
"Anti-PD-1/PD-L1", "Anti-PD-1/PD-L1 + Anti-CTLA-4", "Anti-PD-1/PD-L1",
"Anti-PD-1/PD-L1", "Anti-PD-1/PD-L1", "Anti-PD-1/PD-L1", "Anti-PD-1/PD-L1"
), Effect = c(0.63, 0.85, 0.543, 0.72, 1.16, 2.46, 0.87, 0.61,
1, 0.61, 1.01, 0.58, 0.9, 0.87, 0.27, 1.04, 0.8, 0.76, 0.47,
0.78, 0.44, 1, 0.18, 0.7, 0.8, 0.863, 1.859, 1.18, 0.94, 0.54,
1.01, 1.14, 0.022, 0.32), LowerCI = c(0.41, 0.61, 0.278, 0.49,
0.7, 1.03, 0.72, 0.42, 0.34, 0.45, 0.74, 0.38, 0.45, 0.59, 0.12,
0.56, 0.54, 0.55, 0.22, 0.51, 0.17, 0.4, 0.03, 0.42, 0.41, 0.091,
1.156, 0.76, 0.62, 0.42, 0.84, 0.68, 0.001, 0.17), UpperCI = c(0.95,
1.19, 1.064, 1.06, 1.94, 5.89, 1.04, 0.89, 2.94, 0.82, 1.39,
0.89, 1.82, 1.28, 0.61, 1.92, 1.19, 1.04, 0.98, 1.19, 1.13, 2.45,
0.93, 1.14, 1.56, 8.156, 2.989, 1.82, 1.44, 0.71, 1.21, 1.92,
0.326, 0.59)), row.names = c(NA, -34L), class = "data.frame")


reml_meta <- function (yi, sei)
{
    keep <- is.finite(yi) & is.finite(sei) & sei > 0
    yi <- yi[keep]
    sei <- sei[keep]
    vi <- sei^2
    k <- length(yi)
    if (!k)
        stop("No valid estimates.")
    if (k == 1L) {
        z <- qnorm(0.975)
        return(list(k = 1L, mu = yi, se_mu = sei, ci_low = yi - z * sei, ci_high = yi + z * sei, p = 2 * pnorm(abs(yi/sei), lower.tail = FALSE), tau2 = NA_real_, Q = NA_real_, df_Q = 0L, Q_p = NA_real_, I2 = NA_real_, weights = 100))
    }
    wf <- 1/vi
    muf <- sum(wf * yi)/sum(wf)
    Q <- sum(wf * (yi - muf)^2)
    dfQ <- k - 1
    I2 <- if (Q > 0)
        max(0, (Q - dfQ)/Q) * 100
    else 0
    obj <- function(tau2) {
        w <- 1/(vi + tau2)
        mu <- sum(w * yi)/sum(w)
        0.5 * (sum(log(vi + tau2)) + log(sum(w)) + sum(w * (yi - mu)^2))
    }
    upper <- max(1, var(yi) * 10, max(vi) * 10)
    opt <- optimize(obj, c(0, upper), tol = 1e-12)
    tau2 <- if (opt$minimum < 1e-10)
        0
    else opt$minimum
    w <- 1/(vi + tau2)
    mu <- sum(w * yi)/sum(w)
    se <- sqrt(1/sum(w))
    ci <- mu + c(-1, 1) * qnorm(0.975) * se
    list(k = k, mu = mu, se_mu = se, ci_low = ci[1], ci_high = ci[2], p = 2 * pnorm(abs(mu/se), lower.tail = FALSE), tau2 = tau2, Q = Q, df_Q = dfQ, Q_p = pchisq(Q, dfQ, lower.tail = FALSE), I2 = I2, weights = w/sum(w) * 100)
}
prepare_effects <- function (d, effect_col = "Effect")
{
    d[[effect_col]] <- as.numeric(d[[effect_col]])
    d$LowerCI <- as.numeric(d$LowerCI)
    d$UpperCI <- as.numeric(d$UpperCI)
    ok <- is.finite(d[[effect_col]]) & is.finite(d$LowerCI) & is.finite(d$UpperCI) & d[[effect_col]] > 0 & d$LowerCI > 0 & d$UpperCI > d$LowerCI
    d <- d[ok, , drop = FALSE]
    d$Effect <- d[[effect_col]]
    d$yi <- log(d$Effect)
    d$sei <- (log(d$UpperCI) - log(d$LowerCI))/(2 * qnorm(0.975))
    d
}
fmt_p <- function (p)
if (!is.finite(p)) "NR" else if (p < 1e-04) "< 0.0001" else paste0("= ", formatC(p, format = "f", digits = 4))
subgroup_forest <- function (d, var, label, endpoint, measure, file)
{
    preferred <- if (var == "CancerType")
        c("Melanoma", "NSCLC", "RCC", "HCC", "HNSCC", "Multiple/Other", "Multiple/other")
    else if (var == "StudyRegion")
        c("America", "United States", "Europe", "Asia", "Others", "Multiple regions")
    else c("Anti-PD-1/PD-L1", "Anti-CTLA-4", "Anti-PD-1/PD-L1 + Anti-CTLA-4")
    levels <- c(preferred[preferred %in% unique(d[[var]])], setdiff(unique(d[[var]]), preferred))
    rows <- list()
    models <- list()
    for (g in levels) {
        z <- d[d[[var]] == g, , drop = FALSE]
        m <- reml_meta(z$yi, z$sei)
        models[[g]] <- m
        rows[[length(rows) + 1]] <- data.frame(type = "title", g = g, i = NA)
        for (i in which(d[[var]] == g)) rows[[length(rows) + 1]] <- data.frame(type = "study", g = g, i = i)
        rows[[length(rows) + 1]] <- data.frame(type = "sum", g = g, i = NA)
        rows[[length(rows) + 1]] <- data.frame(type = "het", g = g, i = NA)
        rows[[length(rows) + 1]] <- data.frame(type = "space", g = g, i = NA)
    }
    rp <- do.call(rbind, rows)
    rp$y <- rev(seq_len(nrow(rp))) + 5
    overall <- reml_meta(d$yi, d$sei)
    h <- max(8, 3.7 + 0.255 * nrow(rp))
    jpeg(file, width = 11, height = h, units = "in", res = 300, quality = 100)
    on.exit(dev.off(), add = TRUE)
    par(mar = c(4.7, 0.65, 1.8, 0.65), family = "sans", xpd = NA)
    plot.new()
    plot.window(xlim = c(0, 1), ylim = c(-2.1, max(rp$y) + 3))
    xs <- 0.02
    xln <- 0.31
    xse <- 0.41
    pl <- 0.515
    pr <- 0.7
    xe <- 0.775
    xci <- 0.89
    xw <- 0.98
    amin <- 0.1
    amax <- 10
    map <- function(v) pl + (log(v) - log(amin))/(log(amax) - log(amin)) * (pr - pl)
    clamp <- function(v) pmin(pr, pmax(pl, map(v)))
    hy <- max(rp$y) + 2
    text(xs, hy, "Subgroup / Study", adj = 0, font = 2, cex = 0.84)
    text(xln, hy, paste0("log", measure), adj = 1, font = 2, cex = 0.84)
    text(xse, hy, paste0("SE(log", measure, ")"), adj = 1, font = 2, cex = 0.84)
    text((pl + pr)/2, hy, if (measure == "HR")
        "Hazard Ratio"
    else "Odds Ratio", font = 2, cex = 0.84)
    text(xe, hy, measure, adj = 1, font = 2, cex = 0.84)
    text(xci, hy, "95% CI", font = 2, cex = 0.84)
    text(xw, hy, "Weight", adj = 1, font = 2, cex = 0.84)
    segments(map(1), -0.4, map(1), max(rp$y) + 1.2)
    segments(clamp(exp(overall$mu)), -0.4, clamp(exp(overall$mu)), max(rp$y) + 1.2, lty = 3)
    for (r in seq_len(nrow(rp))) {
        z <- rp[r, ]
        y <- z$y
        if (z$type == "title")
            text(xs, y, paste0(z$g, " (k = ", sum(d[[var]] == z$g), ")"), adj = 0, font = 2, col = "#16836F", cex = 0.8)
        if (z$type == "study") {
            i <- z$i
            segments(clamp(d$LowerCI[i]), y, clamp(d$UpperCI[i]), y)
            if (d$LowerCI[i] < amin)
                arrows(pl + 0.012, y, pl, y, length = 0.04)
            if (d$UpperCI[i] > amax)
                arrows(pr - 0.012, y, pr, y, length = 0.04)
            points(clamp(d$Effect[i]), y, pch = 15, col = "#0A4F78", cex = 0.55 + 1.1 * sqrt(overall$weights[i]/max(overall$weights)))
            text(xs, y, d$Study[i], adj = 0, cex = 0.68)
            text(xln, y, formatC(d$yi[i], format = "f", digits = 4), adj = 1, cex = 0.65)
            text(xse, y, formatC(d$sei[i], format = "f", digits = 4), adj = 1, cex = 0.65)
            text(xe, y, sprintf("%.2f", d$Effect[i]), adj = 1, cex = 0.68)
            text(xci, y, sprintf("[%.2f; %.2f]", d$LowerCI[i], d$UpperCI[i]), cex = 0.68)
            text(xw, y, sprintf("%.1f%%", overall$weights[i]), adj = 1, cex = 0.68)
        }
        if (z$type == "sum") {
            m <- models[[z$g]]
            polygon(c(clamp(exp(m$ci_low)), clamp(exp(m$mu)), clamp(exp(m$ci_high)), clamp(exp(m$mu))), c(y, y + 0.24, y, y - 0.24), col = "red", border = "black")
            text(xs, y, "Random effects model", adj = 0, font = 2, col = "#16836F", cex = 0.7)
            text(xe, y, sprintf("%.2f", exp(m$mu)), adj = 1, font = 2, col = "#16836F", cex = 0.7)
            text(xci, y, sprintf("[%.2f; %.2f]", exp(m$ci_low), exp(m$ci_high)), font = 2, col = "#16836F", cex = 0.7)
        }
        if (z$type == "het") {
            m <- models[[z$g]]
            text(xs, y, sprintf("Heterogeneity: I² = %.1f%%, τ² = %.4f, p %s", m$I2, m$tau2, fmt_p(m$Q_p)), adj = 0, col = "#16836F", cex = 0.65)
        }
    }
    py <- 0.15
    polygon(c(clamp(exp(overall$ci_low)), clamp(exp(overall$mu)), clamp(exp(overall$ci_high)), clamp(exp(overall$mu))), c(py, py + 0.22, py, py - 0.22), col = "red", border = "black")
    text(xs, py, "Overall random effects model", adj = 0, font = 2, cex = 0.74)
    text(xe, py, sprintf("%.2f", exp(overall$mu)), adj = 1, font = 2, cex = 0.74)
    text(xci, py, sprintf("[%.2f; %.2f]", exp(overall$ci_low), exp(overall$ci_high)), font = 2, cex = 0.74)
    text(xw, py, "100.0%", adj = 1, font = 2, cex = 0.74)
    text(xs, py - 0.65, sprintf("Overall heterogeneity: I² = %.1f%%, τ² = %.4f, p %s", overall$I2, overall$tau2, fmt_p(overall$Q_p)), adj = 0, cex = 0.64)
    vi <- d$sei^2
    tau2 <- overall$tau2
    w <- 1/(vi + tau2)
    g <- factor(d[[var]], levels = levels)
    X <- model.matrix(~g)
    A <- crossprod(X, w * X)
    bb <- solve(A, crossprod(X, w * d$yi))
    VV <- solve(A)
    Qm <- if (ncol(X) > 1)
        as.numeric(crossprod(bb[-1], solve(VV[-1, -1, drop = FALSE], bb[-1])))
    else NA
    df <- ncol(X) - 1
    pm <- if (df > 0)
        pchisq(Qm, df, lower.tail = FALSE)
    else NA
    text(xs, -1.55, sprintf("Test for subgroup differences: Qm = %.2f, df = %d (p = %.4f)", Qm, df, pm), adj = 0, cex = 0.62)
    text(xs, -1.9, "Random-effects model (REML; normal 95% CI). Subgroups with k < 3 are exploratory.", adj = 0, cex = 0.58, col = "#555555")
    ay <- -0.75
    segments(pl, ay, pr, ay)
    for (t in c(0.1, 0.5, 1, 2, 10)) {
        segments(map(t), ay, map(t), ay - 0.15)
        text(map(t), ay - 0.35, t, cex = 0.66)
    }
    text((pl + pr)/2, ay - 0.75, if (measure == "HR")
        endpoint
    else "Odds Ratio", cex = 0.72)
}
d<-prepare_effects(as.data.frame(embedded_input));subgroup_forest(d,'CancerType','Cancer type',"PFS","HR",'subgroup_CancerType.jpg');subgroup_forest(d,'StudyRegion','Study region',"PFS","HR",'subgroup_StudyRegion.jpg');subgroup_forest(d,'ICIClass','ICI class',"PFS","HR",'subgroup_ICIClass.jpg')
```

**Supplementary Code S21.**Subgroup analysis — adjusted ORR

```
# Analysis: 21_subgroup_ORR_adjusted
# All analysis data are embedded. Running this file creates figures only.
rm(list = ls())
options(stringsAsFactors = FALSE, scipen = 999)
analysis_id <- "21_subgroup_ORR_adjusted"
script_arg <- grep('^--file=', commandArgs(trailingOnly = FALSE), value = TRUE)
script_file <- if (length(script_arg)) sub('^--file=', '', script_arg[1]) else NA_character_
if ((is.na(script_file) || !nzchar(script_file)) && interactive() && requireNamespace('rstudioapi', quietly = TRUE)) {
  p <- tryCatch(rstudioapi::getSourceEditorContext()$path, error = function(e) '')
  if (nzchar(p)) script_file <- p
}
script_dir <- if (!is.na(script_file) && nzchar(script_file)) dirname(normalizePath(script_file, winslash='/', mustWork=TRUE)) else normalizePath(getwd(), winslash='/', mustWork=TRUE)
output_dir <- file.path(script_dir, paste0(analysis_id, '_output'))
dir.create(output_dir, recursive=TRUE, showWarnings=FALSE)
setwd(output_dir)
.libPaths(unique(c(file.path(Sys.getenv('USERPROFILE'), 'R-library'), .libPaths())))
embedded_input <- structure(list(Study = c("Cortellini 2019", "Cortellini 2020",
"Esposito 2021", "Jain 2023", "McManus 2023", "Alam 2024", "Bernard 2024",
"Wang 2024"), CancerType = c("Multiple/Other", "NSCLC", "Multiple/Other",
"NSCLC", "RCC", "Multiple/Other", "Multiple/Other", "HCC"), StudyRegion = c("Europe",
"Others", "Europe", "USA", "USA", "USA", "USA", "Asia"), ICIClass = c("Anti-PD-1/PD-L1",
"Anti-PD-1/PD-L1", "Anti-PD-1/PD-L1", "Anti-PD-1/PD-L1", "Anti-PD-1/PD-L1 + Anti-CTLA-4",
"Anti-PD-1/PD-L1 + Anti-CTLA-4", "Anti-PD-1/PD-L1", "Anti-PD-1/PD-L1"
), Effect = c(1.56, 1.61, 0.96, 2.67, 0.268, 1.18, 2.62, 6.22
), LowerCI = c(1.061, 1.04, 0.3, 1.11, 0.078, 0.81, 0.66, 1.75
), UpperCI = c(2.295, 2.5, 3.1, 6.4, 0.773, 1.73, 10.78, 22.17
)), row.names = c(NA, -8L), class = "data.frame")


reml_meta <- function (yi, sei)
{
    keep <- is.finite(yi) & is.finite(sei) & sei > 0
    yi <- yi[keep]
    sei <- sei[keep]
    vi <- sei^2
    k <- length(yi)
    if (!k)
        stop("No valid estimates.")
    if (k == 1L) {
        z <- qnorm(0.975)
        return(list(k = 1L, mu = yi, se_mu = sei, ci_low = yi - z * sei, ci_high = yi + z * sei, p = 2 * pnorm(abs(yi/sei), lower.tail = FALSE), tau2 = NA_real_, Q = NA_real_, df_Q = 0L, Q_p = NA_real_, I2 = NA_real_, weights = 100))
    }
    wf <- 1/vi
    muf <- sum(wf * yi)/sum(wf)
    Q <- sum(wf * (yi - muf)^2)
    dfQ <- k - 1
    I2 <- if (Q > 0)
        max(0, (Q - dfQ)/Q) * 100
    else 0
    obj <- function(tau2) {
        w <- 1/(vi + tau2)
        mu <- sum(w * yi)/sum(w)
        0.5 * (sum(log(vi + tau2)) + log(sum(w)) + sum(w * (yi - mu)^2))
    }
    upper <- max(1, var(yi) * 10, max(vi) * 10)
    opt <- optimize(obj, c(0, upper), tol = 1e-12)
    tau2 <- if (opt$minimum < 1e-10)
        0
    else opt$minimum
    w <- 1/(vi + tau2)
    mu <- sum(w * yi)/sum(w)
    se <- sqrt(1/sum(w))
    ci <- mu + c(-1, 1) * qnorm(0.975) * se
    list(k = k, mu = mu, se_mu = se, ci_low = ci[1], ci_high = ci[2], p = 2 * pnorm(abs(mu/se), lower.tail = FALSE), tau2 = tau2, Q = Q, df_Q = dfQ, Q_p = pchisq(Q, dfQ, lower.tail = FALSE), I2 = I2, weights = w/sum(w) * 100)
}
prepare_effects <- function (d, effect_col = "Effect")
{
    d[[effect_col]] <- as.numeric(d[[effect_col]])
    d$LowerCI <- as.numeric(d$LowerCI)
    d$UpperCI <- as.numeric(d$UpperCI)
    ok <- is.finite(d[[effect_col]]) & is.finite(d$LowerCI) & is.finite(d$UpperCI) & d[[effect_col]] > 0 & d$LowerCI > 0 & d$UpperCI > d$LowerCI
    d <- d[ok, , drop = FALSE]
    d$Effect <- d[[effect_col]]
    d$yi <- log(d$Effect)
    d$sei <- (log(d$UpperCI) - log(d$LowerCI))/(2 * qnorm(0.975))
    d
}
fmt_p <- function (p)
if (!is.finite(p)) "NR" else if (p < 1e-04) "< 0.0001" else paste0("= ", formatC(p, format = "f", digits = 4))
subgroup_forest <- function (d, var, label, endpoint, measure, file)
{
    preferred <- if (var == "CancerType")
        c("Melanoma", "NSCLC", "RCC", "HCC", "HNSCC", "Multiple/Other", "Multiple/other")
    else if (var == "StudyRegion")
        c("America", "United States", "Europe", "Asia", "Others", "Multiple regions")
    else c("Anti-PD-1/PD-L1", "Anti-CTLA-4", "Anti-PD-1/PD-L1 + Anti-CTLA-4")
    levels <- c(preferred[preferred %in% unique(d[[var]])], setdiff(unique(d[[var]]), preferred))
    rows <- list()
    models <- list()
    for (g in levels) {
        z <- d[d[[var]] == g, , drop = FALSE]
        m <- reml_meta(z$yi, z$sei)
        models[[g]] <- m
        rows[[length(rows) + 1]] <- data.frame(type = "title", g = g, i = NA)
        for (i in which(d[[var]] == g)) rows[[length(rows) + 1]] <- data.frame(type = "study", g = g, i = i)
        rows[[length(rows) + 1]] <- data.frame(type = "sum", g = g, i = NA)
        rows[[length(rows) + 1]] <- data.frame(type = "het", g = g, i = NA)
        rows[[length(rows) + 1]] <- data.frame(type = "space", g = g, i = NA)
    }
    rp <- do.call(rbind, rows)
    rp$y <- rev(seq_len(nrow(rp))) + 5
    overall <- reml_meta(d$yi, d$sei)
    h <- max(8, 3.7 + 0.255 * nrow(rp))
    jpeg(file, width = 11, height = h, units = "in", res = 300, quality = 100)
    on.exit(dev.off(), add = TRUE)
    par(mar = c(4.7, 0.65, 1.8, 0.65), family = "sans", xpd = NA)
    plot.new()
    plot.window(xlim = c(0, 1), ylim = c(-2.1, max(rp$y) + 3))
    xs <- 0.02
    xln <- 0.31
    xse <- 0.41
    pl <- 0.515
    pr <- 0.7
    xe <- 0.775
    xci <- 0.89
    xw <- 0.98
    amin <- 0.1
    amax <- 10
    map <- function(v) pl + (log(v) - log(amin))/(log(amax) - log(amin)) * (pr - pl)
    clamp <- function(v) pmin(pr, pmax(pl, map(v)))
    hy <- max(rp$y) + 2
    text(xs, hy, "Subgroup / Study", adj = 0, font = 2, cex = 0.84)
    text(xln, hy, paste0("log", measure), adj = 1, font = 2, cex = 0.84)
    text(xse, hy, paste0("SE(log", measure, ")"), adj = 1, font = 2, cex = 0.84)
    text((pl + pr)/2, hy, if (measure == "HR")
        "Hazard Ratio"
    else "Odds Ratio", font = 2, cex = 0.84)
    text(xe, hy, measure, adj = 1, font = 2, cex = 0.84)
    text(xci, hy, "95% CI", font = 2, cex = 0.84)
    text(xw, hy, "Weight", adj = 1, font = 2, cex = 0.84)
    segments(map(1), -0.4, map(1), max(rp$y) + 1.2)
    segments(clamp(exp(overall$mu)), -0.4, clamp(exp(overall$mu)), max(rp$y) + 1.2, lty = 3)
    for (r in seq_len(nrow(rp))) {
        z <- rp[r, ]
        y <- z$y
        if (z$type == "title")
            text(xs, y, paste0(z$g, " (k = ", sum(d[[var]] == z$g), ")"), adj = 0, font = 2, col = "#16836F", cex = 0.8)
        if (z$type == "study") {
            i <- z$i
            segments(clamp(d$LowerCI[i]), y, clamp(d$UpperCI[i]), y)
            if (d$LowerCI[i] < amin)
                arrows(pl + 0.012, y, pl, y, length = 0.04)
            if (d$UpperCI[i] > amax)
                arrows(pr - 0.012, y, pr, y, length = 0.04)
            points(clamp(d$Effect[i]), y, pch = 15, col = "#0A4F78", cex = 0.55 + 1.1 * sqrt(overall$weights[i]/max(overall$weights)))
            text(xs, y, d$Study[i], adj = 0, cex = 0.68)
            text(xln, y, formatC(d$yi[i], format = "f", digits = 4), adj = 1, cex = 0.65)
            text(xse, y, formatC(d$sei[i], format = "f", digits = 4), adj = 1, cex = 0.65)
            text(xe, y, sprintf("%.2f", d$Effect[i]), adj = 1, cex = 0.68)
            text(xci, y, sprintf("[%.2f; %.2f]", d$LowerCI[i], d$UpperCI[i]), cex = 0.68)
            text(xw, y, sprintf("%.1f%%", overall$weights[i]), adj = 1, cex = 0.68)
        }
        if (z$type == "sum") {
            m <- models[[z$g]]
            polygon(c(clamp(exp(m$ci_low)), clamp(exp(m$mu)), clamp(exp(m$ci_high)), clamp(exp(m$mu))), c(y, y + 0.24, y, y - 0.24), col = "red", border = "black")
            text(xs, y, "Random effects model", adj = 0, font = 2, col = "#16836F", cex = 0.7)
            text(xe, y, sprintf("%.2f", exp(m$mu)), adj = 1, font = 2, col = "#16836F", cex = 0.7)
            text(xci, y, sprintf("[%.2f; %.2f]", exp(m$ci_low), exp(m$ci_high)), font = 2, col = "#16836F", cex = 0.7)
        }
        if (z$type == "het") {
            m <- models[[z$g]]
            text(xs, y, sprintf("Heterogeneity: I² = %.1f%%, τ² = %.4f, p %s", m$I2, m$tau2, fmt_p(m$Q_p)), adj = 0, col = "#16836F", cex = 0.65)
        }
    }
    py <- 0.15
    polygon(c(clamp(exp(overall$ci_low)), clamp(exp(overall$mu)), clamp(exp(overall$ci_high)), clamp(exp(overall$mu))), c(py, py + 0.22, py, py - 0.22), col = "red", border = "black")
    text(xs, py, "Overall random effects model", adj = 0, font = 2, cex = 0.74)
    text(xe, py, sprintf("%.2f", exp(overall$mu)), adj = 1, font = 2, cex = 0.74)
    text(xci, py, sprintf("[%.2f; %.2f]", exp(overall$ci_low), exp(overall$ci_high)), font = 2, cex = 0.74)
    text(xw, py, "100.0%", adj = 1, font = 2, cex = 0.74)
    text(xs, py - 0.65, sprintf("Overall heterogeneity: I² = %.1f%%, τ² = %.4f, p %s", overall$I2, overall$tau2, fmt_p(overall$Q_p)), adj = 0, cex = 0.64)
    vi <- d$sei^2
    tau2 <- overall$tau2
    w <- 1/(vi + tau2)
    g <- factor(d[[var]], levels = levels)
    X <- model.matrix(~g)
    A <- crossprod(X, w * X)
    bb <- solve(A, crossprod(X, w * d$yi))
    VV <- solve(A)
    Qm <- if (ncol(X) > 1)
        as.numeric(crossprod(bb[-1], solve(VV[-1, -1, drop = FALSE], bb[-1])))
    else NA
    df <- ncol(X) - 1
    pm <- if (df > 0)
        pchisq(Qm, df, lower.tail = FALSE)
    else NA
    text(xs, -1.55, sprintf("Test for subgroup differences: Qm = %.2f, df = %d (p = %.4f)", Qm, df, pm), adj = 0, cex = 0.62)
    text(xs, -1.9, "Random-effects model (REML; normal 95% CI). Subgroups with k < 3 are exploratory.", adj = 0, cex = 0.58, col = "#555555")
    ay <- -0.75
    segments(pl, ay, pr, ay)
    for (t in c(0.1, 0.5, 1, 2, 10)) {
        segments(map(t), ay, map(t), ay - 0.15)
        text(map(t), ay - 0.35, t, cex = 0.66)
    }
    text((pl + pr)/2, ay - 0.75, if (measure == "HR")
        endpoint
    else "Odds Ratio", cex = 0.72)
}
d<-prepare_effects(as.data.frame(embedded_input));subgroup_forest(d,'CancerType','Cancer type',"ORR","OR",'subgroup_CancerType.jpg');subgroup_forest(d,'StudyRegion','Study region',"ORR","OR",'subgroup_StudyRegion.jpg');subgroup_forest(d,'ICIClass','ICI class',"ORR","OR",'subgroup_ICIClass.jpg')
```

**Supplementary Code S22.**Subgroup analysis — unadjusted ORR

```
# Analysis: 22_subgroup_ORR_unadjusted
# All analysis data are embedded. Running this file creates figures only.
rm(list = ls())
options(stringsAsFactors = FALSE, scipen = 999)
analysis_id <- "22_subgroup_ORR_unadjusted"
script_arg <- grep('^--file=', commandArgs(trailingOnly = FALSE), value = TRUE)
script_file <- if (length(script_arg)) sub('^--file=', '', script_arg[1]) else NA_character_
if ((is.na(script_file) || !nzchar(script_file)) && interactive() && requireNamespace('rstudioapi', quietly = TRUE)) {
  p <- tryCatch(rstudioapi::getSourceEditorContext()$path, error = function(e) '')
  if (nzchar(p)) script_file <- p
}
script_dir <- if (!is.na(script_file) && nzchar(script_file)) dirname(normalizePath(script_file, winslash='/', mustWork=TRUE)) else normalizePath(getwd(), winslash='/', mustWork=TRUE)
output_dir <- file.path(script_dir, paste0(analysis_id, '_output'))
dir.create(output_dir, recursive=TRUE, showWarnings=FALSE)
setwd(output_dir)
.libPaths(unique(c(file.path(Sys.getenv('USERPROFILE'), 'R-library'), .libPaths())))
embedded_input <- structure(list(Study = c("Cortellini 2019", "Giorgi 2019", "Rogado 2020",
"Minami 2020", "Imai 2021", "Esposito 2021", "Chen 2022", "Ged 2022",
"Imai 2022", "Tateishi 2022", "Wasamoto 2023", "Incorvaia 2023",
"Vithayathil 2023", "McManus 2023", "Miyamoto 2023", "Bernard 2024",
"Nie 2024", "Şen 2024", "Yamamoto 2024", "Demir 2025", "Krejčí 2025",
"Anami 2025"), CancerType = c("Multiple/Other", "RCC", "Multiple/Other",
"NSCLC", "NSCLC", "Multiple/Other", "HCC", "RCC", "NSCLC", "NSCLC",
"Multiple/Other", "Multiple/Other", "HCC", "RCC", "HNSCC", "Multiple/Other",
"NSCLC", "Multiple/Other", "HCC", "NSCLC", "NSCLC", "RCC"), StudyRegion = c("Europe",
"Europe", "Europe", "Asia", "Asia", "Europe", "Asia", "USA",
"Asia", "Asia", "Asia", "Europe", "Others", "USA", "Asia", "USA",
"Others", "Europe", "Asia", "Europe", "Europe", "Asia"), ICIClass = c("Anti-PD-1/PD-L1",
"Anti-PD-1/PD-L1", "Anti-PD-1/PD-L1", "Anti-PD-1/PD-L1", "Anti-PD-1/PD-L1",
"Anti-PD-1/PD-L1", "Anti-PD-1/PD-L1", "Anti-PD-1/PD-L1 + Anti-CTLA-4",
"Anti-PD-1/PD-L1", "Anti-PD-1/PD-L1", "Anti-PD-1/PD-L1", "Anti-PD-1/PD-L1",
"Anti-PD-1/PD-L1", "Anti-PD-1/PD-L1 + Anti-CTLA-4", "Anti-PD-1/PD-L1",
"Anti-PD-1/PD-L1", "Anti-PD-1/PD-L1", "Anti-PD-1/PD-L1", "Anti-PD-1/PD-L1",
"Anti-PD-1/PD-L1", "Anti-PD-1/PD-L1", "Anti-PD-1/PD-L1 + Anti-CTLA-4"
), Effect = c(2.668, 1.168, 2.4, 1.06, 3.37, 0.93, 2.29, 0.89,
1.06, 0.87, 1.61, 0.48, 1.33, 0.364, 0.72, 3.2, 1.52, 0.84, 1.17,
0.98, 2.21, 1.4), LowerCI = c(1.99, 0.675, 0.81, 0.2, 1.65, 0.31,
0.6, 0.44, 0.39, 0.43, 0.61, 0.11, 0.65, 0.132, 0.32, 0.9, 0.7,
0.28, 0.35, 0.6, 1.09, 0.74), UpperCI = c(3.577, 2.022, 7.13,
5.55, 6.86, 2.79, 8.67, 1.8, 2.88, 1.73, 4.27, 2.04, 2.71, 1.005,
1.64, 12.3, 3.3, 2.56, 3.88, 1.6, 4.51, 2.62)), row.names = c(NA,
-22L), class = "data.frame")


reml_meta <- function (yi, sei)
{
    keep <- is.finite(yi) & is.finite(sei) & sei > 0
    yi <- yi[keep]
    sei <- sei[keep]
    vi <- sei^2
    k <- length(yi)
    if (!k)
        stop("No valid estimates.")
    if (k == 1L) {
        z <- qnorm(0.975)
        return(list(k = 1L, mu = yi, se_mu = sei, ci_low = yi - z * sei, ci_high = yi + z * sei, p = 2 * pnorm(abs(yi/sei), lower.tail = FALSE), tau2 = NA_real_, Q = NA_real_, df_Q = 0L, Q_p = NA_real_, I2 = NA_real_, weights = 100))
    }
    wf <- 1/vi
    muf <- sum(wf * yi)/sum(wf)
    Q <- sum(wf * (yi - muf)^2)
    dfQ <- k - 1
    I2 <- if (Q > 0)
        max(0, (Q - dfQ)/Q) * 100
    else 0
    obj <- function(tau2) {
        w <- 1/(vi + tau2)
        mu <- sum(w * yi)/sum(w)
        0.5 * (sum(log(vi + tau2)) + log(sum(w)) + sum(w * (yi - mu)^2))
    }
    upper <- max(1, var(yi) * 10, max(vi) * 10)
    opt <- optimize(obj, c(0, upper), tol = 1e-12)
    tau2 <- if (opt$minimum < 1e-10)
        0
    else opt$minimum
    w <- 1/(vi + tau2)
    mu <- sum(w * yi)/sum(w)
    se <- sqrt(1/sum(w))
    ci <- mu + c(-1, 1) * qnorm(0.975) * se
    list(k = k, mu = mu, se_mu = se, ci_low = ci[1], ci_high = ci[2], p = 2 * pnorm(abs(mu/se), lower.tail = FALSE), tau2 = tau2, Q = Q, df_Q = dfQ, Q_p = pchisq(Q, dfQ, lower.tail = FALSE), I2 = I2, weights = w/sum(w) * 100)
}
prepare_effects <- function (d, effect_col = "Effect")
{
    d[[effect_col]] <- as.numeric(d[[effect_col]])
    d$LowerCI <- as.numeric(d$LowerCI)
    d$UpperCI <- as.numeric(d$UpperCI)
    ok <- is.finite(d[[effect_col]]) & is.finite(d$LowerCI) & is.finite(d$UpperCI) & d[[effect_col]] > 0 & d$LowerCI > 0 & d$UpperCI > d$LowerCI
    d <- d[ok, , drop = FALSE]
    d$Effect <- d[[effect_col]]
    d$yi <- log(d$Effect)
    d$sei <- (log(d$UpperCI) - log(d$LowerCI))/(2 * qnorm(0.975))
    d
}
fmt_p <- function (p)
if (!is.finite(p)) "NR" else if (p < 1e-04) "< 0.0001" else paste0("= ", formatC(p, format = "f", digits = 4))
subgroup_forest <- function (d, var, label, endpoint, measure, file)
{
    preferred <- if (var == "CancerType")
        c("Melanoma", "NSCLC", "RCC", "HCC", "HNSCC", "Multiple/Other", "Multiple/other")
    else if (var == "StudyRegion")
        c("America", "United States", "Europe", "Asia", "Others", "Multiple regions")
    else c("Anti-PD-1/PD-L1", "Anti-CTLA-4", "Anti-PD-1/PD-L1 + Anti-CTLA-4")
    levels <- c(preferred[preferred %in% unique(d[[var]])], setdiff(unique(d[[var]]), preferred))
    rows <- list()
    models <- list()
    for (g in levels) {
        z <- d[d[[var]] == g, , drop = FALSE]
        m <- reml_meta(z$yi, z$sei)
        models[[g]] <- m
        rows[[length(rows) + 1]] <- data.frame(type = "title", g = g, i = NA)
        for (i in which(d[[var]] == g)) rows[[length(rows) + 1]] <- data.frame(type = "study", g = g, i = i)
        rows[[length(rows) + 1]] <- data.frame(type = "sum", g = g, i = NA)
        rows[[length(rows) + 1]] <- data.frame(type = "het", g = g, i = NA)
        rows[[length(rows) + 1]] <- data.frame(type = "space", g = g, i = NA)
    }
    rp <- do.call(rbind, rows)
    rp$y <- rev(seq_len(nrow(rp))) + 5
    overall <- reml_meta(d$yi, d$sei)
    h <- max(8, 3.7 + 0.255 * nrow(rp))
    jpeg(file, width = 11, height = h, units = "in", res = 300, quality = 100)
    on.exit(dev.off(), add = TRUE)
    par(mar = c(4.7, 0.65, 1.8, 0.65), family = "sans", xpd = NA)
    plot.new()
    plot.window(xlim = c(0, 1), ylim = c(-2.1, max(rp$y) + 3))
    xs <- 0.02
    xln <- 0.31
    xse <- 0.41
    pl <- 0.515
    pr <- 0.7
    xe <- 0.775
    xci <- 0.89
    xw <- 0.98
    amin <- 0.1
    amax <- 10
    map <- function(v) pl + (log(v) - log(amin))/(log(amax) - log(amin)) * (pr - pl)
    clamp <- function(v) pmin(pr, pmax(pl, map(v)))
    hy <- max(rp$y) + 2
    text(xs, hy, "Subgroup / Study", adj = 0, font = 2, cex = 0.84)
    text(xln, hy, paste0("log", measure), adj = 1, font = 2, cex = 0.84)
    text(xse, hy, paste0("SE(log", measure, ")"), adj = 1, font = 2, cex = 0.84)
    text((pl + pr)/2, hy, if (measure == "HR")
        "Hazard Ratio"
    else "Odds Ratio", font = 2, cex = 0.84)
    text(xe, hy, measure, adj = 1, font = 2, cex = 0.84)
    text(xci, hy, "95% CI", font = 2, cex = 0.84)
    text(xw, hy, "Weight", adj = 1, font = 2, cex = 0.84)
    segments(map(1), -0.4, map(1), max(rp$y) + 1.2)
    segments(clamp(exp(overall$mu)), -0.4, clamp(exp(overall$mu)), max(rp$y) + 1.2, lty = 3)
    for (r in seq_len(nrow(rp))) {
        z <- rp[r, ]
        y <- z$y
        if (z$type == "title")
            text(xs, y, paste0(z$g, " (k = ", sum(d[[var]] == z$g), ")"), adj = 0, font = 2, col = "#16836F", cex = 0.8)
        if (z$type == "study") {
            i <- z$i
            segments(clamp(d$LowerCI[i]), y, clamp(d$UpperCI[i]), y)
            if (d$LowerCI[i] < amin)
                arrows(pl + 0.012, y, pl, y, length = 0.04)
            if (d$UpperCI[i] > amax)
                arrows(pr - 0.012, y, pr, y, length = 0.04)
            points(clamp(d$Effect[i]), y, pch = 15, col = "#0A4F78", cex = 0.55 + 1.1 * sqrt(overall$weights[i]/max(overall$weights)))
            text(xs, y, d$Study[i], adj = 0, cex = 0.68)
            text(xln, y, formatC(d$yi[i], format = "f", digits = 4), adj = 1, cex = 0.65)
            text(xse, y, formatC(d$sei[i], format = "f", digits = 4), adj = 1, cex = 0.65)
            text(xe, y, sprintf("%.2f", d$Effect[i]), adj = 1, cex = 0.68)
            text(xci, y, sprintf("[%.2f; %.2f]", d$LowerCI[i], d$UpperCI[i]), cex = 0.68)
            text(xw, y, sprintf("%.1f%%", overall$weights[i]), adj = 1, cex = 0.68)
        }
        if (z$type == "sum") {
            m <- models[[z$g]]
            polygon(c(clamp(exp(m$ci_low)), clamp(exp(m$mu)), clamp(exp(m$ci_high)), clamp(exp(m$mu))), c(y, y + 0.24, y, y - 0.24), col = "red", border = "black")
            text(xs, y, "Random effects model", adj = 0, font = 2, col = "#16836F", cex = 0.7)
            text(xe, y, sprintf("%.2f", exp(m$mu)), adj = 1, font = 2, col = "#16836F", cex = 0.7)
            text(xci, y, sprintf("[%.2f; %.2f]", exp(m$ci_low), exp(m$ci_high)), font = 2, col = "#16836F", cex = 0.7)
        }
        if (z$type == "het") {
            m <- models[[z$g]]
            text(xs, y, sprintf("Heterogeneity: I² = %.1f%%, τ² = %.4f, p %s", m$I2, m$tau2, fmt_p(m$Q_p)), adj = 0, col = "#16836F", cex = 0.65)
        }
    }
    py <- 0.15
    polygon(c(clamp(exp(overall$ci_low)), clamp(exp(overall$mu)), clamp(exp(overall$ci_high)), clamp(exp(overall$mu))), c(py, py + 0.22, py, py - 0.22), col = "red", border = "black")
    text(xs, py, "Overall random effects model", adj = 0, font = 2, cex = 0.74)
    text(xe, py, sprintf("%.2f", exp(overall$mu)), adj = 1, font = 2, cex = 0.74)
    text(xci, py, sprintf("[%.2f; %.2f]", exp(overall$ci_low), exp(overall$ci_high)), font = 2, cex = 0.74)
    text(xw, py, "100.0%", adj = 1, font = 2, cex = 0.74)
    text(xs, py - 0.65, sprintf("Overall heterogeneity: I² = %.1f%%, τ² = %.4f, p %s", overall$I2, overall$tau2, fmt_p(overall$Q_p)), adj = 0, cex = 0.64)
    vi <- d$sei^2
    tau2 <- overall$tau2
    w <- 1/(vi + tau2)
    g <- factor(d[[var]], levels = levels)
    X <- model.matrix(~g)
    A <- crossprod(X, w * X)
    bb <- solve(A, crossprod(X, w * d$yi))
    VV <- solve(A)
    Qm <- if (ncol(X) > 1)
        as.numeric(crossprod(bb[-1], solve(VV[-1, -1, drop = FALSE], bb[-1])))
    else NA
    df <- ncol(X) - 1
    pm <- if (df > 0)
        pchisq(Qm, df, lower.tail = FALSE)
    else NA
    text(xs, -1.55, sprintf("Test for subgroup differences: Qm = %.2f, df = %d (p = %.4f)", Qm, df, pm), adj = 0, cex = 0.62)
    text(xs, -1.9, "Random-effects model (REML; normal 95% CI). Subgroups with k < 3 are exploratory.", adj = 0, cex = 0.58, col = "#555555")
    ay <- -0.75
    segments(pl, ay, pr, ay)
    for (t in c(0.1, 0.5, 1, 2, 10)) {
        segments(map(t), ay, map(t), ay - 0.15)
        text(map(t), ay - 0.35, t, cex = 0.66)
    }
    text((pl + pr)/2, ay - 0.75, if (measure == "HR")
        endpoint
    else "Odds Ratio", cex = 0.72)
}
d<-prepare_effects(as.data.frame(embedded_input));subgroup_forest(d,'CancerType','Cancer type',"ORR","OR",'subgroup_CancerType.jpg');subgroup_forest(d,'StudyRegion','Study region',"ORR","OR",'subgroup_StudyRegion.jpg');subgroup_forest(d,'ICIClass','ICI class',"ORR","OR",'subgroup_ICIClass.jpg')
```

**Supplementary Code S23.**Subgroup analysis — unadjusted any-grade irAEs

```
# Analysis: 23_subgroup_any_grade_irAEs_unadjusted
# All analysis data are embedded. Running this file creates figures only.
rm(list = ls())
options(stringsAsFactors = FALSE, scipen = 999)
analysis_id <- "23_subgroup_any_grade_irAEs_unadjusted"
script_arg <- grep('^--file=', commandArgs(trailingOnly = FALSE), value = TRUE)
script_file <- if (length(script_arg)) sub('^--file=', '', script_arg[1]) else NA_character_
if ((is.na(script_file) || !nzchar(script_file)) && interactive() && requireNamespace('rstudioapi', quietly = TRUE)) {
  p <- tryCatch(rstudioapi::getSourceEditorContext()$path, error = function(e) '')
  if (nzchar(p)) script_file <- p
}
script_dir <- if (!is.na(script_file) && nzchar(script_file)) dirname(normalizePath(script_file, winslash='/', mustWork=TRUE)) else normalizePath(getwd(), winslash='/', mustWork=TRUE)
output_dir <- file.path(script_dir, paste0(analysis_id, '_output'))
dir.create(output_dir, recursive=TRUE, showWarnings=FALSE)
setwd(output_dir)
.libPaths(unique(c(file.path(Sys.getenv('USERPROFILE'), 'R-library'), .libPaths())))
embedded_input <- structure(list(Study = c("Cortellini 2019", "Kichenadasse 2019",
"Rogado 2020", "Bartl 2021", "Collet 2021", "Esposito 2021",
"Chen 2022", "Vithayathil 2023", "Zhang 2023", "Gómez-Banoy 2024",
"Şen 2024", "Anami 2025"), CancerType = c("Multiple/Other",
"NSCLC", "Multiple/Other", "Multiple/Other", "Multiple/Other",
"Multiple/Other", "HCC", "HCC", "HNSCC", "Multiple/Other", "Multiple/Other",
"RCC"), StudyRegion = c("Europe", "Others", "Europe", "Europe",
"Europe", "Europe", "Asia", "Others", "Asia", "USA", "Europe",
"Asia"), ICIClass = c("Anti-PD-1/PD-L1", "Anti-PD-1/PD-L1", "Anti-PD-1/PD-L1",
"Anti-PD-1/PD-L1", "Anti-PD-1/PD-L1 + Anti-CTLA-4", "Anti-PD-1/PD-L1",
"Anti-PD-1/PD-L1", "Anti-PD-1/PD-L1", "Anti-PD-1/PD-L1", "Anti-PD-1/PD-L1",
"Anti-PD-1/PD-L1", "Anti-PD-1/PD-L1 + Anti-CTLA-4"), Effect = c(3.713,
1.34, 1.42, 1.31, 1.37, 0.76, 0.54, 1.01, 2.48, 1.87, 2.28, 1.12
), LowerCI = c(2.831, 0.97, 0.68, 0.23, 0.83, 0.32, 0.14, 0.57,
0.49, 1.21, 0.91, 0.63), UpperCI = c(4.87, 1.85, 2.97, 7.57,
2.28, 1.78, 2.02, 1.8, 12.59, 2.91, 5.71, 1.99)), row.names = c(NA,
-12L), class = "data.frame")


reml_meta <- function (yi, sei)
{
    keep <- is.finite(yi) & is.finite(sei) & sei > 0
    yi <- yi[keep]
    sei <- sei[keep]
    vi <- sei^2
    k <- length(yi)
    if (!k)
        stop("No valid estimates.")
    if (k == 1L) {
        z <- qnorm(0.975)
        return(list(k = 1L, mu = yi, se_mu = sei, ci_low = yi - z * sei, ci_high = yi + z * sei, p = 2 * pnorm(abs(yi/sei), lower.tail = FALSE), tau2 = NA_real_, Q = NA_real_, df_Q = 0L, Q_p = NA_real_, I2 = NA_real_, weights = 100))
    }
    wf <- 1/vi
    muf <- sum(wf * yi)/sum(wf)
    Q <- sum(wf * (yi - muf)^2)
    dfQ <- k - 1
    I2 <- if (Q > 0)
        max(0, (Q - dfQ)/Q) * 100
    else 0
    obj <- function(tau2) {
        w <- 1/(vi + tau2)
        mu <- sum(w * yi)/sum(w)
        0.5 * (sum(log(vi + tau2)) + log(sum(w)) + sum(w * (yi - mu)^2))
    }
    upper <- max(1, var(yi) * 10, max(vi) * 10)
    opt <- optimize(obj, c(0, upper), tol = 1e-12)
    tau2 <- if (opt$minimum < 1e-10)
        0
    else opt$minimum
    w <- 1/(vi + tau2)
    mu <- sum(w * yi)/sum(w)
    se <- sqrt(1/sum(w))
    ci <- mu + c(-1, 1) * qnorm(0.975) * se
    list(k = k, mu = mu, se_mu = se, ci_low = ci[1], ci_high = ci[2], p = 2 * pnorm(abs(mu/se), lower.tail = FALSE), tau2 = tau2, Q = Q, df_Q = dfQ, Q_p = pchisq(Q, dfQ, lower.tail = FALSE), I2 = I2, weights = w/sum(w) * 100)
}
prepare_effects <- function (d, effect_col = "Effect")
{
    d[[effect_col]] <- as.numeric(d[[effect_col]])
    d$LowerCI <- as.numeric(d$LowerCI)
    d$UpperCI <- as.numeric(d$UpperCI)
    ok <- is.finite(d[[effect_col]]) & is.finite(d$LowerCI) & is.finite(d$UpperCI) & d[[effect_col]] > 0 & d$LowerCI > 0 & d$UpperCI > d$LowerCI
    d <- d[ok, , drop = FALSE]
    d$Effect <- d[[effect_col]]
    d$yi <- log(d$Effect)
    d$sei <- (log(d$UpperCI) - log(d$LowerCI))/(2 * qnorm(0.975))
    d
}
fmt_p <- function (p)
if (!is.finite(p)) "NR" else if (p < 1e-04) "< 0.0001" else paste0("= ", formatC(p, format = "f", digits = 4))
subgroup_forest <- function (d, var, label, endpoint, measure, file)
{
    preferred <- if (var == "CancerType")
        c("Melanoma", "NSCLC", "RCC", "HCC", "HNSCC", "Multiple/Other", "Multiple/other")
    else if (var == "StudyRegion")
        c("America", "United States", "Europe", "Asia", "Others", "Multiple regions")
    else c("Anti-PD-1/PD-L1", "Anti-CTLA-4", "Anti-PD-1/PD-L1 + Anti-CTLA-4")
    levels <- c(preferred[preferred %in% unique(d[[var]])], setdiff(unique(d[[var]]), preferred))
    rows <- list()
    models <- list()
    for (g in levels) {
        z <- d[d[[var]] == g, , drop = FALSE]
        m <- reml_meta(z$yi, z$sei)
        models[[g]] <- m
        rows[[length(rows) + 1]] <- data.frame(type = "title", g = g, i = NA)
        for (i in which(d[[var]] == g)) rows[[length(rows) + 1]] <- data.frame(type = "study", g = g, i = i)
        rows[[length(rows) + 1]] <- data.frame(type = "sum", g = g, i = NA)
        rows[[length(rows) + 1]] <- data.frame(type = "het", g = g, i = NA)
        rows[[length(rows) + 1]] <- data.frame(type = "space", g = g, i = NA)
    }
    rp <- do.call(rbind, rows)
    rp$y <- rev(seq_len(nrow(rp))) + 5
    overall <- reml_meta(d$yi, d$sei)
    h <- max(8, 3.7 + 0.255 * nrow(rp))
    jpeg(file, width = 11, height = h, units = "in", res = 300, quality = 100)
    on.exit(dev.off(), add = TRUE)
    par(mar = c(4.7, 0.65, 1.8, 0.65), family = "sans", xpd = NA)
    plot.new()
    plot.window(xlim = c(0, 1), ylim = c(-2.1, max(rp$y) + 3))
    xs <- 0.02
    xln <- 0.31
    xse <- 0.41
    pl <- 0.515
    pr <- 0.7
    xe <- 0.775
    xci <- 0.89
    xw <- 0.98
    amin <- 0.1
    amax <- 10
    map <- function(v) pl + (log(v) - log(amin))/(log(amax) - log(amin)) * (pr - pl)
    clamp <- function(v) pmin(pr, pmax(pl, map(v)))
    hy <- max(rp$y) + 2
    text(xs, hy, "Subgroup / Study", adj = 0, font = 2, cex = 0.84)
    text(xln, hy, paste0("log", measure), adj = 1, font = 2, cex = 0.84)
    text(xse, hy, paste0("SE(log", measure, ")"), adj = 1, font = 2, cex = 0.84)
    text((pl + pr)/2, hy, if (measure == "HR")
        "Hazard Ratio"
    else "Odds Ratio", font = 2, cex = 0.84)
    text(xe, hy, measure, adj = 1, font = 2, cex = 0.84)
    text(xci, hy, "95% CI", font = 2, cex = 0.84)
    text(xw, hy, "Weight", adj = 1, font = 2, cex = 0.84)
    segments(map(1), -0.4, map(1), max(rp$y) + 1.2)
    segments(clamp(exp(overall$mu)), -0.4, clamp(exp(overall$mu)), max(rp$y) + 1.2, lty = 3)
    for (r in seq_len(nrow(rp))) {
        z <- rp[r, ]
        y <- z$y
        if (z$type == "title")
            text(xs, y, paste0(z$g, " (k = ", sum(d[[var]] == z$g), ")"), adj = 0, font = 2, col = "#16836F", cex = 0.8)
        if (z$type == "study") {
            i <- z$i
            segments(clamp(d$LowerCI[i]), y, clamp(d$UpperCI[i]), y)
            if (d$LowerCI[i] < amin)
                arrows(pl + 0.012, y, pl, y, length = 0.04)
            if (d$UpperCI[i] > amax)
                arrows(pr - 0.012, y, pr, y, length = 0.04)
            points(clamp(d$Effect[i]), y, pch = 15, col = "#0A4F78", cex = 0.55 + 1.1 * sqrt(overall$weights[i]/max(overall$weights)))
            text(xs, y, d$Study[i], adj = 0, cex = 0.68)
            text(xln, y, formatC(d$yi[i], format = "f", digits = 4), adj = 1, cex = 0.65)
            text(xse, y, formatC(d$sei[i], format = "f", digits = 4), adj = 1, cex = 0.65)
            text(xe, y, sprintf("%.2f", d$Effect[i]), adj = 1, cex = 0.68)
            text(xci, y, sprintf("[%.2f; %.2f]", d$LowerCI[i], d$UpperCI[i]), cex = 0.68)
            text(xw, y, sprintf("%.1f%%", overall$weights[i]), adj = 1, cex = 0.68)
        }
        if (z$type == "sum") {
            m <- models[[z$g]]
            polygon(c(clamp(exp(m$ci_low)), clamp(exp(m$mu)), clamp(exp(m$ci_high)), clamp(exp(m$mu))), c(y, y + 0.24, y, y - 0.24), col = "red", border = "black")
            text(xs, y, "Random effects model", adj = 0, font = 2, col = "#16836F", cex = 0.7)
            text(xe, y, sprintf("%.2f", exp(m$mu)), adj = 1, font = 2, col = "#16836F", cex = 0.7)
            text(xci, y, sprintf("[%.2f; %.2f]", exp(m$ci_low), exp(m$ci_high)), font = 2, col = "#16836F", cex = 0.7)
        }
        if (z$type == "het") {
            m <- models[[z$g]]
            text(xs, y, sprintf("Heterogeneity: I² = %.1f%%, τ² = %.4f, p %s", m$I2, m$tau2, fmt_p(m$Q_p)), adj = 0, col = "#16836F", cex = 0.65)
        }
    }
    py <- 0.15
    polygon(c(clamp(exp(overall$ci_low)), clamp(exp(overall$mu)), clamp(exp(overall$ci_high)), clamp(exp(overall$mu))), c(py, py + 0.22, py, py - 0.22), col = "red", border = "black")
    text(xs, py, "Overall random effects model", adj = 0, font = 2, cex = 0.74)
    text(xe, py, sprintf("%.2f", exp(overall$mu)), adj = 1, font = 2, cex = 0.74)
    text(xci, py, sprintf("[%.2f; %.2f]", exp(overall$ci_low), exp(overall$ci_high)), font = 2, cex = 0.74)
    text(xw, py, "100.0%", adj = 1, font = 2, cex = 0.74)
    text(xs, py - 0.65, sprintf("Overall heterogeneity: I² = %.1f%%, τ² = %.4f, p %s", overall$I2, overall$tau2, fmt_p(overall$Q_p)), adj = 0, cex = 0.64)
    vi <- d$sei^2
    tau2 <- overall$tau2
    w <- 1/(vi + tau2)
    g <- factor(d[[var]], levels = levels)
    X <- model.matrix(~g)
    A <- crossprod(X, w * X)
    bb <- solve(A, crossprod(X, w * d$yi))
    VV <- solve(A)
    Qm <- if (ncol(X) > 1)
        as.numeric(crossprod(bb[-1], solve(VV[-1, -1, drop = FALSE], bb[-1])))
    else NA
    df <- ncol(X) - 1
    pm <- if (df > 0)
        pchisq(Qm, df, lower.tail = FALSE)
    else NA
    text(xs, -1.55, sprintf("Test for subgroup differences: Qm = %.2f, df = %d (p = %.4f)", Qm, df, pm), adj = 0, cex = 0.62)
    text(xs, -1.9, "Random-effects model (REML; normal 95% CI). Subgroups with k < 3 are exploratory.", adj = 0, cex = 0.58, col = "#555555")
    ay <- -0.75
    segments(pl, ay, pr, ay)
    for (t in c(0.1, 0.5, 1, 2, 10)) {
        segments(map(t), ay, map(t), ay - 0.15)
        text(map(t), ay - 0.35, t, cex = 0.66)
    }
    text((pl + pr)/2, ay - 0.75, if (measure == "HR")
        endpoint
    else "Odds Ratio", cex = 0.72)
}
d<-prepare_effects(as.data.frame(embedded_input));subgroup_forest(d,'CancerType','Cancer type',"Any-grade irAEs","OR",'subgroup_CancerType.jpg');subgroup_forest(d,'StudyRegion','Study region',"Any-grade irAEs","OR",'subgroup_StudyRegion.jpg');subgroup_forest(d,'ICIClass','ICI class',"Any-grade irAEs","OR",'subgroup_ICIClass.jpg')
```
